# Solvophobic Effect-Driven C4-Selective C–H Oxidation of Diverse Alkyl Substrates by a Macrocyclic Supramolecular Catalyst

**DOI:** 10.1021/jacs.6c08837

**Published:** 2026-07-31

**Authors:** Kanstantsin Anisovich, Yiheng Lu, Bernd Goldfuss, Konrad Tiefenbacher

**Affiliations:** † Department of Chemistry, 27209 University of Basel, Mattenstrasse 22, Basel 4058, Switzerland; ‡ Institute of Organic Chemistry, University of Cologne, Greinstrasse 4, Cologne 50939, Germany; § Department of Biosystems Science and Engineering, ETH Zurich, Klingelbergstrasse 48, Basel 4056, Switzerland

## Abstract

The selective oxidation of inert aliphatic C–H bonds remains a formidable challenge in organic synthesis, particularly at medial positions in substrates lacking directing groups. We report a macrocyclic supramolecular manganese catalyst that achieves C4-site-selective C–H oxidation across a broad substrate range by harnessing the solvophobic effect for substrate binding. It is effective on alkanes as well as more complex molecules containing functional groups such as chlorides, nitriles, esters, and protected amines, underscoring its potential for late-stage functionalization. Notably, the catalyst also provides moderate enantioselectivity, a rare feat on unfunctionalized alkanes. This macrocyclic platform offers a blueprint for the rational design of next-generation supramolecular catalysts with tunable site selectivity for C–H functionalization.

## Graphical abstract

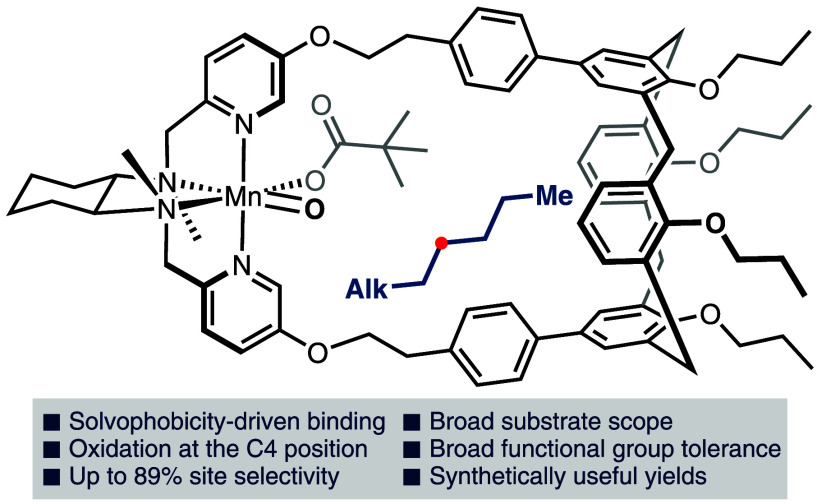

## Introduction

The oxidation of aliphatic C–H bonds represents a transformative strategy in organic synthesis, enabling the direct functionalization of bonds traditionally considered inert. Given their exceptional strength, both heterolytic (p*K*_a_ ≥ 50)^1^ and homolytic (bond dissociation energies approaching 100 kcal/mol),^2^ achieving selective oxidation at specific sites remains a formidable challenge. Nevertheless, chemists have devised creative strategies that allow for selective oxidation at certain sites. Selective oxidation of a specific C–H bond within a molecule can often be achieved and predicted by electronic, steric, or stereoelectronic influences,^3–7^ as shown in 2007.^8^ By contrast, oxidation at less reactive positions remains challenging. Although a sterically more accessible site might be targeted using a sterically restricted catalyst,^5^ selective oxidation typically depends on a nearby functional group to direct the reaction.^9–13^ One promising strategy to achieve site-selective C–H oxidation is catalyst-directed oxidation.^14–22^ In this approach, the catalyst is equipped with a recognition motif that binds the substrate and orients it in a defined geometry toward the oxidant, thereby enabling selective functionalization. A major drawback of this method, however, is its dependence on the presence of a suitable functional group within the substrate to mediate binding to the catalyst. Even in the broader field of C–H functionalization,^23–25^ site-selective functionalization of alkanes remains a major challenge,^26^ especially at medial positions.^27–30^

Recently, our group demonstrated that the solvophobic effect in fluorinated alcohol solvents^31^ can be exploited to achieve catalyst–substrate binding without the need for specific functional groups being present on the substrate.^32^ It was shown by the Diederich group that the solvophobic effect in trifluoroethanol (TFE) is very close to the hydrophobic effect in water.^31^ A strong relationship has been reported between the empirical solvent polarity parameter *E*_T_ and the binding affinity of nonpolar guests. TFE (*E*_T_^N^ = 0.898) possesses a polarity approaching that of water (*E*_T_^N^ = 1.00), whereas 1,1,1,3,3,3-hexafluoro-2-propanol (HFIP), another commonly used solvent for C–H oxidation reactions, exhibits solvophobic properties that are even more comparable to those of water. As a result, the incorporation of apolar substrates into suitable binding pockets is favored in fluorinated alcohols, mirroring the hydrophobic interactions observed in aqueous solution. A suitable recognition motif, a resorcin[4]­arene derivative,^33,34^ was attached to the state-of-the-art tetradentate iron and manganese aminopyridine complexes (**1**–**2**, Figure 1a),^6,35–37^ to provide the supramolecular catalyst **3**. Its use enabled the preferential oxidation of C–H bonds at the fifth position from the less hindered side of aliphatic substrates (red dot, Figure 1a).

**Figure 1:**
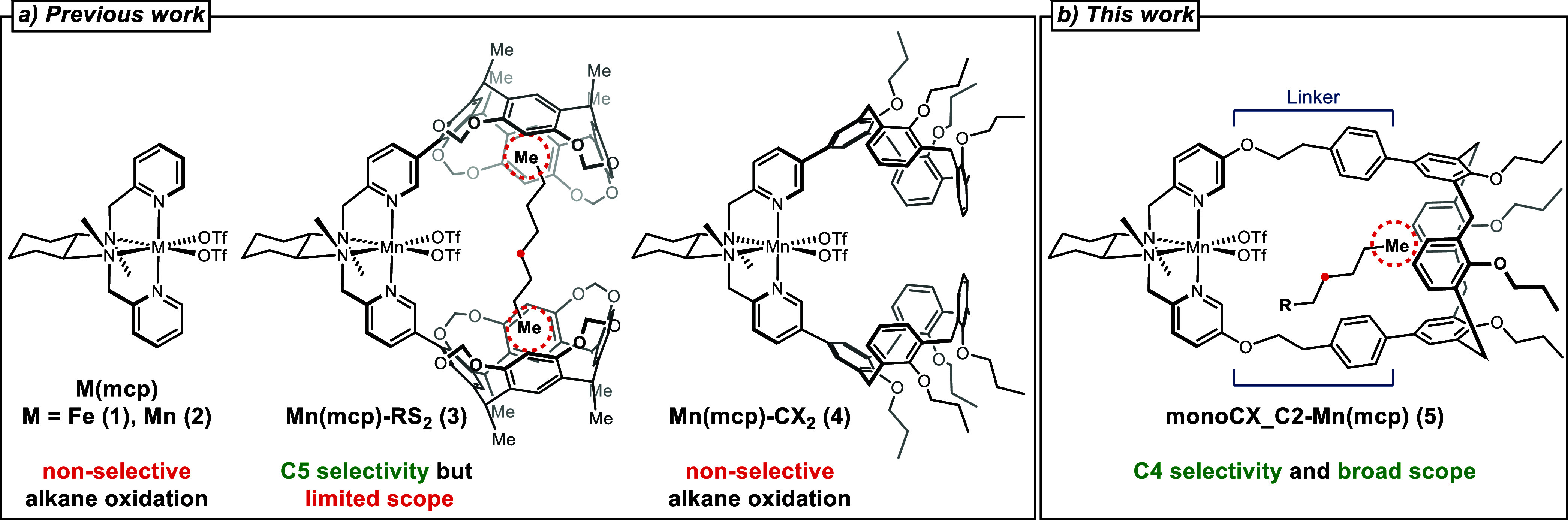
Previously reported C–H oxidation catalysts exhibiting different site selectivities for alkyl chain oxidation (a), and the catalyst developed in this work (b).

However, the catalyst exhibited promising site selectivity of 79% only for octane, which is optimally suited to interact with both recognition motifs. In contrast, other alkyl substrates displayed significantly lower selectivities, typically in the range of 40–60%. Catalyst **4** (Figure 1a), which features the same topology but incorporates the calix[4]­arene recognition motif,^38^ did not show any difference in selectivity compared to the unfunctionalized catalyst **2**. This lack of selectivity likely arises from the greater conformational flexibility and smaller cavity size of the calix[4]­arene.

To overcome this limitation, we introduce the novel, preorganized macrocyclic catalyst design **5** (Figure 1b). Preorganization of the host is well-established to not only enhance guest binding strength but, more importantly, to improve selectivity.^39^ Although the calix[4]­arene recognition motif proved unsuccessful in the open catalyst design **4**, we chose to explore it in a macrocyclic framework because the required difunctionalized derivative is readily available.^40^ In this case, the preorganized macrocyclic binding pocket led to markedly improved selectivity. Furthermore, as substrate binding relies on interactions with only one end of the substrate, the approach offers a considerably broader substrate scope. In contrast to our previous catalyst **3**, which preferentially oxidized the fifth carbon from the less hindered terminus, this new catalyst targets the fourth carbon, thereby delivering both enhanced and complementary site selectivity.

## Results and Discussion

The synthesis of the macrocyclic catalyst **5** (Scheme 1) started with the literature-known iridium-catalyzed C–H borylation of calix[4]­arene derivative **6**.^40^ The reaction proceeds in a regioselective, distal manner, affording the diborylated isomer **7** in 53% isolated yield. Etherification between phenol **8** and bromide **9** afforded the linker precursor **10**, which was subsequently coupled to calix[4]­arene **7** through a double Suzuki reaction. The formyl groups were then converted into benzylic bromides through reduction, followed by Appel bromination to deliver **11**. Remarkably, macrocyclization under standard conditions^32^ afforded ligand **12** in good yield (57%), despite the inherent risk of undesired oligomerization. Complexation with manganese bis­(trifluoromethanesulfonate) proceeded in nearly quantitative yield, affording the target catalyst **5**. Overall, catalyst **5** was obtained in 8.5% yield over seven linear steps from commercially available calix[4]­arene, the direct precursor of calix[4]­arene derivative **6** (see SI, sections 2.1 and 2.2 for more details on synthesis).

**Scheme 1:**
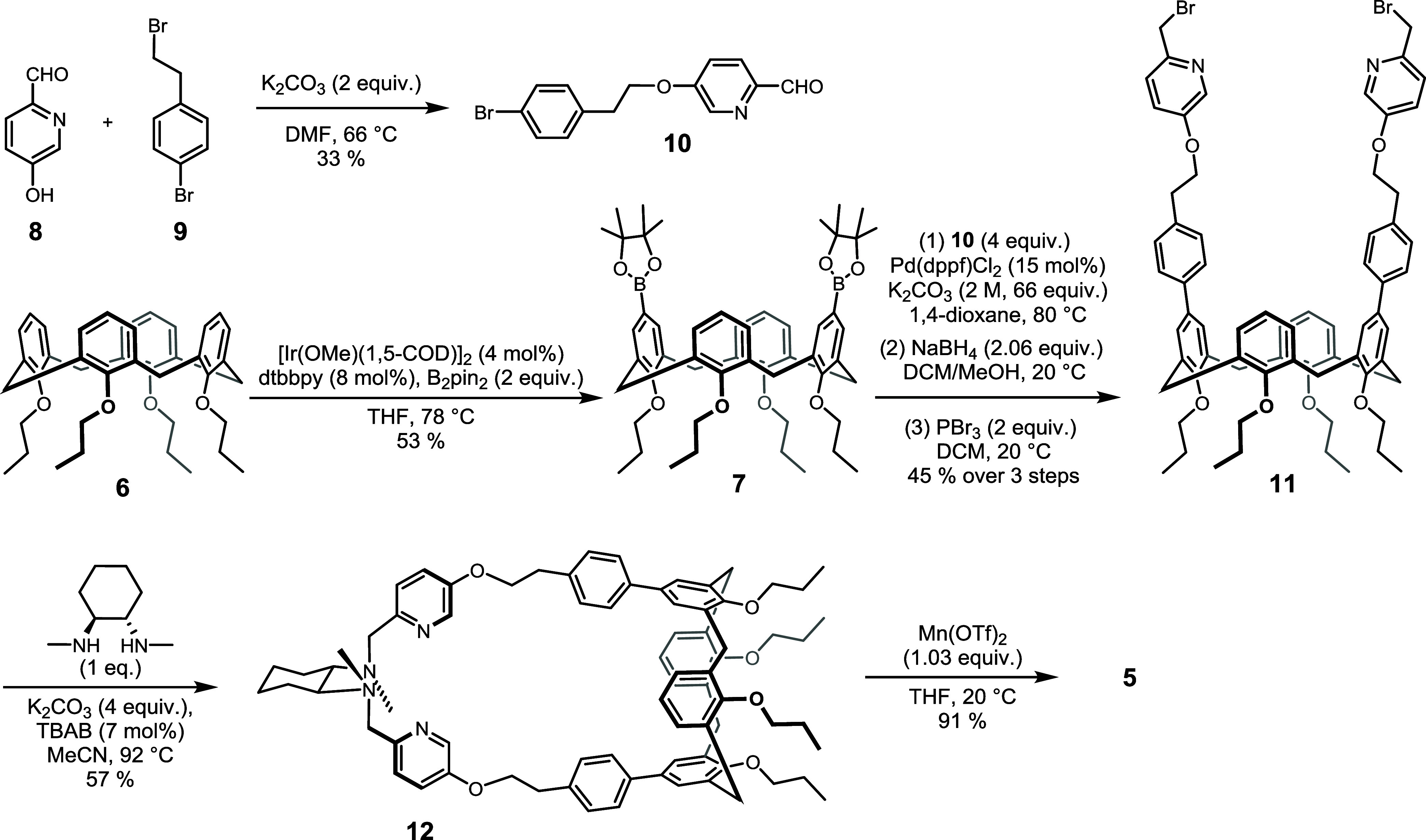
Synthesis of the Macrocyclic Catalyst 5

With the supramolecular catalyst in hand, octane was selected as a model substrate for the C–H oxidation studies (Table 1). Since the reaction generally produced a mixture of alcohols and ketones, the crude mixture was treated with IBX to convert all alcohols to ketones, simplifying analysis and quantification. Conversion and product yields were determined by gas chromatography (GC) (see SI, section 4.1).

**Table 1:**
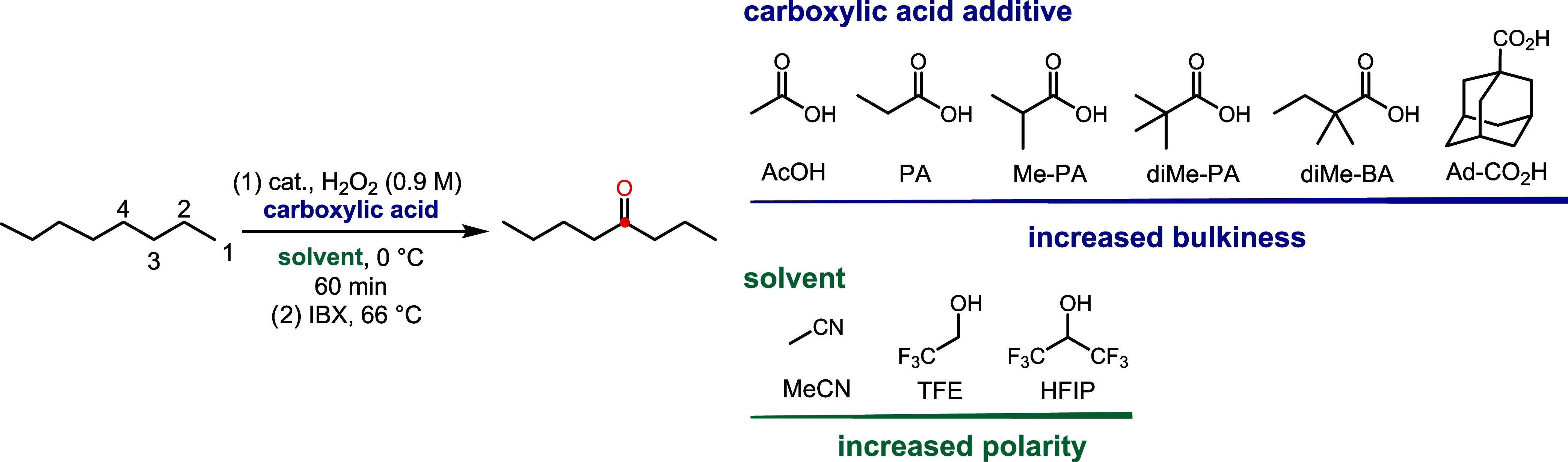
Optimization of the Reaction Conditions^g^

						site selectivity^b^, %		
entry	cat. (mol %)	H_2_O_2_ addition, equiv/min	solvent	carboxylic acid (equiv)	conversion/total yield (C4 yield)^a^, %	C4	C3	C2
0	**2** (1)	1/15	MeCN	AcOH (22)	98/46 (13)	28	27	45
**solvents**								
1	**5** (1)	1/15	MeCN	AcOH (22)	94/19 (5)	26	26	48
2	**5** (1)	1/15	TFE	AcOH (22)	66/21 (9)	**42**	25	33
3	**5** (1)	1/15	HFIP	AcOH (22)	68/44 (16)	36	28	36
**carboxylic acids**								
4	**5** (1)	1/15	TFE	PA (22)	62/26 (15)	60	18	22
5	**5** (1)	1/15	TFE	Me-PA (22)	58/23 (16)	70	15	15
6	**5** (1)	1/15	TFE	diMe-PA (22)	70/40 (32)	80	11	9
7	**5** (1)	1/15	TFE	diMe-BA (22)	56/17 (15)	86	8	6
8	**5** (1)	1/15	TFE	diMe-BA (5)	75/34 (23)	87	6	7
9	**5** (1)	1/15	TFE	diMe-BA (1)	75/42 (38)	**90**	8	2
10	**5** (1)	1/15	TFE	Ad-CO_2_H (1)	46/16 (13)	85	11	4
**reagents ratio and addition pattern**								
11	**5** (1)	2/30	TFE	diMe-BA (1)	75/40 (36)	90	8	2
12	**5** (2)	1/15	TFE	diMe-BA (1)	72/31 (26)	86	6	8
13	**5** (2)	2/30	TFE	diMe-BA (1)	73/42 (36)	87	9	4
14^c^	**5** (1 + 1)	1 + 1/15 + 15	TFE	diMe-BA (1)	94/55 (50)	91	7	2
15^c^	**5** (1 + 1)	1 + 1/15 + 15	TFE	diMe-PA (1)	98/**67** (59)	**89** ^d^	6	5
**control experiments**								
16	**2** (1)	1/15	TFE	diMe-PA (1)	83/35 (11)	31^e^	29	40
17^f^	**2** (1)	1/15	TFE	diMe-PA (1)	90/40 (13)	32	28	40
18	**4** (1)	1/15	TFE	diMe-PA (1)	69/34 (11)	32	30	38

a Conversion calculated based on the remaining substrate. GC total yield corresponds to the total of all monoketones and tertiary alcohols after IBX oxidation. Yield for targeted 4-octanone (C4 yield) is calculated by multiplication of total yield and C4 selectivity. For example, entry 0:0.46 × 0.29 × 100%=13%.

b Site selectivity at carbon (Cx) is defined as the GC yield of a specific ketone (corrected using the calculated response factor) divided by the total GC yield of all monoketones.

c Additional portion of catalyst (1 mol %) and H_2_O_2_ (0.9 M, 1 equiv, 20.56 μL, 1.37 μL/min) were added after 30 min.

d *ee* = 39%

e *ee* < 5%.

f Calix­[4]­arene **6** (1 mol %) was added to the reaction mixture. AcOH = acetic acid, PA = propanoic acid, Me-PA = 2-methylpropanoic acid, diMe-PA = 2,2-dimethylpropanoic acid, diMe-BA = 2,2-dimethylbutanoic acid, Ad-CO_2_H = adamantane-1-carboxylic acid; MeCN = acetonitrile, TFE = 2,2,2-trifluoroethanol; and HFIP = 1,1,1,3,3,3-hexafluoro-2-propanol.

g General reaction conditions: octane (1 equiv), catalyst (1 mol %), H_2_O_2_ (0.9 M, 1 equiv., 20.56 μL, 1.37 μL/min), carboxylic acid, solvent (0.0925 M), 0 °C, 1 h.

Reaction optimization focused on solvent polarity, carboxylic acid bulkiness, and reagent stoichiometry (see SI, sections 3.1 and 3.2). Because our concept of alkane binding relies on the solvophobic effect,^32^ solvent polarity was examined first. Three solvents of increasing polarity^41^ (entries 1–3) were tested: acetonitrile, 2,2,2-trifluoroethanol (TFE), and 1,1,1,3,3,3-hexafluoro-2-propanol (HFIP), solvents of choice for C–H oxidation.^42,43^ As expected, no improvement in selectivity was observed in the low-polarity solvent acetonitrile (entry 1) compared to the reference catalyst **2** in the same solvent (entry 0).

In contrast, both fluorinated alcohols, TFE (entry 2) and HFIP (entry 3), promoted C4-selective oxidation of octane, with TFE providing slightly higher selectivity (42 vs 36%). Accordingly, TFE was selected as the solvent for all of the subsequent studies.

A second key additive in this reaction is a carboxylic acid. It promotes the catalytic cycle by facilitating O–O bond cleavage, leading to the formation of the highly reactive manganese­(V)–oxo species (Scheme 2). Furthermore, the resulting carboxylate ligand may contribute to catalyst organization by occupying space around the metal center, thereby influencing the shape and steric properties of the substrate-binding pocket. As previously demonstrated, the structure of the acid additive can strongly influence both the yield and enantioselectivity of C–H oxidation reactions.^44–46^ Our group recently showed that it also has a major impact on site selectivity in solvophobicity-driven C–H oxidation of alkyl substrates.^32^

**Scheme 2:**
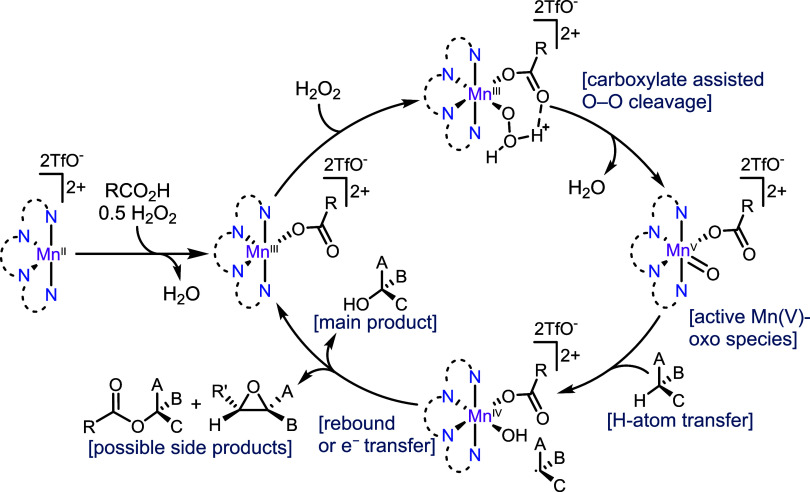
General Carboxylate-Assisted Catalytic Cycle for Mn-Catalyzed C­(sp^3^)–H Oxidation^47^

For optimal performance, its steric bulk must complement the cavity while still allowing substrate binding. By progressively increasing the volume of the carboxylic acid residue (entries 2, 4–7), the reaction selectivity improved markedly, with C4 selectivity rising from 42% (entry 2, acetic acid) to 86% (entry 7, 2,2-dimethylbutanoic acid). Moreover, the acid loading proved critical for reaction efficiency. Reducing the amount from 22 to 1 equiv (entries 7–9) enhanced the total yield nearly 2.5-fold, from 17 to 42%. In contrast, employing the even bulkier adamantane-1-carboxylic acid (entry 10) failed to improve either yield or C4 selectivity, suggesting that both the structure and the optimal loading of the carboxylic acid are key determinants of catalyst performance.

While 1 equiv of 2,2-dimethylbutanoic acid additive (entry 9) resulted in a high site selectivity of 90%, the conversion (75%) and total yield (42%) required further optimization. Doubling the amount of hydrogen peroxide (entry 11), increasing the catalyst loading (entry 12), or simultaneously increasing both (entry 13) did not improve conversion or yield. As an alternative approach, stepwise addition of catalyst (1 + 1 mol %) and oxidant (1 + 1 equiv) was explored (entries 14, 15) (see SI, section 3.2). This strategy afforded a clear increase in yield while preserving high C4 selectivity. The improvement was observed with both 2,2-dimethylbutanoic (entry 14) and 2,2-dimethylpropanoic (entry 15) acids, with the latter providing the most favorable overall performance (67% total yield, 89% C4 selectivity). The reduced mass balance observed for several entries likely arises from overoxidation at additional C–H sites and side products formation (see SI, section 3.3). A reduced mass balance in TFE has also been reported by other researchers.^48^ This behavior is consistent with its lower hydrogen-bond donating ability and reduced capacity to stabilize cationic or radical intermediates compared to HFIP, which can lead to less controlled oxidation and increased overoxidation pathways. Nevertheless, given its superior performance compared to HFIP in terms of site selectivity (entries 2–3), we selected TFE as the solvent for the present study.

Finally, to evaluate supramolecular catalyst **5**, control experiments were conducted with reference catalyst **2** under identical conditions: catalyst **2** alone (entry 16) and in combination with calix[4]­arene **6** (entry 17). Catalyst **2** delivered 31% C4 selectivity with 35% total yield, and the addition of calix[4]­arene **6** did not alter the selectivity. Similarly, catalyst **4**, bearing two calix[4]­arene units on the Mn­(mcp) core (Figure 1a), showed no improvement over catalyst **2** (entry 18). These results provide strong evidence that the high degree of preorganization of catalyst **5** is responsible for the observed high site selectivity.

Computational investigations were conducted to examine the role of the binding pocket in substrate positioning (see SI, section 5). Geometry optimizations and molecular dynamics simulations of the host–guest complex indicate that octane preferentially adopts conformations in which the C4 methylene group is located closest to the Mn=O oxidant. The resulting spatial bias is consistent with the experimentally observed site selectivity and suggests that substrate preorganization within the macrocyclic binding pocket is responsible for the observed selective C–H oxidation.

Since the oxidation of octane to a secondary alcohol generates chiral products and the catalysts utilized are derived from an enantiopure diamine backbone, we also examined the enantioselectivity of the reaction (see SI, section 3.3). To this end, oxidation of the initially formed alcohols with IBX, typically used to facilitate site selectivity analysis, was omitted, and the enantiomeric excess of 4-octanol was determined directly after workup. Notably, supramolecular catalyst **5** delivered an enantiomeric excess of 39% (entry 15), whereas catalyst **2** produced a racemic mixture (entry 16). This observation is notable, as enantioselective C–H functionalization of unfunctionalized alkanes remains an unsolved challenge.

With the optimized conditions established, we explored the reaction scope (Figure 2) and found efficient oxidation across a broad range of substrates. Alkanes of varying chain lengths (**S1**–**4**) were successfully oxidized, exhibiting high C4 selectivity of 64–76%. Notably, octane (**S2**) displayed exceptional selectivity of 89%, a consequence of its C2 symmetry, which renders the C4 and C5 positions equivalent. For other substrates, however, C5 remained a significant site of oxidation, accounting for approximately 20% of the product mixture (see SI, section 4 for more detailed oxidation results).

**Figure 2:**
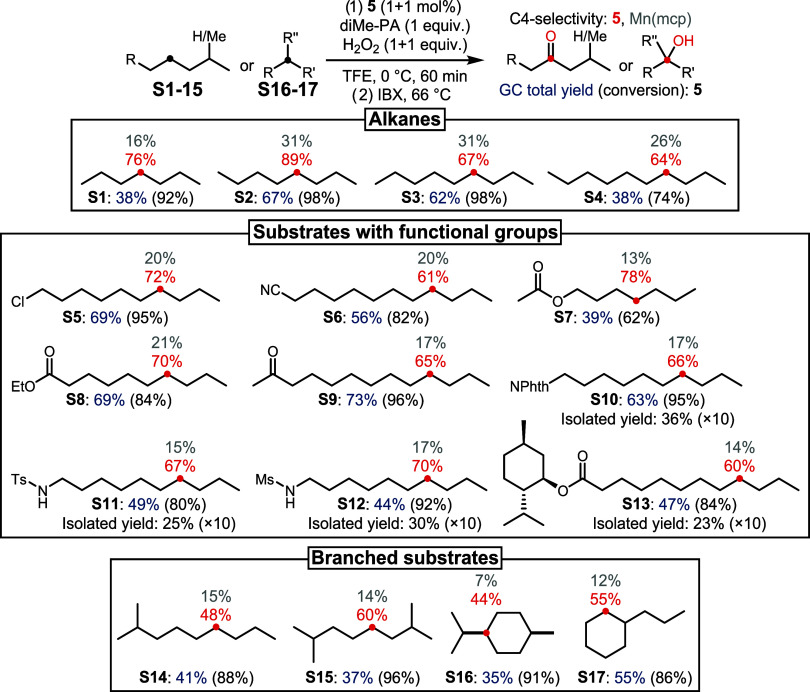
Substrate Scope and Comparison between Catalysts **5** and **2**. Regular scale (18.5 μmol); increased scale (×10, 185 μmol).

Next, functionalized alkyl substrates (**S5–S13**) with potential synthetic relevance were examined. C4 selectivity remained consistently high (60–78%), comparable to that observed with alkanes, highlighting the catalyst’s tolerance to a wide range of functional groups, including chloride (**S5**), nitrile (**S6**), esters (**S7–S8**, **S13**), ketone (**S9**), and protected amines (**S10–S12**). These results demonstrate the robustness and applicability of the methodology for late-stage C–H oxidation in more complex molecules. Furthermore, selected branched substrates (**S14–S17**) were examined to probe the catalyst’s capacity to accommodate bulkier guests within its cavity. The diminished C4 selectivity observed for **S14** suggests that the *i*-propyl terminus can also bind within the catalyst. This interpretation is supported by **S15**, which bears branching at both ends yet still affords 60% C4 selectivity. Notably, the catalyst exhibits selectivity for secondary C–H bonds in **S15**, overriding the inherent preference for more electron-rich tertiary centers observed with catalyst **2**.

Remarkably, even the cyclic substrate *cis*-*p*-menthane (**S16**) bound to the catalyst cavity, resulting in preferential oxidation at the sterically most hindered tertiary carbon (44%), a position that is hardly oxidized with catalyst **2** at all (7%). In the case of substrate **S17**, which contains a bulky cyclohexyl group, oxidation occurred preferentially adjacent to the tertiary center with 55% selectivity. For both **S16** and **S17**, the observed shift in oxidation site from the C4- to C5-position, (counting from the methyl group) can be rationalized by the greater conformational rigidity of the cyclohexyl unit compared to a more flexible alkyl chain. This rigidity likely positions the C5–H bond in closer proximity to the catalytic center, thereby favoring oxidation at this site.

The oxidation of the nonvolatile substrates **S10–13** was also performed on a larger scale (10-fold; 185 μmol) with no visible drop in catalyst performance, underscoring the robustness and scalability of the method. The corresponding C4 oxidation products were isolated from their regioisomers by HPLC in yields comparable to those determined by GC, underscoring the potential for practical synthetic applications.

To compare the catalytic performance of both catalysts, turnover numbers (TONs) and turnover frequencies (TOFs) were calculated for each substrate (see SI, section 4). For catalyst **5**, TON values ranged from 18 to 37 (TOFs are 20–41 × 10^–3^ s^–1^). These values are comparable to those obtained with Mn­(mcp), for which TONs ranged from 9 to 56 (TOFs are 9.9–63 × 10^–3^ s^–1^).

To further validate the broad utility of this approach, we performed several larger-scale (10-fold; 185 μmol) and a preparative-scale (30-fold; 555 μmol) reactions to demonstrate its applicability to the derivatization of complex molecules (Figure 3a).

**Figure 3:**
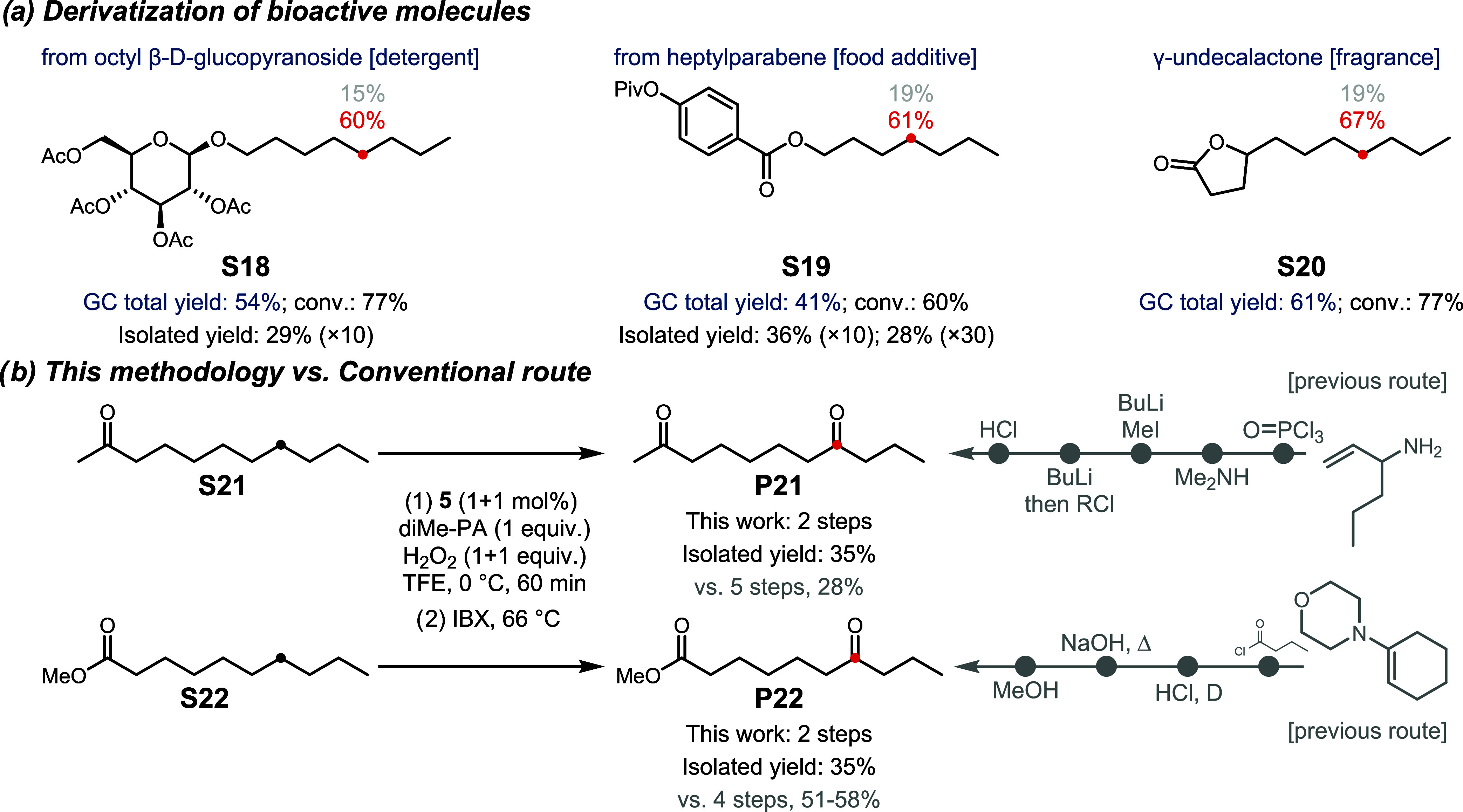
Applications of catalyst **5**: enabling the derivatization of natural products and bioactive molecules (a); shortening synthetic routes of known compounds (b). Regular scale (18.5 μmol); increased scales (×10, 185 μmol or ×30, 555 μmol).

The oxidations of a widely used nonionic surfactant^49,50^ and natural product^51–53^ **S18**, and the bioactive molecule **S19**,^54^ were carried out on their acylated derivatives to protect the hydroxyl groups. The desired products were isolated by HPLC in yields comparable to those determined by GC analysis and were fully characterized. In addition, γ-undecalactone (**S20**), an aroma compound and flavoring agent, was successfully oxidized with 67% C4 selectivity and 61% overall yield. Overall, these results demonstrate the utility of the method for site-selective functionalization across structurally complex and industrially relevant substrates.

To demonstrate the strategic impact of catalyst **5** in shortening synthetic routes, we next explored its application to the synthesis of known compounds (Figure 3b). Previously reported synthetic routes to compounds **P21** (5 steps)^55^ and **P22** (4 steps)^56–58^ were shortened using catalyst **5** and commercially available substrates **S21** and **S22**. This approach enabled more concise synthetic sequences, illustrating the potential of catalyst **5** to reduce step count and enhance synthetic accessibility to propyl carbonyl fragments.

## Conclusion

In conclusion, we have introduced a macrocyclic supramolecular manganese catalyst that achieves unprecedented site-selective oxidation of alkanes, independent of functional groups. By exploiting the solvophobic effect to drive substrate binding, this approach provides a general strategy for selective C–H functionalization of unactivated hydrocarbons at the C4-position. The only previously reported related supramolecular catalyst, **3**, was limited to 40–60% C5 selectivity and a very narrow substrate scope, owing to its requirement to engage both ends of the substrate. In contrast, the macrocyclic design reported here consistently delivers robust C4 oxidation with selectivities of 60–70%, reaching up to 89% in one case, highlighting the decisive role of preorganization in achieving high selectivity. Its application in the larger-scale selective oxidations of complex molecules, including substrates relevant to late-stage functionalization, highlights its broad applicability. Furthermore, this macrocyclic platform establishes a blueprint for the design of next-generation supramolecular oxidation catalysts with tunable site selectivity, offering transformative potential for late-stage functionalization and the broader field of selective C–H bond functionalization.

## Supplementary Material



## Notes

Experimental details, computational details, and NMR spectra of compounds (PDF)

## Abbreviations

AcOH: acetic acid
Ad-CO_2_H: adamantane-1-carboxylic acid
diMe-BA: 2,2-dimethylbutanoic acid
diMe-PA: 2,2-dimethylpropanoic acid
HFIP: 1,1,1,3,3,3-hexafluoro-2-propanol
IBX: 2-iodoxybenzoic acid
Me-PA: 2-methylpropanoic acid
PA: propanoic acid
TFE: 2,2,2-trifluoroethanol
