# Supplementary material for: Solvophobic Effect-Driven C4-Selective C–H Oxidation of Diverse Alkyl Substrates by a Macrocyclic Supramolecular Catalyst
Source: J Am Chem Soc. 2026 Jul 31;148(31):33508–15. doi: 10.1021/jacs.6c08837 (PMC13474948; doi:10.1021/jacs.6c08837)

Solvophobic Effect-Driven C4-Selective C-H Oxidation of Diverse Alkyl Substrates by a Macrocyclic Supramolecular Catalyst

Kanstantsin Anisovich[1], Yiheng Lu[1], Bernd Goldfuss[2] and Konrad Tiefenbacher[1,3]*

[1] Department of Chemistry, University of Basel, Mattenstrasse 22, 4058 Basel, Switzerland

[2] Institute of Organic Chemistry, University of Cologne, Greinstrasse 4, 50939 Cologne, Germany

[3] Department of Biosystems Science and Engineering, ETH Zurich, Klingelbergstrasse 48, 4056 Basel, Switzerland

Table of Contents

1. General information 3

2. Synthesis of catalysts and substrates 6

2.1 Synthesis of ***Mn(S,S-mcp)*** *and* ***Mn(S,S-mcp)-CX$_2$*** *6*

2.2 Synthesis of calix[4]arene-based supramolecular catalyst ***5*** *6*

2.3 Synthesis of substrates *14*

3. Oxidation studies 21

3.1 Catalysts's dependance on TfOH concentration *21*

3.2 Reaction condition optimization with model substrate octane *22*

3.3 Octane oxidation products distribution *26*

3.4 Catalyst's activity and stability under oxidation conditions *28*

4. Substrate Scope and Detailed Oxidation Results 34

4.1 General procedure for oxidation reaction. *34*

*Heptane (**S1**) oxidation* *35*

*Octane (**S2**) oxidation* *36*

*Nonane (**S3**) oxidation* *37*

*Decane (**S4**) oxidation* *38*

*1-Chlorodecane (**S5**) oxidation* *39*

*Dodecanenitrile (**S6**) oxidation* *40*

*Heptyl acetate (**S7**) oxidation* *44*

*Ethyl nonanoate (**S8**) oxidation* *48*

*Tridecan-2-one (**S9**) oxidation* *53*

*2-Decylisoindoline-1,3-dione (**S10**) oxidation* *58*

*N-Decyl-4-methylbenzenesulfonamide (**S11**) oxidation* *60*

*N-Decylmethanesulfonamide (**S12**) oxidation* *62*

*(-)-Menthyl laurate (**S13**) oxidation* *64*

*2-Methylnonane (**S14**) oxidation* *66*

*2,7-Dimethyloctane (**S15**) oxidation* *67*

*cis-1-Isopropyl-4-methylcyclohexane (**S16**) oxidation* *68*

*Propylcyclohexane (**S17**) oxidation* *69*
*Octyl (2,3,4,6-tetra-O-acetyl)-β-D-glucopyranoside (**S18**) oxidation* *74*
*Heptyl 4-(pivaloyloxy)benzoate (**S19**) oxidation* *76*
*5-Heptyldihydrofuran-2(3H)-one (**S20**) oxidation* *79*
*2-Undecanone (**S21**) oxidation* *83*
*Methyl decanoate (**S22**) oxidation* *85*
5. Computational part 87
6. References 89
Appendix A: NMR spectra of compounds 91

1. General information

Experimental:

Reactions were performed under air in oven-dried glassware unless otherwise indicated. For the rhodiun(I)-catalyzed borylation Synthware™ cylindrical pressure vessel with protective coating was used. Catalyst synthesis was done in a glovebox (N_2 atmosphere, $O_2 \leq 0.1$ ppm).

For work-up of scaled up oxidation reactions Robu VitraPOR® glass filters (porosity 3 or 4) were used.

Slow additions were carried out on a KD Scientific Legato 100 Infuse Only Syringe Pump.

Analytical thin-layer chromatography (TLC) was performed on Merck silica gel 60 F_{254} (SiO_2) or Merck aluminium oxide 60 F_{254}, neutral (Al_2O_3) aluminium-backed plates and analyzed under UV light (λ = 254 nm [UV]).

All NMR experiments were performed on a Bruker Avance Neo and a Bruker Avance III HD NMR spectrometer operating at 500 MHz and 600 MHz proton frequency, respectively. The instruments were equipped with a direct observe 5-mm BBFO smart probe (500 MHz) or a five-channel cryogenic 5 mm QCI probe (600 MHz). All probes were equipped with actively shielded z-gradients (10 A). The measurements were performed at 298 K. Chemical shifts of ^{1}H NMR and ^{13}C NMR are given in ppm. The following solvent residual signals of the deuterated solvents were used as reference: $CDCl_3$: 7.26 ppm (δ^1H), 77.00 ppm (δ^{13}C). Coupling constants (*J*) are reported in Hertz (Hz). Standard abbreviations indicating multiplicity were used as follows: s (singlet), br s (broad singlet), d (doublet), t (triplet), br t (broad triplet), dd (doublet of doublets), dt (doublet of triptets), td (triplet of doublets), ddd (doublet of doublets of doublets), m (multiplet).

Medium Pressure Liquid Chromatography (MPLC) was carried out with RediSep® Silica Gel Disposable Flash Columns SiO_2 columns (particle size 40-63 µm) and Al_2O_3 basic columns (particle size 40-63 µm) on a CombiFlash NextGen 300+ version 5.0.55 by Teledyne ISCO with a fraction collector version 00.92.00, detector version 11 and a pump version: 1.47. For all the runs the column type and size, flow rate [ml/min], solvent mixture, column volumes (CV) and run time [min] are given.

HPLC was performed on an LC Prominence Liquid Chromatograph system by Shimadzu equipped with a UV-Vis detector and an ELSD unit. For analytical HPLC, a Reprospher 100 Si (particle size: 5 µm; dimensions: 250 × 4.6 mm) Dr. Maisch column was used. For preparative separation, a Reprospher 100 Si (particle size: 5 µm; dimensions: 250 × 25 mm) Dr. Maisch column was used.

Infrared spectra were measured on a Brucker Alpha IR spectrometer (ATR, attenuated total reflection). Abbreviations indicating intensity were used as follows: s (strong), m (medium), w (weak).

Melting points were recorded on a Büchi Melting Point M-565 apparatus using open capillary tubes.

GC analyses were carried out on a Shimadzu GC-2010 Plus instrument equipped with a flame ionization detector (FID) and column:

- achiral Rtx-5 capillary (length = 30 m). Hydrogen was used as the carrier gas and the linear velocity mode was used (pressure = 106.9 kPa, total flow = 64.1 mL/min, linear velocity = 69.8 cm/s) with a split ratio of 1:20;
- chiral MEGA-DEX DAC β capillary (length = 60 m). Hydrogen was used as the carrier gas and the linear velocity mode was used (pressure = 177.6 kPa, total flow = 68.7 mL/min, linear velocity = 57.4 cm/s) with a split ratio of 1:20

Temperature programs are given for every entry. The response factors of substrates and oxidation products were calculated according to the literature.[1,2]

GC-MS analyses were performed on Agilent 7890B gas chromatograph equipped with electron capture detector (G3442B), combined with mass spectrometer 5977B (ionization: electron impact (EI), detector G7081B). The achiral GC capillary column Agilent J&W HP-5MS UI (length = 30 m) was equipped and helium was used as the carrier gas (split ratio = 50:1, constant flow = 3.25 mL/min, pressure = 26.6 psi, average velocity = 66 cm/s). Same temperature program as on the Shimadzu GC-2010 Plus with achiral column was employed to achieve similar retention times on both columns, unless else specified.

High-resolution mass spectra were obtained on a Bruker maxis 4G (ESIQ-TOF) (ESI source parameters for positive polarity mode were: Set capillary: 4.5 kV, Set Ion Energy 4.0 eV).

Recycling gel permeation chromatography (GPC) purification were performed on a LaboACE LC-5060 Plus II from Japan Analytical Industry Co. Ltd equipped with a JAIGEL-2HR Plus and JAIGEL-2.5HR Plus column in series. Chloroform stabilized with 0.75% ethanol was used as eluent.

Transfer of liquids with a volume ranging from 1 to 10 µL or from 10 to 100 µL was performed with a Microman M1 pipette (Gilson) equipped with 10 µL or 100 µL pipette tips, respectively. 4 and 12 mL screw neck vials were purchased from Fisher Scientific.

Sources of solvents:

Anhydrous acetonitrile (MeCN), dichloromethane (CH_2Cl_2), dimethylformamide (DMF), 1,4-dioxane, hexane, methanol (MeOH), pyridine (Py), tetrahydrofuran (THF) and toluene were purchased from Acros Organics.

Solvents for extractions, chromatography, filtrations, and non-anhydrous reactions were purchased from VWR as HPLC grade solvents and were used without prior purification.

1,4-Dioxane and hexane used for the catalyst synthesis were additionally degassed by freeze-pump-thaw method.

Fluorinated alcohols 2,2,2-trifluoroethanol (TFE) and 1,1,1,3,3,3-hexafluoroisopropanol (HFIP) were bought from Fluorochem.

Deuterated chloroform (stabilized over silver foil) ($CDCl_3$, 99.8%) was purchased from Cambridge Isotope Laboratories.

Sources of chemicals:

Chemical	Cat. No.	Supplier	CAS; comment
**1-Bromo-4-(2-bromoethyl)benzene**	FLUF304695-5g	Fluorochem	1746-28-7
**5-Hydroxypicolinaldehyde**	FLUF214858-5g	Fluorochem	31191-08-9
**Potassium carbonate anhydrous**	ACR44762-0010	Acros	584-08-7; oven dried
**4-*tert*-Butylcalix[4]arene**	ACR29022-0250	Acros	60705-62-6
**Phenol**	ACR18078-1000	Acros	108-95-2
**Aluminium chloride anhydrous**	ACR19578-5000	Acros	7446-70-0
**Sodium hydride (60% in Paraffin oil)**	452912-100G	Sigma-Aldrich	7646-69-7
**1-Bromopropane**	ACR10731-2500	Thermo Scientific	106-94-5
**Bis(pinacolato)diboron**	APOSOR5456-100G	Apollo	73183-34-3
**4,4'-Di-*tert*-butyl-2,2'-bipyridyl**	D3134	TCI	72914-19-3
**(1,5-Cyclooctadiene)(methoxy)iridium(I) dimer**	685062-250MG	Sigma-Aldrich	12148-71-9
**[1,1"-Bis(diphenylphosphino)ferrocene]dichloro-palladium(II)**	APOOR8775-1g	Apollo	72287-26-4
**Sodium borohydride**	452882-100G	Sigma-Aldrich	16940-66-2
**Phosphorus tribromide**	157783-100G	Sigma Aldrich	7789-60-8

**(1*S*,2*S*)-*N*,*N*′-Dimethyl-1,2-cyclohexanediamine**	FLUF034487-1g	Fluorochem	87583-89-9
**Tetrabutylammonium bromide**	193119	Sigma-Aldrich	1643-19-2
**Manganese bis(trifluoromethanesulphonate)**	APOPC49662-1g	Apollo	55120-76-8
**Decylamine**	D2404-25G	Sigma-Aldrich	2016-57-1
**Phthalic anhydride**	ACR15496-2500	Acros	85-44-9
**Methanesulfonyl chloride**	64270	Sigma-Aldrich	124-63-0
**Triethylamine**	ACR15791-0010	Acros	121-44-8
***p*-Toluenesulfonyl chloride**	AB119196	ABCR	98-59-9
**(−)-Menthol**	15785-100G	Sigma-Aldrich	2216-51-5
**Lauroyl chloride**	156930-50ML	Sigma-Aldrich	112-16-3
**Methanesulfonic acid**	APOOR322096-100g	Apollo	75-75-2
**(*cis*-4-Isopropylcyclohexyl)methanol**	BL3H98821E22-5G	Sigma-Aldrich	13828-37-0; 70% *cis*-isomer
**Lithium triethylborohydride in THF (1.0 M)**	179728-4X25ML	Sigma-Aldrich	22560-16-3
**Octyl-β-D-glucopyranoside**	CN23.1	CARL ROTH	29836-26-8
**Acetyl chloride**	00990-100ML	Sigma-Aldrich	75-36-5
**Heptyl 4-hydroxybenzoate**	CBLQF-5017-25g	Combi-Blocks	1085-12-7
**Trimethylacetyl chloride**	T72605-100ML	Sigma-Aldrich	3282-30-2
**Octane**	8069100025	Sigma-Aldrich	111-65-9
**Nonane**	ACR12911-1000	Thermoscientific - Acros	111-84-2
**Decane**	3474	CARL ROTH	124-18-5
**1-Chlorodecane**	TCIC0600-25ML	TCI	1002-69-3
**Dodecanenitrile**	L0110	TCI	2437-25-4
**Heptyl Acetate**	TCIA0904-25ML	TCI	112-06-1
**Ethyl caprate**	ACR16500-0050	Acros	110-38-3
**2-Tridecanone**	T0415	TCI	593-08-8
**2-Methylnonane**	ALFB20849-03	Thermo Scientific - Alfa Aesar	871-83-0
**2,7-Dimethyloctane**	ALFB21646-03	Thermo Scientific - Alfa Aesar	1072-16-8
**Propylcyclohexane**	APOOR1029583-5g	Apollo	1678-92-8
**Undecan-4-olide**	APOOR1015404-25g	Apollo	104-67-6
**Methyl caprate**	ACR38555-0250	Acros	110-42-9
**2-Undecanone**	ACR14069-0050	Acros	112-12-9
**Acetic acid**	33209-1L	Honeywell - Fluka	64-19-7
**Propionic acid**	6026	CARL ROTH	79-09-4
**Isobutyric acid**	ALFL04038-AE	Thermo Scientific - Alfa Aesar	79-31-2
**Trimethylacetic acid**	ACR14002-1000	Thermo Scientific - Acros	75-98-9
**2,2-Dimethylbutyric acid**	D2429	TCI	595-37-9
**1-Adamantanecarboxylic acid**	106399-25G	Sigma-Aldrich	828-51-3
**Hydrogen peroxide solution 50 wt. % in H_2O, stabilized**	516813-500ML	Sigma-Aldrich	7722-84-1
**Biphenyl**	B34656-25G	Sigma-Aldrich	92-52-4
**Trifluoromethanesulfonic acid**	FLUF003328-25g	Fluorochem	1493-13-6
**2-Iodoxybenzoic acid**	APOOR3108-100g	Apollo	61717-82-6

Silica gel (0.040-0.063 mm, 230-400 mesh ASTM) and Celite® 545 (particle size 0.02-0.1 mm) were purchased from Merck KGaA.

2. Synthesis of catalysts and substrates

C(sp[3])-H oxidation catalysts investigated in this work:

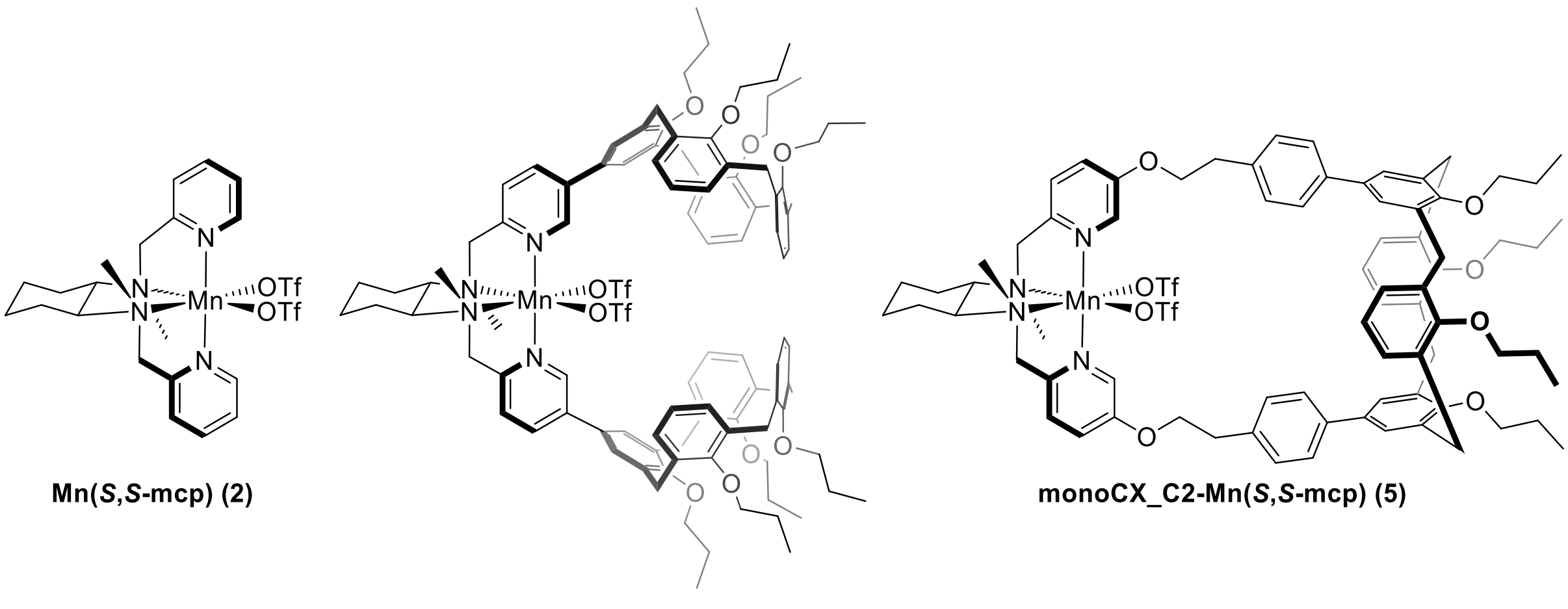

Mn(*S*,*S*-mcp) (2)

monoCX_C2-Mn(*S*,*S*-mcp) (5)

Mn(*S*,*S*-mcp)-CX_2 (4)

2.1 Synthesis of Mn(*S*,*S*-mcp) and Mn(*S*,*S*-mcp)-CX_2

Synthesis of **Mn(*S*,*S*-mcp)** and **Mn(*S*,*S*-mcp)-CX_2** were carried out as previously described.[3,4]

2.2 Synthesis of calix[4]arene-based supramolecular catalyst 5

1,3,5,7(1,3)-Tetrabenzenacyclooctaphane-1^2,3^2,5^2,7^2-tetraol (SI-1)

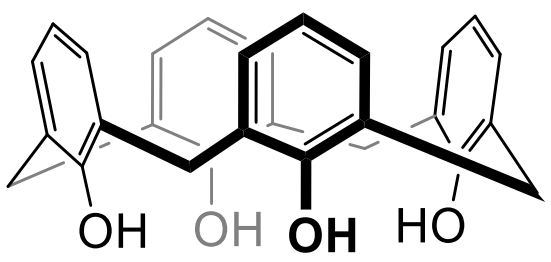

Chemical Formula: $C_{28}H_{24}O_4$
Molecular Weight: 424.50

An oven-dried 500 mL round-bottom one neck flask with a magnetic stirrer was charged with $AlCl_3$ (24.6 g, 185 mmol) and anhydrous toluene (120 mL) under an inert atmosphere. 4-*tert*-Butylcalixarene (15.0 g, 23.1 mmol) and phenol (21.7 g, 231 mmol) were added and the mixture was stirred overnight at room temperature. The reaction mixture was cooled to 0 °C and aqueous HCl solution (0.2 M, 190 mL) was added under vigorous stirring. The mixture was stirred for 30 min and left to stand overnight. The organic layer was separated and the aqueous layer was extracted with DCM (3 × 60 mL). The combined organic layers were washed with water (3 × 50 mL), dried over sodium sulfate, filtered and solvents were removed *in vacuo*. Methanol (190 mL) was added to the resulting oil and the precipitate formed was filtered off, washed with methanol (2 × 10 mL), and dried. The crude product **SI-1** (pale yellow solid) was used in the following steps with no further purification.

Yield: 8.20 g, 84%.

^{1}H NMR (500 MHz, $CDCl_3$, 298K) δ 10.20 (s, 4H, 4×OH), 7.06 (d, ^3J = 7.6 Hz, 8H, 8×CH, Ar), 6.73 (t, ^3J = 7.6 Hz, 4H, 4×CH, Ar), 4.27 (br s, 4H, 4×$CH_{2\,(bridging)}$), 3.55 (br s, 4H, 4×$CH_{2\,(bridging)}$).

^{13}C NMR (125 MHz, $CDCl_3$, 298K) δ 148.8 (4×C, Ar), 129.0 (8×CH, Ar), 128.2 (8×C, Ar), 122.2 (4×CH, Ar), 31.7 (4×$CH_{2\,(bridging)}$).

^{1}H and ^{13}C NMR-shifts match the reported values.[5]

$1^2,3^2,5^2,7^2$-Tetrapropoxy-1,3,5,7(1,3)-tetrabenzenacyclooctaphane (6)

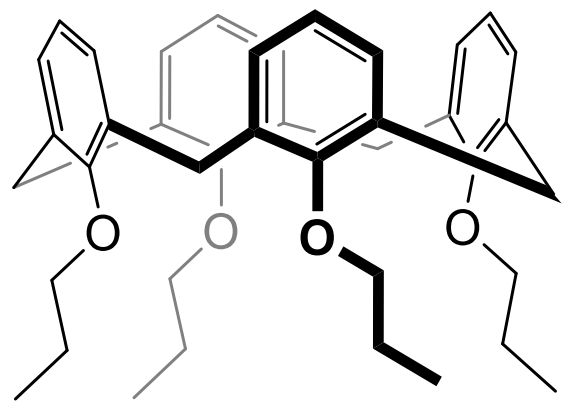

Chemical Formula: $C_{40}H_{48}O_4$
Molecular Weight: 592.82

An oven-dried 500 mL round-bottom one neck flask with a magnetic stirrer was charged with sodium hydride (60% in oil dispersion; 7.55 g, 189 mmol) and DMF (140 mL). Calix[4]arene **SI-1** (5.00 g, 11.8 mmol) was added in 4 portions and the reaction mixture was heated for 30 min at 50 °C. After cooling back to room temperature 1-bromopropane (17.2 mL, 23.2 g, 189 mmol) was added via syringe and the reaction mixture was heated at 50 °C for 22 hours. After cooling to room temperature ice-water mixture (150 mL) was added and the aqueous layer was extracted with DCM (3 ×100 mL). The combined organic layers were washed with water (3 ×100 mL), 1 M aqueous NH_4Cl solution (100 mL), brine (100 mL) and dried over Na_2SO_4. DCM was removed *in vacuo* to give a yellow oil, MeOH (20 mL) was added and the mixture was left to stand overnight. The solids were collected by filtration, washed with methanol (3×20 mL) and dried *in vacuo* to give crude product. The purification was done by flash column chromatography (A: cyclohexane, B: dichloromethane, gradient: 0-15% of B, 80 g RediSep® silica column, run length = 15 CV, eluted at 8% of B, 8-14 CV) to afford the terapropylated calixarene **6** as a white solid.

Yield: 4.80 g, 69%.

^{1}H NMR (500 MHz, $CDCl_3$, 298K) δ 6.69 – 6.50 (m, 12H, 8×CH+4×CH, Ar), 4.46 (d, ^2J = 13.4 Hz, 4H, 4×$CH_{2\,(bridging)}$), 3.85 (t, ^3J = 7.5 Hz, 8H, 4×CH_2), 3.15 (d, ^2J = 13.4 Hz, 4H, 4×$CH_{2\,(bridging)}$), 1.99 – 1.86 (m, 8H, 4×CH_2), 1.00 (t, ^3J = 7.5 Hz, 12H, 4×Me).

^{13}C NMR (125 MHz, $CDCl_3$, 298K) δ 156.6 (4×C, Ar), 135.1 (8×C, Ar), 128.1 (8×CH, Ar), 121.8 (4×CH, Ar), 76.7 (4×CH_2), 31.0 (4×$CH_{2\,(bridging)}$), 23.2 (4×CH_2), 10.3 (4×Me).

^{1}H and ^{13}C NMR-shifts match the reported values.[6]

2,2'-($1^2,3^2,5^2,7^2$-Tetrapropoxy-1,3,5,7(1,3)-tetrabenzenacyclooctaphane-$1^5,5^5$-diyl)bis(4,4,5,5-tetramethyl-1,3,2-dioxaborolane) (7)

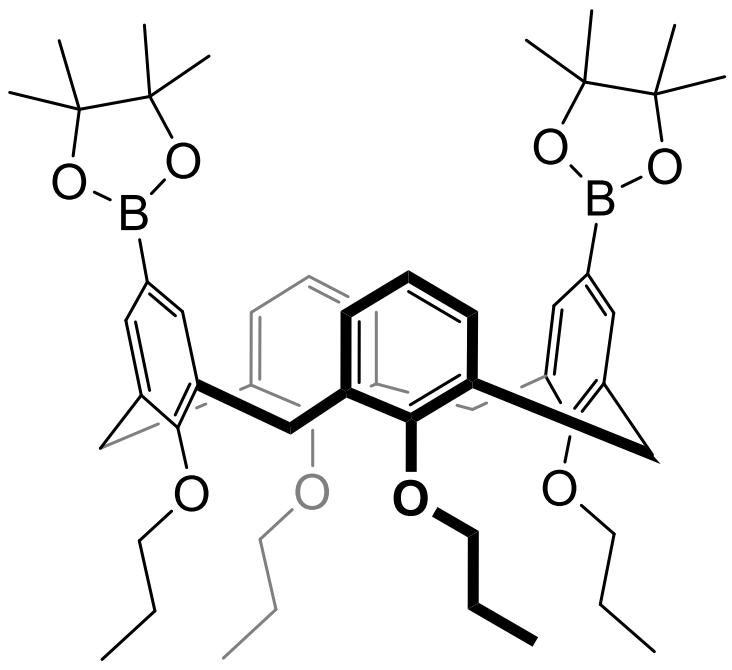

Chemical Formula: $C_{52}H_{70}B_2O_8$
Molecular Weight: 844.74

An oven-dried 38 mL pressure tube was charged with 4,4′-di-*tert*-butyl-2,2′-dipyridyl (14.5 mg, 0.054 mmol), bis(pinacolato)diboron (342 mg, 1.35 mmol), $[Ir(COD)OMe]_2$ (17.9 mg, 0.027 mmol) and

degassed THF (1.00 mL) under an inert atmosphere. The reaction mixture was stirred for 10 min until a dark red colour appeared. Calix[4]arene **6** (400 mg, 0.675 mmol) was added. The reaction mixture was bubbled through with argon for a minute and then heated at 78 °C for 2 days. The mixture was allowed to cool to room temperature, filtered through Celite®, washed with DCM and solvents were removed *in vacuo*. The crude residue was purified by flash column chromatography (A: cyclohexane, B: dichloromethane, gradient: 0-90% of B, 120 g RediSep® silica column, run length = 32 CV, eluted at 60% of B, 21-30 CV) to afford the borylated calix[4]arene **7** as a light-tan powder.

Yield: 300 mg, 53%.

Mp.: 292 – 293 °C (decomposition).

$\mathbf{R}_f$ 0.52 (SiO_2, cyclohexane / ethyl acetate, 8:1).

IR (ATR, ν/cm^{-1}) 2975 (w), 2927 (w), 2875 (w), 1596 (w), 1453 (w), 1362 (s), 1316 (w), 1202 (m), 1142 (s), 1003 (m), 968 (m), 886 (w), 855 (m), 756 (m), 695 (w), 686 (w).

^{1}H NMR (500 MHz, $CDCl_3$, 298K) δ 7.60 (s, 4H, 4×CH, Ar), 6.21 – 6.05 (m, 6H, 4×CH+2×CH, Ar), 4.42 (d, ^2J = 13.4 Hz, 4H, 4×$CH_{2\,(bridging)}$), 4.13 – 4.00 (m, 4H, 2×CH_2), 3.66 (t, ^3J = 6.7 Hz, 4H, 2×CH_2), 3.19 (d, ^2J = 13.4 Hz, 4H, 4×$CH_{2\,(bridging)}$), 1.97 – 1.83 (m, 8H, 4×CH_2), 1.39 (s, 24H, 8×Me), 1.10 (t, ^3J = 7.4 Hz, 6H, 2×Me), 0.85 (t, ^3J = 7.5 Hz, 6H, 2×Me).

^{13}C NMR (125 MHz, $CDCl_3$, 298K) δ 161.2 (2×C, Ar), 155.0 (2×C, Ar), 136.6 (4×C, Ar), 135.7 (4×CH, Ar), 133.0 (4×C, Ar), 127.5 (4×CH, Ar), 122.0 (2×CH, Ar), 83.5 (4×C), 76.9 (2×CH_2), 76.4 (2×CH_2), 30.8 (4×$CH_{2\,(bridging)}$), 25.0 (8×Me), 23.5 (2×CH_2), 23.0 (2×CH_2), 10.8 (2×Me), 9.8 (2×Me).

NOTE: In the ^{13}C NMR, quaternary carbons next to the boron are not observed.

HRMS (ESI$^+$) *m/z* calcd for $C_{52}H_{70}B_2O_8Na$ [M + Na]$^+$: 867.5166; found 867.5168.

^{1}H and ^{13}C NMR-shifts match the reported values.[7]

5-(4-Bromophenethoxy)picolinaldehyde (10)

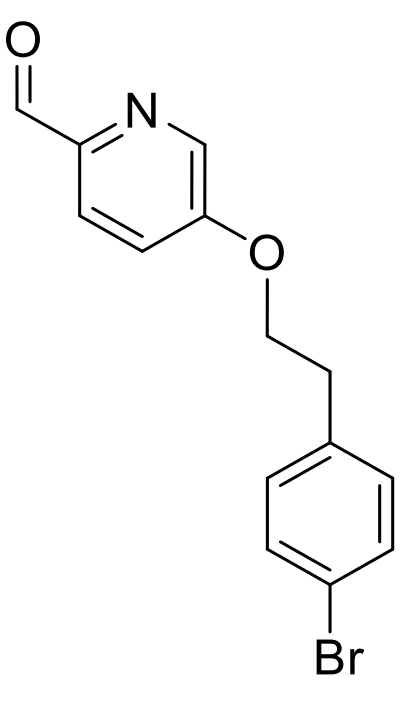

Chemical Formula: $C_{14}H_{12}BrNO_2$
Molecular Weight: 306.16

In an oven-dried 12 mL vial with a magnetic stirrer a mixture of 5-hydroxypicolinaldehyde **8** (492 mg, 4.00 mmol) and potassium carbonate (1.11 g, 8.00 mmol) in *N*,*N*-dimethylformamide (5.00 mL) was heated in an oil bath at 60°C for 30 minutes. The mixture was cooled down to room temperature, 1-bromo-4-(2-bromoethyl)benzene **9** (611 µL, 1.06 g, 4.00 mmol) was added and the reaction mixture was stirred at 66°C for 3 hours and then cooled down to room temperature. The solids were filtered off and the solvent removed *in vacuo*. Water (15 mL) and ethyl acetate (10 mL) were added to the crude, the aqueous layer was extracted with ethyl acetate (3 × 10 mL). The combined organic layers were washed with water (3 × 10 mL), brine (10 mL), dried over sodium sulfate and the solvent removed *in*

vacuo; the crude product was then purified via flash column chromatography (A: cyclohexane, B: ethyl acetate, gradient: 0-80% of B, 120 g RediSep® silica column, run length = 11 CV, eluted at 40% of B, 6.5-8.0 CV) to give **10** as a white solid.

Yield: 406 mg, 33%.

Mp.: 84 – 85 °C.

R_f 0.43 (SiO_2, cyclohexane / ethyl acetate, 2:1).

IR (ATR, ν/cm^{-1}) 2952 (w), 2932 (w), 2880 (w), 2817 (w), 1696 (s), 1570 (s), 1477 (w), 1466 (w), 1313 (m), 1257 (s), 1209 (s), 1196 (s), 1129 (m), 1072 (w), 1021 (m), 1008 (s), 836 (m), 810 (m), 780 (m), 664 (w), 598 (s), 540 (s), 494 (w).

^{1}H NMR (500 MHz, $CDCl_3$, 298K) δ 9.98 (d, ^5J = 0.8 Hz, 1H, CHO), 8.41 (dd, ^4J = 2.7, ^5J = 0.6 Hz, 1H, CH, Ar), 7.94 (dd, ^3J = 8.6, ^5J = 0.6 Hz, 1H, CH, Ar), 7.46 (d, ^3J = 8.4 Hz, 2H, 2×CH, Ar), 7.26 (ddd, ^3J = 8.6, ^4J = 2.7, ^5J = 0.8 Hz, 1H, CH, Ar), 7.17 (d, ^3J = 8.4 Hz, 2H, 2×CH, Ar), 4.29 (t, ^3J = 6.7 Hz, 2H, CH_2), 3.11 (t, ^3J = 6.7 Hz, 2H, CH_2).

^{13}C NMR (150 MHz, $CDCl_3$, 298K) δ 192.0 (CHO), 158.1 (C, Ar), 146.4 (C, Ar), 138.7 (CH, Ar), 136.3 (C, Ar), 131.7 (2×CH, Ar), 130.7 (2×CH, Ar), 123.3 (CH, Ar), 120.7 (C, Ar), 120.5 (CH, Ar), 69.0 (CH_2), 34.9 (CH_2).

HRMS (ESI$^+$) *m/z* calcd for $C_{14}H_{12}BrNO_2Na$ [M + Na]$^+$: 327.9944; found 327.9947.

5,5'-((((1^2,3^2,5^2,7^2-tetrapropoxy-1,3,5,7(1,3)-tetrabenzenacyclooctaphane-1^5,5^5-diyl)bis(4,1-phenylene))bis(ethane-2,1-diyl))bis(oxy))dipicolinaldehyde (SI-2)

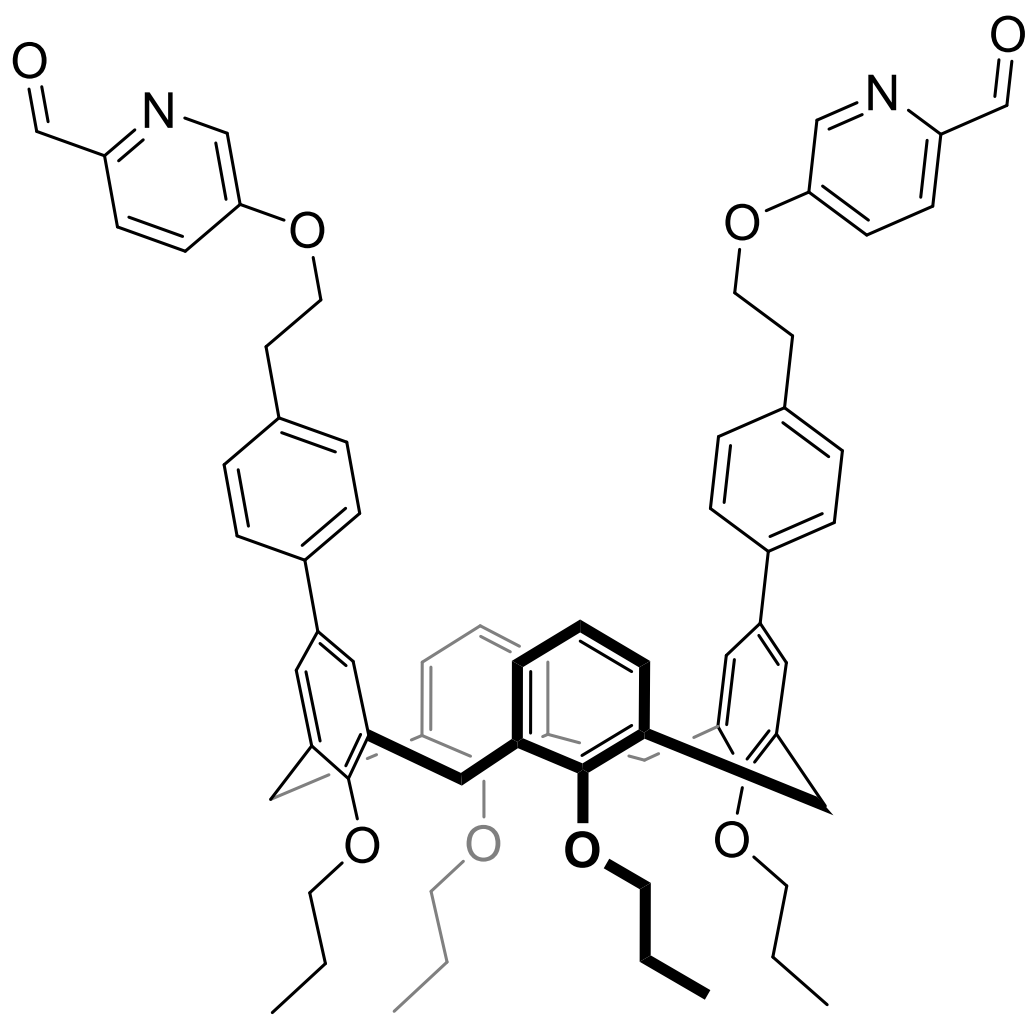

Chemical Formula: $C_{68}H_{70}N_2O_8$
Molecular Weight: 1043.31

An oven-dried 100 mL Schlenk flask was charged with diborylated calix[4]arene **7** (300 mg, 0.355 mmol), linker precursor **10** (435 mg, 1.42 mmol), Pd(dppf)Cl_2 (39.0 mg, 0.053 mmol) and degassed 1,4-dioxane (46.0 mL) under an inert atmosphere. Simultaneously in a separate flask 2 M K_2CO_3 aqueous solution was sparged with argon for 45 minutes and 9.40 mL of this solution was added to the reaction mixture. The biphasic mixture was heated at 80 ˚C for 24 h under vigorous stirring. The reaction was then cooled to room temperature and washed with brine (2 x 40 mL). The organic layer was collected, dried with Na_2SO_4, filtered and concentrated *in vacuo*. Purification by flash column

chromatography (A: cyclohexane, B: ethyl acetate, gradient: 0-50% of B, 80 g RediSep® silica column, run length = 27 CV, eluted at 30% of B, 17-23 CV) gave the product **SI-2** as a white solid.

Yield: 194 mg, 52%.

Mp.: 278 – 279 °C (decomposition).

R*f* 0.32 (SiO_2, cyclohexane / ethyl acetate, 2:1).

IR (ATR, ν/cm^{-1}) 2958 (w), 2931 (w), 2873 (w), 1706 (m), 1581 (m), 1572 (m), 1463 (m), 1315 (w), 1274 (s), 1267 (s), 1197 (s), 1086 (w), 1038 (w), 1006 (s), 827 (m), 761 (m), 610 (m).

^{1}H NMR (500 MHz, $CDCl_3$, 298K) δ 9.99 (d, ^5J = 0.7 Hz, 2H, CHO), 8.44 (dd, ^4J = 2.8, ^5J = 0.5 Hz, 2H, 2×CH, Ar), 7.95 (dd, ^3J = 8.6, ^5J = 0.5 Hz, 2H, 2×CH, Ar), 7.45 (d, ^3J = 8.2 Hz, 4H, 4×CH, Ar), 7.29 (ddd, ^3J = 8.6, ^4J = 2.8, ^5J = 0.7 Hz, 2H, 2×CH, Ar), 7.25 (d, ^3J = 8.2 Hz, 4H, 4×CH, Ar), 7.16 (s, 4H, 4×CH, Ar), 6.35 (s, 6H, 6×CH, Ar), 4.51 (d, ^2J = 13.4 Hz, 4H, 4×$CH_{2\,(bridging)}$), 4.32 (t, ^3J = 7.0 Hz, 4H, 2×CH_2), 4.04 – 3.95 (m, 4H, 2×CH_2), 3.78 (t, ^3J = 7.0 Hz, 4H, 2×CH_2), 3.21 (d, ^2J = 13.4 Hz, 4H, 4×$CH_{2\,(bridging)}$), 3.16 (t, ^3J = 7.0 Hz, 4H, 2×CH_2), 2.03 – 1.89 (m, 8H, 4×CH_2), 1.08 (t, ^3J = 7.5 Hz, 6H, 2×Me), 0.95 (t, ^3J = 7.5 Hz, 6H, 2×Me).

^{13}C NMR (150 MHz, $CDCl_3$, 298K) δ 192.1 (CHO), 192.0 (CHO), 158.3 (2×C, Ar), 157.2 (2×C, Ar), 155.6 (2×C, Ar), 146.3 (2×C, Ar), 139.9 (2×C, Ar), 138.8 (2×CH, Ar), 136.6 (4×C, Ar), 135.2 (2×C, Ar), 134.0 (2×C, Ar), 133.7 (4×C, Ar), 129.1 (4×CH, Ar), 127.7 (4×CH, Ar), 127.1 (4×CH, Ar), 127.0 (4×CH, Ar), 123.4 (2×CH, Ar), 122.1 (2×CH, Ar), 120.5 (2×CH, Ar), 76.9 (2×CH_2), 76.7 (2×CH_2), 69.5 (2×CH_2), 35.1 (2×CH_2), 31.1 (4×$CH_{2\,(bridging)}$), 23.4 (2×CH_2), 23.1 (2×CH_2), 10.7 (2×Me), 10.0 (2×Me).

HRMS (ESI$^+$) *m/z* calcd for $C_{68}H_{72}N_2O_8$ [M + 2H]$^{2+}$: 522.2638; found 522.2638.

5,5'-((((1^2,3^2,5^2,7^2-tetrapropoxy-1,3,5,7(1,3)-tetrabenzenacyclooctaphane-1^5,5^5-diyl)bis(4,1-phenylene))bis(ethane-2,1-diyl))bis(oxy))bis(2-(bromomethyl)pyridine) (11)

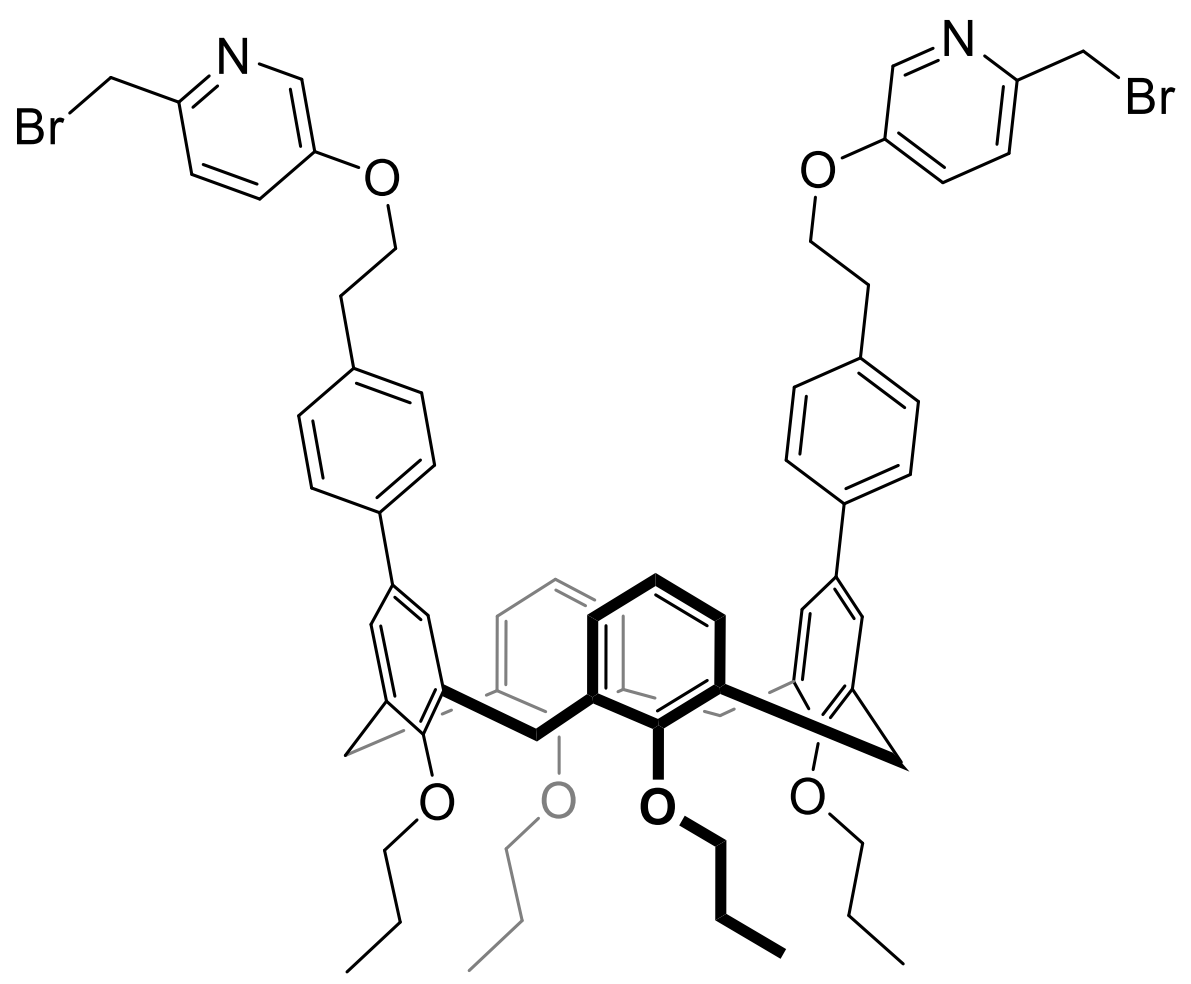

Chemical Formula: $C_{68}H_{72}Br_2N_2O_6$
Molecular Weight: 1173.14

Step 1: dialdehyde **SI-2** (218 mg, 209 µmol) was dissolved in a 1:1 mixture of anhydrous MeOH and DCM (10.8 mL) and the resulting solution was cooled to 0 °C in an ice bath. Then, $NaBH_4$ (16.3 mg, 430 µmol) was added and the resulting reaction mixture was stirred at room temperature for 3 hours. Water (10 mL) was added and the reaction mixture was extracted with CH_2Cl_2 (3 × 10 mL). The combined organic layers were washed with brine, dried over Na_2SO_4, filtered and concentrated *in vacuo*

to give the desired crude product as a white solid, which was used in the following step with no further purification.

Step 2: crude product from the previous step was dissolved in anhydrous DCM (2.10 mL) and the resulting solution was cooled to 0 °C in an ice bath. Then PBr_3 (39.1 µL, 113 mg, 0.418 mmol) was added and the resulting reaction mixture was allowed to warm up to room temperature and stirred overnight. After the reaction is complete, it was quenched with saturated $NaHCO_3$ aqueous solution (3.0 mL) and extracted with DCM (3 x 2.5 mL), washed with brine (5.0 mL), dried over Na_2SO_4, filtered and concentrated *in vacuo*. Purification by flash column chromatography (A: cyclohexane, B: ethyl acetate, gradient: 0-30% of B, 24 g RediSep® silica column, run length = 29 CV, eluted at 22% of B, 22-27 CV) gave the product **11** as a light-tan solid.

Yield: 213 mg, 87%.

Mp.: 214 – 215 °C (decomposition).

R*f* 0.40 (SiO_2, cyclohexane / ethyl acetate, 2:1).

IR (ATR, ν/cm^{-1}) 2958 (w), 2924 (w), 2872 (w), 1593 (w), 1575 (w), 1464 (s), 1390 (w), 1268 (s), 1249 (s), 1197 (s), 1085 (m), 1003 (s), 823 (s), 761 (s), 611 (m), 528 (w), 504 (w).

^{1}H NMR (500 MHz, $CDCl_3$, 298K) δ 8.27 (dd, ^4J = 2.9, ^5J = 0.4 Hz, 2H, 2×CH, Ar), 7.41 (d, ^3J = 8.1 Hz, 4H, 4×CH, Ar), 7.34 (dd, ^3J = 8.5, ^5J = 0.4 Hz, 2H, 2×CH, Ar), 7.22 (d, ^3J = 8.1 Hz, 4H, 4×CH, Ar), 7.16 (dd, ^3J = 8.5, ^4J = 2.9 Hz, 2H, 2×CH, Ar), 7.14 (s, 4H, 4×CH, Ar), 6.37 (s, 6H, 6×CH, Ar), 4.54 (s, 4H, 2×CH_2), 4.50 (d, ^2J = 13.3 Hz, 4H, 4×$CH_{2\ (bridging)}$), 4.23 (t, ^3J = 7.0 Hz, 4H, 2×CH_2), 4.06 – 3.91 (m, 4H, 2×CH_2), 3.79 (t, ^3J = 7.0 Hz, 4H, 2×CH_2), 3.21 (d, ^2J = 13.3 Hz, 4H, 4×$CH_{2\ (bridging)}$), 3.11 (t, ^3J = 7.0 Hz, 4H, 2×CH_2), 2.02 – 1.90 (m, 8H, 4×CH_2), 1.08 (t, ^3J = 7.5 Hz, 6H, 2×Me), 0.95 (t, ^3J = 7.5 Hz, 6H, 2×Me).

^{13}C NMR (150 MHz, $CDCl_3$, 298K) δ 157.1 (2×C, Ar), 155.7 (2×C, Ar), 154.4 (2×C, Ar), 148.7 (2×C, Ar), 139.7 (2×C, Ar), 137.7 (2×CH, Ar), 136.4 (4×C, Ar), 135.5 (2×C, Ar), 134.1 (2×C, Ar), 133.8 (4×C, Ar), 129.1 (4×CH, Ar), 127.8 (4×CH, Ar), 127.1 (4×CH, Ar), 127.0 (4×CH, Ar), 124.0 (2×CH, Ar), 122.1 (2×CH, Ar), 121.9 (2×CH, Ar), 76.9 (2×CH_2), 76.7 (2×CH_2), 69.2 (2×CH_2), 35.2 (2×CH_2), 33.8 (2×CH_2), 31.1 (4×$CH_{2\ (bridging)}$), 23.4 (2×CH_2), 23.1 (2×CH_2), 10.6 (2×Me), 10.0 (2×Me).

HRMS (ESI$^+$) *m/z* calcd for $C_{68}H_{73}Br_2N_2O_6$ [M + H]$^+$: 1171.3830; found 1171.3833.

Ligand monoCX_C2-(*S*,*S*-mcp) (12)

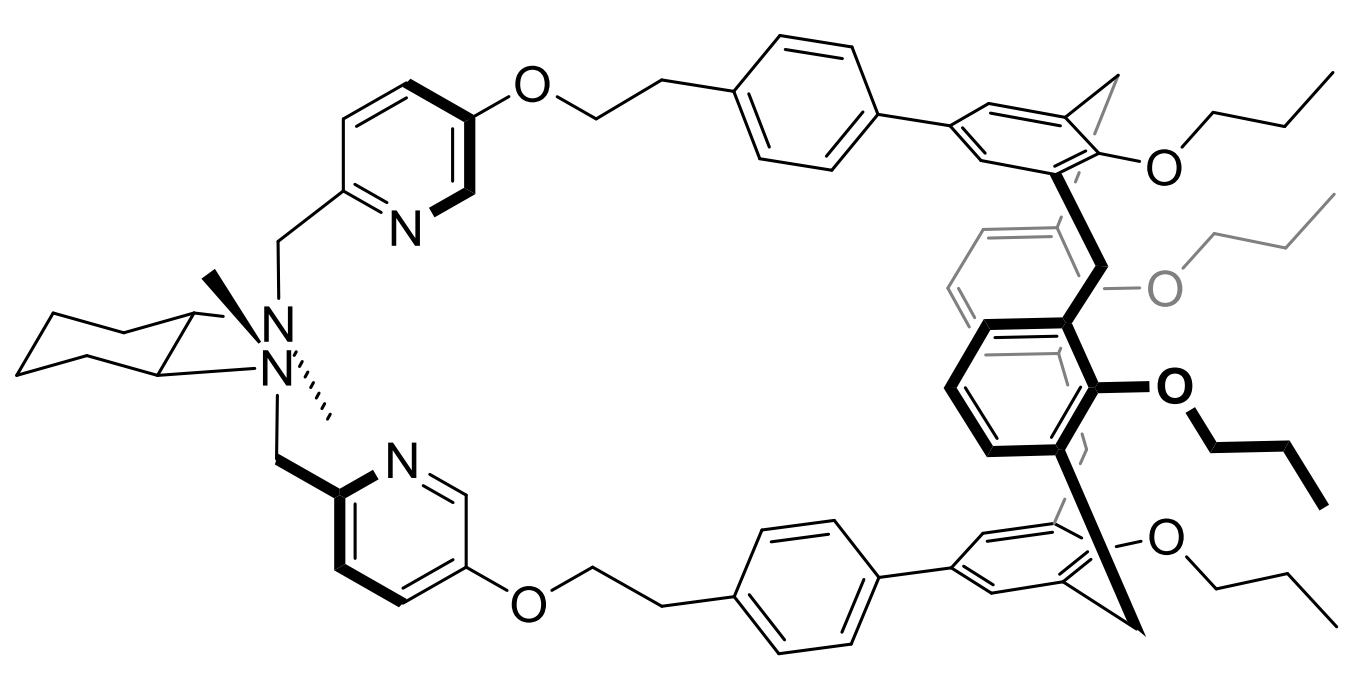

Chemical Formula: $C_{76}H_{88}N_4O_6$
Molecular Weight: 1153.56

Dibromide **11** (50.0 mg, 42.6 µmol) was dissolved in anhydrous MeCN (3.40 mL) in a 12 mL oven-dried vial under an inert atmosphere. Then K_2CO_3 (23.6 mg, 170 µmol) and TBAB (0.962 mg, 2.98 µmol) were added and the resulting mixture was stirred at 92° C for 15 minutes. A solution of (1*S*,2*S*)-

N,*N′*-dimethylcyclohexane-1,2-diamine (6.72 µL, 6.06 mg, 42.6 µmol) in anhydrous MeCN (1.00 mL) was then added dropwise over 1 hour, the cap with septum was changed and the reaction mixture was stirred overnight at 92° C for 20 hours. After that, reaction mixture was cooled down to room temperature, filtered, and washed with CH_2Cl_2 (3 × 2.0 mL). The filtrate was concentrated under reduced pressure, 1 M NaOH (5.0 mL) added, and extracted with CH_2Cl_2 (3 × 2.0 mL). Combined organic phases were washed with brine (4.0 mL), dried over Na_2SO_4, filtered and solvent was removed *in vacuo*. The crude material was purified by flash column chromatography (A: cyclohexane, B: ethyl acetate, gradient: 0-100% of B, 8 g RediSep® basic Al_2O_3 column, run length = 55 CV, eluted at 25% of B, 20-38 CV) to yield ligand **12** as a white solid.

Yield: 28 mg, 57%.

Mp.: 147 – 148 °C.

$\mathbf{R}_f$ 0.57 (Al_2O_3, dichloromethane / methanol, 8:1).

IR (ATR, ν/cm^{-1}) 2958 (w), 2924 (m), 2873 (w), 2856 (w), 1569 (w), 1461 (s), 1383 (m), 1227 (s), 1208 (s), 1083 (w), 1028 (m), 1005 (s), 964 (m), 885 (w), 821 (m), 764 (m), 618 (w), 574 (w).

^{1}H NMR (600 MHz, $CDCl_3$, 298K) δ 8.14 (d, ^4J = 2.9 Hz, 2H, 2×CH, Ar), 7.15 (d, ^3J = 7.5 Hz, 4H, 4×CH, Ar), 7.03 (d, ^3J = 8.6, Hz, 2H, 2×CH, Ar), 6.93 (t, ^3J = 7.5 Hz, 2H, 2×CH, Ar), 6.84 (d, ^3J = 8.1 Hz, 4H, 4×CH, Ar), 6.80 (dd, ^3J = 8.6, ^4J = 3.0 Hz, 2H, 2×CH, Ar), 6.68 (d, ^3J = 8.1 Hz, 4H, 4×CH, Ar), 6.40 (s, 4H, 4×CH, Ar), 4.51 (d, ^2J = 13.3 Hz, 4H, 4×$CH_{2\,(bridging)}$), 4.15 – 4.05 (m, 8H, 4×CH_2), 3.77 (d, ^2J = 14.3 Hz, 2H, 2×CH_2), 3.70 (t, ^3J = 7.1 Hz, 4H, 2×CH_2), 3.42 (d, ^2J = 14.3 Hz, 2H, 2×CH_2), 3.21 (d, ^2J = 13.3 Hz, 4H, 4×$CH_{2\,(bridging)}$), 2.96 (t, ^3J = 7.1 Hz, 4H, 2×CH_2), 2.75 – 2.67 (m, 2H, 2×CH), 2.29 (s, 6H, 2×Me), 2.14 – 2.06 (m, 4H, 2×CH_2), 2.05 – 2.01 (m, 2H, 2×CH_2), 1.97 – 1.88 (m, 4H, 2×CH_2), 1.85 – 1.78 (m, 2H, 2×CH_2), 1.31 – 1.21 (m, 4H, 2×CH_2), 1.11 (t, ^3J = 7.5 Hz, 6H, 2×Me), 0.93 (t, ^3J = 7.5 Hz, 6H, 2×Me).

^{13}C NMR (150 MHz, $CDCl_3$, 298K) δ 157.6 (2×C, Ar), 154.7 (2×C, Ar), 153.8 (2×C, Ar), 153.2 (2×C, Ar), 140.0 (2×C, Ar), 136.9 (2×CH), 136.7 (4×C, Ar), 134.8 (2×C, Ar), 134.6 (2×C, Ar), 133.1 (4×C, Ar), 128.9 (4×CH), 128.4 (4×CH), 126.9 (4×CH), 126.4 (2×CH), 126.3 (2×CH), 123.6 (2×CH), 122.3 (2×CH), 120.6 (2×CH), 77.4 (2×CH_2), 76.5 (2×CH_2), 68.9 (2×CH_2), 65.8 (2×CH), 57.5 (2×CH_2), 36.9 (2×Me), 35.1 (2×CH_2), 31.0 (4×$CH_{2\,(bridging)}$), 25.9 (2×CH_2), 24.2 (2×CH_2), 23.6 (2×CH_2), 22.9 (2×CH_2), 10.8 (2×Me), 9.8 (2×Me).

HRMS (ESI$^+$) *m/z* calcd for $C_{76}H_{89}N_4O_6$ $[M + H]^+$: 1153.6777; found 1153.6781.

Catalyst monoCX_C2-Mn(*S*,*S*-mcp) (5)

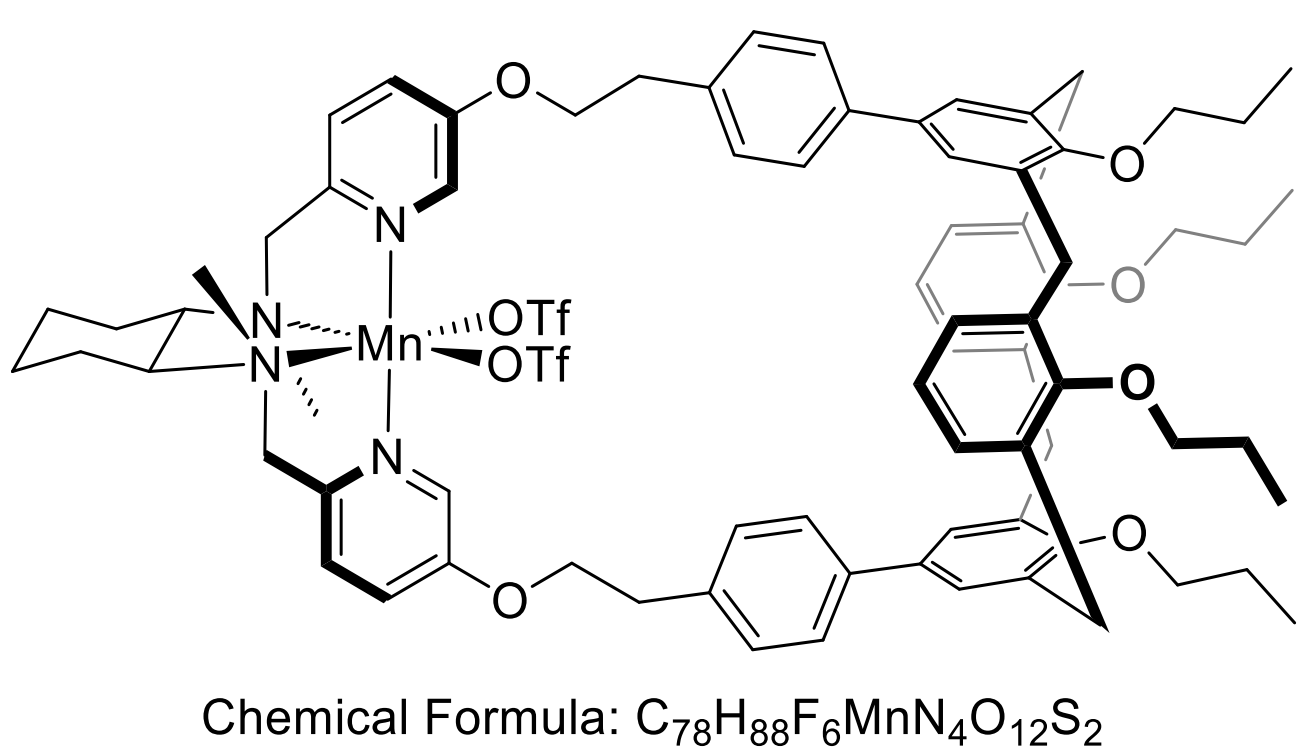

Chemical Formula: $C_{78}H_{88}F_6MnN_4O_{12}S_2$
Molecular Weight: 1506.63

A 4 mL oven-dried vial was charged with ligand **12** (14.3 mg, 12.4 µmol) and a 2 mL oven-dried vial with manganese bis(trifluoromethanesulfonate) (4.60 mg, 13.0 µmol). In a glovebox with constant 15

°C temperature, $Mn(OTf)_2$ was transferred to the vial containing **12** and the 2 mL vial was rinsed with anhydrous degassed (freeze-pump-thaw method) tetrahydrofuran (2 × 210 μL), and the suspensions were added to the 4 mL vial. The resulting reaction mixture was stirred at 15 °C for 5 hours. After that, precipitation and washing steps were done. Anhydrous degassed (freeze-pump-thaw method) hexane (3.0 mL) was added under vigorous stirring and a precipitate was formed. The stirring was discontinued and the mixture was left to stand for 10 minutes to let the solids deposit on the bottom of the vial. The supernatant was removed and the washing step with hexane was repeated two more times. The resulting solid was dried *in vacuo* to give desired catalyst **5** as an off-white solid. The catalyst was stored in a glovebox to prevent decomposition.

Yield: 17 mg, 91%.

Mp.: 217 – 218 °C (decomposition: turning black at that point).

IR (ATR, ν/cm^{-1}) 3467 (w), 2960 (w), 2937 (w), 2874 (w), 1502 (w), 1463 (m), 1251 (s), 1187 (m), 1155 (w), 1031 (s), 1007 (m), 829 (w), 768 (w), 638 (s), 518 (m).

HRMS (ESI$^+$) *m/z* calcd for $C_{77}H_{88}F_3MnN_4O_9S$ [M – OTf]$^+$: 1356.5599; found 1356.5594.

2.3 Synthesis of substrates

2-Decylisoindoline-1,3-dione (S10)

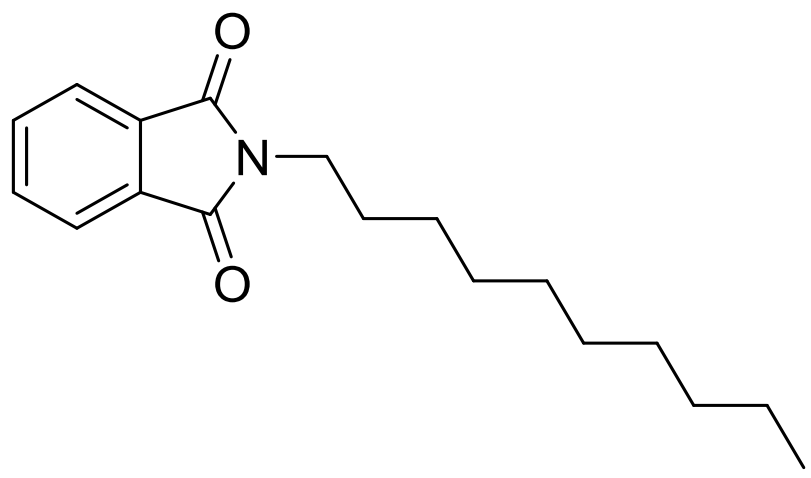

Chemical Formula: $C_{18}H_{25}NO_2$
Molecular Weight: 287.40

Phthalic anhydride (194 µL, 296 mg, 2.00 mmol) and the decylamine (400 µL, 315 mg, 2.00 mmol) were added to a 25 mL round-bottom flask equipped with a magnetic stirring bar and a condenser. *N*,*N*-Dimethylformamide (4.00 mL) was added and the stirred mixture was heated at 120 °C in an oil bath for 12 h. The warm reaction mixture was then poured onto crushed ice prepared in a beaker. The resulting precipitate was filtered, rinsed with deionized water (3 × 2.0 mL) and then dried under vacuum. The obtained crude product was further purified by flash column chromatography (A: cyclohexane, B: ethyl acetate, gradient: 0-11% of B, 24 g RediSep® silica column, run length = 17 CV, eluted at 6% of B, 11.5-14.0 CV) to give compound **S10** as white solid.

Yield: 402 mg, 70%.

^{1}H NMR (500 MHz, $CDCl_3$, 298K) δ 7.86 – 7.80 (m, 2H, 2×CH, Ar), 7.72 – 7.68 (m, 2H, 2×CH, Ar), 3.70 – 3.64 (m, 2H, CH_2), 1.71 – 1.62 (m, 2H, CH_2), 1.37 – 1.19 (m, 14H, 7×CH_2), 0.87 (t, ^3J = 7.0 Hz, 3H, Me).

^{13}C NMR (125 MHz, $CDCl_3$, 298K) δ 168.5 (2×C=O), 133.8 (2×CH, Ar), 132.2 (2×C, Ar), 123.1 (2×CH, Ar), 38.1 (CH_2), 31.9 (CH_2), 29.50 (CH_2), 29.47 (CH_2), 29.3 (CH_2), 29.2 (CH_2), 28.6 (CH_2), 26.9 (CH_2), 22.7 (CH_2), 14.1 (Me).

^{1}H and ^{13}C NMR-shifts match the reported values.[8]

***N*-Decyl-4-methylbenzenesulfonamide (S11)**

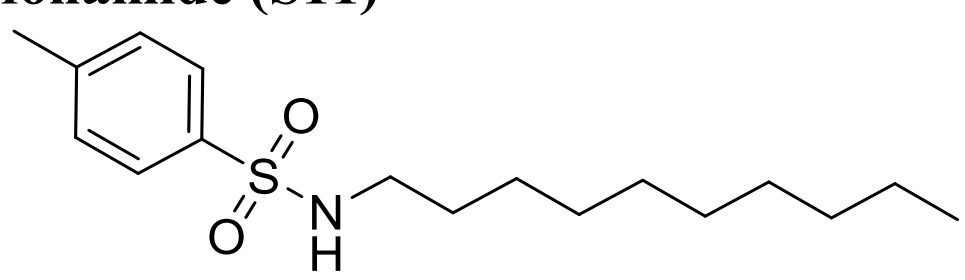

Chemical Formula: $C_{17}H_{29}NO_2S$
Molecular Weight: 311.48

To a stirred solution of decylamine (400 µL, 315 mg, 2.00 mmol) and Et_3N (416 µL, 3.00 mmol) in dry DCM (10.0 mL) at 0 °C TsCl (458 mg, 2.40 mmol) was added and reaction mixture was stirred overnight at room temperature. After completion, the reaction mixture was acidified by addition of 1 M aqueous HCl (10 mL) and the aqueous layer was extracted with DCM (3 × 4.0 mL). The combined organic layers were dried over Na_2SO_4 and concentrated *in vacuo*. The crude product was further purified by flash column chromatography (A: cyclohexane, B: ethyl acetate, gradient: 0-25% of B, 24 g RediSep® silica column, run length = 33 CV, eluted at 9% of B, 14-20 CV) to give compound **S11** as white solid.

Yield: 602 mg, 97%.

^{1}H NMR (500 MHz, $CDCl_3$, 298K) δ 7.74 (d, ^3J = 8.3, Hz, 2H, 2×CH, Ar), 7.31 (d, ^3J = 8.3, Hz, 2H, 2×CH, Ar), 4.33 (t, ^3J = 6.2, Hz, 1H, NH), 2.93 (td, ^3J = 6.9, Hz, ^3J = 6.2, Hz, 2H, CH_2), 2.43 (s, 3H, Me), 1.49 – 1.40 (m, 2H, CH_2), 1.33 – 1.16 (m, 14H, 7×CH_2), 0.87 (t, ^3J = 7.0 Hz, 3H, Me).

^{13}C NMR (125 MHz, $CDCl_3$, 298K) δ 143.3 (C, Ar), 137.0 (C, Ar), 129.7 (2×CH, Ar), 127.1 (2×CH, Ar), 43.2 (CH_2), 31.8 (CH_2), 29.6 (CH_2), 29.45 (CH_2), 29.41 (CH_2), 29.2 (CH_2), 29.0 (CH_2), 26.5 (CH_2), 22.6 (CH_2), 21.5 (Me), 14.1 (Me).

^{1}H and ^{13}C NMR-shifts match the reported values.[9]

N-Decylmethanesulfonamide (S12)

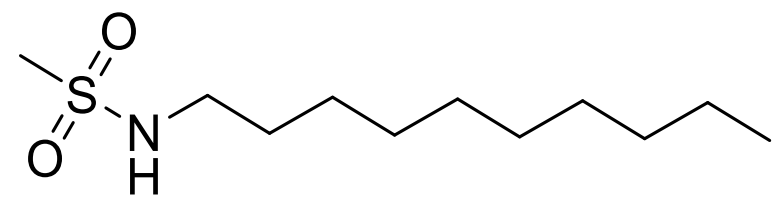

Chemical Formula: $C_{11}H_{25}NO_2S$
Molecular Weight: 235.39

To a stirred solution of decylamine (400 µL, 315 mg, 2.00 mmol) and Et_3N (416 µL, 3.00 mmol) in dry DCM (10.0 mL) at 0 °C MsCl (186 µL, 275 mg, 2.40 mmol) was added and reaction mixture was stirred overnight at room temperature. After completion, the reaction mixture was acidified by addition of 1 M aqueous HCl (10 mL) and the aqueous layer was extracted with DCM (3 × 4.0 mL). The combined organic layers were dried over Na_2SO_4 and concentrated *in vacuo*. The crude product was further purified by flash column chromatography (A: cyclohexane, B: ethyl acetate, gradient: 0-60% of B, 24 g RediSep® silica column, run length = 33 CV, eluted at 24% of B, 15.0-18.5 CV) to give compound **S12** as white solid.

Yield: 442 mg, 94%.

Mp.: 70 – 71 °C.

IR ν_{max}(thin film)/cm^{-1} 3261 (m), 2951 (w), 2914 (s), 2846 (m), 1467 (w), 1430 (m), 1414 (m), 1230 (s), 1140 (s), 1062 (m), 1036 (w), 999 (w), 980 (w), 890 (w), 769 (m), 717 (w), 635 (w), 526 (s), 512 (s), 454 (w).

^{1}H NMR (500 MHz, $CDCl_3$, 298K) δ 4.23 (br s, 1H, NH), 3.17 – 3.07 (m, 2H, CH_2), 2.95 (s, 3H, Me), 1.60 – 1.53 (m, 2H, CH_2), 1.41 – 1.19 (m, 14H, 7×CH_2), 0.88 (t, ^3J = 7.0 Hz, 3H, Me).

^{13}C NMR (125 MHz, $CDCl_3$, 298K) δ 43.4 (CH_2), 40.3 (Me), 31.8 (CH_2), 30.1 (CH_2), 29.48 (CH_2), 29.46 (CH_2), 29.3 (CH_2), 29.1 (CH_2), 26.6 (CH_2), 22.6 (CH_2), 14.1 (Me).

HRMS (ESI$^+$) *m/z* calcd for $C_{11}H_{26}NO_2S$ $[M + H]^+$: 236.1679; found 236.1678.

(1*R*,2*S*,5*R*)-2-Isopropyl-5-methylcyclohexyl dodecanoate ((-)-Menthyl laurate; S13)

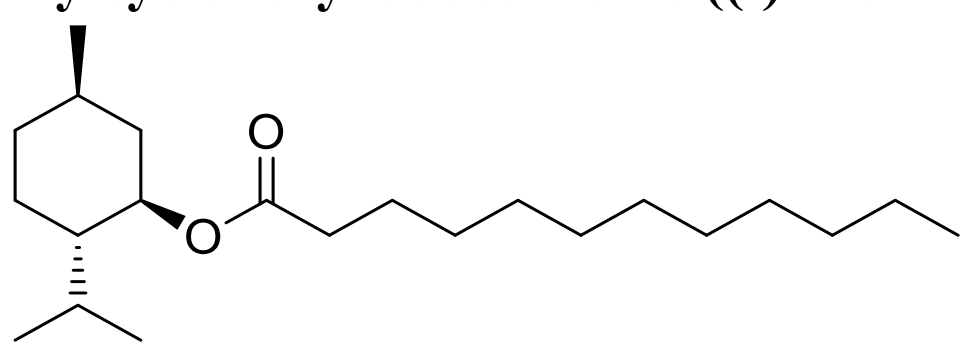

Chemical Formula: $C_{22}H_{42}O_2$
Molecular Weight: 338.58

L-Menthol (500 mg, 3.20 mmol) was suspended in lauroylchlorid (837 µL, 770 mg, 3.52 mmol). MsOH (4.15 µL, 64.0 µmol) was added and the mixture was stirred at room temperature overnight. After completion, the reaction mixture was poured into an ice-cold saturated $NaHCO_3$ aqueous solution (15

mL). The mixture was extracted with ethyl acetate (3 x 5.0 mL). The combined organic layers were washed with brine (10 mL), dried over Na_2SO_4, filtered and concentrated *in vacuo*. The crude product was further purified by flash column chromatography (A: cyclohexane, B: ethyl acetate, gradient: 0-5% of B, 24 g RediSep® silica column, run length = 20 CV, eluted at 0.25% of B, 3-8 CV) to give compound **S13** as colourless oil.

Yield: 1.02 g, 95%.

^{1}H NMR (500 MHz, $CDCl_3$, 298K) δ 4.67 (td, ^3J = 10.9 Hz, ^3J = 4.4 Hz, 1H, CH), 2.30 – 2.23 (m, 2H, CH_2), 2.00 – 1.94 (m, 1H, CH_2), 1.89 – 1.82 (m, 1H, CH), 1.70 – 1.63 (m, 2H, CH_2+CH_2), 1.63 – 1.55 (m, 2H, CH_2), 1.53 – 1.43 (m, 1H, CH), 1.39 – 1.32 (m, 1H, CH), 1.32 – 1.20 (m, 16H, 8×CH_2), 1.09 – 0.99 (m, 1H, CH_2), 0.98 – 0.90 (m, 1H, CH_2), 0.90 – 0.80 (m, 10H, CH+3×Me), 0.75 (d, ^3J = 7.0 Hz, 3H, Me).

^{13}C NMR (125 MHz, $CDCl_3$, 298K) δ 173.4 (C=O), 73.8 (CH), 47.0 (CH), 41.0 (CH_2), 34.7 (CH_2), 34.3 (CH_2), 31.9 (CH_2), 31.4 (CH), 29.59 (CH_2), 29.58 (CH_2), 29.5 (CH_2), 29.32 (CH_2), 29.25 (CH_2), 29.1 (CH_2), 26.2 (CH), 25.1 (CH_2), 23.4 (CH_2), 22.7 (CH_2), 22.0 (Me), 20.7 (Me), 16.3 (Me), 14.1 (Me).

^{1}H and ^{13}C NMR-shifts match the reported values.[10]

(*cis*-4-Isopropylcyclohexyl)methyl 4-methylbenzenesulfonate (SI-3)

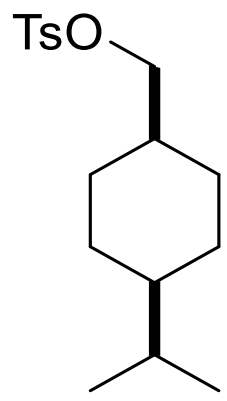

Chemical Formula: $C_{17}H_{26}O_3S$
Molecular Weight: 310.45

In an oven-dried 12 mL vial a mixture of *cis*-*p*-menthan-7-ol (1.11 mL, 1.00 g, 6.40 mmol) and dry pyridine (3.60 mL) was cooled in a water bath and TsCl (1.87 g, 9.60 mmol) was added. The reaction was stirred at room temperature overnight and then carefully poured over a mixture of ice (15 mL) and concentrated aqueous HCl (5.5 mL) and sonicated. The mixture was transferred to a separation funnel and the aqueous layer was extracted with DCM (3 × 10 mL). The combined organic layers were washed with water (4 × 5.0 mL), dried over Na_2SO_4, filtered and concentrated *in vacuo*. The crude product was further purified by flash column chromatography (A: cyclohexane, B: DCM, gradient: 0-70% of B, 24 g RediSep® silica column, run length = 23 CV, eluted at 31% of B, 11-18 CV) to give a mixture of *cis*- and *trans*-isomers of compound **SI-3** as colourless oil.

In order to separate *cis*-isomer from *trans*-isomer recycling GPC was used. Separation was carried out in a single run on a 250 mg scale of the isomeric mixture. After 53 cycles in total (26.5 hours) in chloroform, the largest peak (second in order of elution) was collected and the solvent evaporated. *cis*-Isomer of **SI-3** was obtained as colourless oil.

Yield: 1.99 g, quantitative (mixture of *cis*- and *trans*-isomers before recycling GPC); 126 mg, 72% (single run; only *cis*-isomer after recycling GPC).

IR ν_{max}(thin film)/cm^{-1} 2955 (w), 2922 (w), 2856 (w), 1597 (w), 1451 (w), 1358 (m), 1258 (w), 1171 (s), 1095 (m), 1017 (w), 976 (m), 941 (m), 927 (m), 832 (m), 810 (s), 783 (s), 664 (s), 569 (m), 553 (s), 524 (w).

^{1}H NMR (500 MHz, $CDCl_3$, 298K) δ 7.79 (d, ^3J = 8.3 Hz, 2H, 2×CH, Ar), 7.34 (d, ^3J = 8.3 Hz, 2H, 2×CH, Ar), 3.94 (d, ^3J = 7.5 Hz, 2H, CH_2), 2.44 (s, 3H, Me), 1.97 – 1.85 (m, 1H, CH), 1.50 – 1.33 (m, 7H, CH+3×CH_2), 1.17 – 1.07 (m, 2H, CH_2), 1.07 – 0.99 (m, 1H, CH), 0.79 (d, ^3J = 6.7 Hz, 6H, 2×Me).

^{13}C NMR (125 MHz, $CDCl_3$, 298K) δ 144.6 (C, Ar), 133.1 (C, Ar), 129.7 (2×CH, Ar), 127.9 (2×CH, Ar), 72.6 (CH_2), 42.5 (CH), 33.8 (CH), 30.3 (CH), 25.7 (2×CH_2), 25.1 (2×CH_2), 21.6 (Me), 20.1 (2×Me).

HRMS (ESI$^+$) *m/z* calcd for $C_{17}H_{26}O_3SNa$ $[M + Na]^+$: 333.1495; found 333.1492.

(*trans*-4-Isopropylcyclohexyl)methyl 4-methylbenzenesulfonate

^{1}H NMR (500 MHz, $CDCl_3$, 298K) δ 7.77 (d, ^3J = 8.3, Hz, 2H, 2×CH, Ar), 7.33 (d, ^3J = 8.3, Hz, 2H, 2×CH, Ar), 3.80 (d, ^3J = 6.5, Hz, 2H, CH_2), 2.44 (s, 3H, Me), 1.77 – 1.65 (m, 4H, 2×CH_2), 1.61 – 1.53 (m, 1H, CH), 1.41 – 1.32 (m, 1H, CH), 0.99 – 0.84 (m, 5H, CH+2×CH_2), 0.82 (d, ^3J = 6.8 Hz, 6H, 2×Me).

^{13}C NMR (125 MHz, $CDCl_3$, 298K) δ 144.5 (C, Ar), 133.1 (C, Ar), 129.7 (2×CH, Ar), 127.8 (2×CH, Ar), 75.4 (CH_2), 43.7 (CH), 37.4 (CH), 32.7 (CH), 29.2 (2×CH_2), 28.7 (2×CH_2), 21.6 (Me), 19.7 (2×Me).

***cis*-1-Isopropyl-4-methylcyclohexane (S16)**

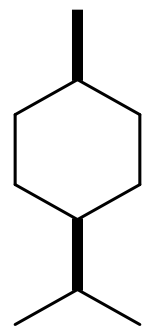

Chemical Formula: $C_{10}H_{20}$
Molecular Weight: 140.27

An oven-dried two neck 50 mL equipped with reflux condenser, stirring bar and rubber septum, under argon atmosphere, was charged with tosylate **SI-3** (500 mg, 1.61 mmol) and dry THF (1.90 mL) under argon atmosphere. The mixture was cooled in an ice bath to 0 °C and $LiEt_3BH$ solution (1.0 M in THF; 4.83 mL, 4.83 mmol) was added. The reaction mixture was allowed to warm up to room temperature and then stirred at 50°C for 2 hours. After that the temperature was increased to 75 °C and stirred for additional 22 h. The excess of $LiEt_3BH$ was quenched with H_2O (5.0 mL) and the organoborane was oxidized with a mixture of 3 M NaOH aqueous solution (3.5 mL) and 50 wt% H_2O_2 aqueous solution (2.1 mL). The organic layer was separated and the aqueous layer was extracted with pentane (3 x 10 mL) and the combined organic layers were washed with H_2O (4 x 8.0 mL), dried over Na_2SO_4. Solvents were carefully removed *in vacuo* to give the desired product **S16** as a colorless liquid.

Yield: 134 mg, 59%.

IR ν_{max}(thin film)/cm^{-1} 2949 (s), 2914 (s), 2862 (s), 2848 (s), 1459 (m), 1451 (m), 1381 (m), 1364 (m), 1169 (w), 968 (w), 631 (w).

^{1}H NMR (500 MHz, $CDCl_3$, 298K) δ 1.76 – 1.69 (m, 1H, CH), 1.56 – 1.50 (m, 1H, CH), 1.49 – 1.43 (m, 2H, CH_2), 1.42 – 1.36 (m, 4H, 2×CH_2), 1.36 – 1.31 (m, 2H, CH_2), 1.08 – 1.01 (m, 1H, CH), 0.92 (d, ^3J = 7.0 Hz, 3H, Me), 0.87 (d, ^3J = 6.7 Hz, 6H, 2×Me).

^{13}C NMR (125 MHz, $CDCl_3$, 298K) δ 43.1 (CH), 31.4 (2×CH_2), 30.5 (CH), 29.2 (CH), 25.4 (2×CH_2), 20.3 (2×Me), 19.3 (Me).

HRMS (ESI$^+$) *m/z* calcd for $C_{10}H_{19}$ $[M – H]^+$: 139.1481; found 139.1483.

Octyl (2,3,4,6-tetra-*O*-acetyl)-β-D-glucopyranoside (S18)

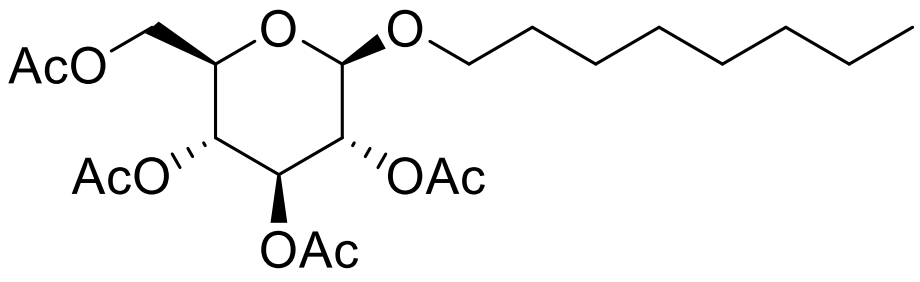

Chemical Formula: $C_{22}H_{36}O_{10}$
Molecular Weight: 460.52

In an oven-dried 12 mL vial a solution of octyl β-D-glucopyranoside (500 mg, 1.71 mmol) in dry pyridine (2.67 mL) was cooled in an ice bath and acetyl chloride (535 µL, 591 mg, 7.52 mmol) was added. The reaction was stirred in an ice bath for 1 h, then warmed up to room temperature and stirred overnight. After completion, the reaction mixture was carefully poured over a mixture of ice (10 mL) and concentrated aqueous HCl (2.5 mL) and sonicated. The mixture was transferred to a separation funnel and the aqueous layer was extracted with DCM (3 × 8.0 mL). The combined organic layers were washed with water (4 × 3.0 mL), dried over Na_2SO_4, filtered and concentrated *in vacuo*. The crude product was further purified by flash column chromatography (A: cyclohexane, B: ethyl acetate, gradient: 0-40% of B, 24 g RediSep® silica column, run length = 30 CV, eluted at 18% of B, 14-19 CV) to give compound **S18** as white solid.

Yield: 725 mg, 92%.

^{1}H NMR (500 MHz, $CDCl_3$, 298K) δ 5.20 (dd, ^3J = 9.6, ^3J = 9.3 Hz, 1H, CH), 5.08 (dd, ^3J = 10.0, ^3J = 9.3 Hz, 1H, CH), 4.98 (dd, ^3J = 9.6, ^3J = 8.0 Hz, 1H, CH), 4.49 (d, ^3J = 8.0 Hz, 1H, CH), 4.26 (dd, ^2J = 12.3, ^3J = 4.7 Hz, 1H, CH_2), 4.13 (dd, ^2J = 12.3, ^3J = 2.5 Hz, 1H, CH_2), 3.86 (dt, ^2J = 9.6, ^3J = 6.4 Hz, 1H, CH_2), 3.68 (ddd, ^3J = 10.0, ^3J = 4.7, ^3J = 2.5 Hz, 1H, CH), 3.46 (dt, ^2J = 9.6, ^3J = 6.8 Hz, 1H, CH_2), 2.08 (s, 3H, MeCO), 2.03 (s, 3H, MeCO), 2.02 (s, 3H, MeCO), 2.00 (s, 3H, MeCO), 1.64 – 1.48 (m, 2H, CH_2), 1.34 – 1.21 (m, 10H, 5×CH_2), 0.87 (t, ^3J = 6.9 Hz, 3H, Me).

^{13}C NMR (125 MHz, $CDCl_3$, 298K) δ 170.7 (Me*C*O), 170.3 (Me*C*O), 169.4 (Me*C*O), 169.3 (Me*C*O), 100.8 (CH), 72.9 (CH), 71.7 (CH), 71.4 (CH), 70.3 (CH_2), 68.5 (CH), 62.0 (CH_2), 31.8 (CH_2), 29.4 (CH_2), 29.24 (CH_2), 29.23 (CH_2), 25.8 (CH_2), 22.6 (CH_2), 20.7 (*Me*CO), 20.63 (*Me*CO), 20.62 (*Me*CO), 20.59 (*Me*CO), 14.1 (Me).

^{1}H and ^{13}C NMR-shifts match the reported values.[11]

Heptyl 4-(pivaloyloxy)benzoate (S19)

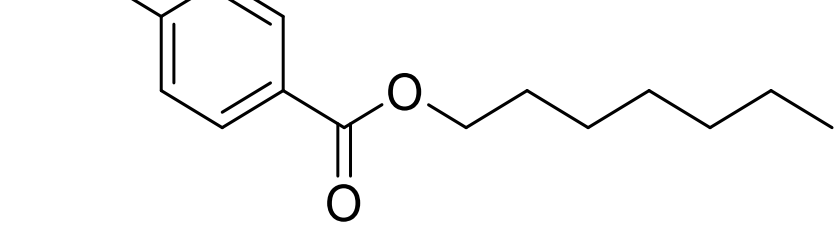

Chemical Formula: $C_{19}H_{28}O_4$
Molecular Weight: 320.43

In an oven-dried 12 mL vial a suspention of heptyl 4-hydroxybenzoate (300 mg, 1.27 mmol) and dry pyridine (126 µL, 124 mg, 1.56 mmol) in DCM (3.10 mL) was cooled in an ice bath and pivalyl chloride (173 µL, 170 mg, 1.41 mmol) was slowly added. The reaction was stirred in an ice bath for 1 h, then warmed up to room temperature and stirred for additional 3 h. After completion, the reaction mixture was transferred to a separation funnel and diluted with DCM (10 mL). The organic layer was washed with water (3 × 4.0 mL), brine (5.0 mL), dried over Na_2SO_4, filtered and concentrated *in vacuo*. The crude product was further purified by flash column chromatography (A: cyclohexane, B: ethyl acetate, gradient: 0-15% of B, 12 g RediSep® silica column, run length = 45 CV, eluted at 3% of B, 9-13 CV) to give compound **S19** as colorless liquid.

Yield: 394 mg, 97%.

IR ν_{max}(thin film)/cm^{-1}.2958 (w), 2929 (m), 2857 (w), 1754 (m), 1719 (s), 1603 (w), 1505 (w), 1480 (w), 1461 (w), 1412 (w), 1270 (s), 1202 (m), 1161 (m), 1091 (s), 1015 (m), 900 (w), 860 (w), 757 (m), 692 (w), 650 (w), 506 (w).

^{1}H NMR (500 MHz, $CDCl_3$, 298K) δ 8.06 (d, ^3J = 8.9 Hz, 2H, 2×CH, Ar), 7.13 (d, ^3J = 8.9 Hz, 2H, 2×CH, Ar), 4.31 (t, ^3J = 6.7 Hz, 2H, CH_2), 1.80 – 1.70 (m, 2H, CH_2), 1.46 – 1.40 (m, 2H, CH_2), 1.40 – 1.33 (m, 11H, Me_3C + CH_2), 1.33 – 1.25 (m, 4H, 2×CH_2), 0.89 (t, ^3J = 6.9 Hz, 3H, Me).

^{13}C NMR (125 MHz, $CDCl_3$, 298K) δ 176.6 (C=O), 166.0 (C=O), 154.7 (C, Ar), 131.0 (2×CH, Ar), 127.9 (C, Ar), 121.5 (2×CH, Ar), 65.2 (CH_2), 39.2 (Me_3C), 31.7 (CH_2), 28.9 (CH_2), 28.7 (CH_2), 27.1 (Me_3C), 26.0 (CH_2), 22.6 (CH_2), 14.1 (Me).

HRMS (ESI$^+$) *m/z* calcd for $C_{19}H_{29}O_4$ $[M + H]^+$: 321.2060; found 321.2058.

3. Oxidation studies

3.1 Catalysts's dependance on TfOH concentration

In certain catalyst batches, diminished yields were observed. Triflic acid (TfOH) has been demonstrated to act as an effective additive for improving yields in certain reactions.[12–14]

Procedure: 2,2,2-Trifluoroethanol (TFE, 200 µL), octane (2.98 µL, 2.11 mg, 18.5 µmol), 2,2-dimethylbutanoic acid (2.32 µL, 2.15 mg, 18.5 µmol) and 20 µL of TfOH solution in TFE (0–6 mol% of TfOH in relation to octane) were added to a 2 mL vial charged with catalyst **5** (0.279 mg, 0.185 µmol) and a stirring bar. The mixture was cooled to 0 °C. A solution of an 50% aqueous H_2O_2 diluted in TFE (20.6 µL; stock solution: 0.1 mL 50 % aqueous H_2O_2 in 1.9 mL TFE) was slowly added over 15 minutes (1.37 µL/min) under stirring via a syringe pump. After the addition, the mixture was left stirring for another 45 min.

Work-up and GC analysis: after the time indicated, H_2O (1.5 mL) and 0.0925 M biphenyl solution in MeCN (40.0 µL) were added and the mixture was extracted with HPLC grade EtOAc (1.0 mL × 2), washed with sat. $NaHCO_3$ (2.0 mL), dried over Na_2SO_4, filtered through a silica plug and rinsed with EtOAc.

IBX Oxidation: 0.5 mL of the mixture was transferred into another 2 mL vial equipped with a stirring bar. IBX (3.50 mg, 12.5 µmol) was added and the mixture was heated to 66 °C overnight. The mixture was allowed to cool down to room temperature, filtered through a silica plug and rinsed with EtOAc (1.0 mL × 2). GC-samples were prepared in hexane and analyzed by GC to check the TfOH dependance of the catalyst (Figure 1).

The following temperature-program for GC was used: Rtx-5; 60 °C for 3 min, 15 °C/min to 250 °C and 250 °C for 5 min.

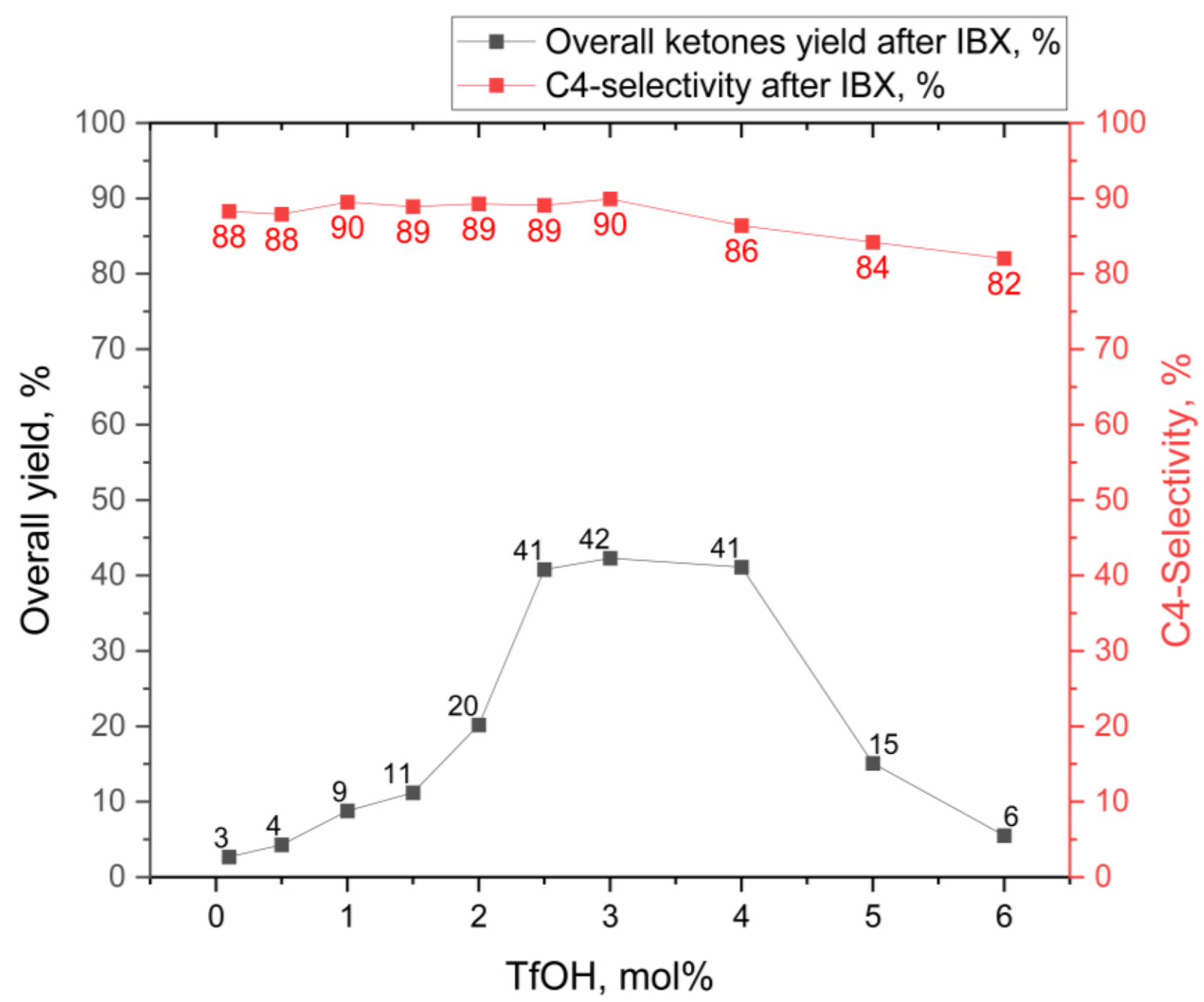

Figure 1. Yield and selectivity dependence on TfOH concentration for CH-oxidation reaction with catalyst **5**.

As a result, 20 µL of TfOH solution in TFE (2.5 mol% of TfOH in relation to octane) were added in each oxidation reaction.

3.2 Reaction condition optimization with model substrate octane

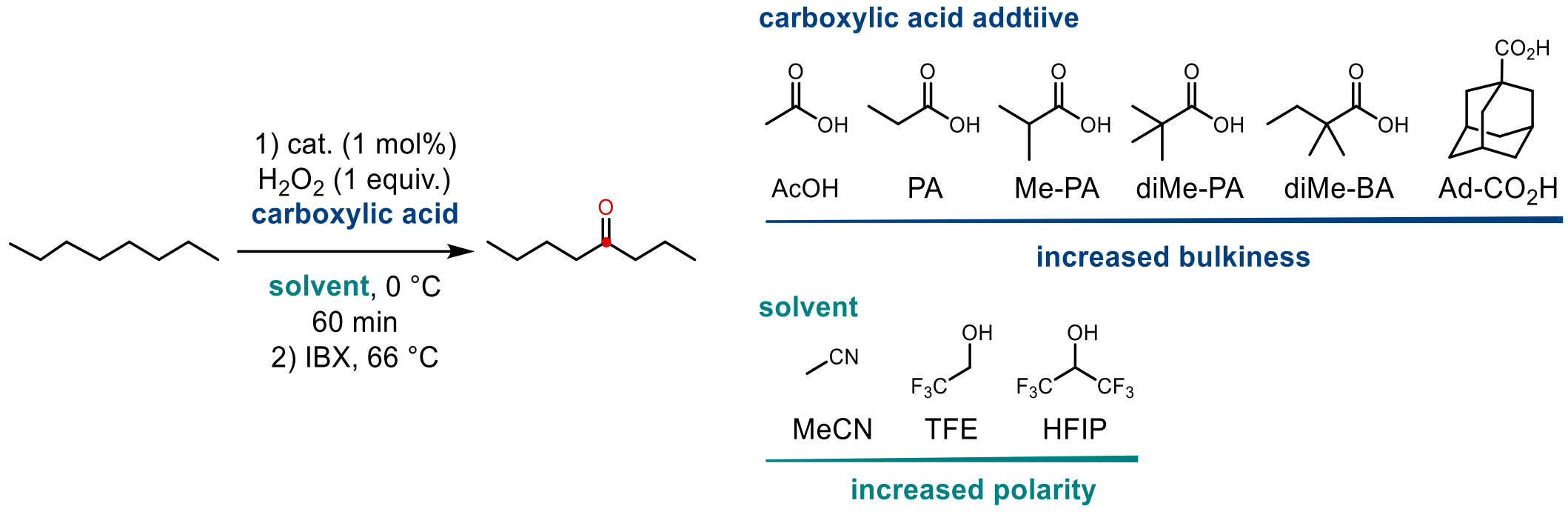

General procedure: solvent (200 µL), octane (2.98 µL, 2.11 mg, 18.5 µmol), carboxylic acid and 20 µL of TfOH solution in the chosen solvent (2.5 mol% of TfOH in relation to octane) were added to a 2 mL vial charged with catalyst (0.185 µmol), additive (0.185 µmol) and a stirring bar until otherwise stated. The mixture was cooled to 0 °C. A solution of an 50% aqueous H_2O_2 diluted in TFE (20.6 µL; stock solution: 0.1 mL 50 % aqueous H_2O_2 in 1.9 mL TFE) was slowly added over 15 minutes (1.37 µL/min) under stirring via a syringe pump. After the addition, the mixture was left stirring for another 45 min.

Work-up and GC analysis: after the time indicated, H_2O (1.5 mL) and 0.0925 M biphenyl solution in MeCN (40.0 µL) were added and the mixture was extracted with HPLC grade EtOAc (1.0 mL × 2), washed with sat. $NaHCO_3$ (2.0 mL), dried over Na_2SO_4, filtered through a silica plug and rinsed with EtOAc.

IBX oxidation: 0.5 mL of the mixture was transferred into another 2 mL vial equipped with a stirring bar. IBX (3.50 mg, 12.5 µmol) was added and the mixture was heated to 66 °C overnight. The mixture was allowed to cool down to room temperature, filtered through a silica plug and rinsed with EtOAc (1.0 mL × 2). GC-samples were prepared in hexane and analyzed by GC.

Reaction optimization was initiated by examining the influence of the solvent, which we consider to be the primary factor of the observed changes in selectivity. Three solvents (MeCN, TFE and HFIP) were selected due to their compatibility with the oxidation system[15] and their distinctly different polarity properties. Solvent polarity was evaluated using the normalized empirical solvent polarity parameter (E_T^N), which has been shown to correlate well with the binding strength of apolar guests.[16]

Within this series, solvent polarity increases from MeCN (E_T^N = 0.460) to TFE (E_T^N = 0.898), with HFIP (E_T^N = 1.07) being the most polar one.[17] Notably, in comparison to water (E_T^N = 1.00), TFE approaches a similar polarity, while HFIP exceeds it. As summarized in the table below, the highest selectivity was obtained in TFE (entry 2), affording the desired product with 42% selectivity. In contrast, the lower polarity of MeCN (entry 1) appears insufficient, whereas in HFIP (entry 3) the even stronger solvent polarity is presumed to disturb the catalyst conformation, resulting in diminished selectivity. Accordingly, TFE was selected for further reaction optimization.

Entry	Cat. (mol%)	H_2O_2 addition, equiv. / min	Solvent	Carboxylic acid (equiv.)	Conversion / total yield (C4-yield), %	Site selectivity, %		
						C4	C3	C2
0	**2** (1)	1/15	MeCN	AcOH (22)	98/46 (13)	28	27	45
**Solvents**								
1	**5** (1)	1/15	MeCN	AcOH (22)	94/19 (5)	26	26	48
2			TFE		66/21 (9)	**42**	25	33
3			HFIP		68/44 (16)	36	28	36

In the subsequent stage of the study, attention was directed toward the influence of both the nature (entries 4–7, 10) and loading (entries 7–9) of the carboxylic acid employed as a coligand. A clear steric effect was observed: progression from the least bulky AcOH (entry 2) to the bulkiest diMe-BA (entry 7) resulted in a substantial increase in C4-oxidation selectivity, from 42% to 86%. We attribute this trend to coordination of the carboxylate ligand to the manganese center, whereby the alkyl substituent contributes to shaping the catalyst environment. Specifically, the steric bulk of the acid is proposed to define the third wall (first two walls are made by two linkers connecting calix[4]arene and catalytic core) of the catalytic pocket, thereby enhancing site selectivity. Within this series, diMe-BA provided the optimal performance, affording the highest selectivity.

The effect of carboxylic acid loading was subsequently examined. While variation in the amount of acid did not significantly influence regioselectivity (entries 7–9), a pronounced effect on yield was observed. Reducing the loading from 22 equivalents (entry 7) to 1 equivalent (entry 9) led to an increase in total product yield from 17% to 42%.

Entry	Cat. (mol%)	H_2O_2 addition, equiv. / min	Solvent	Carboxylic acid (equiv.)	Conversion / total yield (C4-yield), %	Site selectivity, %		
						C4	C3	C2
**Carboxylic acids**								
4	**5** (1)	1/15	TFE	PA (22)	62/26 (15)	60	18	22
5				Me-PA (22)	58/23 (16)	70	15	15
6				diMe-PA (22)	70/40 (32)	80	11	9
7				diMe-BA (22)	56/17 (15)	86	8	6
8				diMe-BA (5)	75/34 (23)	87	6	7
9				diMe-BA (1)	75/42 (38)	**90**	8	2
10				Ad-CO_2H (1)	46/16 (13)	85	11	4

Subsequently, the amounts of the remaining reagents were varied. Doubling the quantity of H_2O_2 (entry 11), catalyst **5** (entry 12), or both H_2O_2 and catalyst **5** (entry 13) did not result in improved yields relative to entry 9. Considering the susceptibility of catalyst **5** to self-oxidation (see Section 3.4), a subsequent addition was tried. The addition of a second portion of both catalyst and H_2O_2 after 30 min (entry 14) led to an increase in yield compared to entry 9. Under these conditions, replacement of diMe-BA with diMe-PA (entry 15) resulted in a further enhancement of the yield while maintaining comparable selectivity (entry 14 versus entry 15), likely due to diMe-PA being less oxidation sensitive.

Entry	Cat. (mol%)	H_2O_2 addition, equiv. / min	Solvent	Carboxylic acid (equiv.)	Conversion / total yield (C4-yield), %	Site selectivity, %		
						C4	C3	C2
**Reagents ratio and addition pattern**								
11	**5** (1)	2/30	TFE	diMe-BA (1)	75/40 (36)	90	8	2
12	**5** (2)	1/15		diMe-BA (1)	72/31 (26)	86	6	8
13		2/30		diMe-BA (1)	73/42 (36)	87	9	4
14[a]	**5** (1+1)	1+1/15+15		diMe-BA (1)	94/55 (50)	91	7	2
15[a]				diMe-PA (1)	98/**67** (59)	**89**[d]	6	5

[a]Additional portion of catalyst (1 mol%) and H_2O_2 (0.9 M, 20.56 μL, 1.37 μL/min) were added after 30 minutes.

Following reaction optimization, the conditions corresponding to entry 15 were determined to provide the best so far overall performance and were therefore selected for evaluation of the substrate scope, as detailed in Section 4.

Further optimization of the H_2O_2 addition time revealed that the yield of oxidized products could be increased while preserving the selectivity of the process. Extending the H_2O_2 addition time from 7.5 to 60 min (entries SI-1–SI-5) led to an almost 50% increase in yield. Since entries SI-4 and SI-5 gave

comparable results, an addition time of 45 min was identified as optimal. Further increasing the amount of H_2O_2 while maintaining the same addition rate as in entry SI-4 (entry SI-6) did not lead to any additional improvement in yield.

Compared to entry 15, entry SI-4 provided comparable yield and C4-selectivity, while slightly improving the mass balance (31% vs. 20%) and usage of only 1 mol% of the catalyst **5**.

Entry	Cat. (mol%)	H_2O_2 addition, equiv. / min	Solvent	Carboxylic acid (equiv.)	Conversion / total yield (C4-yield), %	Site selectivity, %		
						C4	C3	C2
**H_2O_2 addition rate**								
SI-1	**5** (1)	1/7.5	TFE	diMe-PA (1)	73/48 (41)	86	10	4
SI-2		1/15			73/52 (45)	87	11	2
SI-3		1/30			92/68 (58)	85	11	4
SI-4		1/45			91/71 (64)	90	9	1
SI-5[b]		1/60			92/70 (61)	87	10	3
SI-6		1.2/54			91/70 (61)	88	10	2

[b]Reaction time was prolonged to 65 minutes: 60 minutes of H_2O_2 addition and 5 minutes of additional stirring.

Although entry SI-4 and entry 15 gave comparable results in terms of yield and selectivity, simply prolonging the H_2O_2 addition time did not lead to higher yields for all substrates. For substrate **S19**, entry SI-7 provided a higher yield, whereas entry SI-8 showed an improved mass balance. By combining prolonged H_2O_2 addition with subsequent addition of catalyst **5** (entry SI-9), a higher yield was achieved while maintaining a good mass balance.

Entry	Substrate	Cat. (mol%)	H_2O_2 addition, equiv. / min	Solvent	Carboxylic acid (equiv.)	Conversion / total yield (C4-yield), %	C4-selectivity, %
SI-7[c]	**S19**	**5** (1+1)	1+1/15+15	TFE	diMe-PA (1)	60/41 (25)	61
SI-8		**5** (1)	1/45			42/32 (19)	57
SI-9[d]		**5** (1+1)	1+1/45+45			67/52 (35)	67

[c]Additional portion of catalyst (1 mol%) and H_2O_2 (0.9 M, 20.56 µL, 1.37 µL/min) were added after 30 minutes. [d]Additional portion of catalyst (1 mol%) and H_2O_2 (0.9 M, 20.56 µL, 0.46 µL/min) were added after 60 minutes; overall reaction time is 120 minutes.

Control experiments: Oxidation reactions were performed in TFE (200 µL) using 2,2-dimethylpropanoic acid (1.89 mg, 18.5 µmol) under otherwise identical conditions, but without the second addition of catalyst **5** and H_2O_2.

To justify the synthesis of catalyst **5**, a series of control experiments was conducted (Figure 2). **Mn(mcp)** was selected as a reference catalyst due to its close structural similarity to the catalytic core of catalyst **5**. Oxidation reaction catalyzed by **Mn(mcp)** resulted in an almost statistical distribution of ketone products, with octan-2-one being the main product (Figure 2a).

The addition of calix[4]arene **6** (1 mol%) (Figure 2b), as well as the use of the less preorganized catalyst **4** (Figure 2c), did not result in any measurable change in ketone distribution relative to **Mn(mcp)**. This observation suggests that the binding capability of the calix[4]arene unit alone is insufficient to influence product selectivity. We therefore propose that effective substrate recognition and selectivity enhancement require a more rigid and preorganized catalytic environment, as realized in catalyst **5** (Figure 2d).

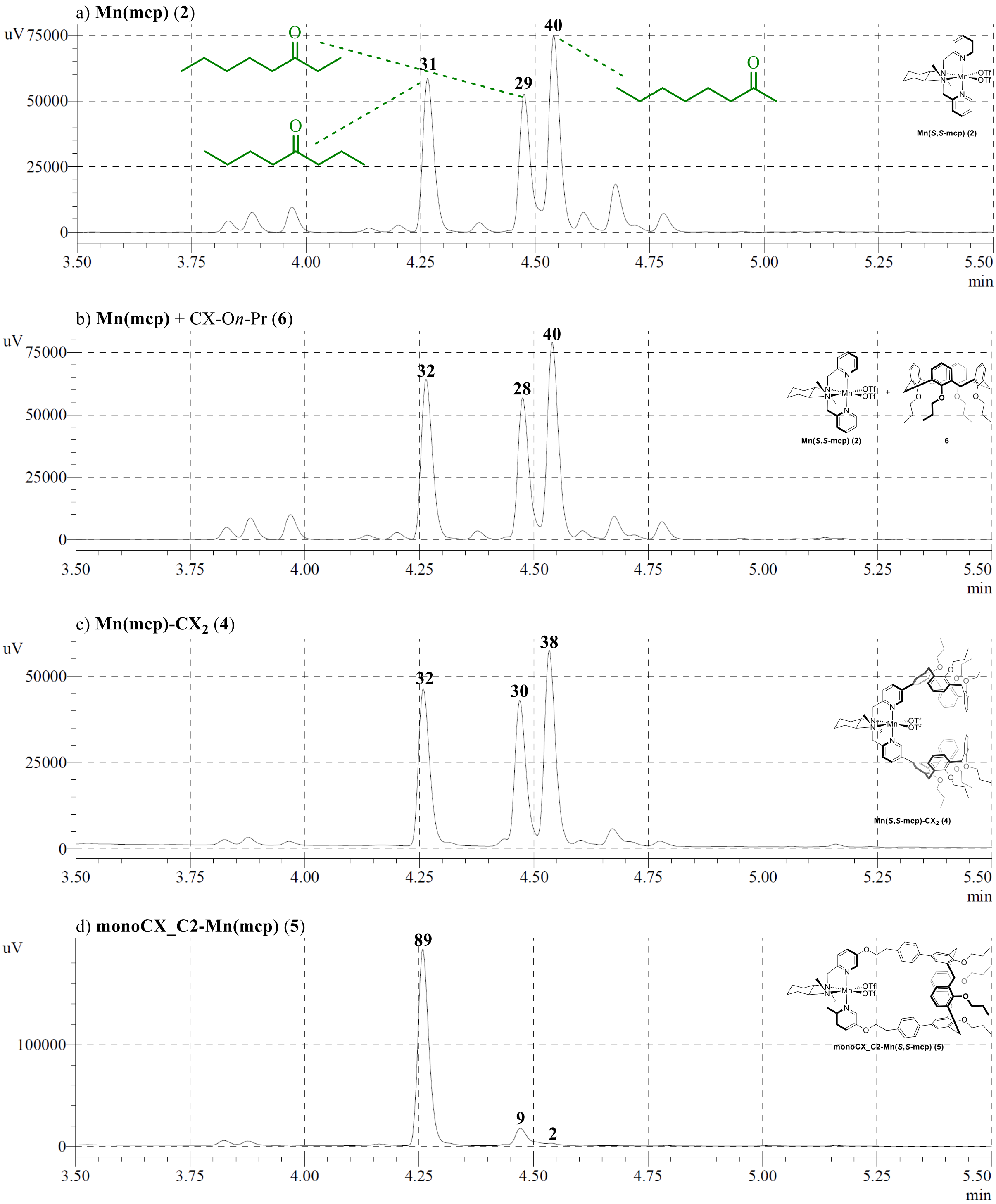

Figure 2. Control experiments: a) reference catalyst **Mn(mcp)**; b) reference catalyst **Mn(mcp)** with addition of calix[4]arene **6** (1 mol%); c) catalyst **4** with two calix[4]arenes attached to the **Mn(mcp)** core; d) investigated catalyst **5**. Selectivities (in %) are provided on chromatograms.

3.3 Octane oxidation products distribution

For the further used reaction conditions (section 3.2, entry 15) composition of the reaction mixtures was checked directly after oxidation with catalysts Mn(mcp) and **5** with gas chromatography. In both cases alcohols were the main products with some addition of ketones. Based on analysis from our previous paper[3], traces of epoxides and esters were also found. Besides that, minor additional unidentified compounds are observed on GC analysis.

The following temperature-program for GC was used: Rtx-5; 60 °C for 3 min, 15 °C/min to 250 °C and 250 °C for 5 min.

GC analysis of the **Mn(mcp)** catalyzed oxidation of octane before IBX:

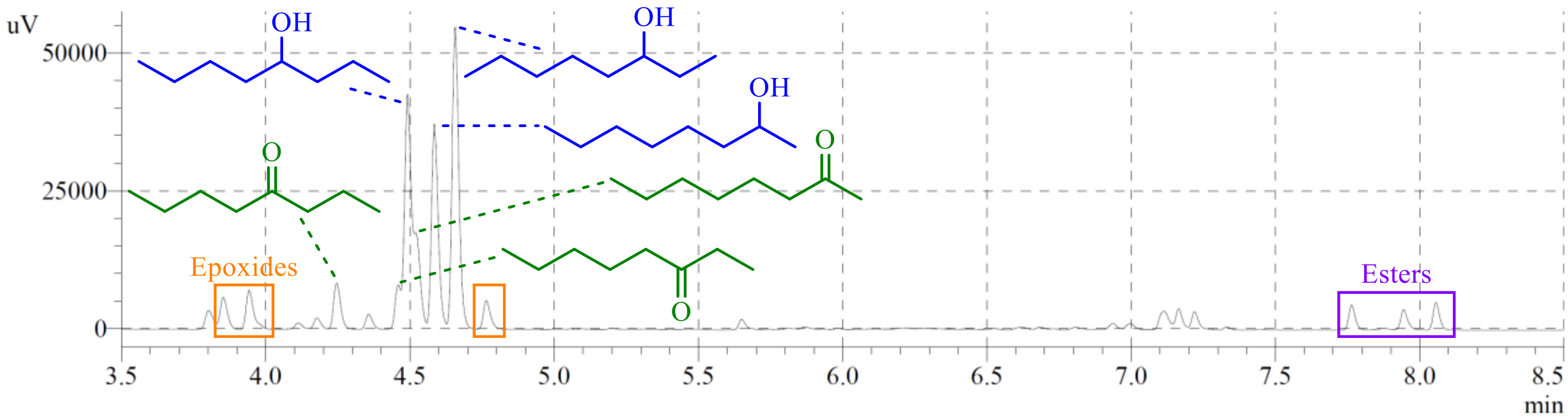

Retention time, min	Yield, %	Retention time, min	Yield, %
1.741 (substrate)	20.8	3.853	1.6
4.246	2.2	3.942	2.0
4.460	1.5	4.764	1.3
4.518	4.1	7.764	0.6
4.492	10.8	7.944	0.6
4.585	9.3	8.056	0.7
4.656	13.6		
Conversion: 79.2 %		Total product yield: 48.3 %	

GC analysis of the **5**-catalyzed oxidation of octane before IBX:

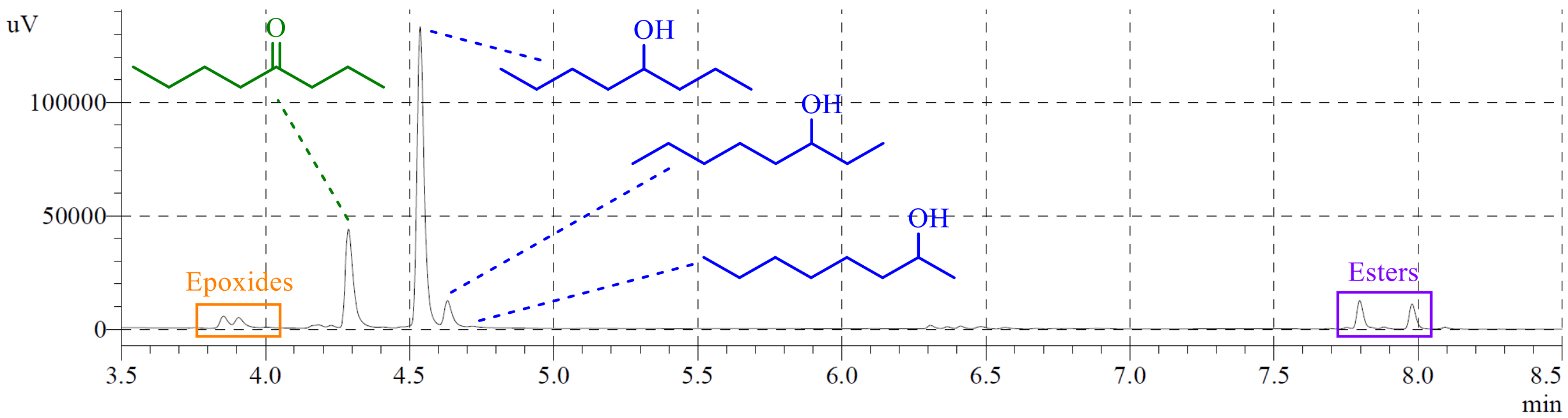

Retention time, min	Yield, %	Retention time, min	Yield, %
1.786 (substrate)	1.8	3.854	1.8
4.288	16.3	3.907	1.9
4.537	43.7	4.803	0.1
4.632	4.7	7.797	2.1
4.705	0.5	7.979	1.9
Conversion: 98.2 %		Total product yield: 73.0 %	

For the main target product, octan-4-ol, chiral GC analysis was performed in order to identify the enantiomeric ratio of the product. The analysis showed, that catalyst **5** provides *ee* = 39 %, while reference catalyst **Mn(mcp)** gives racemic mixture.

The following temperature-program for GC was used: MEGA-DEX DAC β; start at 50 °C, 0.50 °C/min to 75 °C, 25 °C/min to 220 °C and 220 °C for 2 min.

Chiral GC analyses of the **Mn(mcp)** (top) and **5**-catalyzed oxidation of octane before IBX (middle) with reference spectrum (bottom) of 4-octanol racemic mixture:

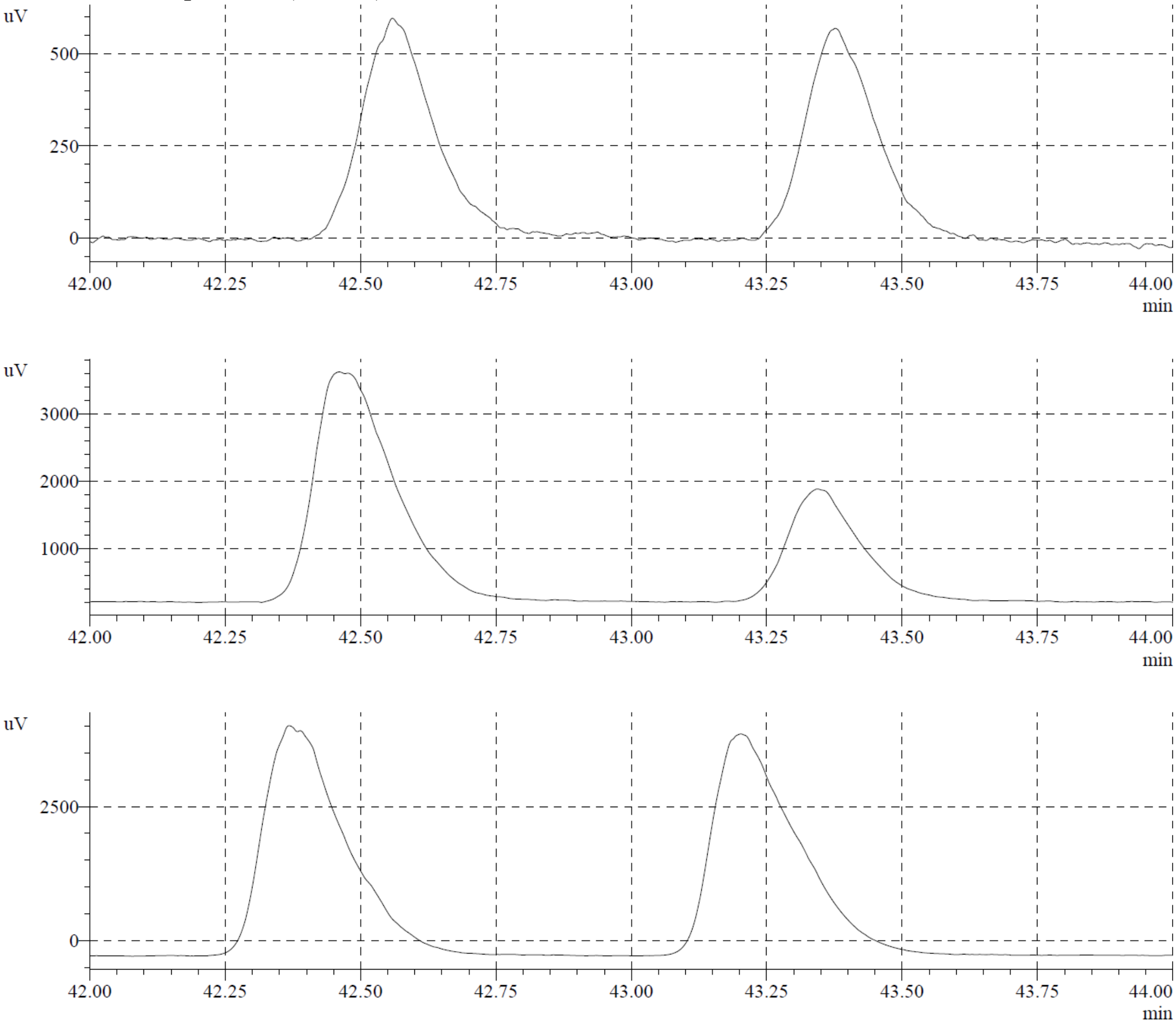

**Mn(mcp)** (top)		Catalyst **5** (middle)		Racemic mixture (bottom)	
Retention time, min	Area	Retention time, min	Area	Retention time, min	Area
42.559	5384	42.460	35307	42.369	44604
43.377	5250	43.344	15612	43.202	44674
ee < 5 %		*ee* = 39 %		*ee* < 5 %	

For better quantification, the reaction mixture was subjected to IBX oxidation in order to convert all alcohols into ketones. After that, the reaction mixture was analyzed by GC.

The following temperature-program for GC was used: Rtx-5; 60 °C for 3 min, 15 °C/min to 250 °C and 250 °C for 5 min.

GC analysis of the **Mn(mcp)** catalyzed oxidation of octane after IBX:

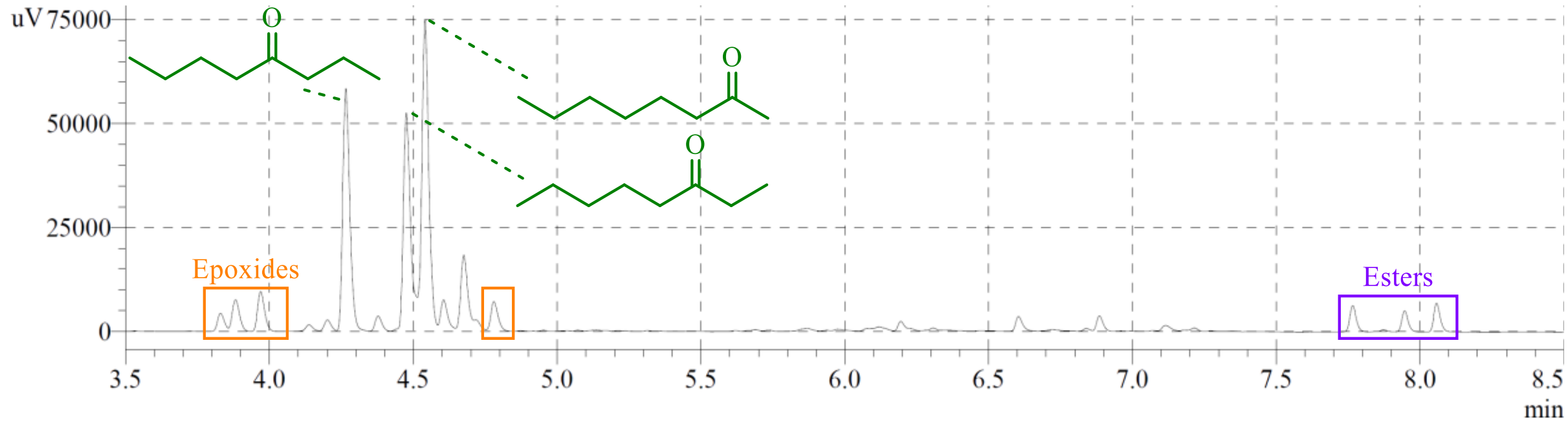

Retention time, min	Yield, %	Retention time, min	Yield, %
1.783 (substrate)	17.4	3.969	1.7
4.265	11.0	4.780	1.2
4.476	10.3	7.766	0.6
4.540	13.9	7.946	0.5
3.882	1.3	8.058	0.7
Conversion: 82.6 %		Total product yield: 41.2 %	

GC analysis of the **5**-catalyzed oxidation of octane after IBX:

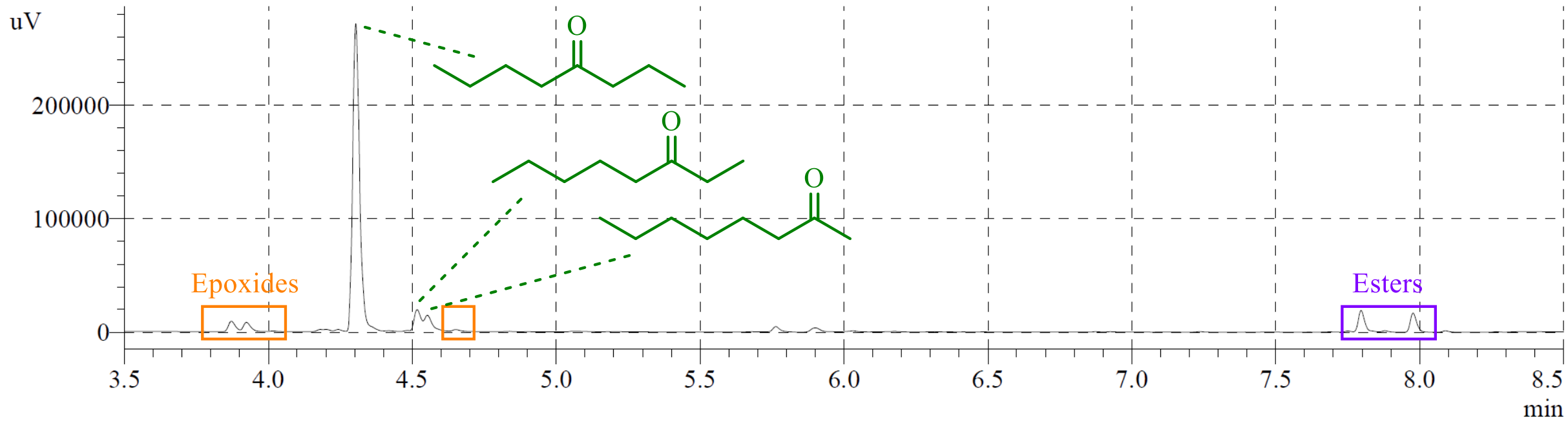

Retention time, min	Yield, %	Retention time, min	Yield, %
1.821 (substrate)	2.1	3.924	1.7
4.304	59.3	4.830	0.1
4.508	4.1	7.797	2.0
4.553	3.3	7.977	2.0
3.872	1.8		
Conversion: 97.9 %		Total product yield: 74.3 %	

As the total yield of epoxides and esters is less than 10% points for both **Mn(mcp)** and catalyst **5**, these oxidation products will not be taken into account in further calculations.

3.4 Catalyst's activity and stability under oxidation conditions

Activity test – kinetic profile of the reaction

4 oxidation reactions were set up in parallel according to the following procedure.
Procedure: TFE (200 µL), octane (2.98 µL, 2.11 mg, 18.5 µmol), 2,2-dimethylpropanoic acid (1.89 mg, 18.5 µmol) and 20 µL of TfOH solution in TFE (2.5 mol% of TfOH in relation to octane) were added to a 2 mL vial charged with catalyst **5** (0.279 mg, 0.185 µmol) and a stirring bar. The mixture was cooled to 0 °C. A solution of an 50% aqueous H_2O_2 diluted in TFE (20.6 µL; stock solution: 0.1 mL 50 % aqueous H_2O_2 in 1.9 mL TFE) was slowly added over 15 minutes (1.37 µL/min) under stirring via a syringe pump.

After 7.5, 15, 30, 60 min one of the reactions was stopped by addition of H_2O (1.5 mL) and 0.0925 M biphenyl solution in MeCN (40.0 µL) and the mixture was extracted with HPLC grade EtOAc (1.0 mL × 2), washed with sat. $NaHCO_3$ (2.0 mL), dried over Na_2SO_4, filtered through a silica plug and rinsed with EtOAc.

0.5 mL of the mixture was transferred into another 2 mL vial equipped with a stirring bar. IBX (3.50 mg, 12.5 µmol) was added and the mixture was heated to 66 °C overnight. The mixture was allowed to cool down to room temperature, filtered through a silica plug and rinsed with EtOAc (1.0 mL × 2). GC-samples were prepared in hexane and analyzed by GC.

The 4 obtained organic fractions were analyzed by GC to check the kinetic profile of the reaction (Fig. 3). The following temperature-program for GC was used: Rtx-5; 60 °C for 3 min, 15 °C/min to 250 °C and 250 °C for 5 min.

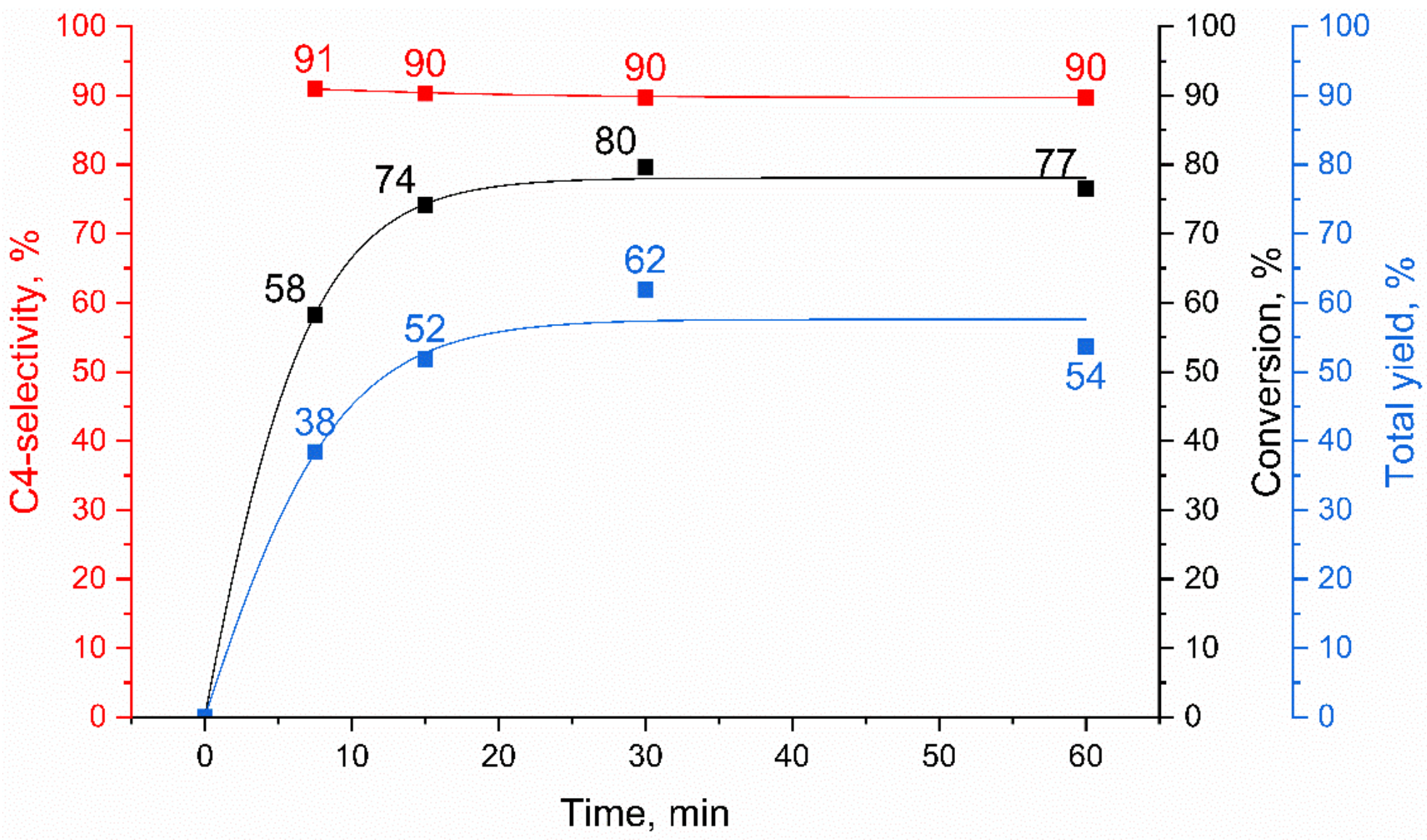

Figure 3. Kinetic profile of the reaction analyzed by GC.

The data obtained suggests the oxidation reaction happens at the rate of H_2O_2 addition, as no significant increase of conversion and yield is observed after 15 minutes (after time of H_2O_2 addition). The C4-selectivity is maintained through the whole oxidation run within confidence interval (see table below).

The mean value and standard deviation at the 95% confidence level were determined from six independent experiments performed under identical conditions (procedure above; 60 minutes). To evaluate repeatability, three different batches of the catalyst were used.

Experiment	Conversion, %	Yield, %	C4-selectivity, %
1	72.7	51.9	87.39
2	73.3	52.5	90.65
3	75.2	47.9	89.16
4	77.8	50.8	90.97
5	77.2	50.0	91.53
6	94.6	62.6	91.29

Mean value	78.5	52.6	90.16
Standard deviation	6.0	3.8	1.17
	78±6	53±4	90±1

Stability test – analysis of the catalyst's self-oxidation

Procedure: TFE (600 µL), octane (8.95 µL, 55.5 µmol) and 2,2-dimethylbutanoic acid (6.95 µL, 55.5 µmol) were added to a vial charged with catalyst **5** (0.837 mg, 0.555 µmol) and a stirring bar. The mixture was cooled to 0 °C. A solution of a 50% aqueous H_2O_2 diluted in TFE (61.7 µL; stock solution: 0.1 mL 50 % aqueous H_2O_2 in 1.9 mL TFE) was slowly added (4.11 µL/min) under stirring to the reaction mixture via a syringe pump. After 0, 7.5, 15, 30, 60 min samples (60 µL) were taken from the reaction mixture and put into ethyl acetate (1.0 mL). Decomplexation of the metal was performed in the following way. $NaHCO_3$ saturated water solution (2.0 mL) was added and the mixture was extracted with HPLC grade EtOAc (1 mL × 2), dried over Na_2SO_4. The obtained organic fraction were analyzed by HRMS to check the ligand oxidation (Fig. 4). Quantification of the mixture composition was done based on extracted ion chromatograms (EIC) of each compound.

The analysis shows presence of several oxidized species. We assume benzylic positions, being susceptible to oxidation, are unstable under the reaction conditions and contribute to catalyst decomposition. Nevertheless, the catalyst retains structural integrity long enough to sustain catalytic activity. Furthermore, it appears that the initial, non-oxidized form of catalyst **5** governs the oxidation selectivity, as the high regioselectivity observed at the onset of the reaction is consistently maintained throughout.

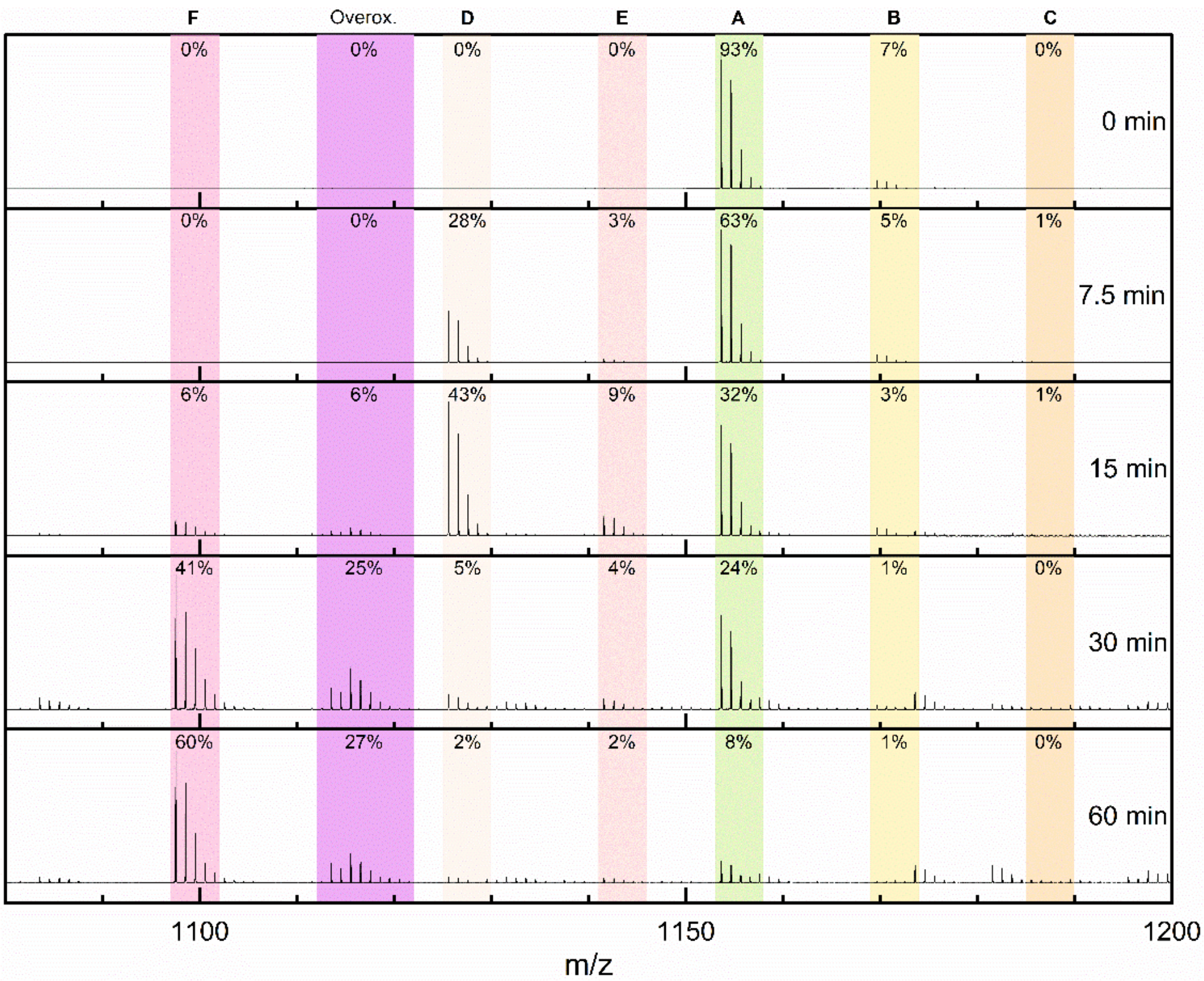

Figure 4. HRMS analyses of reaction mixture samples at different reaction times.

The following isotopic patterns were obtained and calculated for the sample after 15 min of H_2O_2 addition.

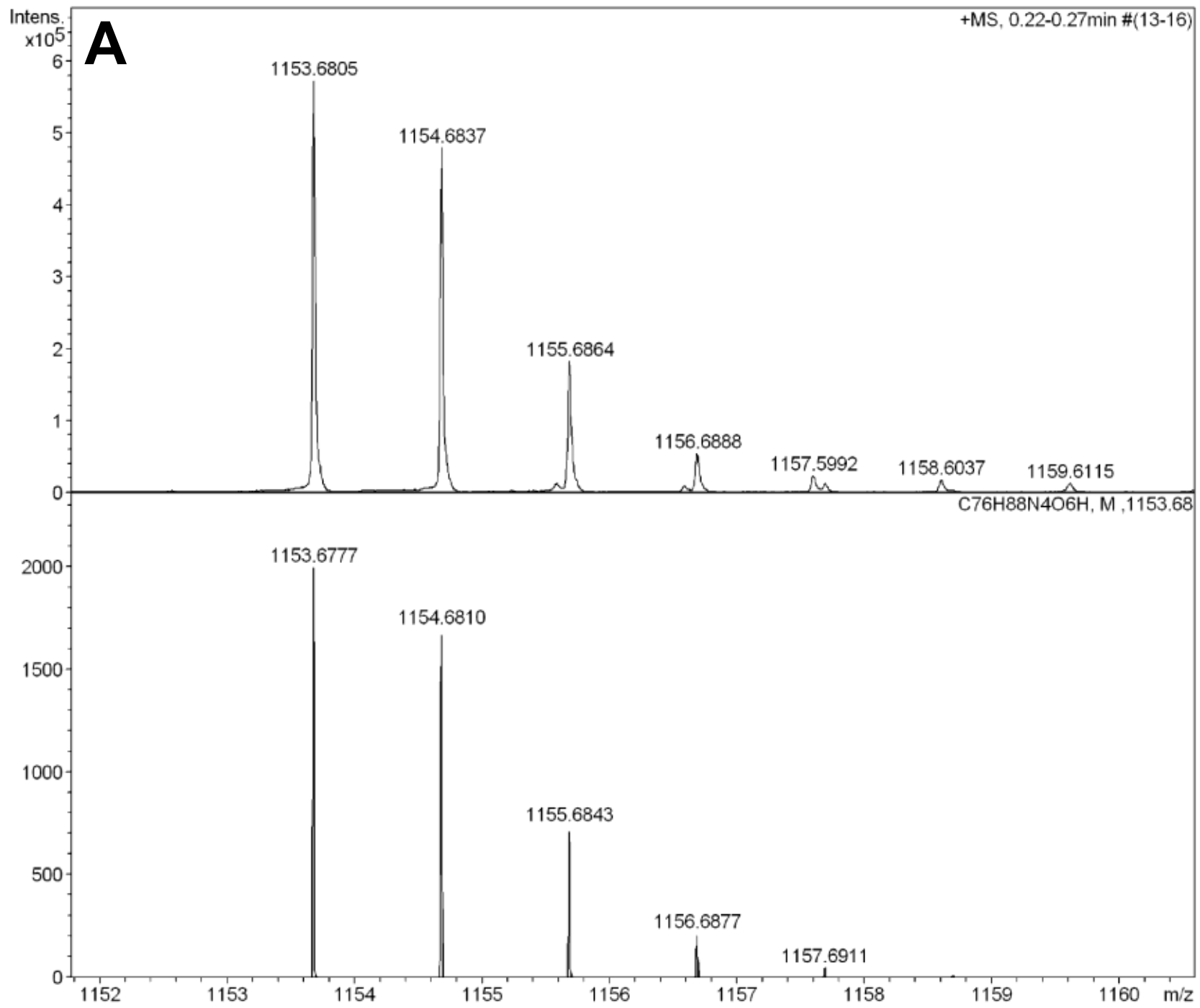

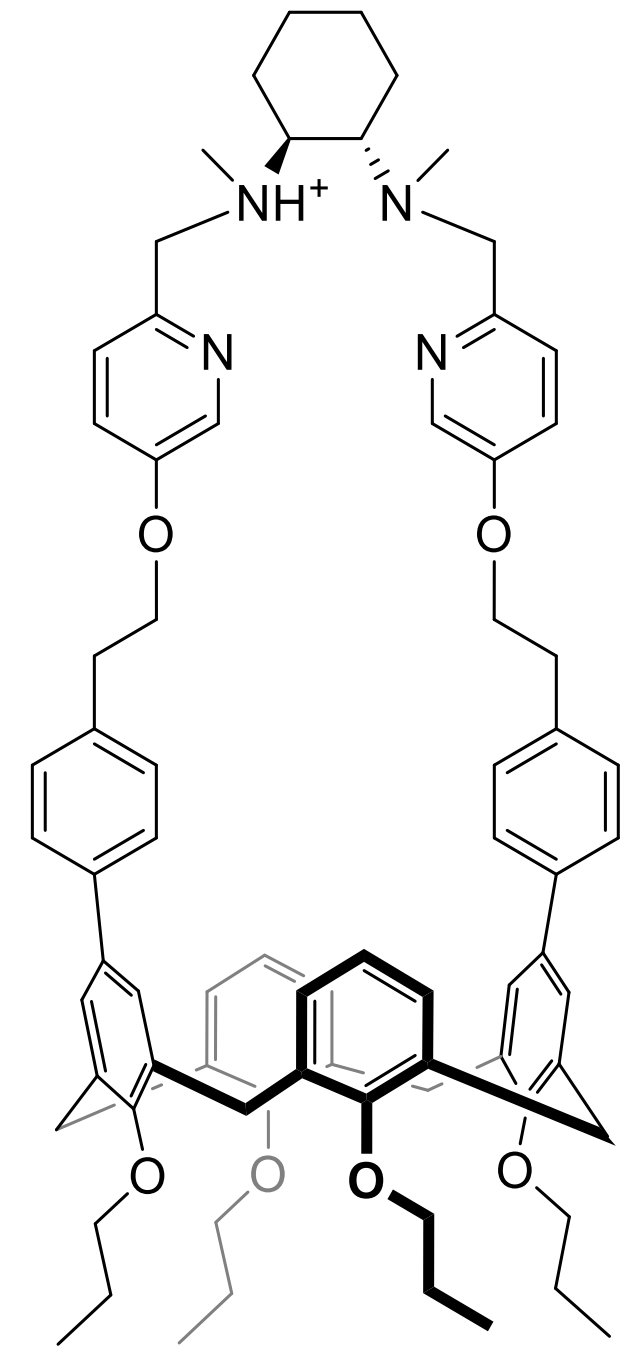

Chemical Formula: $C_{76}H_{89}N_4O_6^+$
Exact Mass: 1153.6777

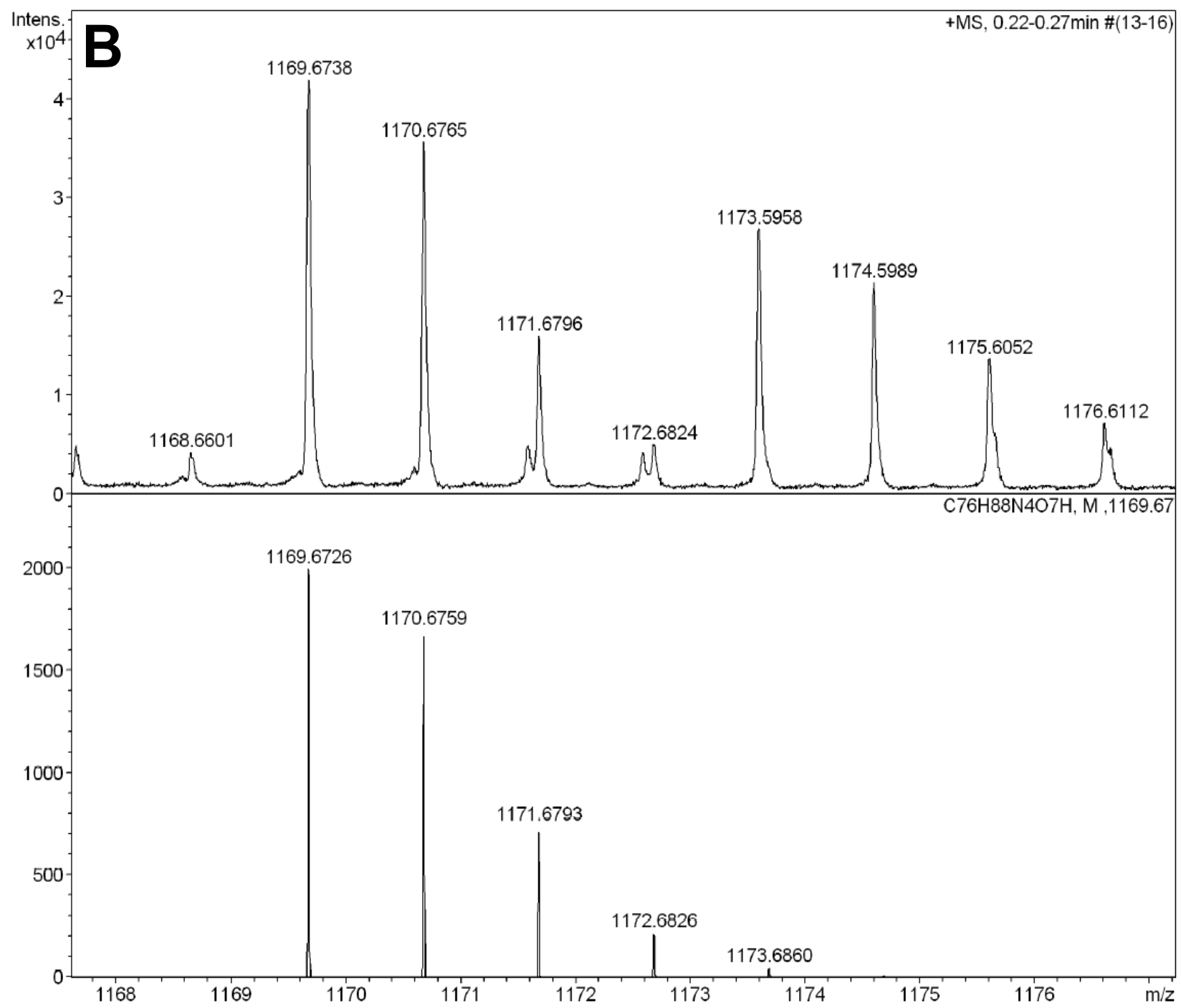

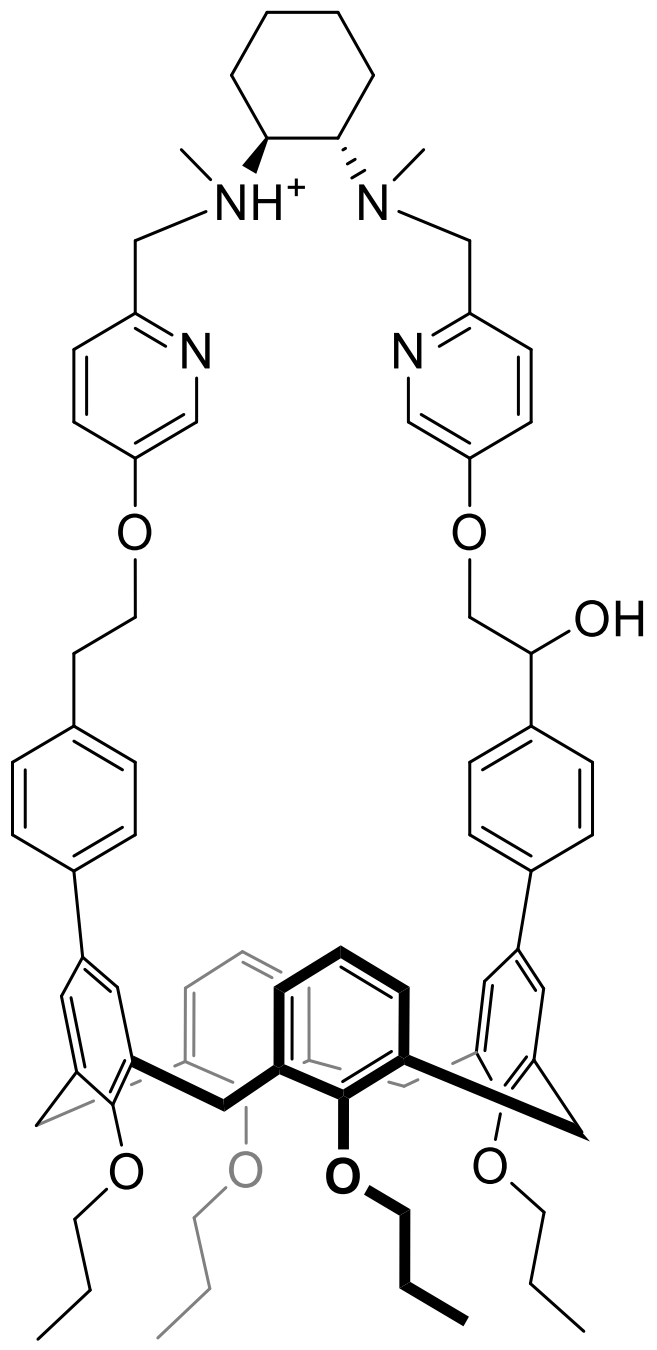

Chemical Formula: $C_{76}H_{89}N_4O_7^+$
Exact Mass: 1169.67

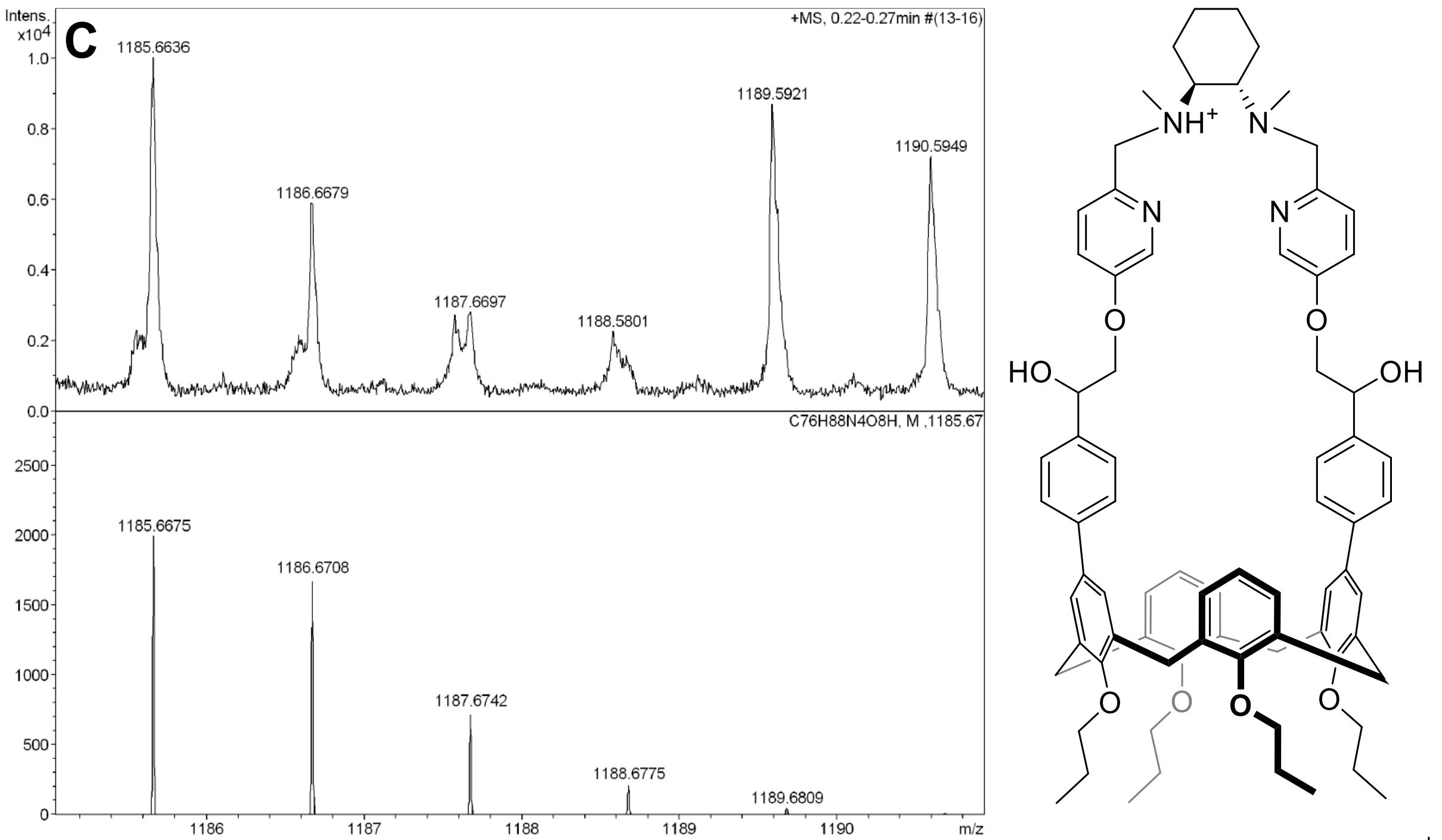

Chemical Formula: $C_{76}H_{89}N_4O_8^+$
Exact Mass: 1185.67

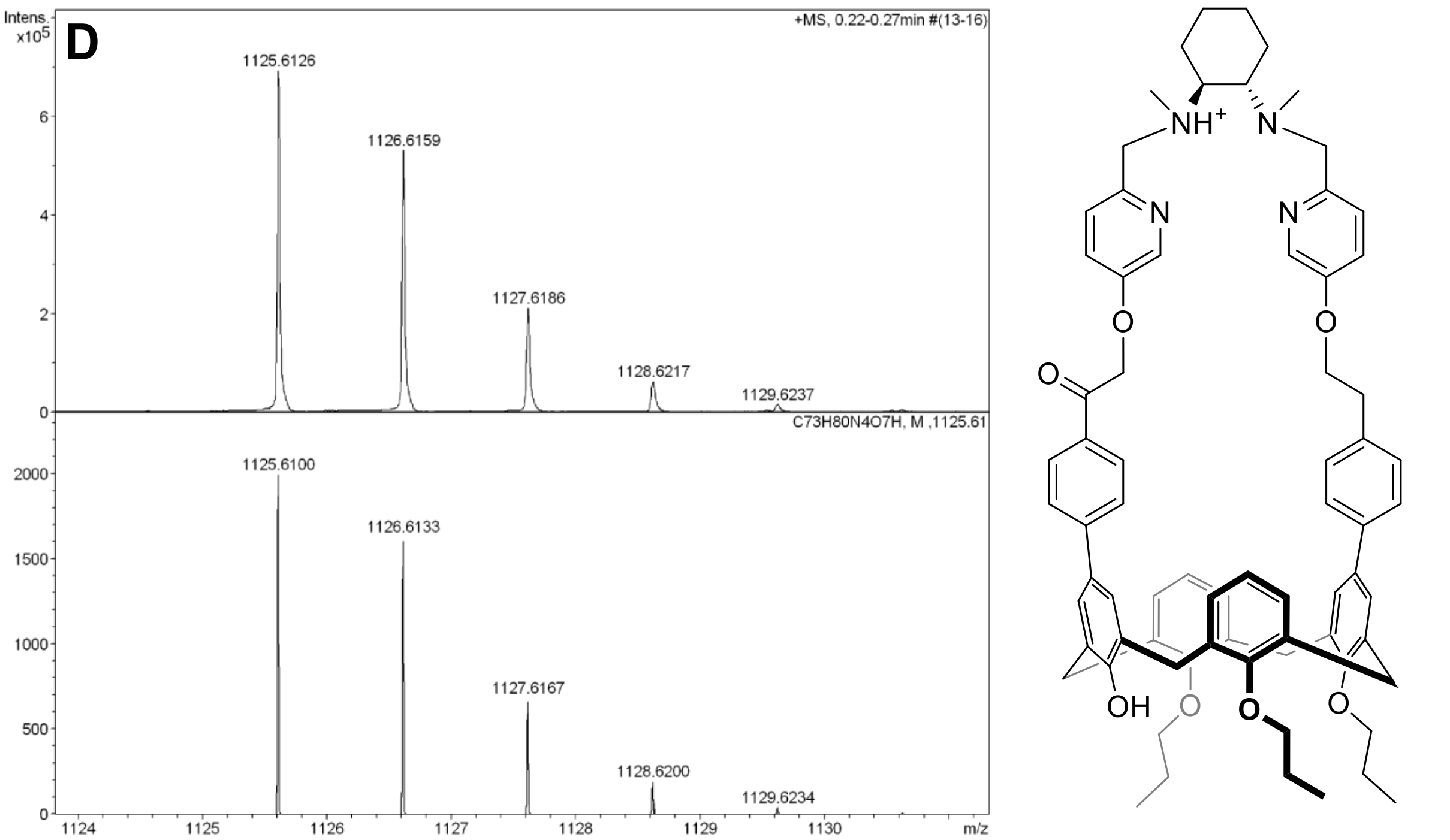

Chemical Formula: $C_{73}H_{81}N_4O_7^+$
Exact Mass: 1125.6100

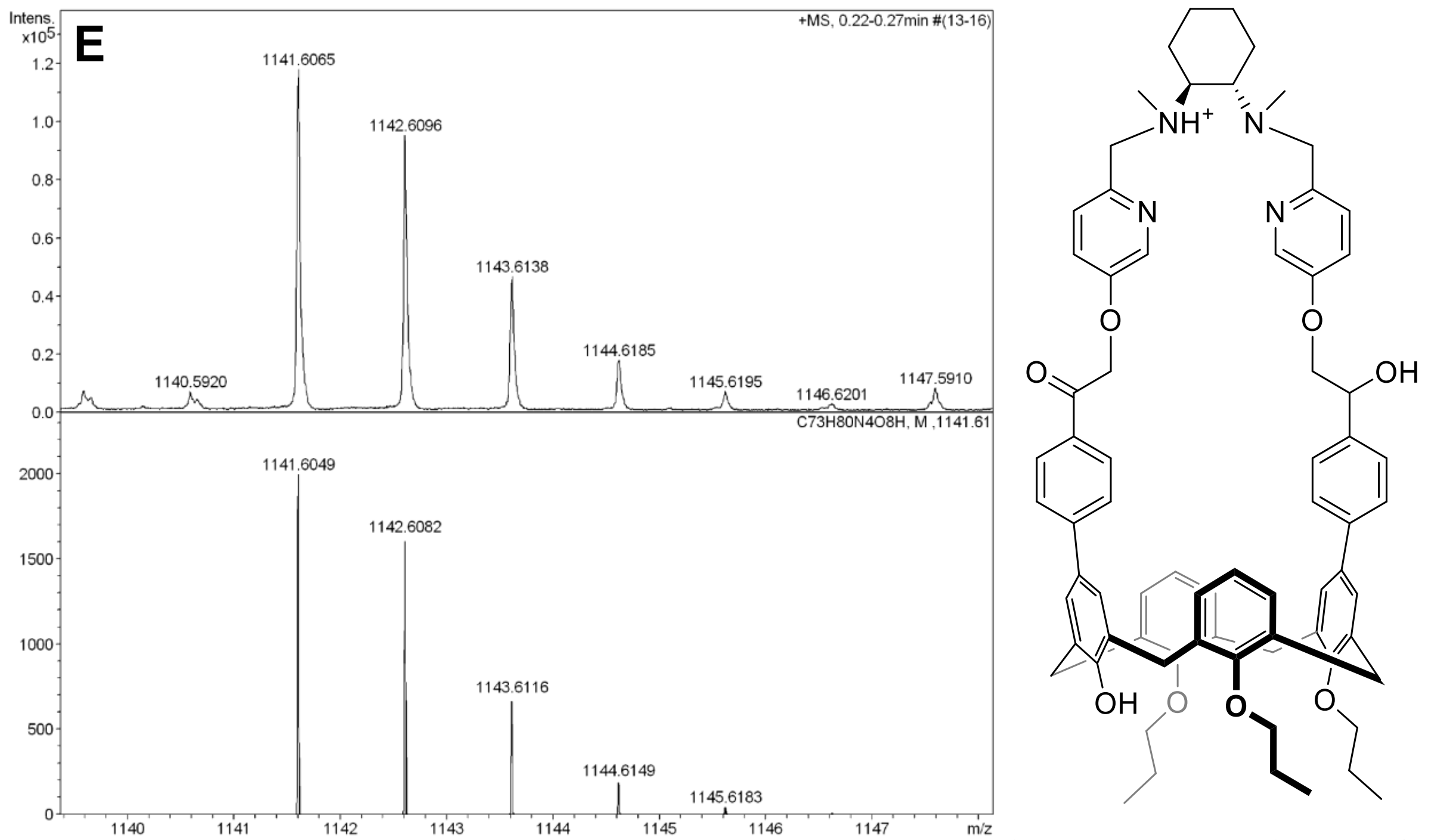

Chemical Formula: $C_{73}H_{81}N_4O_8^+$
Exact Mass: 1141.6049

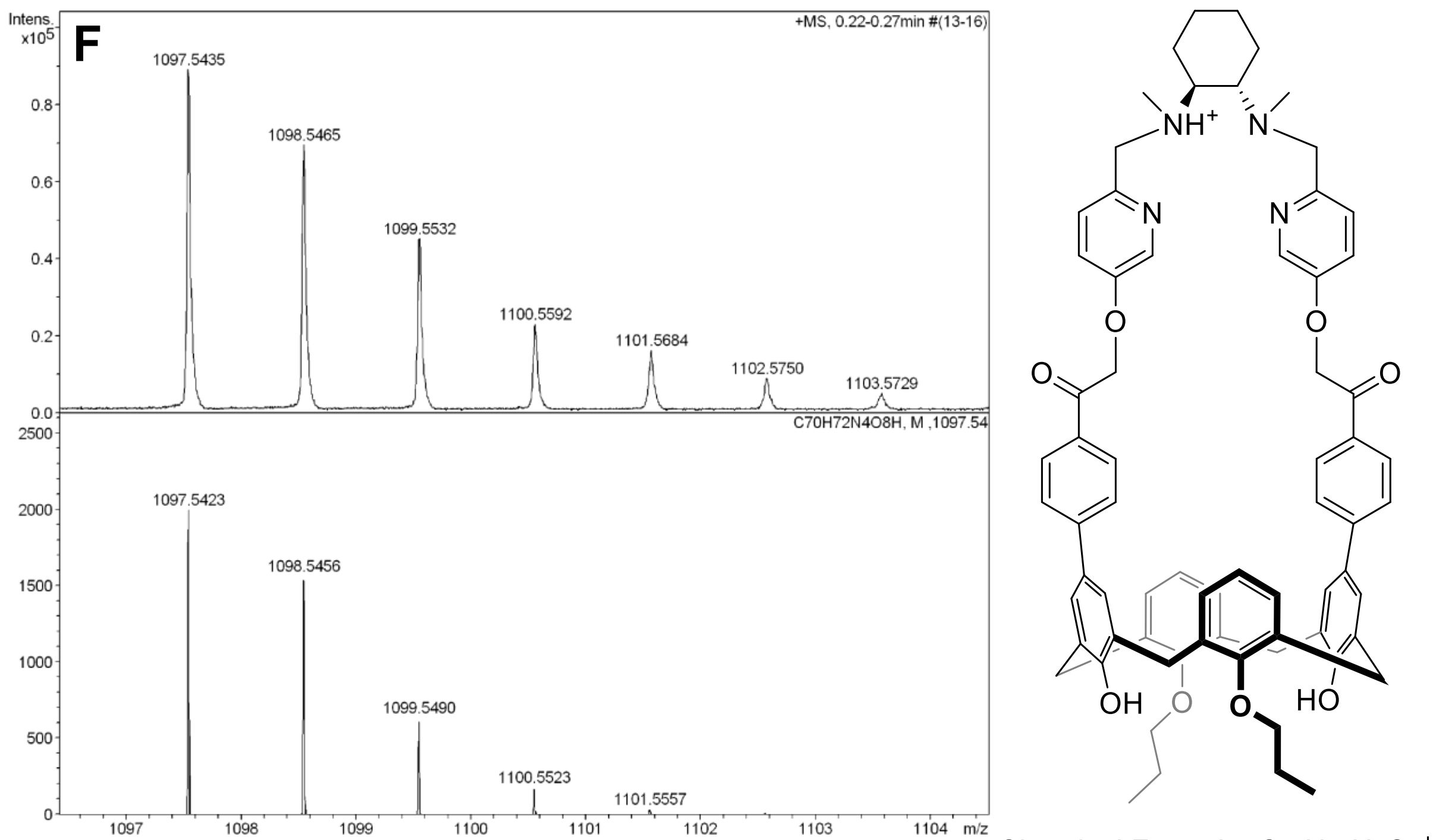

Chemical Formula: $C_{70}H_{73}N_4O_8^+$
Exact Mass: 1097.5423

Overoxidized compounds are structurally unidentified, but their composition can be considered as the following: $[\mathbf{F} + O]^+$ (1113.5378) and $[\mathbf{F} + O + 2H]^+$ (1115.5534).

4. Substrate Scope and Detailed Oxidation Results

4.1 General procedure for oxidation reaction.

Oxidation with Mn(mcp): TFE (200 µL), substrate (18.5 µmol) and 2,2-dimethylpropanoic acid (1.89 mg, 18.5 µmol) were added to a 2 mL vial charged with Mn(mcp) (0.125 mg, 0.185 µmol) and a stirring bar. The mixture was cooled to 0 °C. A solution of an 50% aqueous H_2O_2 diluted in TFE (20.6 µL; stock solution: 0.1 mL 50 % aqueous H_2O_2 in 1.9 mL TFE) was slowly added over 15 minutes (1.37 µL/min) under stirring via a syringe pump. After the addition, the mixture was left stirring for another 45 min.

Oxidation with catalyst 5: TFE (200 µL), substrate (18.5 µmol), 2,2-dimethylpropanoic acid (1.89 mg, 18.5 µmol) and 20 µL of TfOH solution in TFE (2.5 mol% of TfOH in relation to substrate) were added to a 2 mL vial charged with catalyst **5** (0.279 mg, 0.185 µmol) and a stirring bar. The mixture was cooled to 0 °C. A solution of an 50% aqueous H_2O_2 diluted in TFE (20.6 µL; stock solution: 0.1 mL 50 % aqueous H_2O_2 in 1.9 mL TFE) was slowly added over 15 minutes (1.37 µL/min) under stirring via a syringe pump, After the addition, the mixture was left stirring for another 15 min.

After that time the second oxidation cycle was done: another portion of catalyst **5** (0.279 mg, 0.185 µmol) in 50 µL TFE was added. Then another portion of the solution of an 50% aqueous H_2O_2 in TFE (20.6 µL) was slowly added over 15 minutes (1.37 µL/min) under stirring via a syringe pump. After the addition, the mixture was left stirring for another 15 min.

Work-up and GC analysis: after the time indicated, H_2O (1.5 mL) and 0.0925 M biphenyl solution in MeCN (40.0 µL) were added and the mixture was extracted with HPLC grade EtOAc (1.0 mL × 2), washed with sat. $NaHCO_3$ (2.0 mL), dried over Na_2SO_4, filtered through a silica plug and rinsed with EtOAc.

IBX Oxidation: 0.5 mL of the mixture was transferred into another 2 mL vial equipped with a stirring bar. IBX (3.50 mg, 12.5 µmol) was added and the mixture was heated to 66 °C overnight. The mixture was allowed to cool down to room temperature, filtered through a silica plug and rinsed with EtOAc (1.0 mL × 2). GC-samples were prepared in hexane and analyzed by GC.

Calculations

The relative response factors (RRF) of substrates and oxidation products were calculated according to the literature.[1,2] "Integral" turnover number (TON) was obtained by dividing the total yield by the percentage of the catalyst **5** loading. "Integral" turnover frequency (TOF) was calculated assuming complete reaction after 15 minutes. Conversion, total product yield and C_i-selectivity were calculated according to the following equations:

$$\mathrm{P}_i, \% = \frac{\mathrm{A}_i}{\mathrm{A}_{biphenyl}} \times 100\%;$$

$$\mathrm{P}_i^{\mathrm{RRF}}, \% = \mathrm{C}_i - \mathrm{Yield}, \% = \frac{\mathrm{P}_i}{\mathrm{RRF}_i} \times 100\%$$

$$\mathrm{Conversion}, \% = 100\% - \frac{\mathrm{A}_{substrate}}{\mathrm{A}_{biphenyl}} \times 100\%;$$

$$\text{Total product yield}, \% = \sum_i \mathrm{P}_i^{\mathrm{RRF}} = \frac{\sum_i \frac{\mathrm{A}_i}{\mathrm{RRF}_i}}{\mathrm{A}_{biphenyl}} \times 100\%;$$

$$\mathrm{C}_i - \mathrm{Selectivity}, \% = \frac{\mathrm{P}_i^{\mathrm{RRF}}}{\sum_i \mathrm{P}_i^{\mathrm{RRF}}} = \frac{\frac{\mathrm{A}_i}{\mathrm{RRF}_i}}{\sum_i \frac{\mathrm{A}_i}{\mathrm{RRF}_i}} \times 100\%.$$

Heptane (S1) oxidation

The oxidation was performed using heptane (2.71 µL, 1.85 mg).
The following temperature-program was used: Rtx-5; 60 °C for 3 min, 15 °C/min to 250 °C and 250 °C for 5 min.

GC analysis of the **Mn(mcp)** catalyzed oxidation of heptane:

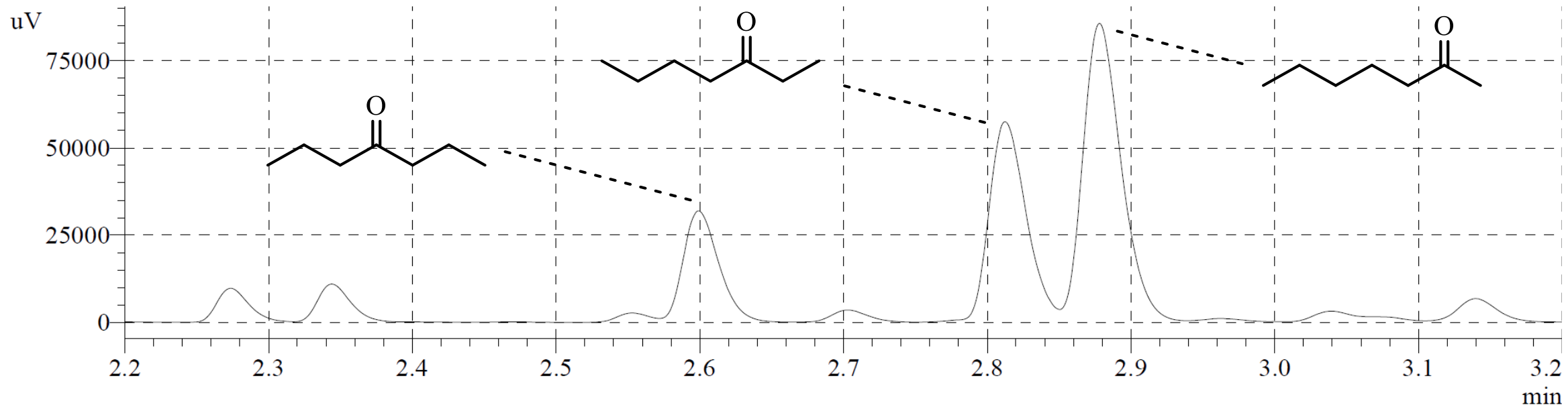

RRF(substrate): 0.583 RRF(ketone): 0.500

i	Retention time, min	Area (A)	Percentage (P), %	Percentage with RRF (P^{RRF}), %	Selectivity, %
-	1.229 (substrate)	88525	5.9779	10.2	
4	2.599	50998	3.4438	6.9	16.4
3	2.813	102786	6.9409	13.9	33.2
2	2.878	156308	10.555	21.1	50.4
	9.031 (biphenyl)	296173	TON = 42; TOF = $4.7\cdot10^{-2}$ s^{-1}		
	Conversion: 89.8		Total product yield: 41.9		

GC analysis of the **5**-catalyzed oxidation of heptane:

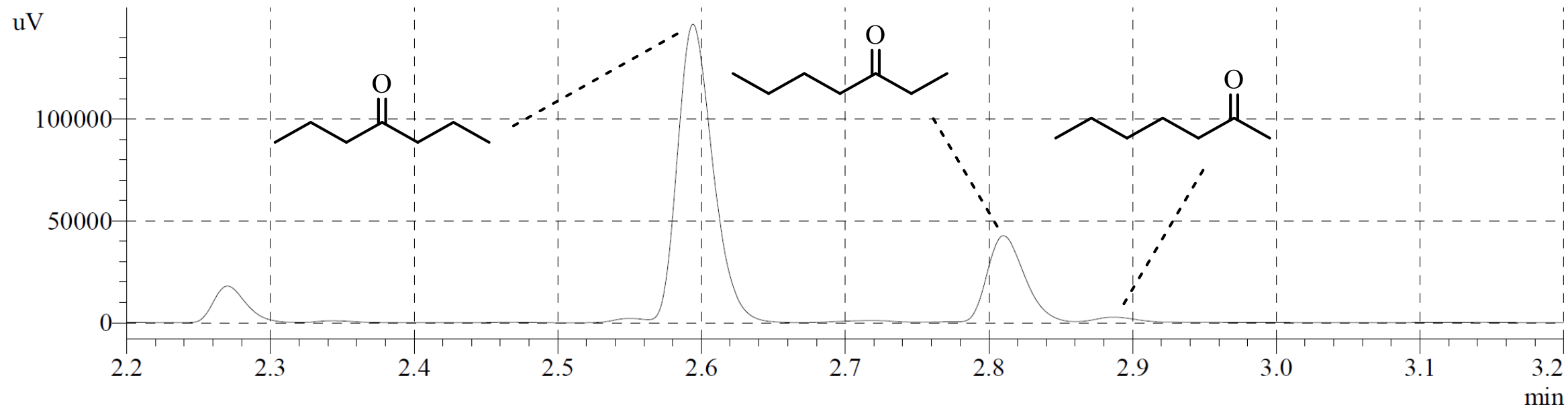

RRF(substrate): 0.583 RRF(ketone): 0.500

i	Retention time, min	Area (A)	Percentage (P), %	Percentage with RRF (P^{RRF}), %	Selectivity, %
-	1.287 (substrate)	80094	4.5454	7.8	
4	2.594	253217	14.370	28.7	76.0
3	2.810	75652	4.2933	8.6	22.7
2	2.887	4428	0.25129	0.5	1.3
	9.030 (biphenyl)	352418	TON = 19; TOF = $2.1\cdot10^{-2}$ s^{-1}		
	Conversion: 92.2		Total product yield: 37.8		

The different oxidation products were assigned according to literature data.[3]

Octane (S2) oxidation

The oxidation was performed using octane (2.98 µL, 2.11 mg).
The following temperature-program was used: Rtx-5; 60 °C for 3 min, 15 °C/min to 250 °C and 250 °C for 5 min.

GC analysis of the **Mn(mcp)** catalyzed oxidation of octane:

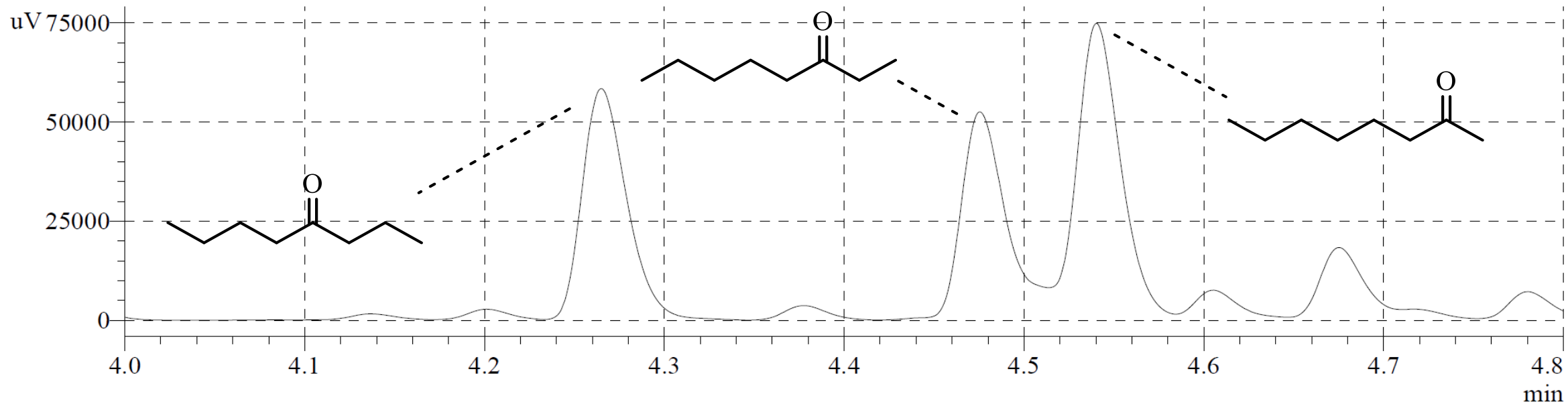

RRF(substrate): 0.667 RRF(ketone): 0.583

i	Retention time, min	Area (A)	Percentage (P), %	Percentage with RRF (P^{RRF}), %	Selectivity, %
-	1.783 (substrate)	180890	11.596	17.4	
4	4.265	99825	6.3991	11.0	31.3
3	4.476	93372	5.9854	10.3	29.2
2	4.54	126069	8.0814	13.8	39.5
	9.031 (biphenyl)	311996	TON = 35; TOF = $3.9 \cdot 10^{-2}$ s^{-1}		
	Conversion: 82.6		Total product yield: 35.1		

GC analysis of the **5**-catalyzed oxidation of octane:

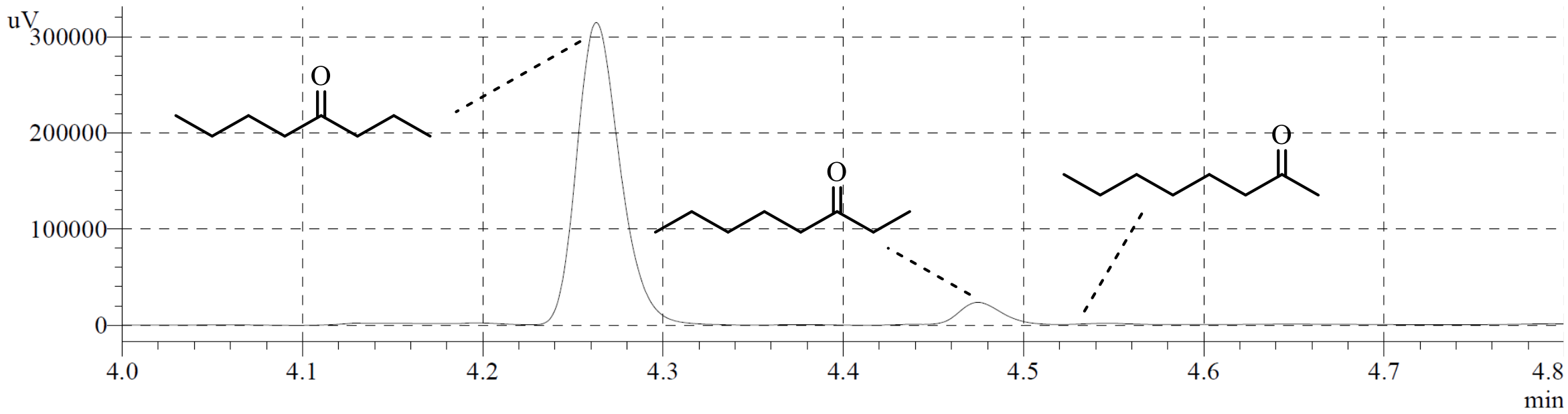

RRF(substrate): 0.667 RRF(ketone): 0.583

i	Retention time, min	Area (A)	Percentage (P), %	Percentage with RRF (P^{RRF}), %	Selectivity, %
-	1.821 (substrate)	17996	1.3709	2.1	
4	4.304	454017	34.586	59.3	88.8
3	4.508	31506	2.4000	4.1	6.2
2	4.553	25499	1.9425	3.3	5.0
	9.069 (biphenyl)	262543	TON = 33; TOF = $3.7 \cdot 10^{-2}$ s^{-1}		
	Conversion: 97.9		Total product yield: 66.7		

The different oxidation products were assigned according to literature data.[3]

Nonane (S3) oxidation

The oxidation was performed using nonane (3.30 μL, 2.37 mg).
The following temperature-program was used: MEGA-DEX DAC β; start at 50 °C, 5 °C/min to 120 °C, 120 °C for 6 min, 20 °C/min to 220 °C and 220 °C for 3 min.

GC analysis of the **Mn(mcp)** catalyzed oxidation of nonane:

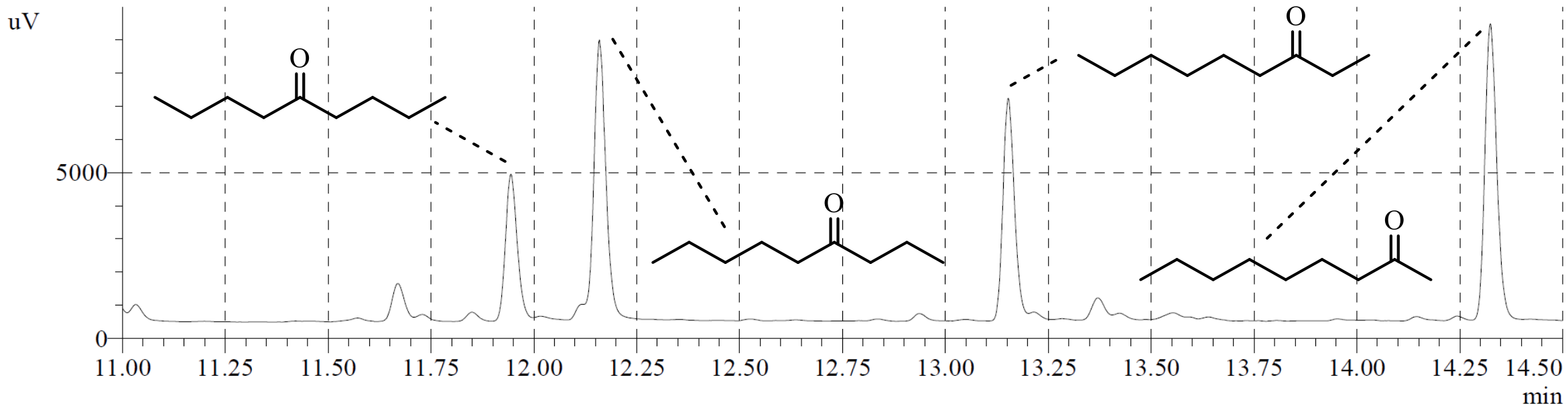

RRF(substrate): 0.750 RRF(ketone): 0.667

i	Retention time, min	Area (A)	Percentage (P), %	Percentage with RRF (P^{RRF}), %	Selectivity, %
-	5.173 (substrate)	58522	20.623	27.5	
5	11.944	8849	3.1184	4.7	15.2
4	12.159	17772	6.2628	9.4	30.6
3	13.153	13548	4.7743	7.2	23.3
2	14.325	17883	6.3019	9.4	30.8
	22.608 (biphenyl)	56754	TON = 31; TOF = $3.4\cdot10^{-2}$ s^{-1}		
	Conversion: 72.5		Total product yield: 30.7		

GC analysis of the **5**-catalyzed oxidation of nonane:

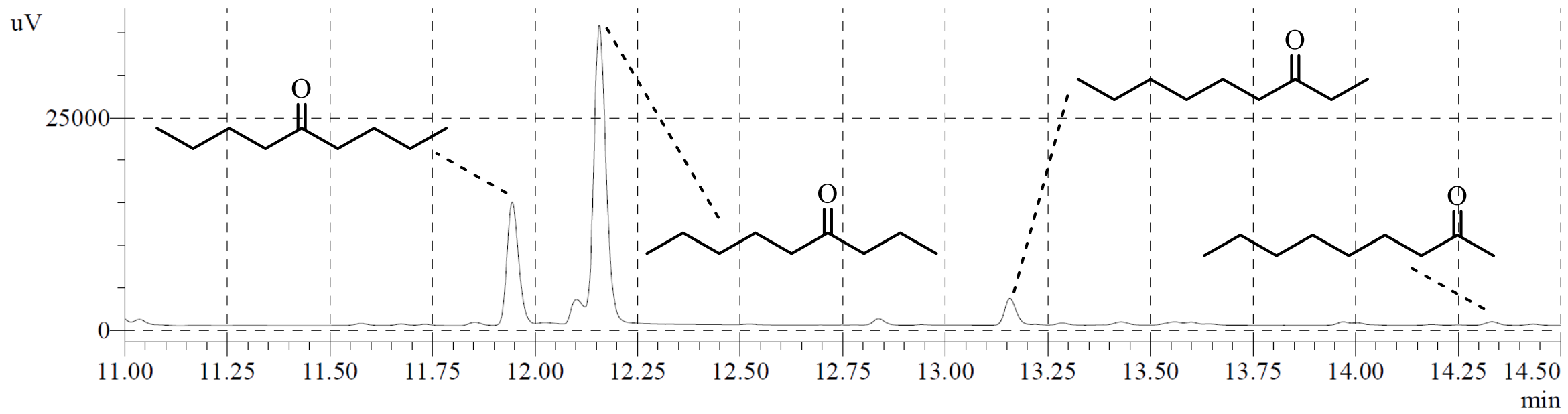

RRF(substrate): 0.750 RRF(ketone): 0.667

i	Retention time, min	Area (A)	Percentage (P), %	Percentage with RRF (P^{RRF}), %	Selectivity, %
-	5.167 (substrate)	4354	1.6409	2.2	
5	11.945	28850	10.873	16.3	26.2
4	12.157	73936	27.864	41.8	67.0
3	13.158	6362	2.3976	3.6	5.8
2	14.333	1107	0.41719	0.6	1.0
	22.611 (biphenyl)	53069	TON = 31; TOF = $3.5\cdot10^{-2}$ s^{-1}		
	Conversion: 97.8		Total product yield: 62.3		

The different oxidation products were assigned according to standard samples.[3]

Decane (S4) oxidation

The oxidation was performed using decane (3.61 µL, 2.63 mg).
The following temperature-program was used: MEGA-DEX DAC β; start at 50 °C, 5 °C/min to 120 °C, 120 °C for 6 min, 20 °C/min to 220 °C and 220 °C for 3 min.

GC analysis of the **Mn(mcp)** catalyzed oxidation of decane:

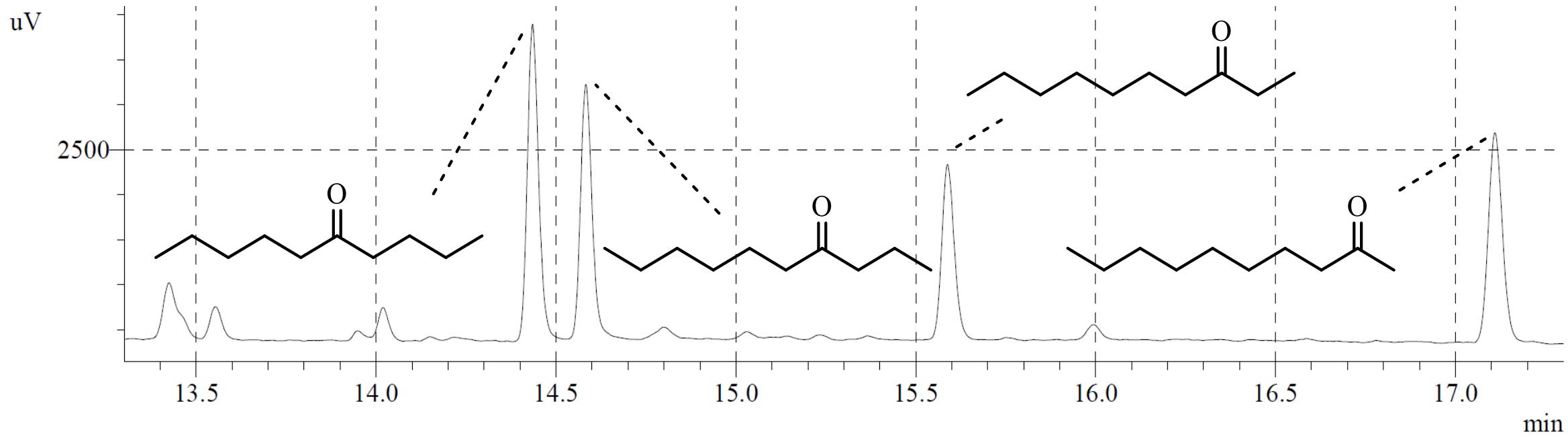

RRF(substrate): 0.833 RRF(ketone): 0.750

i	Retention time, min	Area (A)	Percentage (P), %	Percentage with RRF (P^{RRF}), %	Selectivity, %
-	7.319 (substrate)	111616	40.599	48.7	
5	14.435	7765	2.8244	3.8	30.6
4	14.583	6516	2.3701	3.2	25.7
3	15.589	4738	1.7234	2.3	18.7
2	17.11	6312	2.2959	3.1	24.9
	22.608 (biphenyl)	54985	TON = 12; TOF = $1.4\cdot10^{-2}$ s^{-1}		
	Conversion: 51.3		Total product yield: 12.4		

GC analysis of the **5**-catalyzed oxidation of decane:

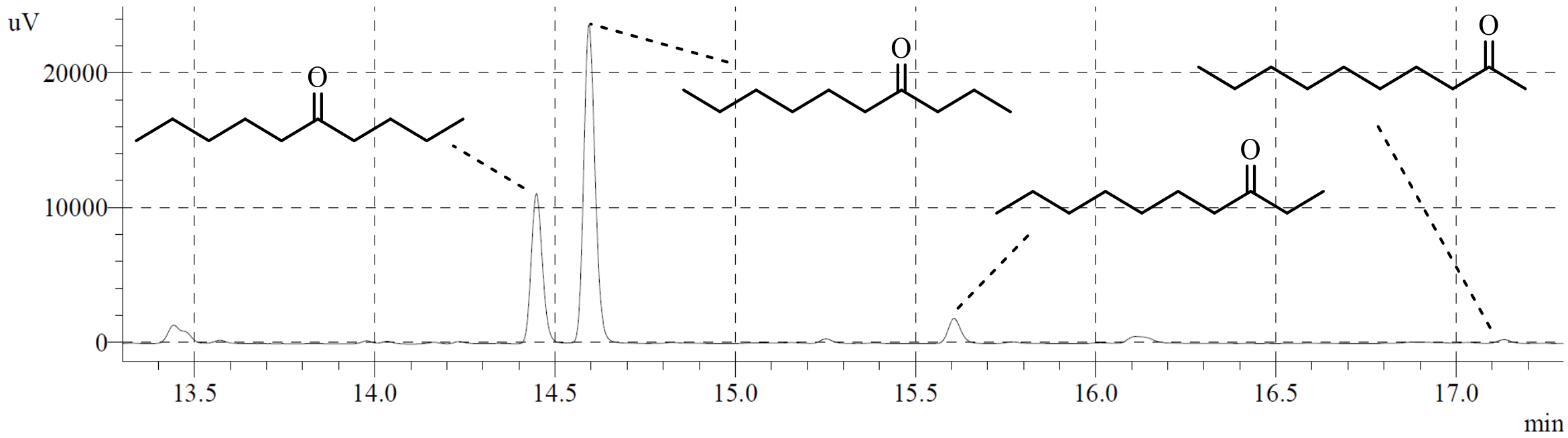

RRF(substrate): 0.833 RRF(ketone): 0.750

i	Retention time, min	Area (A)	Percentage (P), %	Percentage with RRF (P^{RRF}), %	Selectivity, %
-	7.320 (substrate)	63161	21.625	26.0	
5	14.448	24349	8.337	11.1	29.6
4	14.594	52285	17.902	23.9	63.6
3	15.607	4734	1.6208	2.2	5.8
2	17.134	790	0.27048	0.4	1.0
	22.614 (biphenyl)	58414	TON = 19; TOF = $2.1\cdot10^{-2}$ s^{-1}		
	Conversion: 74.0		Total product yield: 37.6		

The different oxidation products were assigned according to standard samples.[3]

1-Chlorodecane (S5) oxidation

The oxidation was performed using 1-chlorodecane (3.77 μL, 3.27 mg).
The following temperature-program was used: Rtx-5; 60 °C for 3 min, 25 °C/min to 130 °C, 0.5 °C/min to 140 °C, 25 °C/min to 250 °C and 250 °C for 5 min.

GC analysis of the **Mn(mcp)** catalyzed oxidation of 1-chlorodecane:

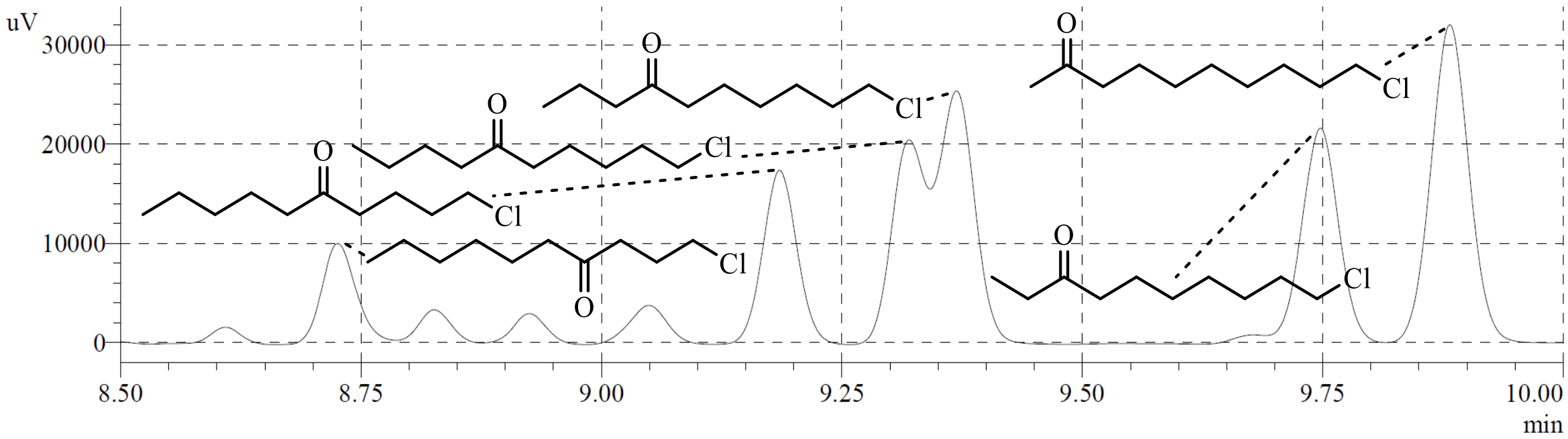

RRF(substrate): 0.833 RRF(ketone): 0.750

i	Retention time, min	Area (A)	Percentage (P), %	Percentage with RRF (P^{RRF}), %	Selectivity, %
-	6.757 (substrate)	604872	36.806	44.2	
7	8.726	25833	1.5719	2.1	7.6
6	9.185	43895	2.6709	3.6	13.0
5	9.320	51005	3.1036	4.1	15.0
4	9.369	67070	4.0811	5.4	19.8
3	9.748	61181	3.7228	5.0	18.1
2	9.882	89757	5.4616	7.3	26.5
	8.255 (biphenyl)	328685	TON = 28; TOF = $3.0 \cdot 10^{-2}$ s^{-1}		
	Conversion: 55.8		Total product yield: 27.5		

GC analysis of the **5**-catalyzed oxidation of 1-chlorodecane:

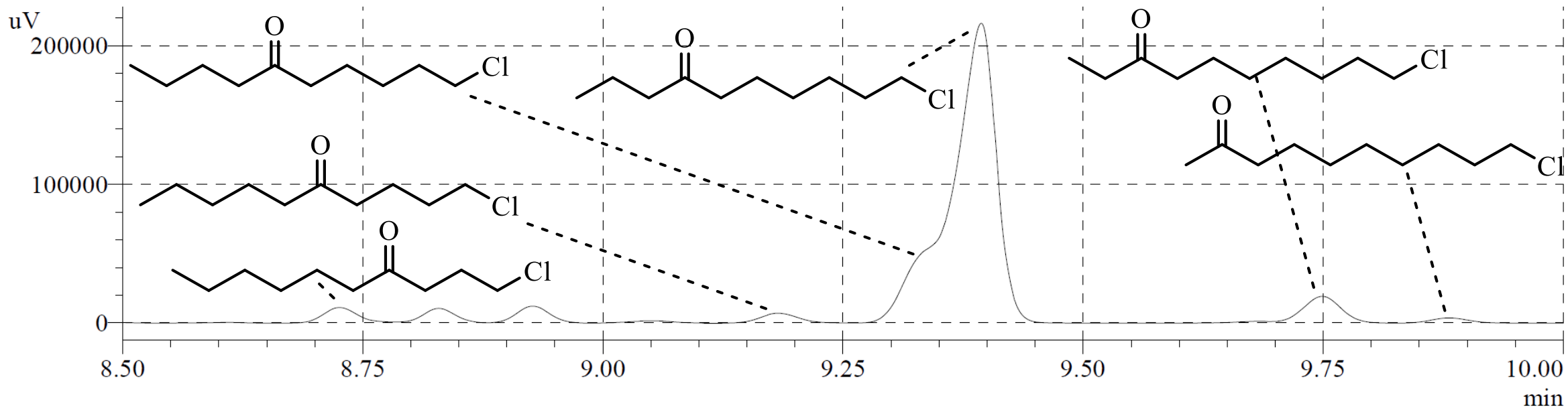

RRF(substrate): 0.833 RRF(ketone): 0.750

i	Retention time, min	Area (A)	Percentage (P), %	Percentage with RRF (P^{RRF}), %	Selectivity, %
-	6.753 (substrate)	67175	4.1718	5.0	
7	8.726	29226	1.8150	2.4	3.5
6	9.183	19929	1.2376	1.6	2.4
5	9.344	121773	7.5625	10.1	14.6
4	9.394	603599	37.485	50.0	72.2
3	9.749	50919	3.1622	4.2	6.1
2	9.881	10002	0.62116	0.8	1.2
	8.256 (biphenyl)	322044	TON = 35; TOF = $3.8 \cdot 10^{-2}$ s^{-1}		
	Conversion: 95.0		Total product yield: 69.1		

The different oxidation products were assigned according to literature data.[3]

Dodecanenitrile (S6) oxidation

The oxidation was performed using dodecanenitrile (4.04 µL, 3.35 mg).
The following temperature-program was used: Rtx-5; 60 °C for 3 min, 15 °C/min to 250 °C and 250 °C for 5 min.

GC analysis of the **Mn(mcp)** catalyzed oxidation of dodecanenitrile:

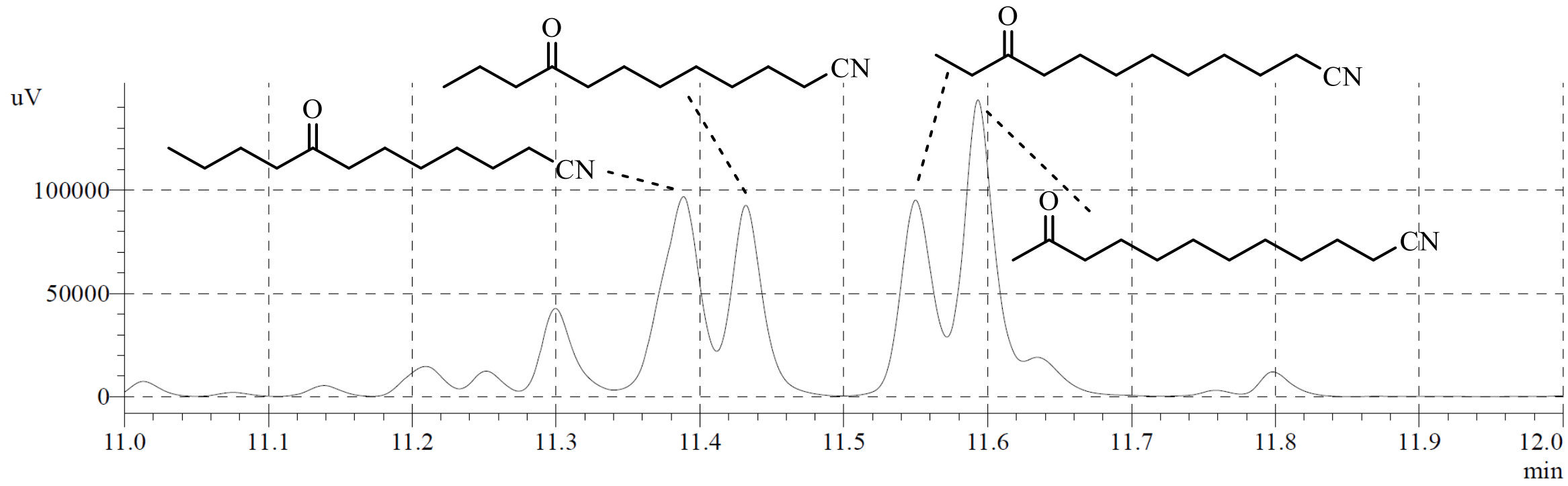

RRF(substrate): 0.942 RRF(ketone): 0.858

i	Retention time, min	Area (A)	Percentage (P), %	Percentage with RRF (P^{RRF}), %	Selectivity, %
-	9.969 (substrate)	455123	29.334	31.2	
5	11.388	190548	12.281	14.3	27.2
4	11.432	139046	8.9619	10.4	19.9
3	11.55	152936	9.8571	11.5	21.8
2	11.593	217587	14.024	16.3	31.1
	9.030 (biphenyl)	310305	TON = 53; TOF = $5.8 \cdot 10^{-2}$ s^{-1}		
	Conversion: 68.8		Total product yield: 52.6		

GC analysis of the **5**-catalyzed oxidation of dodecanenitrile:

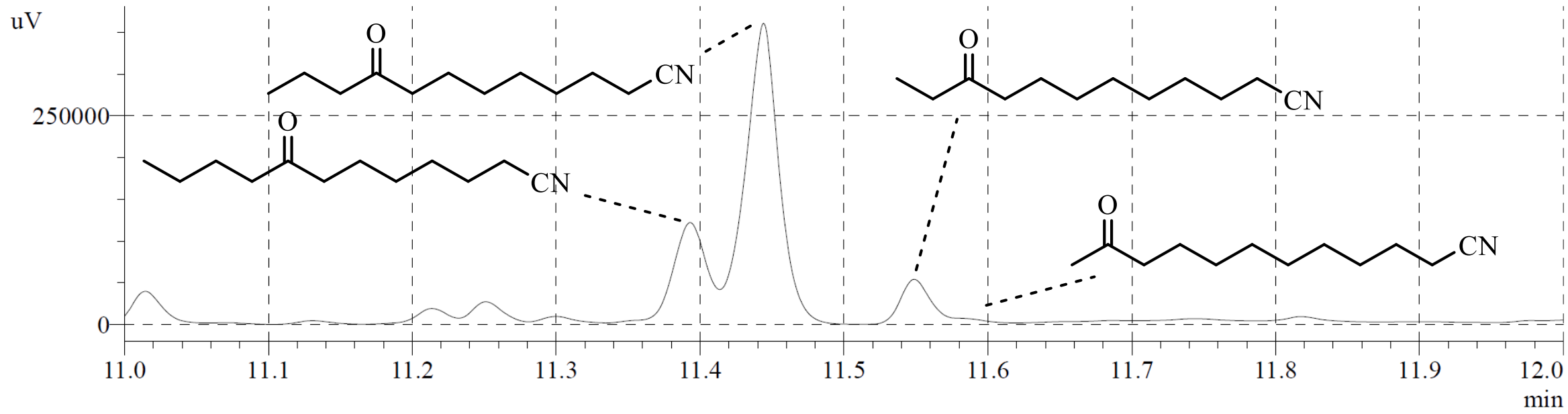

RRF(substrate): 0.942 RRF(ketone): 0.858

i	Retention time, min	Area (A)	Percentage (P), %	Percentage with RRF (P^{RRF}), %	Selectivity, %
-	9.960 (substrate)	260121	17.071	18.1	
5	11.386	146857	9.6380	11.2	20.1
4	11.434	443491	29.106	33.9	60.6
3	11.548	129128	8.4744	9.9	17.6
2	11.607	12196	0.80040	0.9	1.7
	9.024 (biphenyl)	304747	TON = 28; TOF = $3.1 \cdot 10^{-2}$ s^{-1}		
	Conversion: 81.9		Total product yield: 55.9		

GC-MS analyses of the **Mn(mcp)** (top) and **5**-catalyzed (bottom) oxidation of dodecanenitrile. The different oxidation products were assigned according to their fragmentation pattern.

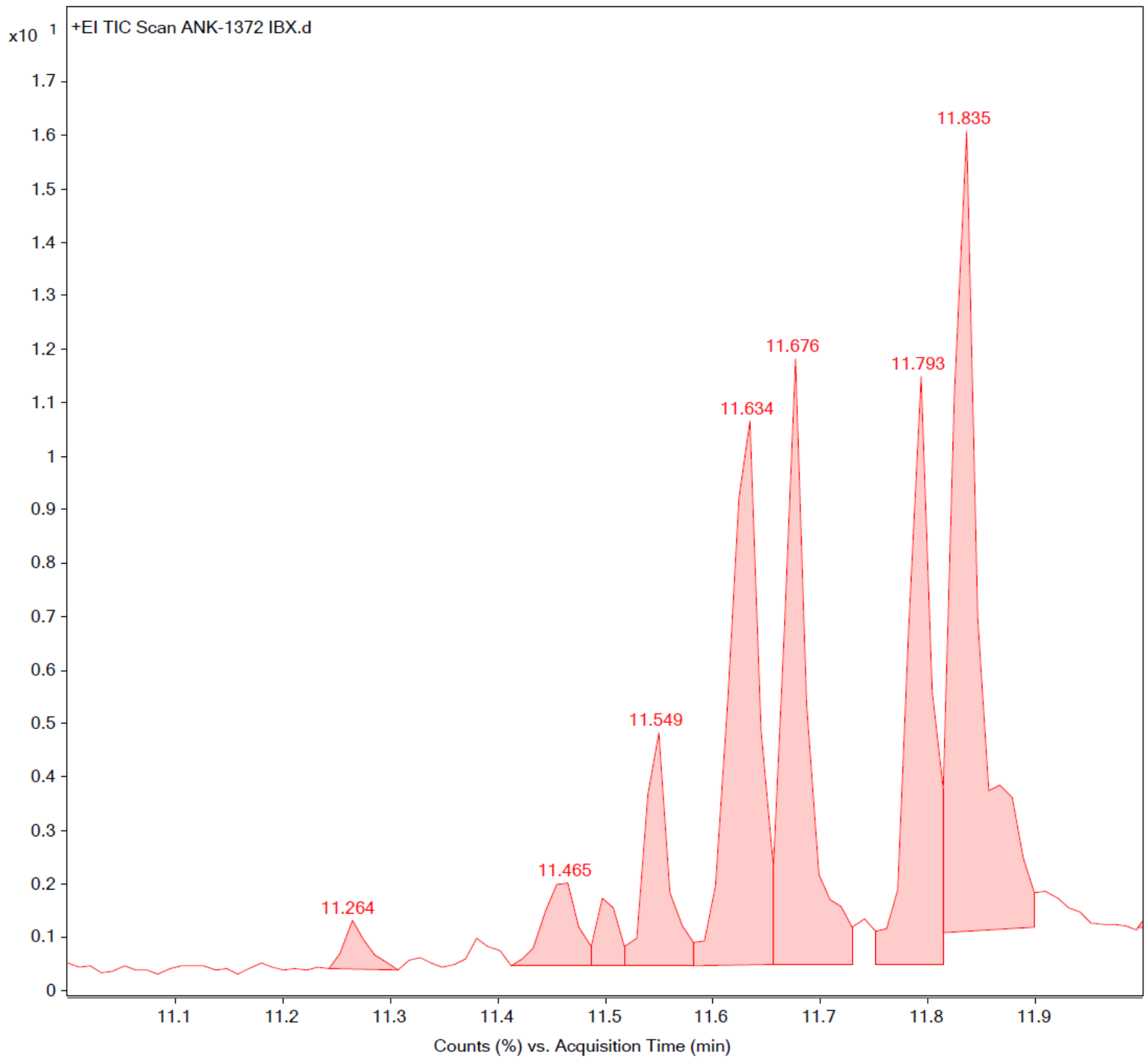

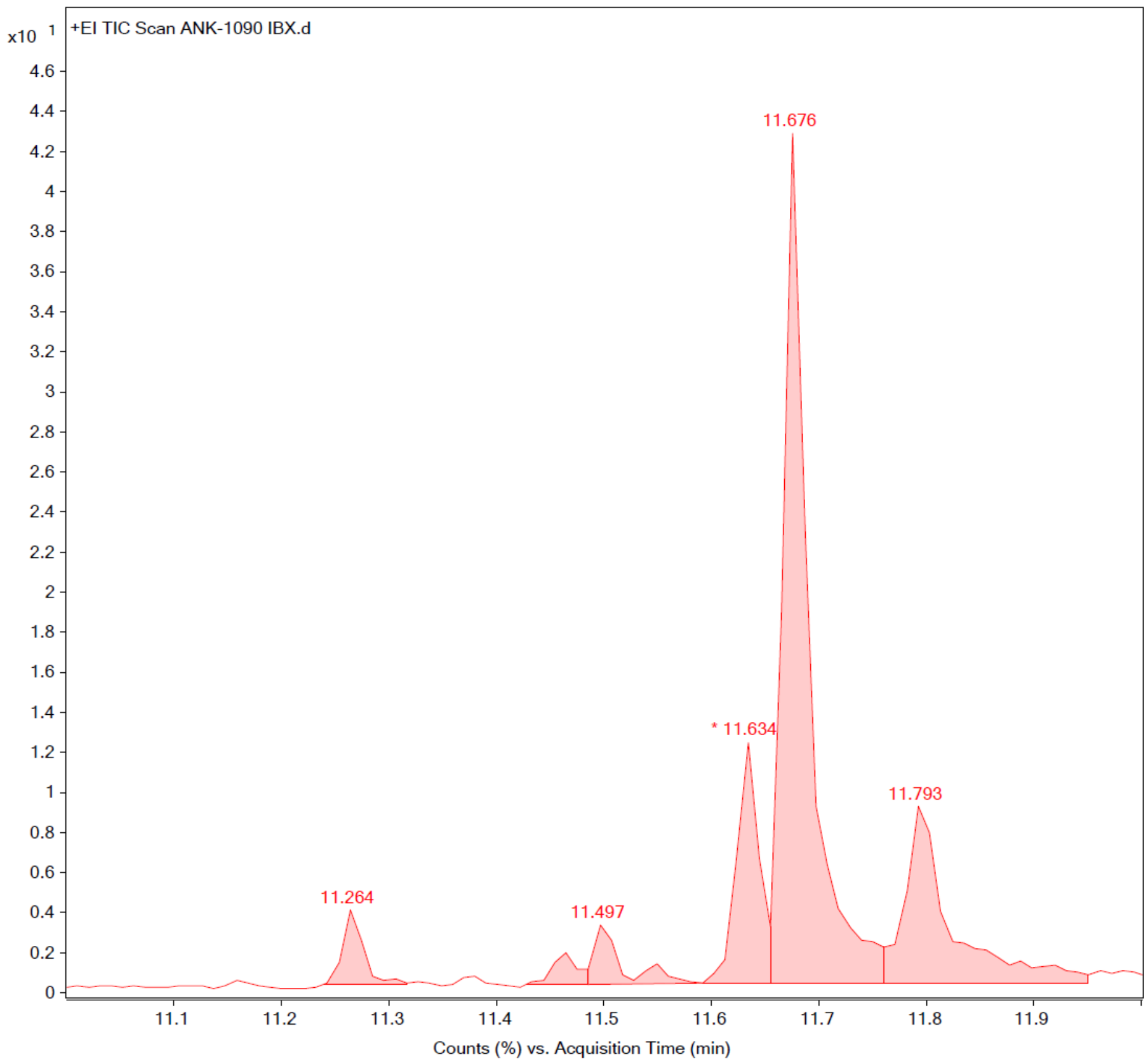

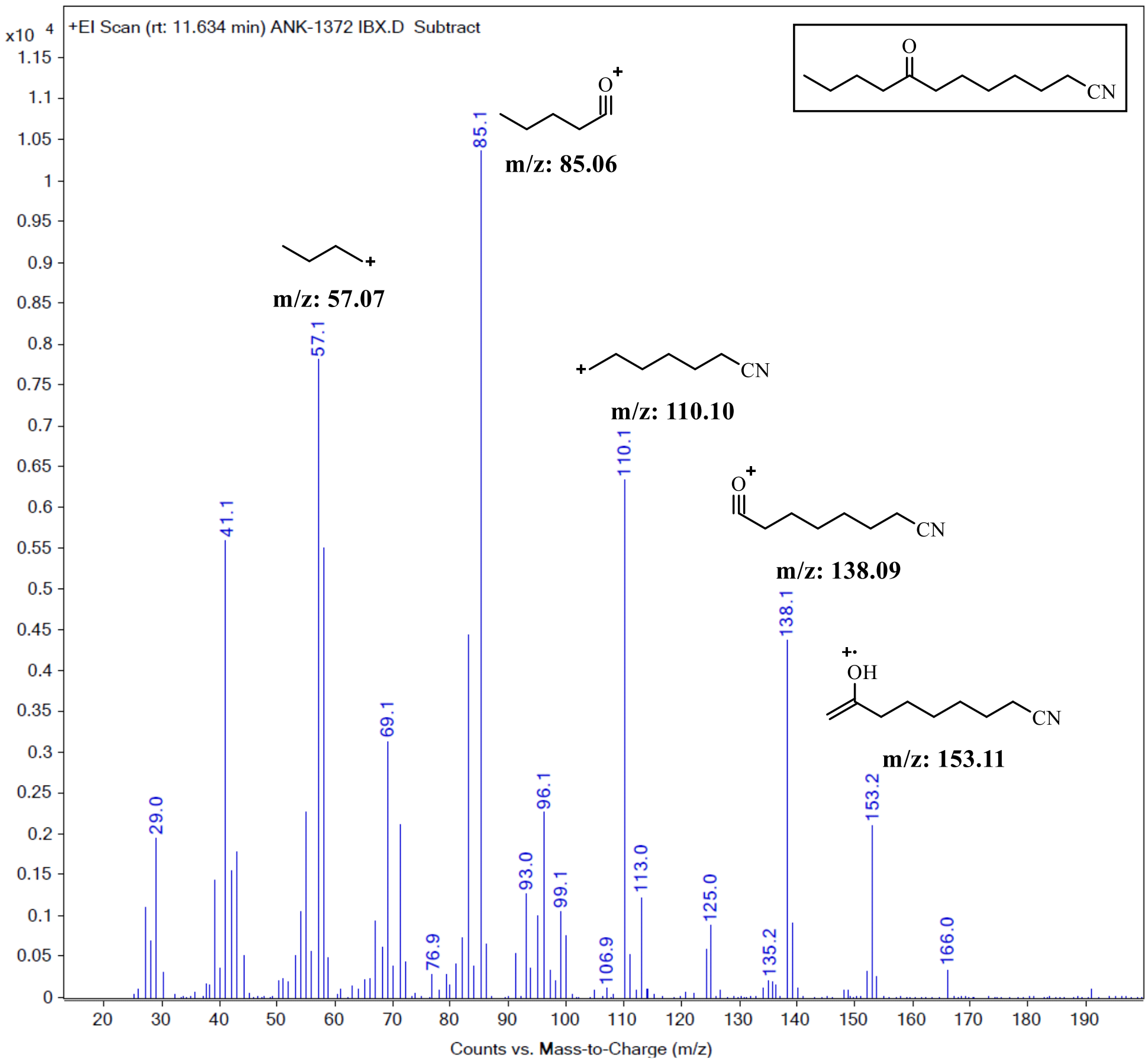
x10 4
+EI Scan (rt: 11.634 min) ANK-1372 IBX.D Subtract
CN
O
m/z: 85.06
m/z: 57.07
m/z: 110.10
m/z: 138.09
OH
m/z: 153.11
85.1
57.1
41.1
69.1
29.0
96.1
93.0
99.1
110.1
113.0
125.0
138.1
153.2
166.0
76.9
106.9
135.2
Counts vs. Mass-to-Charge (m/z)

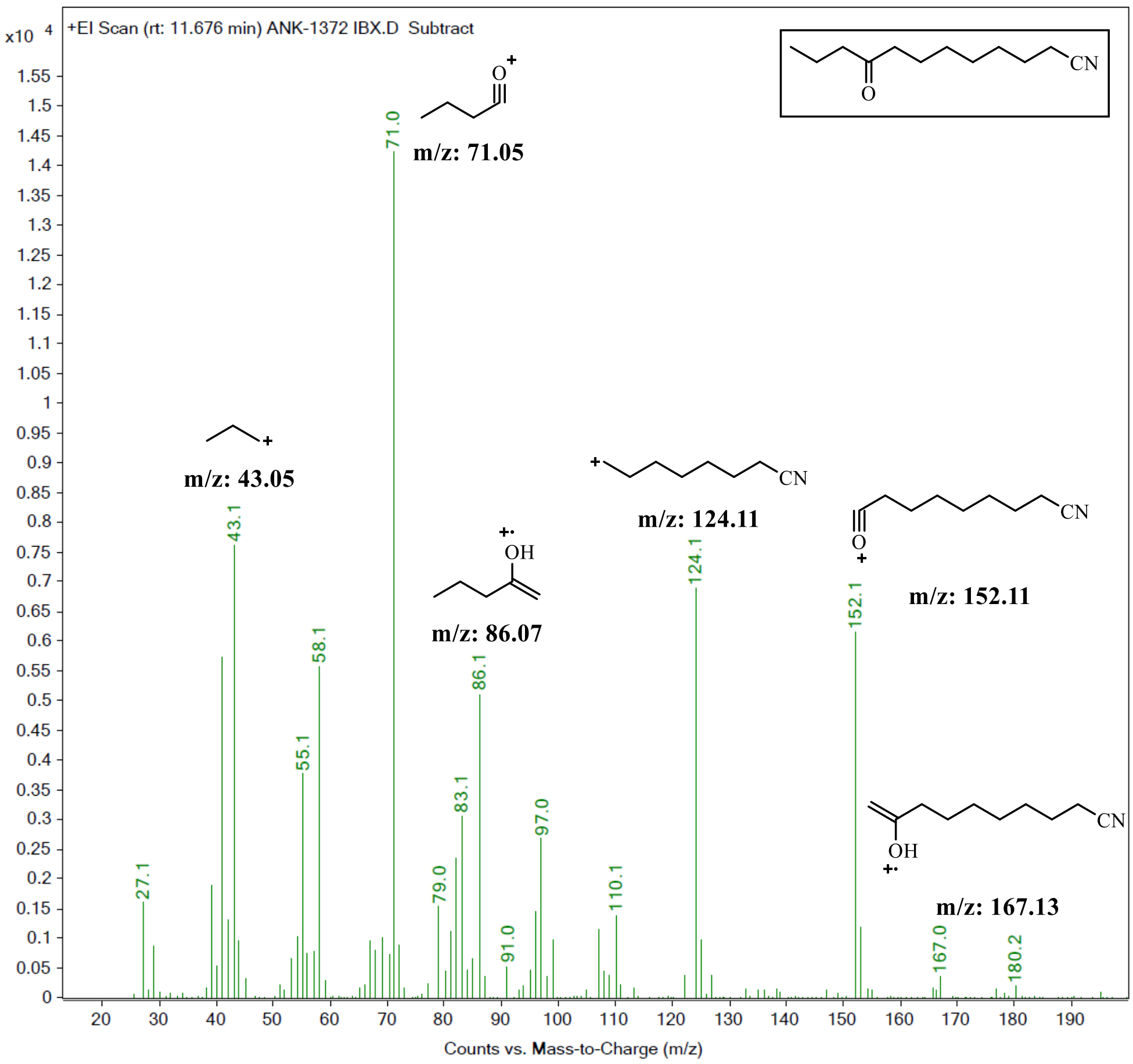
x10 4
+EI Scan (rt: 11.676 min) ANK-1372 IBX.D Subtract
CN
O
m/z: 71.05
m/z: 43.05
m/z: 124.11
m/z: 152.11
OH
m/z: 86.07
m/z: 167.13
71.0
43.1
58.1
55.1
86.1
83.1
97.0
79.0
27.1
91.0
110.1
124.1
152.1
167.0
180.2
Counts vs. Mass-to-Charge (m/z)

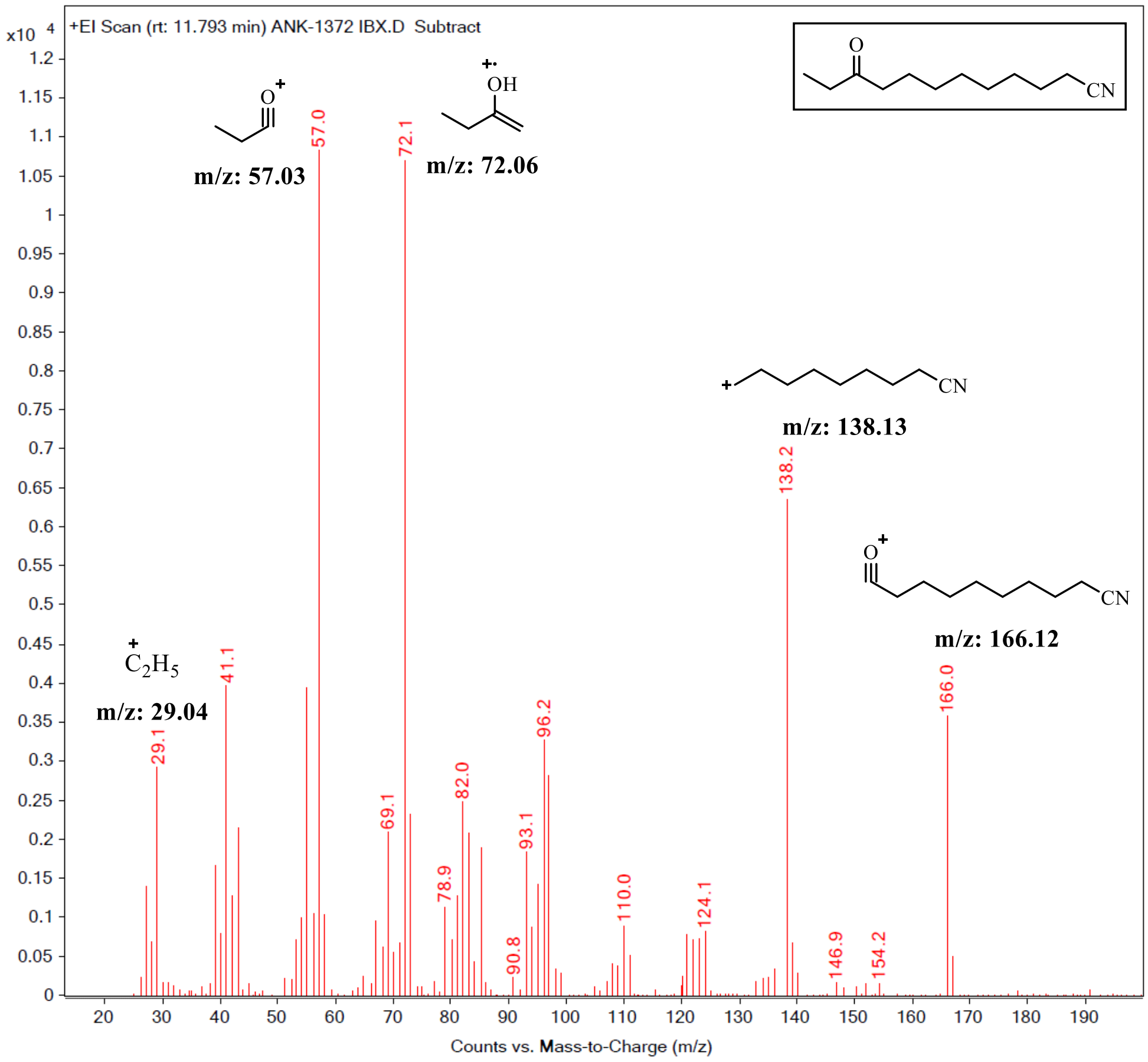
x10 4
+EI Scan (rt: 11.793 min) ANK-1372 IBX.D Subtract
m/z: 57.03
m/z: 72.06
m/z: 138.13
m/z: 166.12
m/z: 29.04
57.0
72.1
138.2
166.0
41.1
29.1
96.2
82.0
69.1
93.1
78.9
110.0
124.1
90.8
146.9
154.2
CN
OH
Counts vs. Mass-to-Charge (m/z)

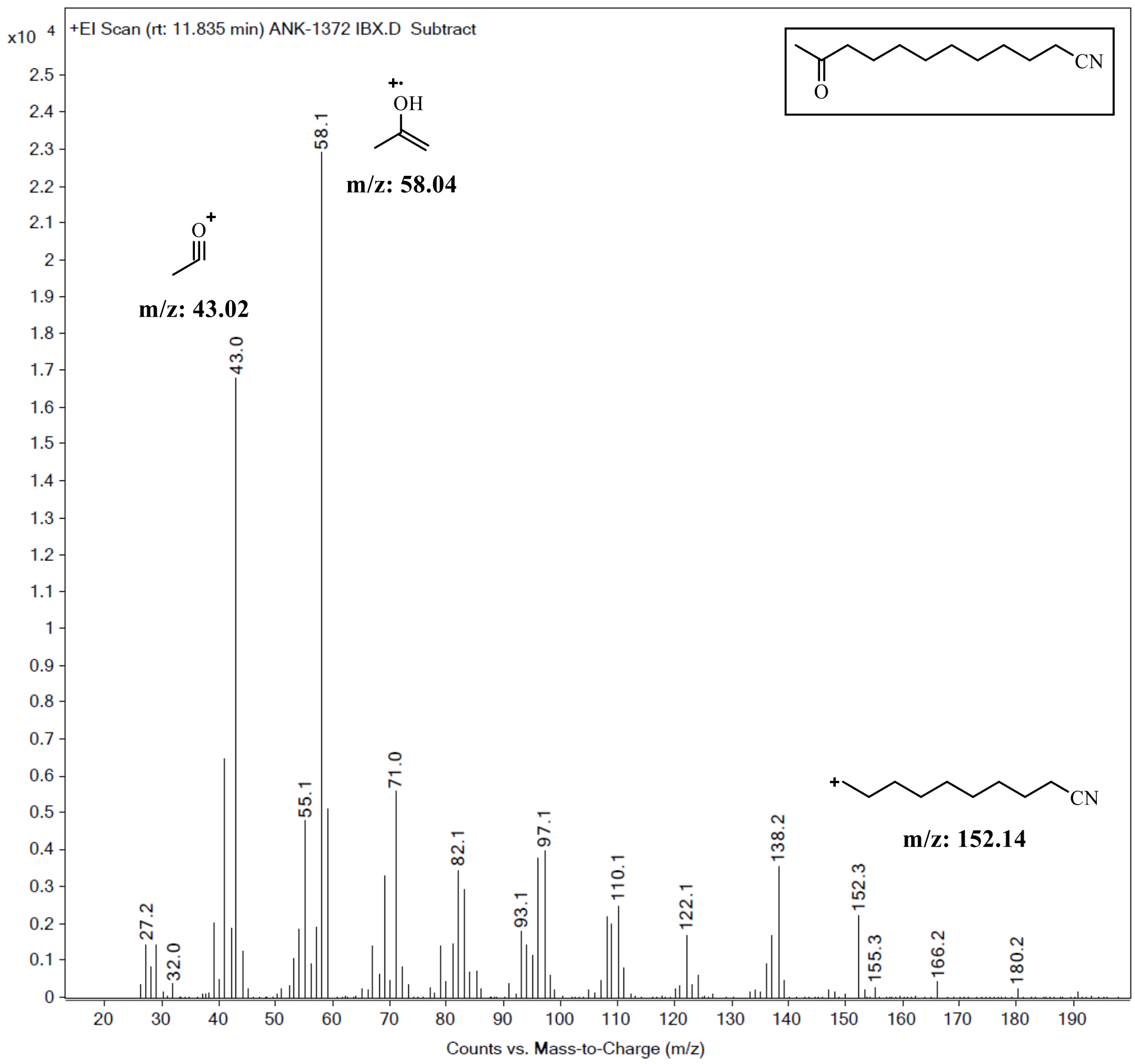
x10 4
+EI Scan (rt: 11.835 min) ANK-1372 IBX.D Subtract
m/z: 58.04
m/z: 43.02
m/z: 152.14
58.1
43.0
71.0
55.1
97.1
82.1
138.2
110.1
93.1
122.1
152.3
27.2
32.0
155.3
166.2
180.2
CN
OH
Counts vs. Mass-to-Charge (m/z)

Heptyl acetate (S7) oxidation

The oxidation was performed using heptyl acetate (3.36 µL, 2.93 mg).
The following temperature-program was used: Rtx-5; start at 110 °C, 0.5 °C/min to 120 °C, 25 °C/min to 250 °C and 250 °C for 3 min.

GC analysis of the **Mn(mcp)** catalyzed oxidation of heptyl acetate:

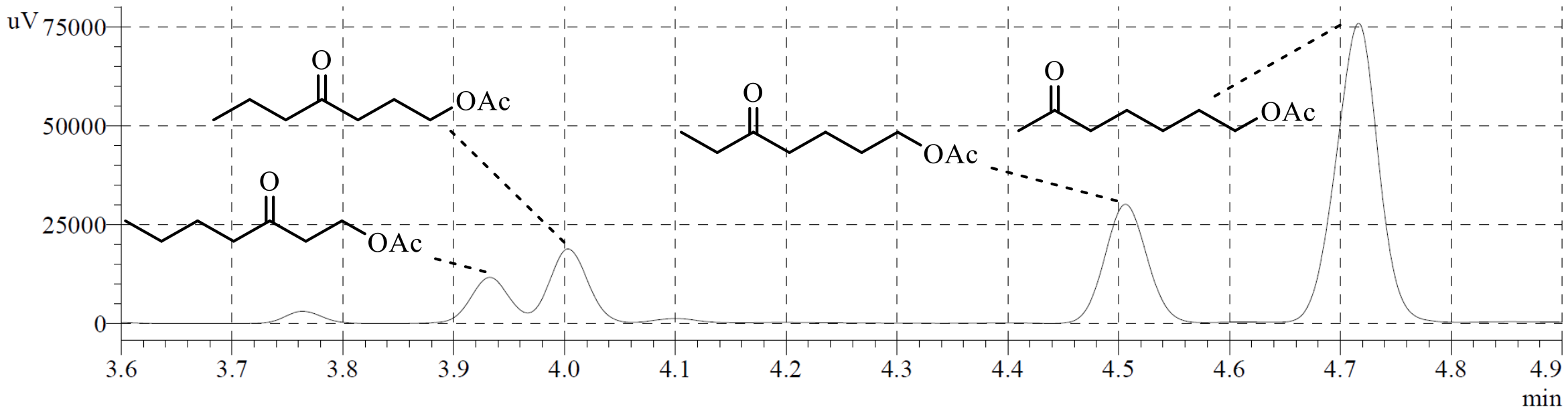

RRF(substrate): 0.667 RRF(ketone): 0.583

i	Retention time, min	Area (A)	Percentage (P), %	Percentage with RRF (P^{RRF}), %	Selectivity, %
-	2.035 (substrate)	207751	15.323	23.0	
5	3.933	28074	2.0706	3.5	7.9
4	4.003	45012	3.3199	5.7	12.6
3	4.506	80062	5.9051	10.1	22.5
2	4.716	203106	14.980	25.7	57.0
	6.919 (biphenyl)	271162	TON = 45; TOF = $5.0 \cdot 10^{-2}$ s^{-1}		
	Conversion: 77.0		Total product yield: 45.0		

GC analysis of the **5**-catalyzed oxidation of heptyl acetate:

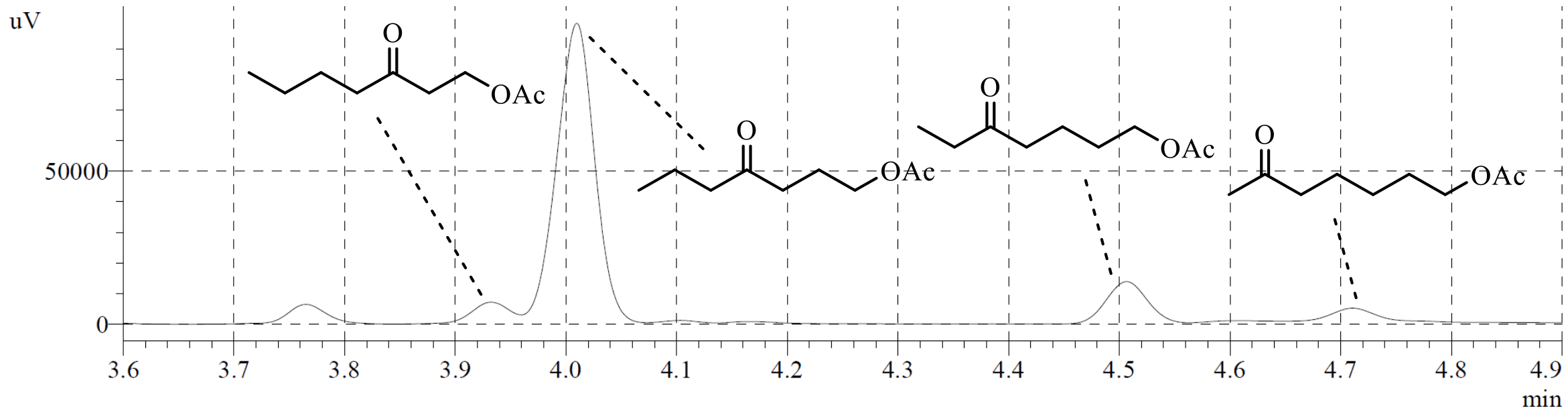

RRF(substrate): 0.667 RRF(ketone): 0.583

i	Retention time, min	Area (A)	Percentage (P), %	Percentage with RRF (P^{RRF}), %	Selectivity, %
-	2.037 (substrate)	335400	25.204	37.8	
5	3.932	18208	1.3682	2.3	6.0
4	4.01	235844	17.723	30.4	78.2
3	4.507	35061	2.6347	4.5	11.6
2	4.711	12490	0.93857	1.6	4.1
	6.920 (biphenyl)	266150	TON = 19; TOF = $2.1 \cdot 10^{-2}$ s^{-1}		
	Conversion: 62.2		Total product yield: 38.8		

GC-MS analyses of the **Mn(mcp)** (top) and **5**-catalyzed (bottom) oxidation of heptyl acetate. The different oxidation products were assigned according to their fragmentation pattern.

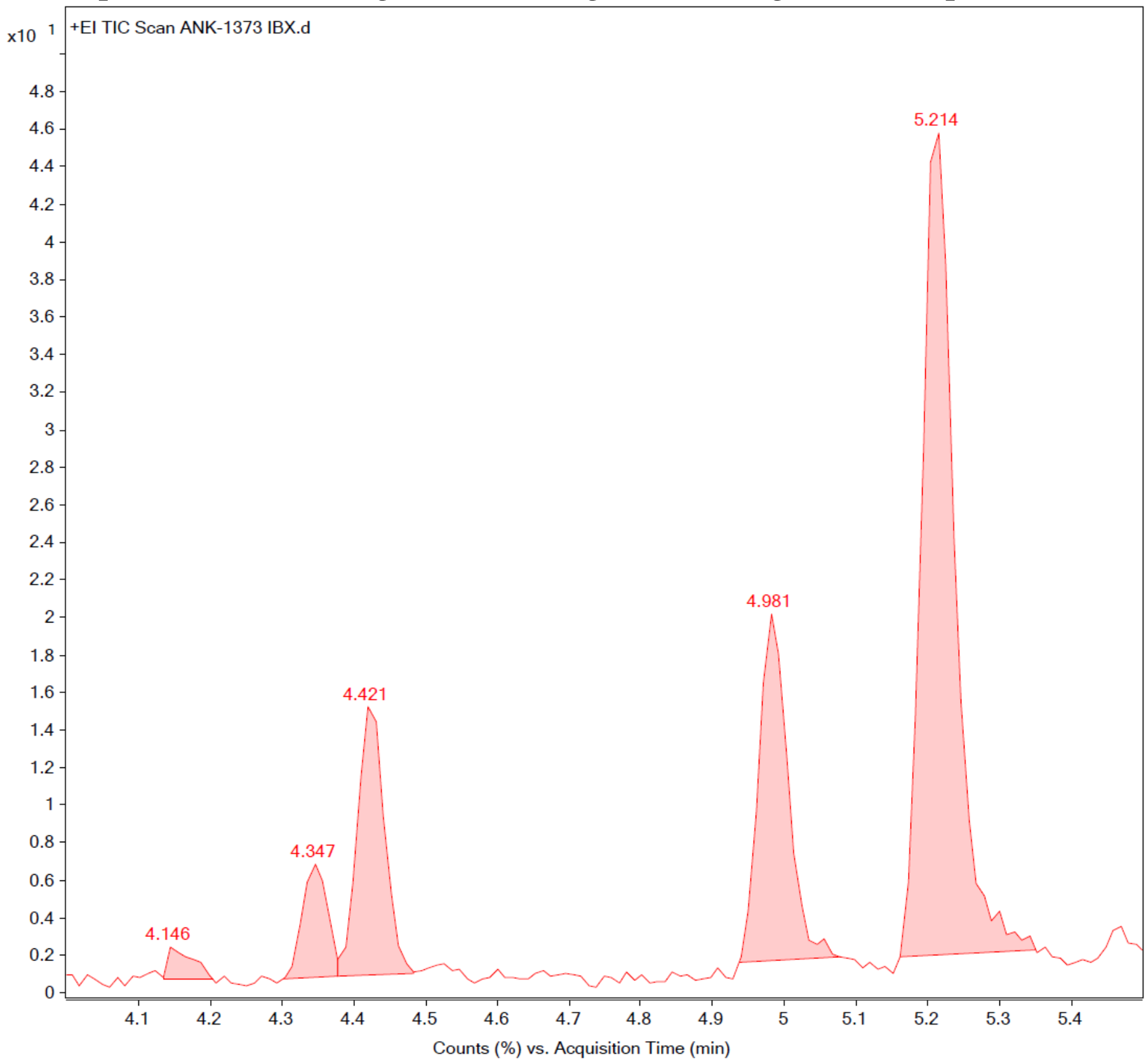

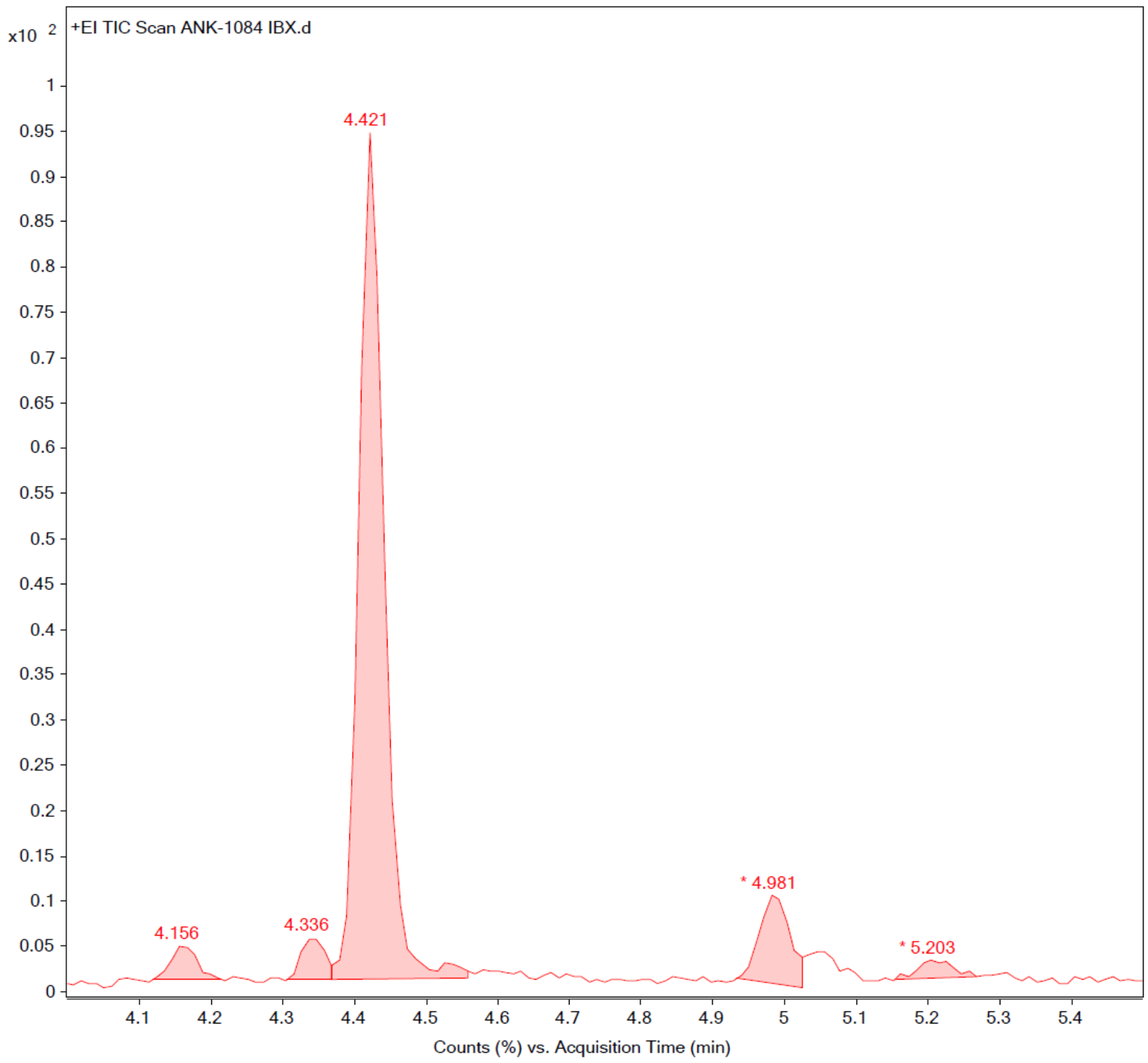

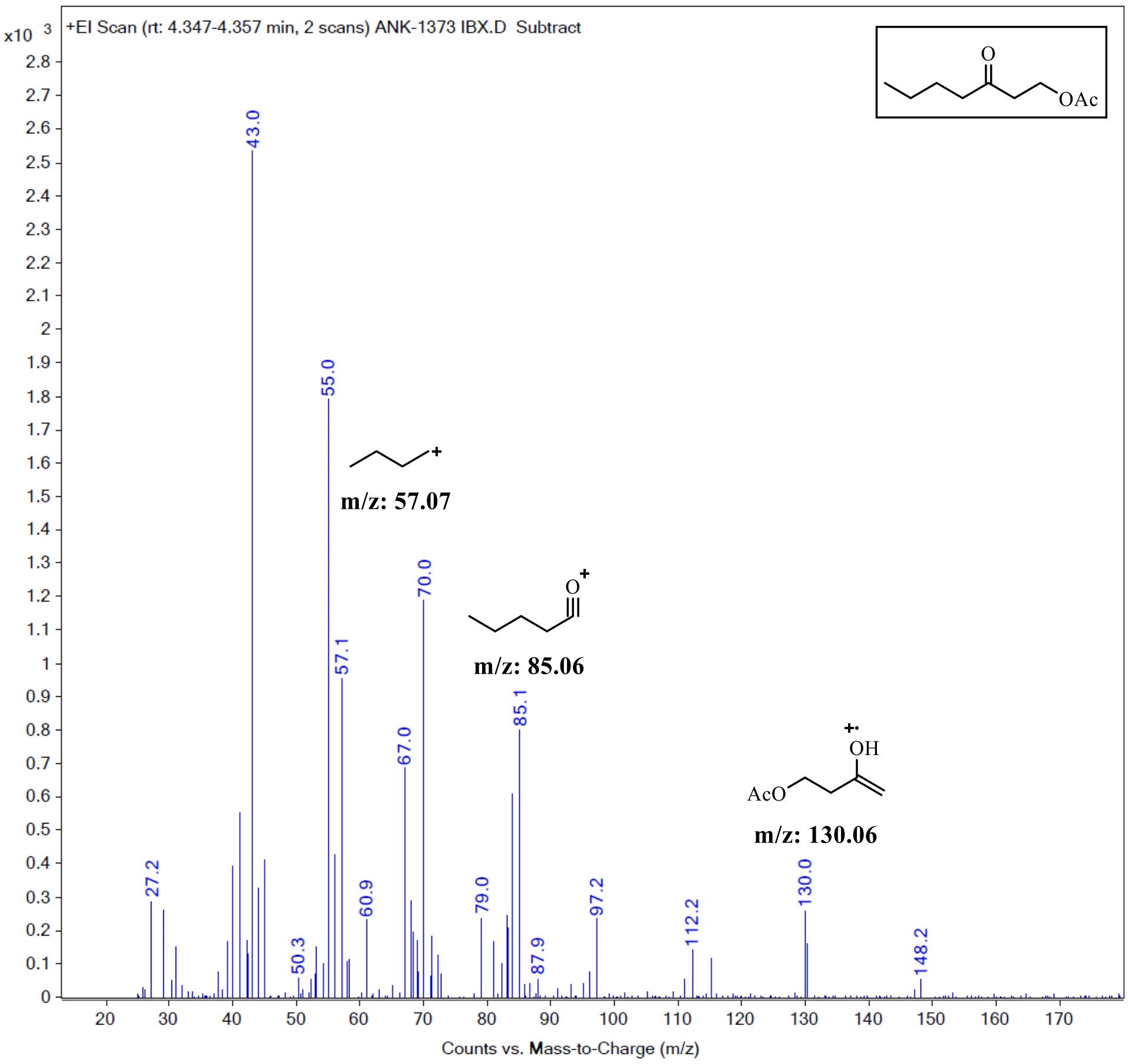

+EI Scan (rt: 4.347-4.357 min, 2 scans) ANK-1373 IBX.D Subtract
x10 3
m/z: 57.07
m/z: 85.06
m/z: 130.06
OAc
AcO
OH
Counts vs. Mass-to-Charge (m/z)

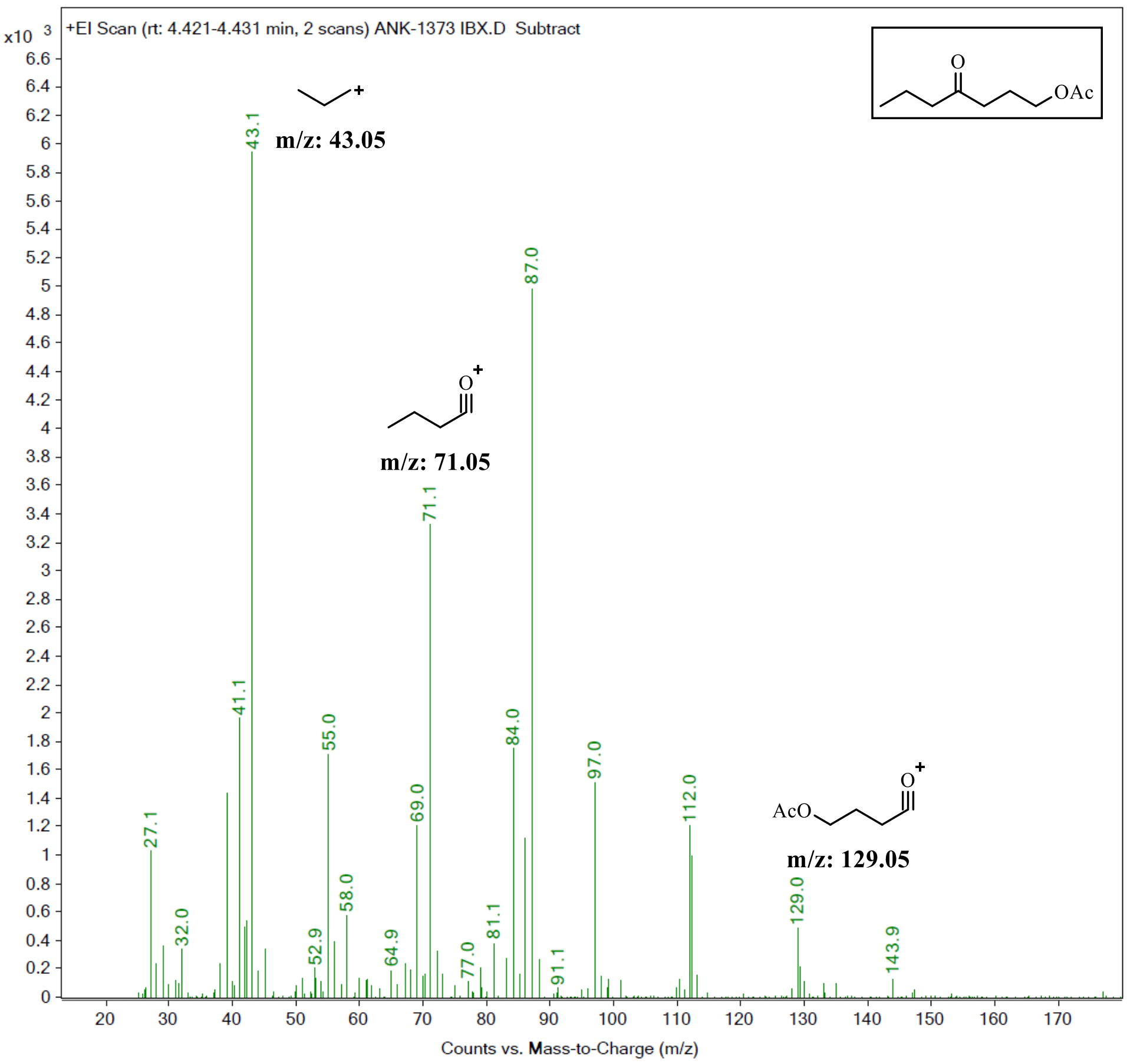

+EI Scan (rt: 4.421-4.431 min, 2 scans) ANK-1373 IBX.D Subtract
x10 3
m/z: 43.05
m/z: 71.05
m/z: 129.05
OAc
AcO
Counts vs. Mass-to-Charge (m/z)

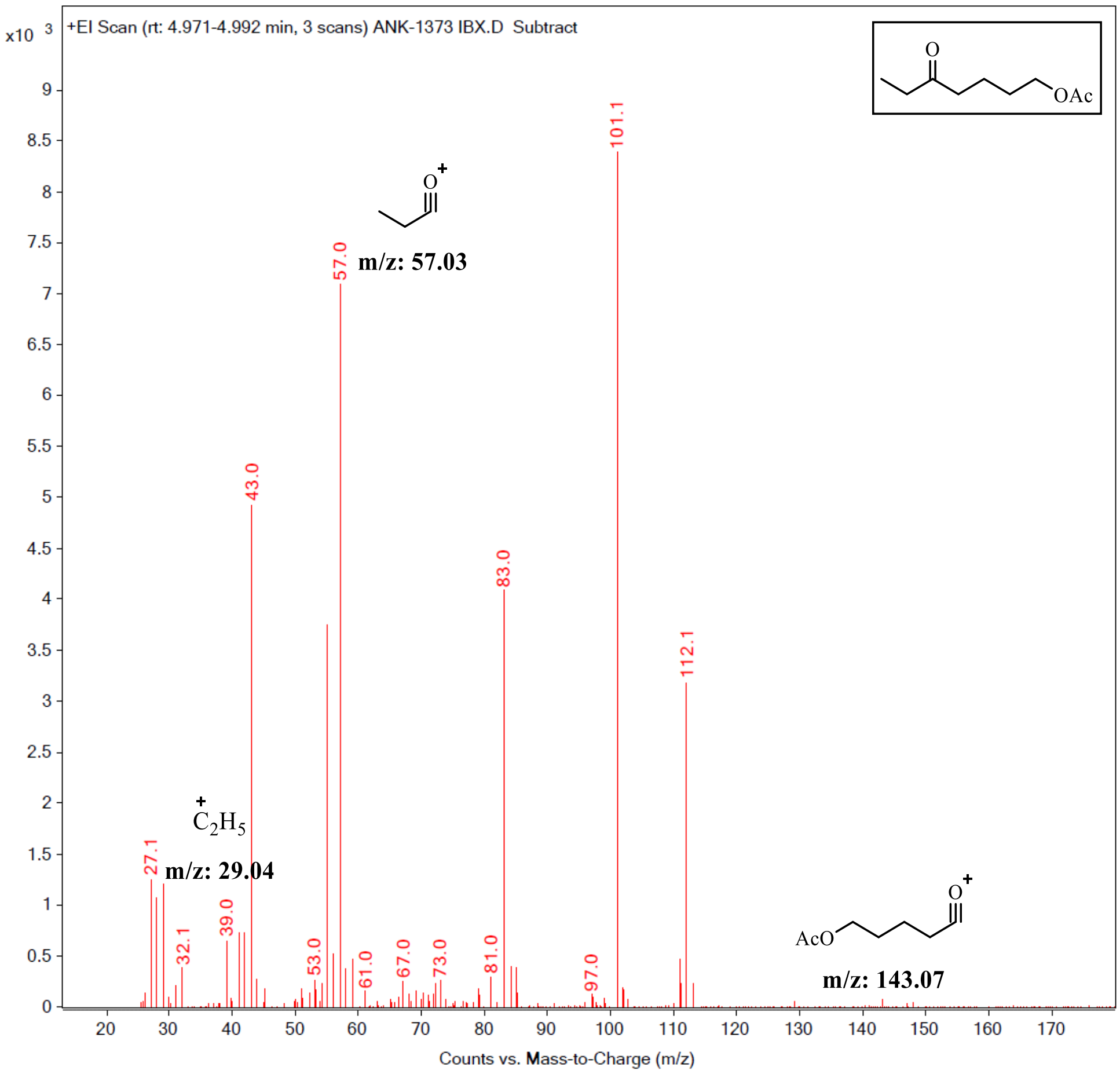
+EI Scan (rt: 4.971-4.992 min, 3 scans) ANK-1373 IBX.D Subtract
x10 3
OAc
m/z: 57.03
m/z: 29.04
AcO
m/z: 143.07
101.1
57.0
43.0
83.0
112.1
27.1
32.1
39.0
53.0
61.0
67.0
73.0
81.0
97.0
Counts vs. Mass-to-Charge (m/z)

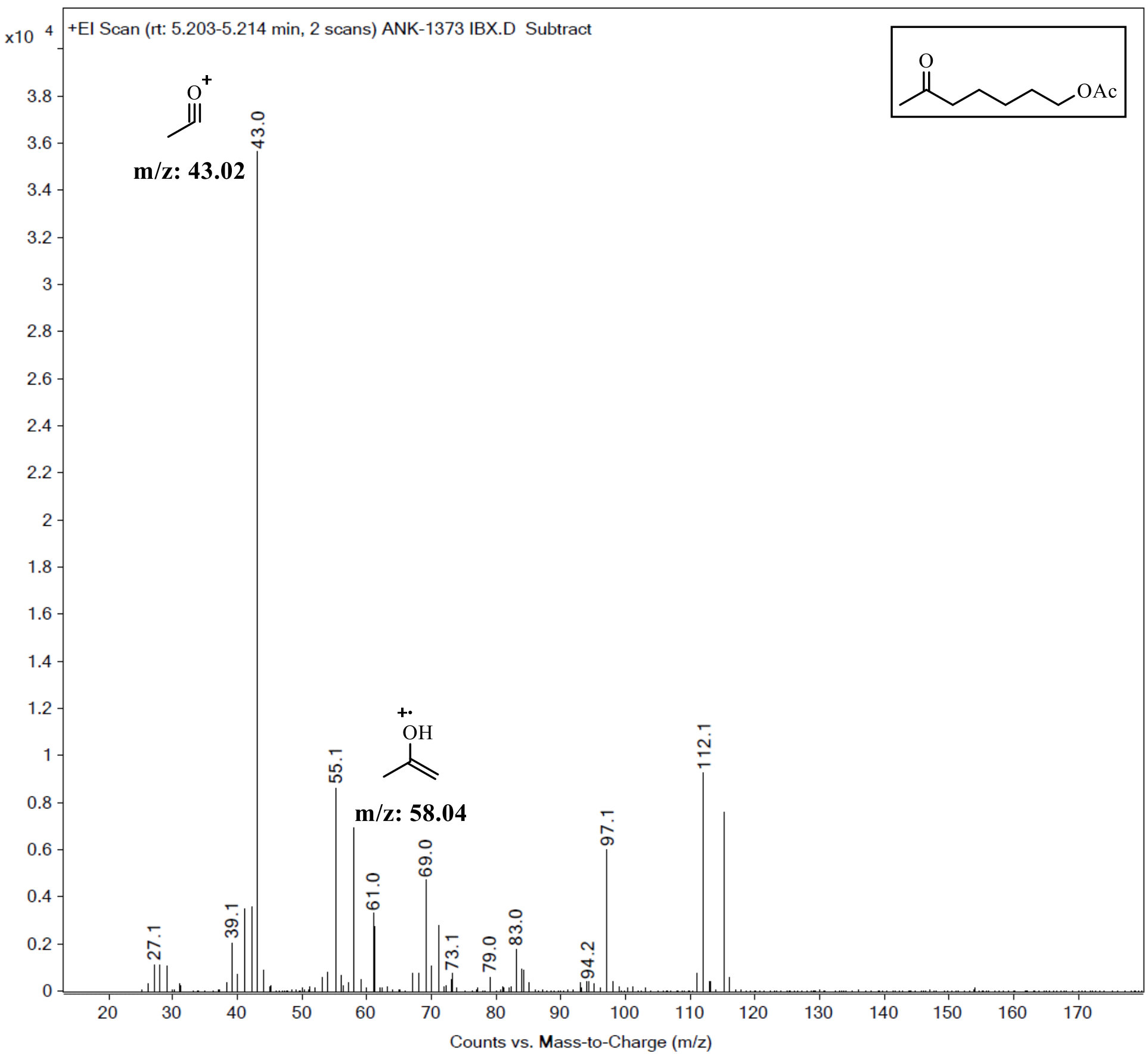
+EI Scan (rt: 5.203-5.214 min, 2 scans) ANK-1373 IBX.D Subtract
x10 4
OAc
m/z: 43.02
OH
m/z: 58.04
43.0
55.1
112.1
97.1
69.0
61.0
39.1
27.1
73.1
79.0
83.0
94.2
Counts vs. Mass-to-Charge (m/z)

Ethyl nonanoate (S8) oxidation

The oxidation was performed using ethyl nonanoate (4.28 µL, 3.71 mg).
The following temperature-program was used: Rtx-5; 60 °C for 3 min, 25 °C/min to 150 °C, 0.5 °C/min to 160 °C, 25 °C/min to 250 °C and 250 °C for 5 min.

GC analysis of the **Mn(mcp)** catalyzed oxidation of ethyl nonanoate:

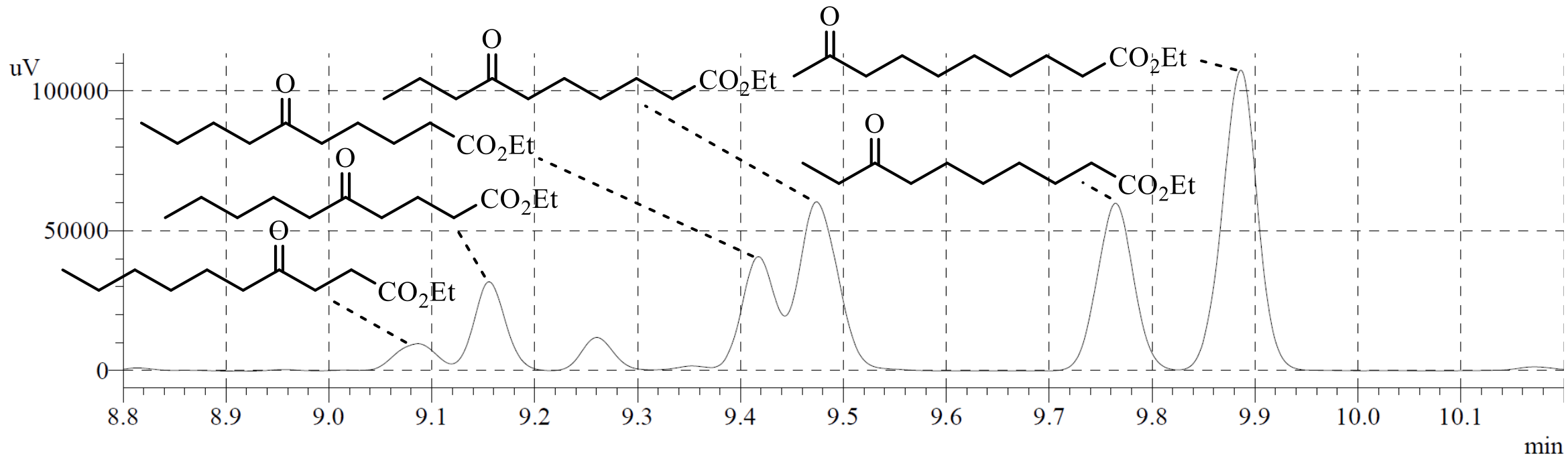

RRF(substrate): 0.917 RRF(ketone): 0.833

i	Retention time, min	Area (A)	Percentage (P), %	Percentage with RRF (P^{RRF}), %	Selectivity, %
-	7.478 (substrate)	390689	24.178	26.4	
7	9.086	26104	1.6155	1.9	3.4
6	9.156	68417	4.2340	5.1	9.0
5	9.417	100243	6.2036	7.4	13.2
4	9.474	158661	9.8189	11.8	20.8
3	9.765	149300	9.2395	11.1	19.6
2	9.886	258410	15.992	19.2	34.0
	7.434 (biphenyl)	323176	TON = 56; TOF = $6.3\cdot10^{-2}$ s^{-1}		
	Conversion: 73.6		Total product yield: 56.5		

GC analysis of the **5**-catalyzed oxidation of ethyl nonanoate:

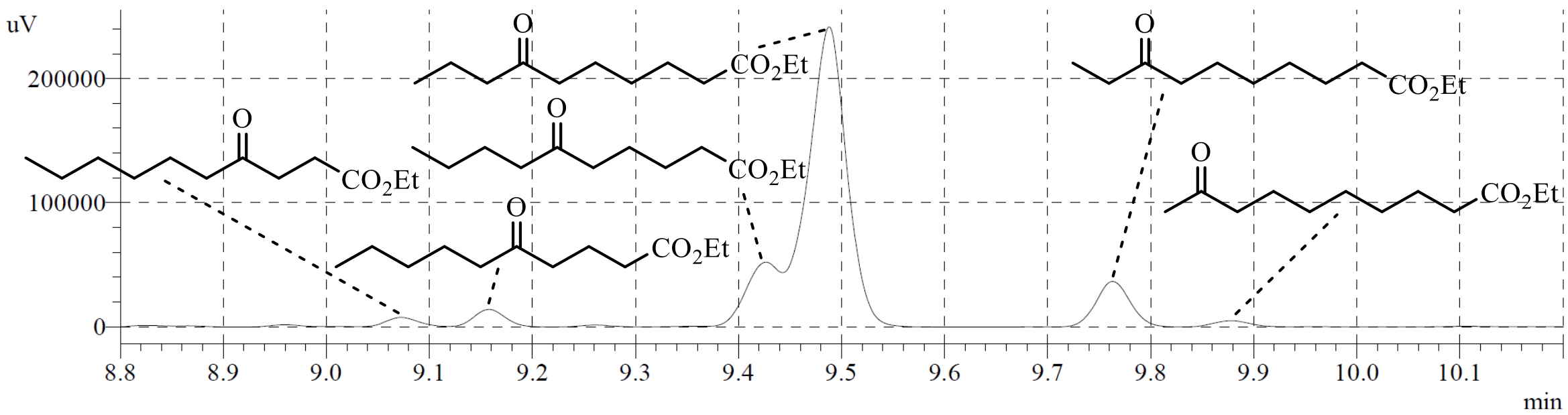

RRF(substrate): 0.917 RRF(ketone): 0.833

i	Retention time, min	Area (A)	Percentage (P), %	Percentage with RRF (P^{RRF}), %	Selectivity, %
-	7.476 (substrate)	235798	15.051	16.4	
7	9.072	16386	1.0459	1.2	1.8
6	9.158	29887	1.9077	2.3	3.3
5	9.427	127149	8.1162	9.7	14.1
4	9.488	628222	40.100	48.1	69.8
3	9.763	85631	5.4660	6.6	9.5
2	9.878	12709	0.81124	1.0	1.4
	7.434 (biphenyl)	313323	TON = 34; TOF = $3.8\cdot10^{-2}$ s^{-1}		
	Conversion: 83.6		Total product yield: 68.9		

GC-MS analyses of the **Mn(mcp)** (top) and **5**-catalyzed (bottom) oxidation of ethyl nonanoate. The different oxidation products were assigned according to their fragmentation pattern.

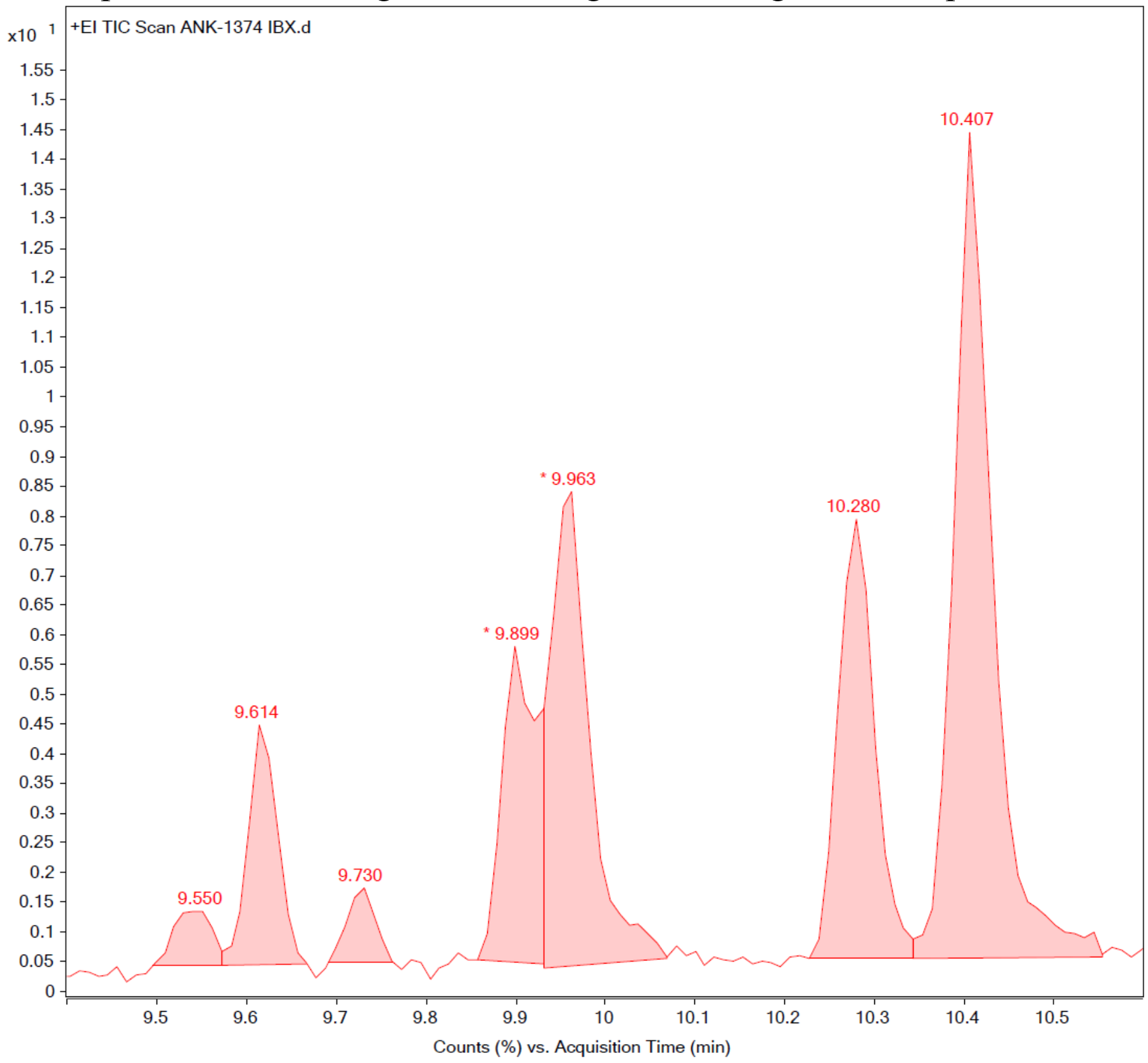

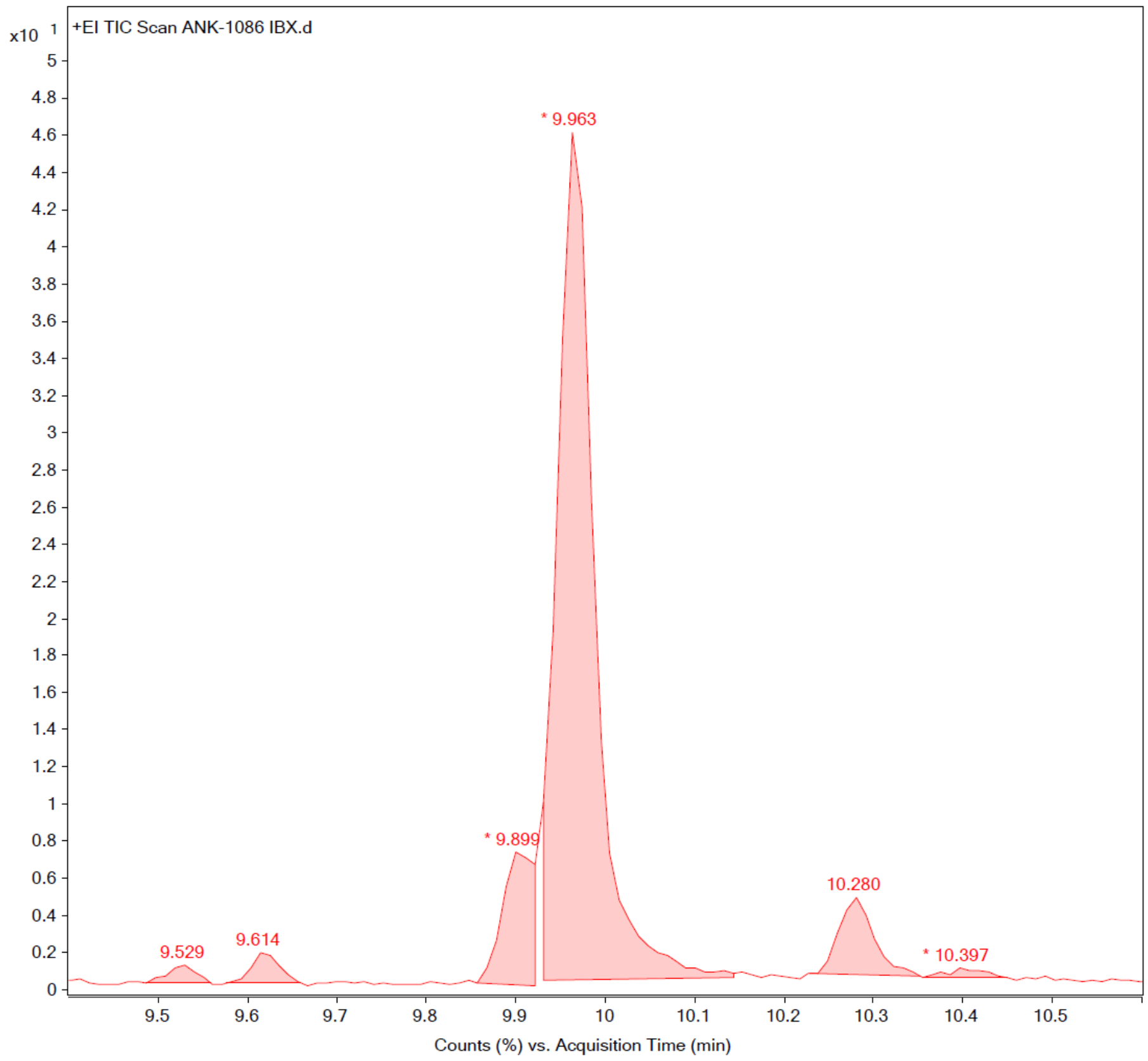

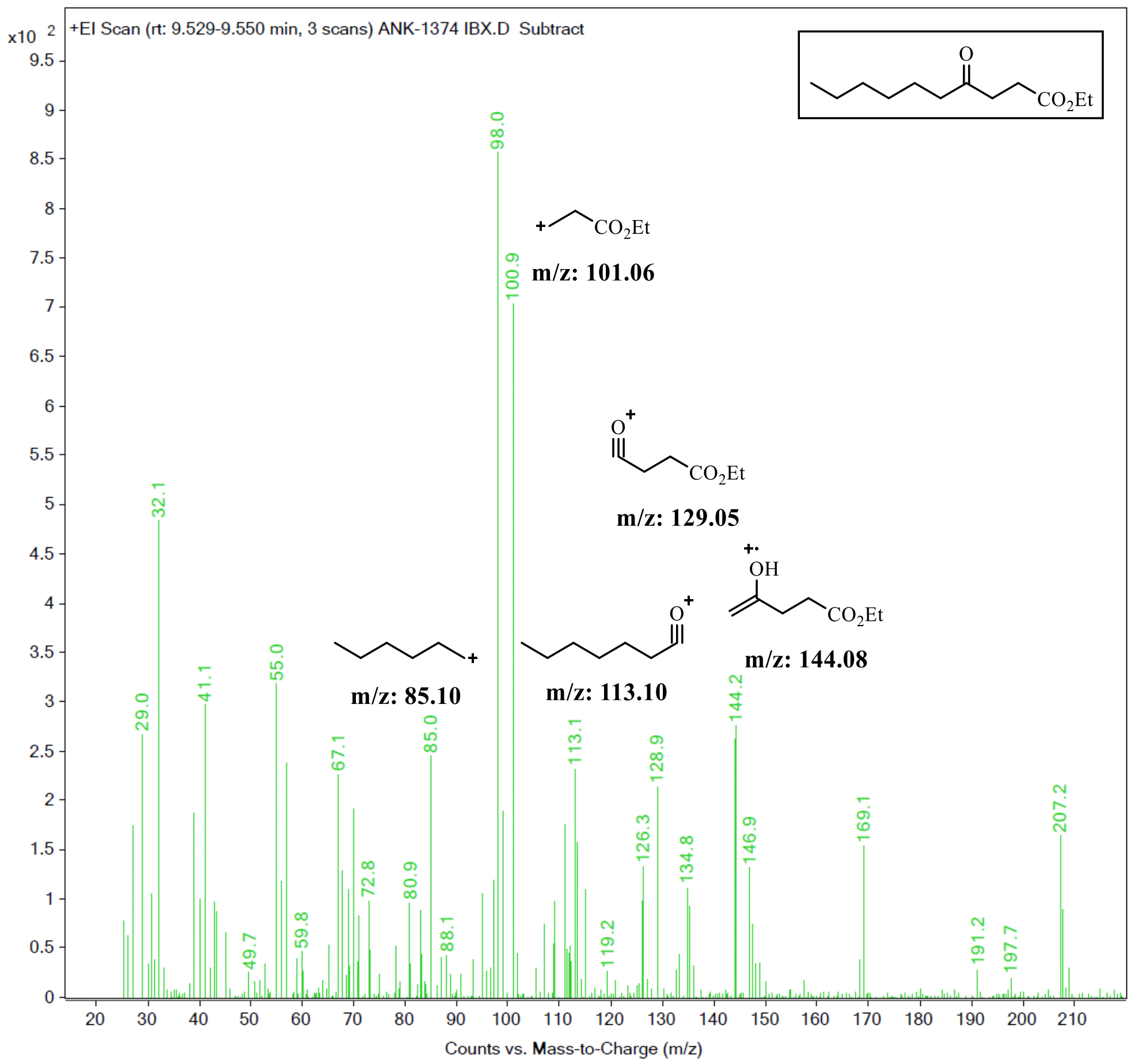

+EI Scan (rt: 9.529-9.550 min, 3 scans) ANK-1374 IBX.D Subtract
x10 2
CO2Et
m/z: 101.06
m/z: 129.05
m/z: 144.08
m/z: 85.10
m/z: 113.10
Counts vs. Mass-to-Charge (m/z)

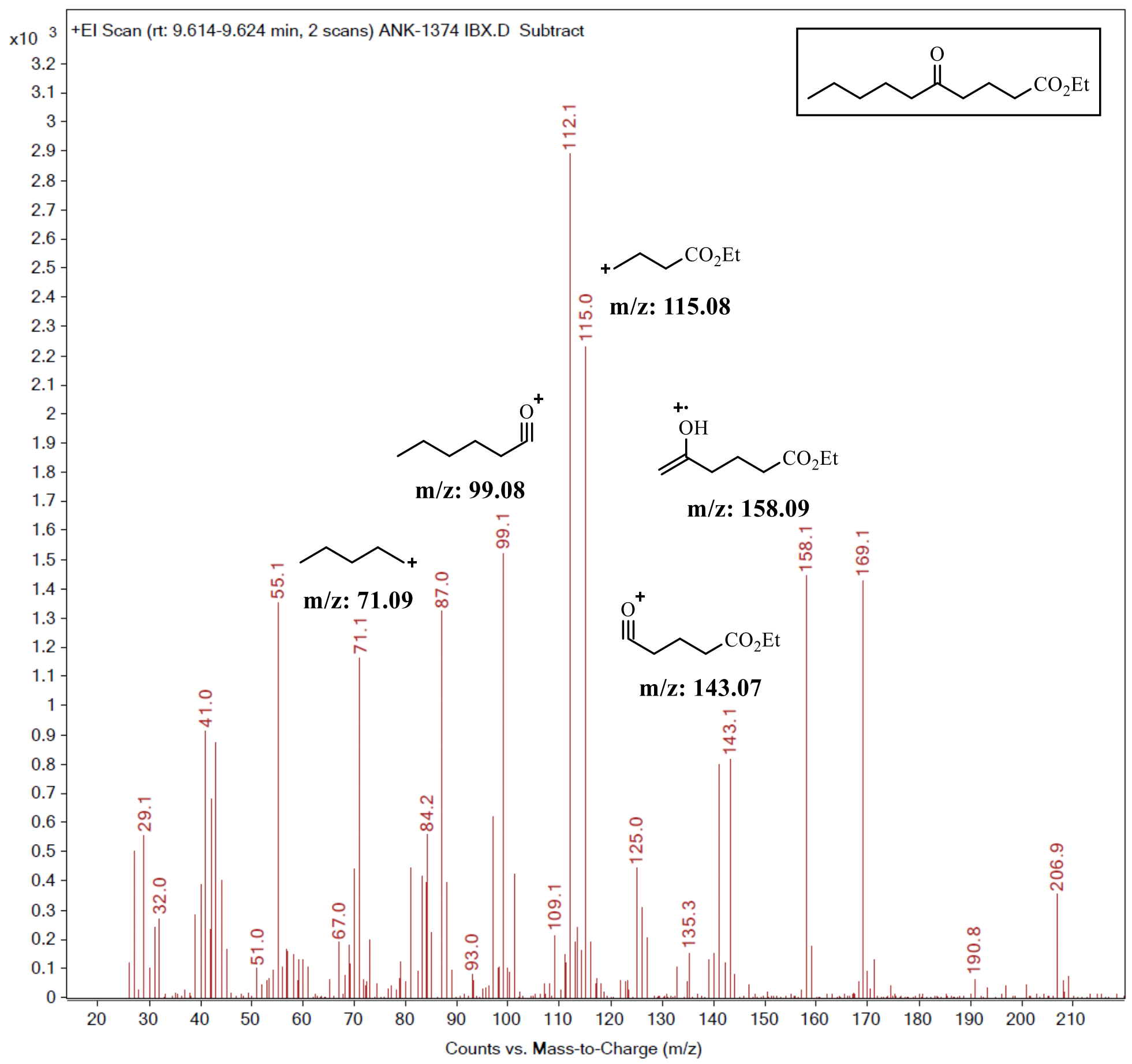

+EI Scan (rt: 9.614-9.624 min, 2 scans) ANK-1374 IBX.D Subtract
x10 3
CO2Et
m/z: 115.08
m/z: 99.08
m/z: 158.09
m/z: 71.09
m/z: 143.07
Counts vs. Mass-to-Charge (m/z)

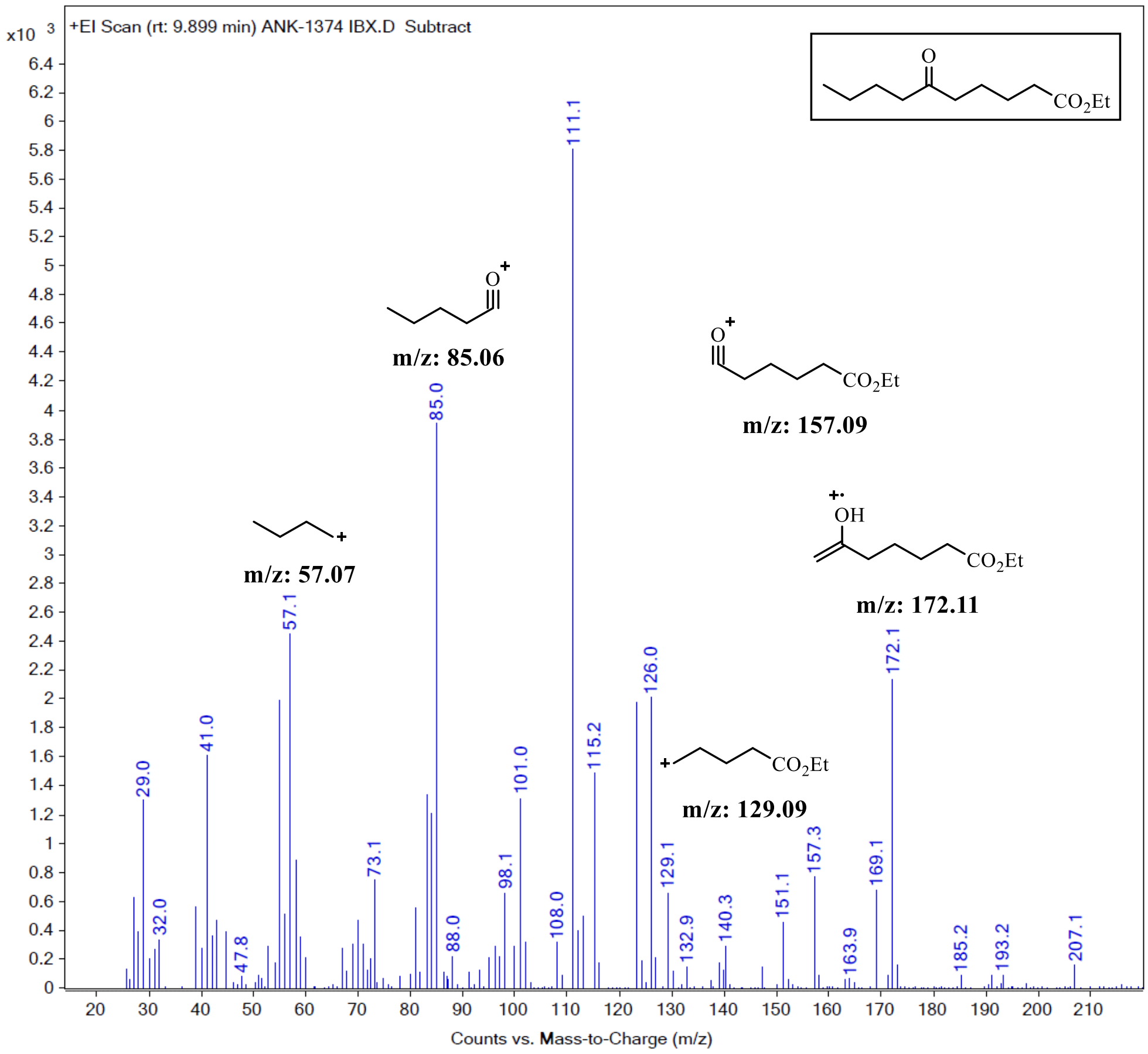
+EI Scan (rt: 9.899 min) ANK-1374 IBX.D Subtract
x10 3
m/z: 85.06
m/z: 157.09
m/z: 57.07
m/z: 172.11
m/z: 129.09
Counts vs. Mass-to-Charge (m/z)

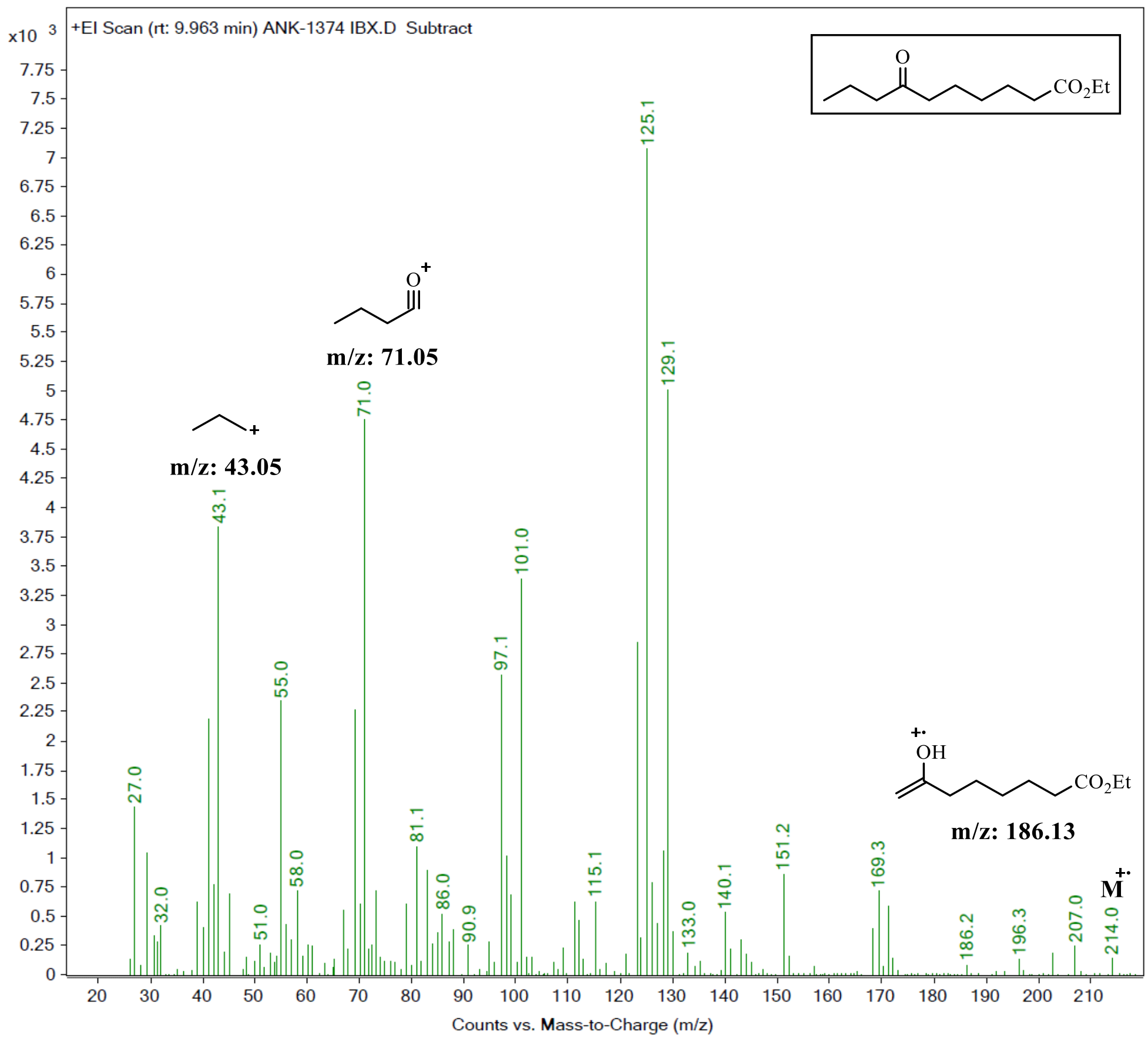
+EI Scan (rt: 9.963 min) ANK-1374 IBX.D Subtract
x10 3
m/z: 71.05
m/z: 43.05
m/z: 186.13
Counts vs. Mass-to-Charge (m/z)

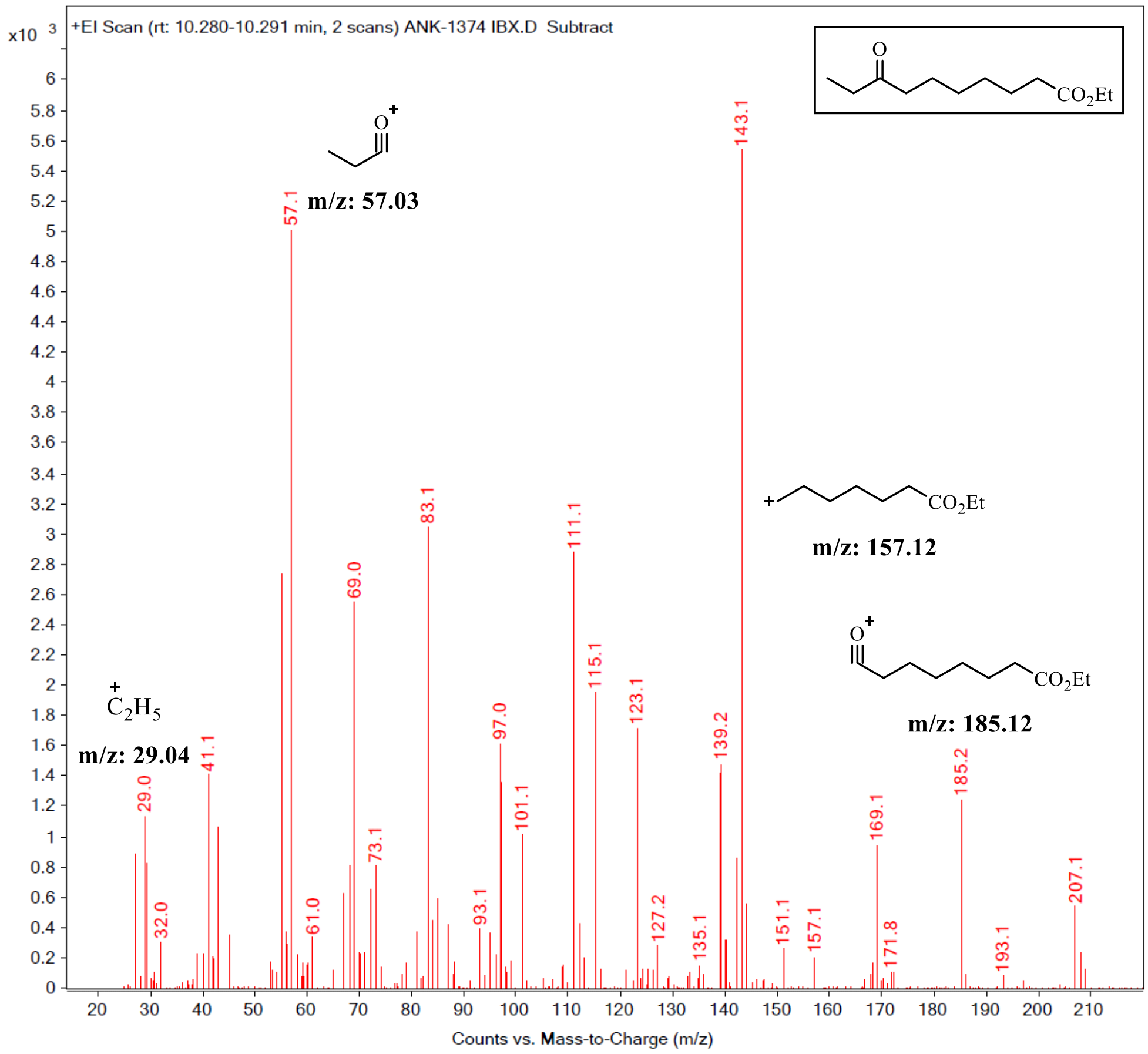

x10 3
+EI Scan (rt: 10.280-10.291 min, 2 scans) ANK-1374 IBX.D Subtract
CO2Et
m/z: 57.03
C2H5
m/z: 29.04
CO2Et
m/z: 157.12
CO2Et
m/z: 185.12
57.1
143.1
83.1
111.1
69.0
115.1
123.1
97.0
139.2
41.1
185.2
29.0
101.1
169.1
73.1
207.1
61.0
93.1
32.0
127.2
151.1
157.1
135.1
171.8
193.1
Counts vs. Mass-to-Charge (m/z)

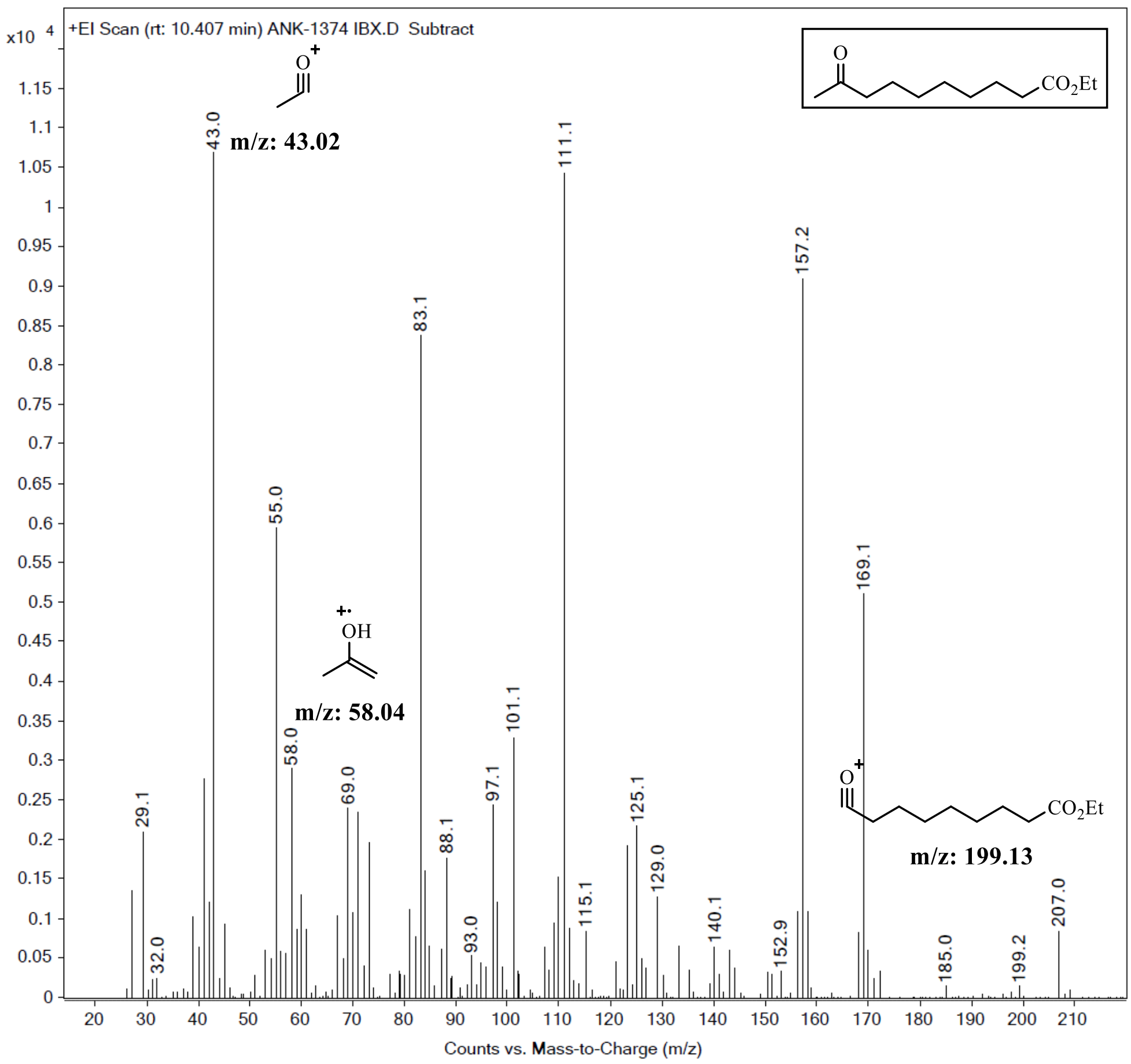

x10 4
+EI Scan (rt: 10.407 min) ANK-1374 IBX.D Subtract
CO2Et
m/z: 43.02
43.0
111.1
157.2
83.1
55.0
169.1
OH
m/z: 58.04
101.1
58.0
97.1
69.0
29.1
125.1
88.1
129.0
CO2Et
m/z: 199.13
115.1
140.1
207.0
93.0
152.9
32.0
185.0
199.2
Counts vs. Mass-to-Charge (m/z)

Tridecan-2-one (S9) oxidation

The oxidation was performed using tridecan-2-one (3.67 mg).
The following temperature-program was used: Rtx-5; 60 °C for 3 min, 25 °C/min to 165 °C, 0.5 °C/min to 175 °C, 25 °C/min to 250 °C and 250 °C for 5 min.

GC analysis of the **Mn(mcp)** catalyzed oxidation of tridecan-2-one:

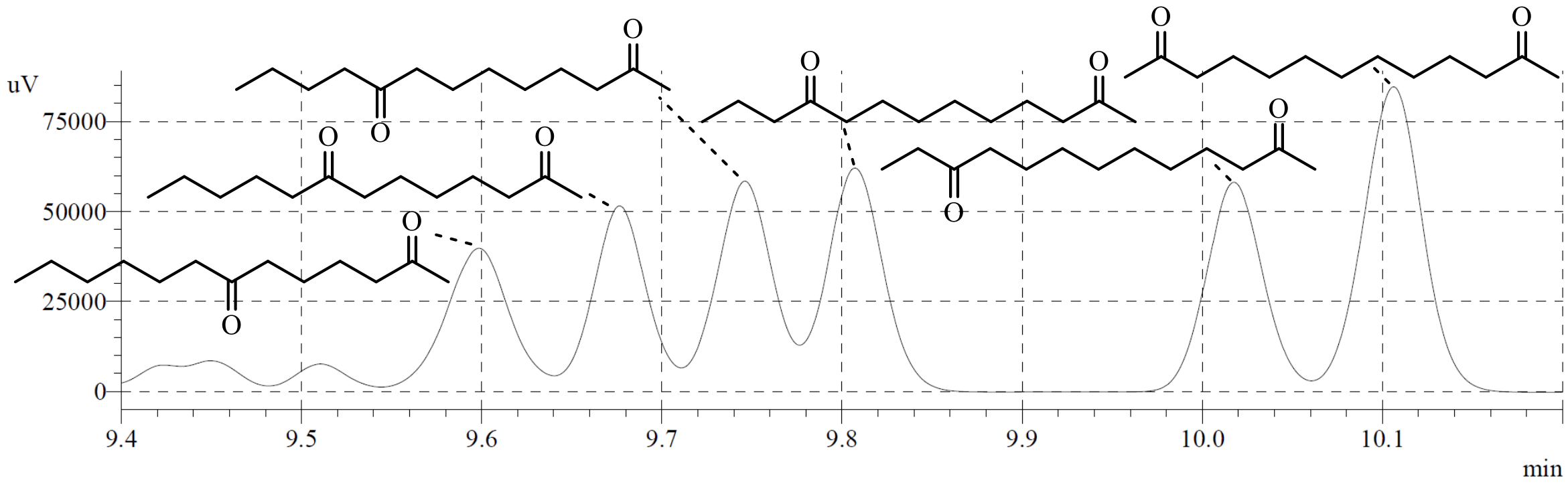

RRF(substrate): 1.00 RRF(ketone): 0.917

i	Retention time, min	Area (A)	Percentage (P), %	Percentage with RRF (P^{RRF}), %	Selectivity, %
-	7.997 (substrate)	520931	32.485	32.5	
7	9.598	94121	5.8693	6.4	12.0
6	9.677	104162	6.4955	7.1	13.3
5	9.746	124312	7.7520	8.4	15.8
4	9.807	134555	8.3908	9.2	17.2
3	10.018	130189	8.1185	8.8	16.6
2	10.106	196738	12.268	13.4	25.1
	7.299 (biphenyl)	320721	TON = 53; TOF = $5.9\cdot10^{-2}$ s^{-1}		
	Conversion: 67.5		Total product yield: 53.3		

GC analysis of the **5**-catalyzed oxidation of tridecan-2-one:

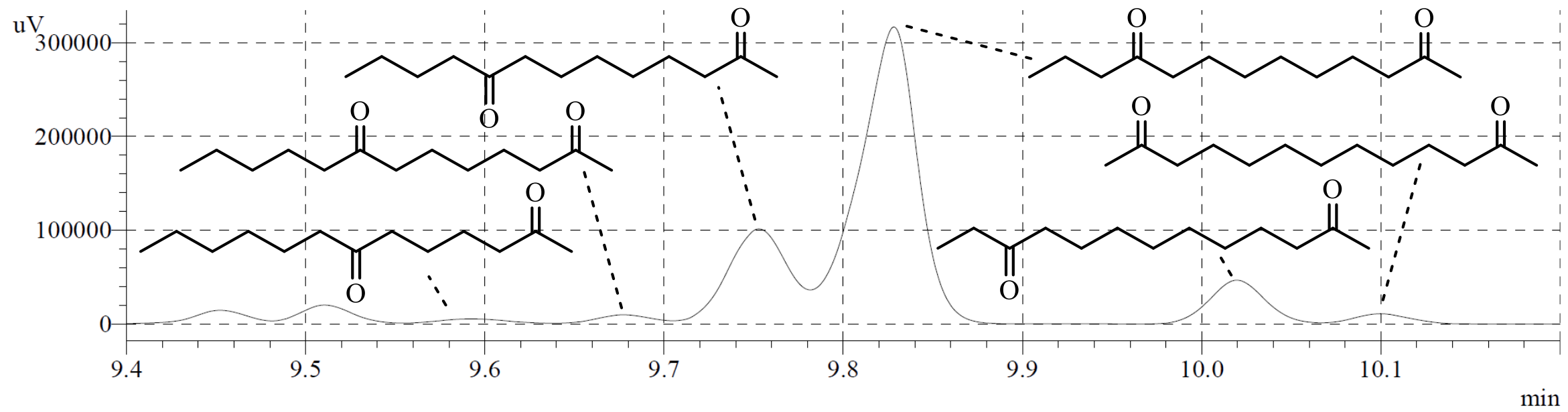

RRF(substrate): 1.00 RRF(ketone): 0.917

i	Retention time, min	Area (A)	Percentage (P), %	Percentage with RRF (P^{RRF}), %	Selectivity, %
-	7.993 (substrate)	66650	4.0017	4.0	
7	9.593	10808	0.64892	0.7	1.0
6	9.678	19063	1.1446	1.2	1.7
5	9.753	239747	14.394	15.7	21.4
4	9.828	724577	43.504	47.4	64.6
3	10.02	102716	6.1671	6.7	9.2
2	10.1	24387	1.4642	1.6	2.2
	7.299 (biphenyl)	333109	TON = 37; TOF = $4.1\cdot10^{-2}$ s^{-1}		
	Conversion: 96.0		Total product yield: 73.3		

GC-MS analyses of the **Mn(mcp)** (top) and **5**-catalyzed (bottom) oxidation of tridecan-2-one. The different oxidation products were assigned according to their fragmentation pattern.

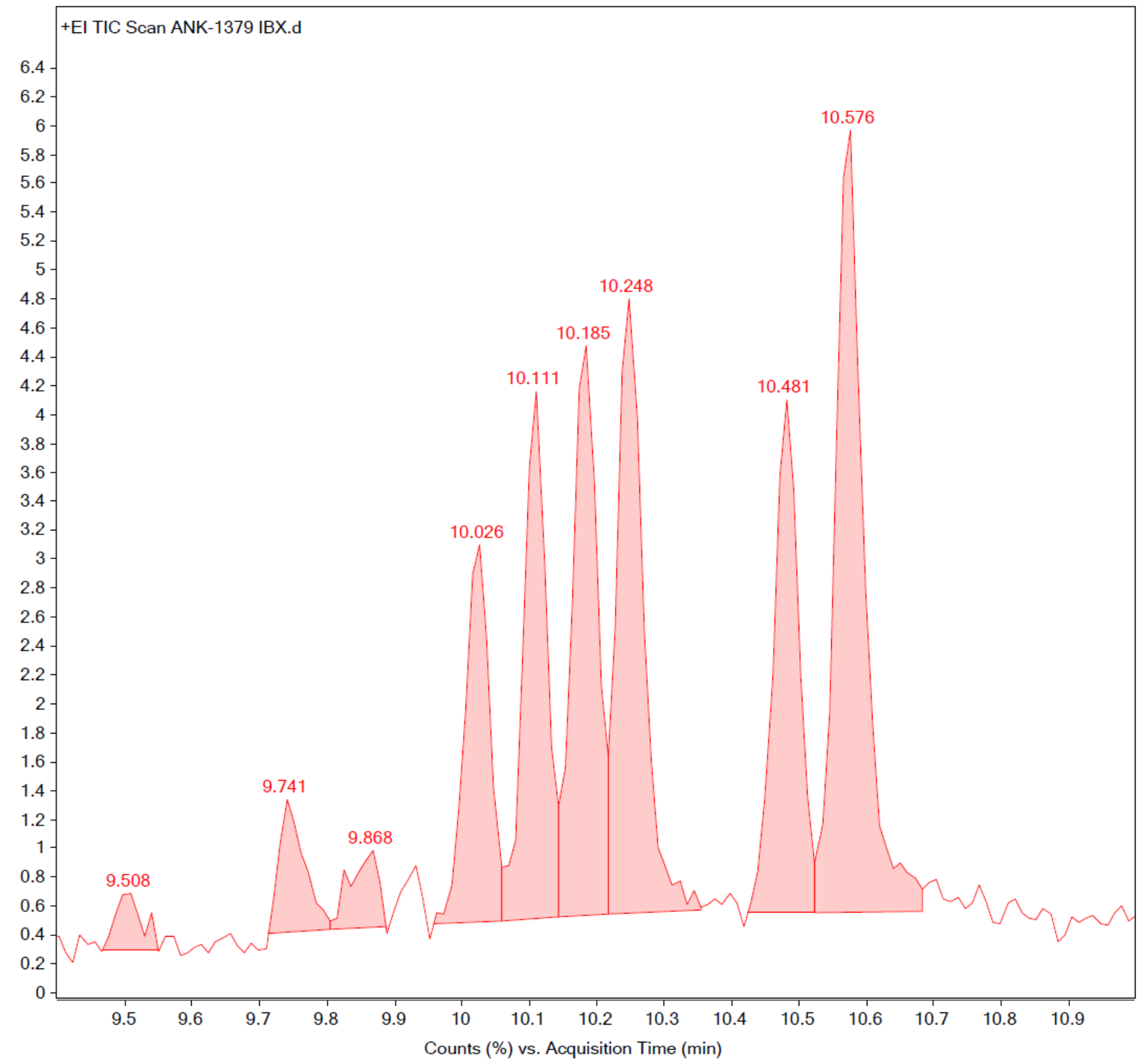

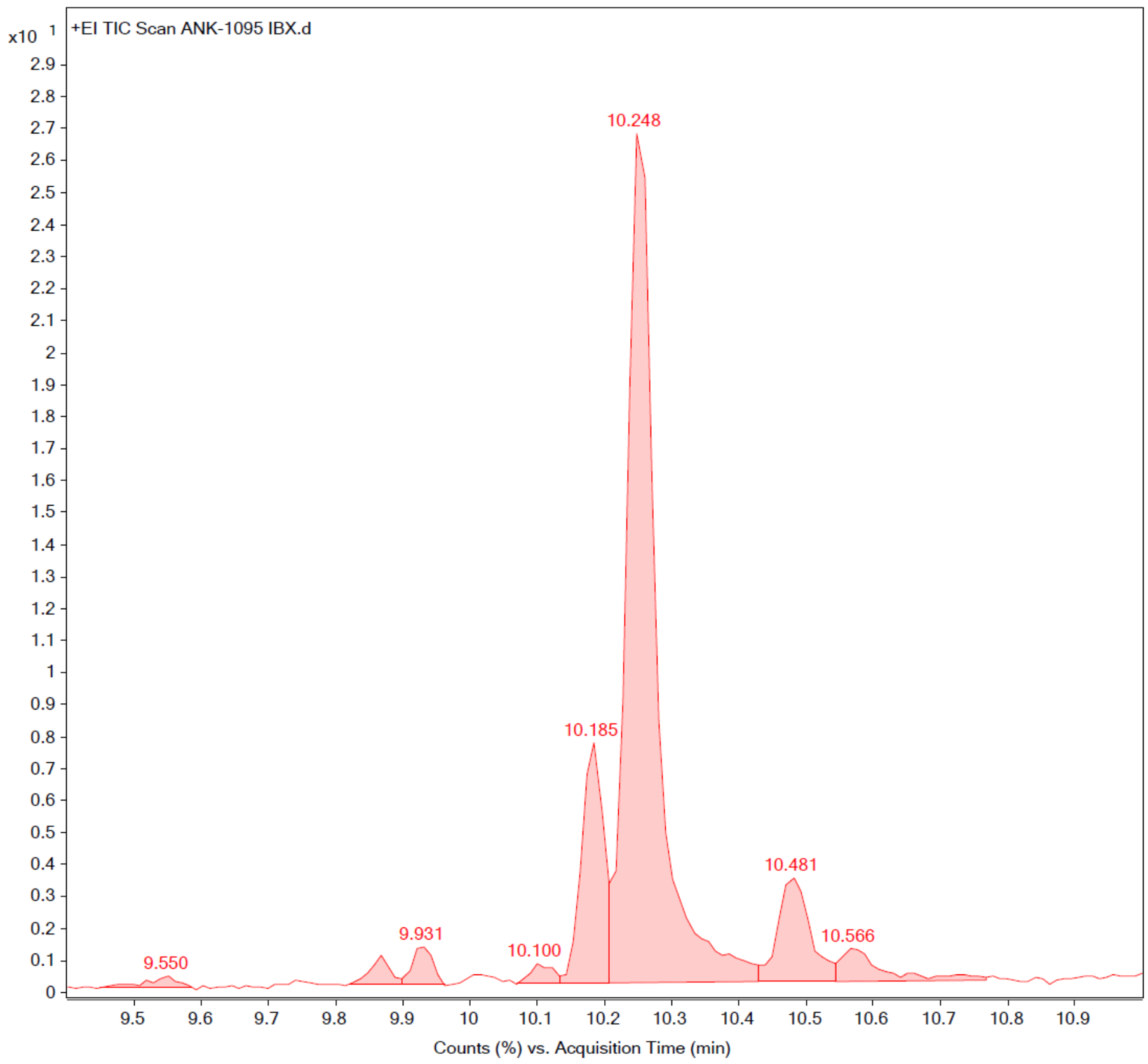

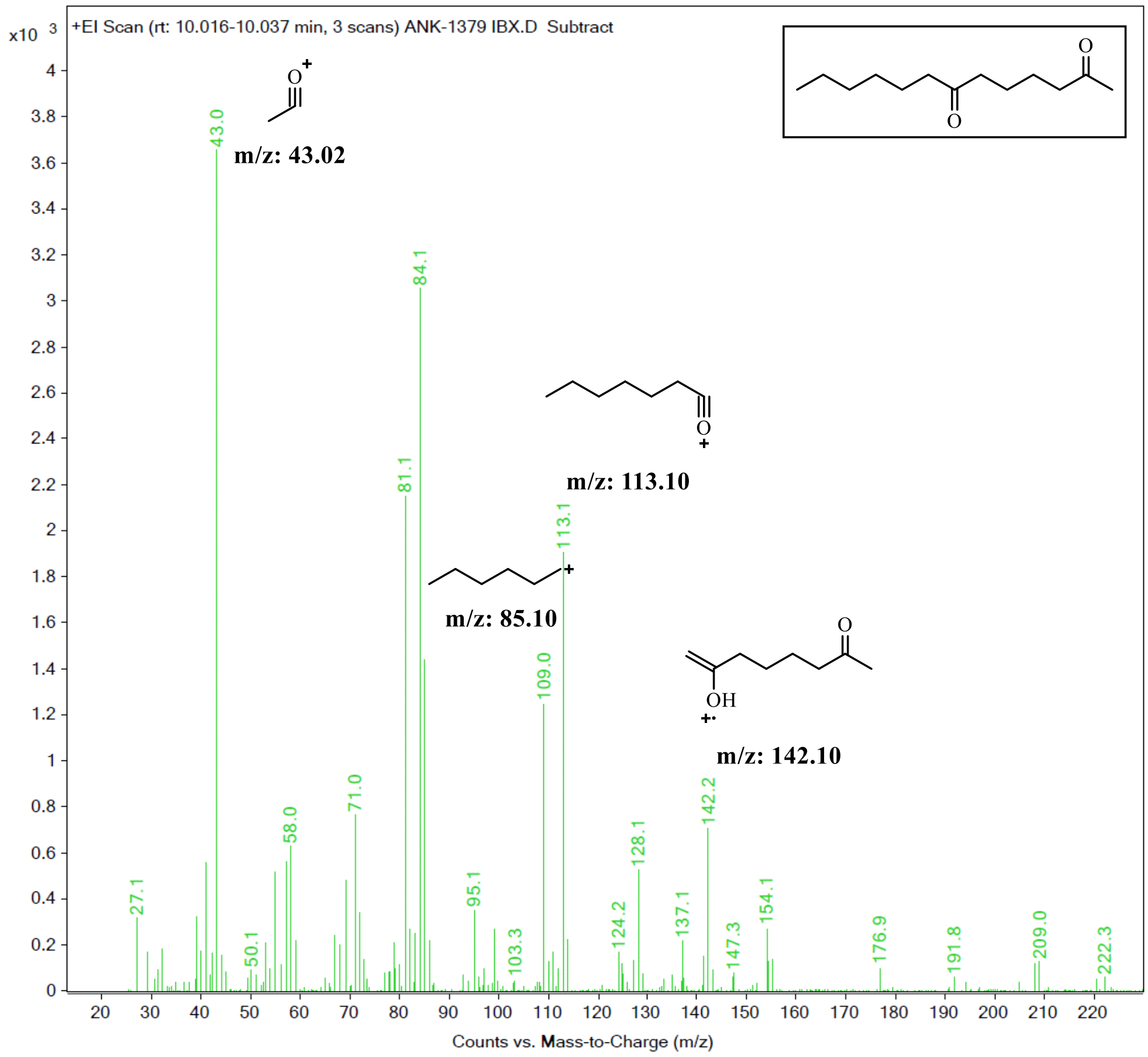

x10 3
+EI Scan (rt: 10.016-10.037 min, 3 scans) ANK-1379 IBX.D Subtract
m/z: 43.02
m/z: 113.10
m/z: 85.10
m/z: 142.10
OH
O
Counts vs. Mass-to-Charge (m/z)

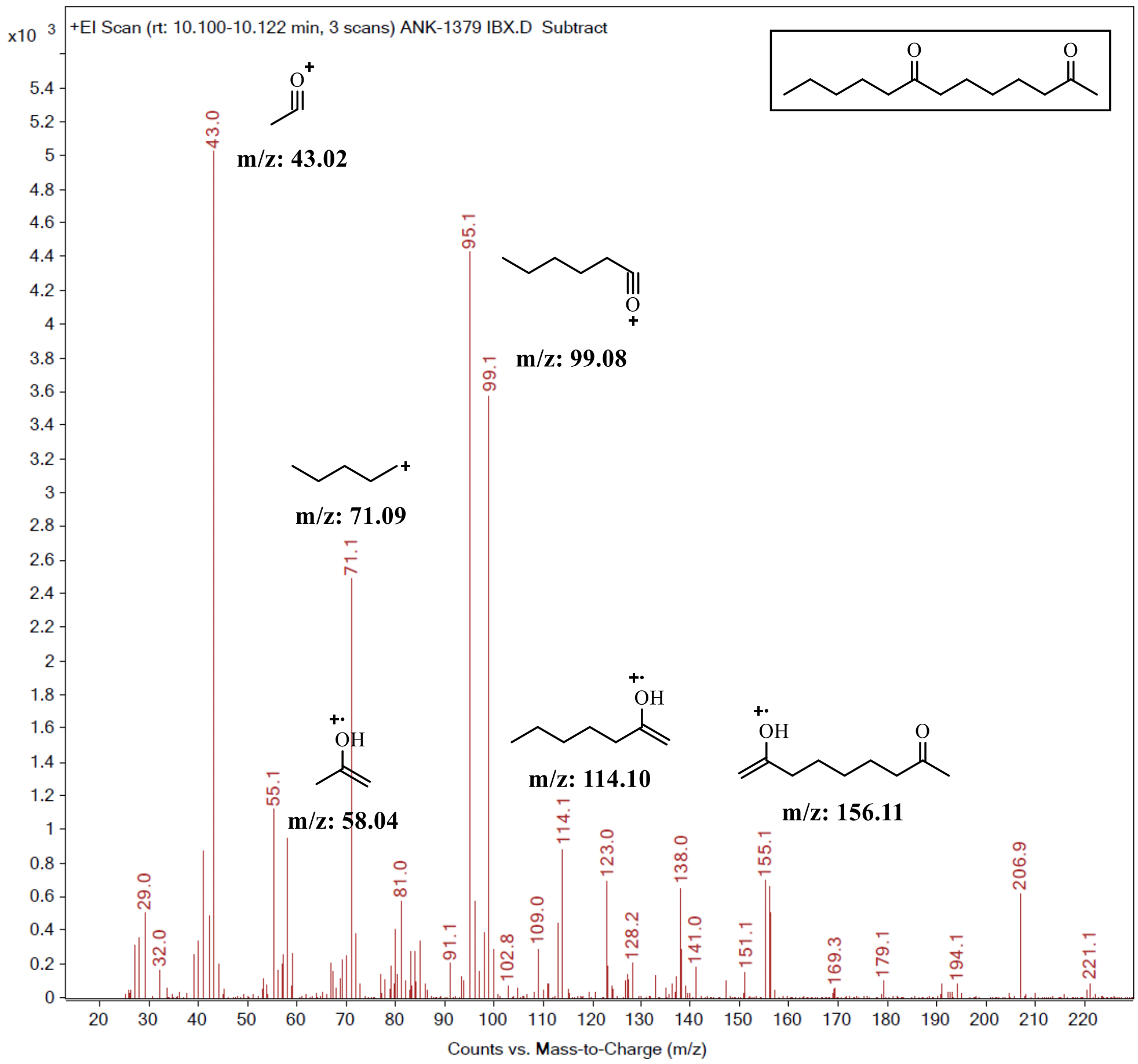

x10 3
+EI Scan (rt: 10.100-10.122 min, 3 scans) ANK-1379 IBX.D Subtract
m/z: 43.02
m/z: 99.08
m/z: 71.09
m/z: 58.04
m/z: 114.10
m/z: 156.11
OH
O
Counts vs. Mass-to-Charge (m/z)

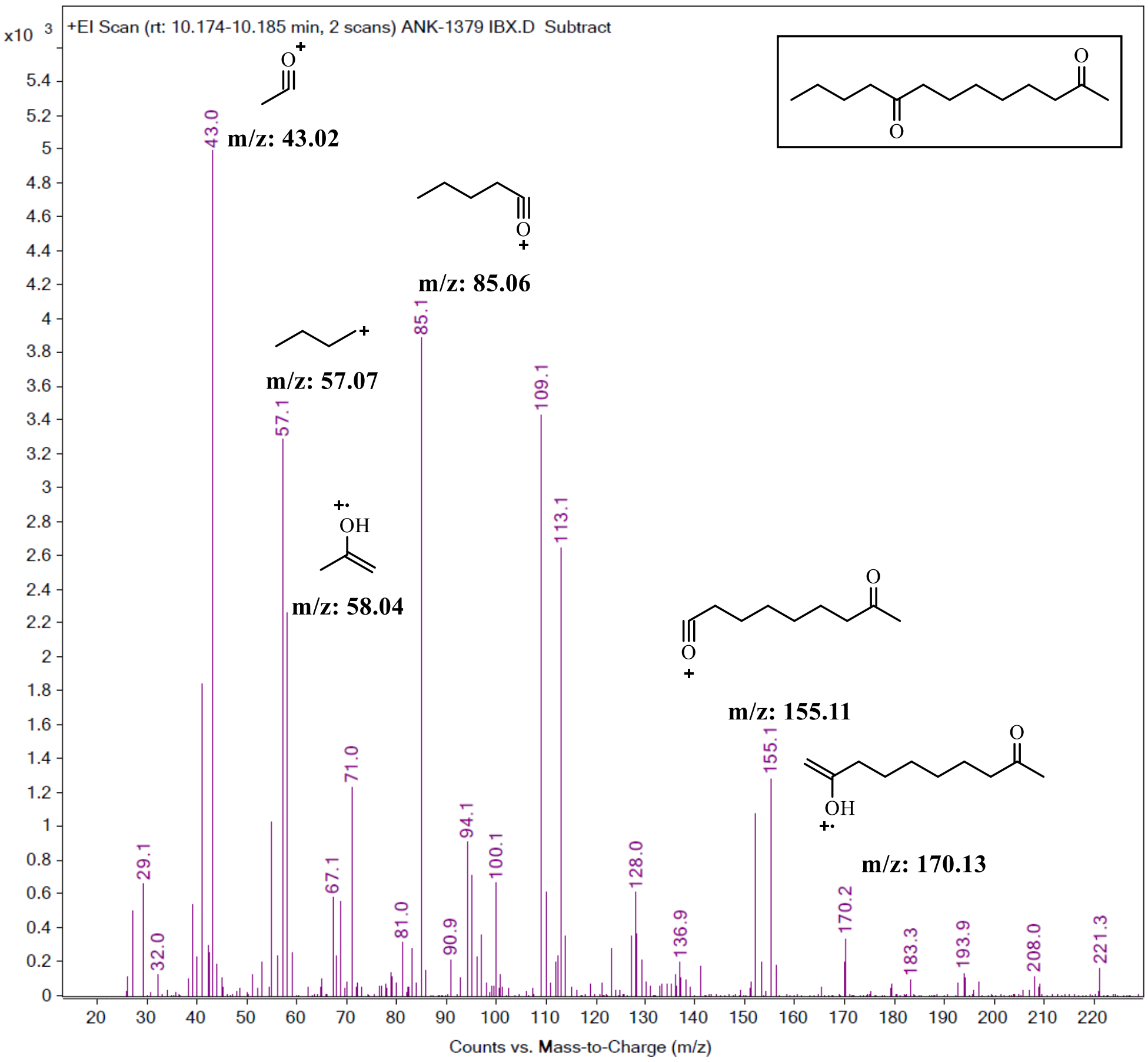

+EI Scan (rt: 10.174-10.185 min, 2 scans) ANK-1379 IBX.D Subtract
m/z: 43.02
m/z: 85.06
m/z: 57.07
m/z: 58.04
m/z: 155.11
m/z: 170.13
Counts vs. Mass-to-Charge (m/z)

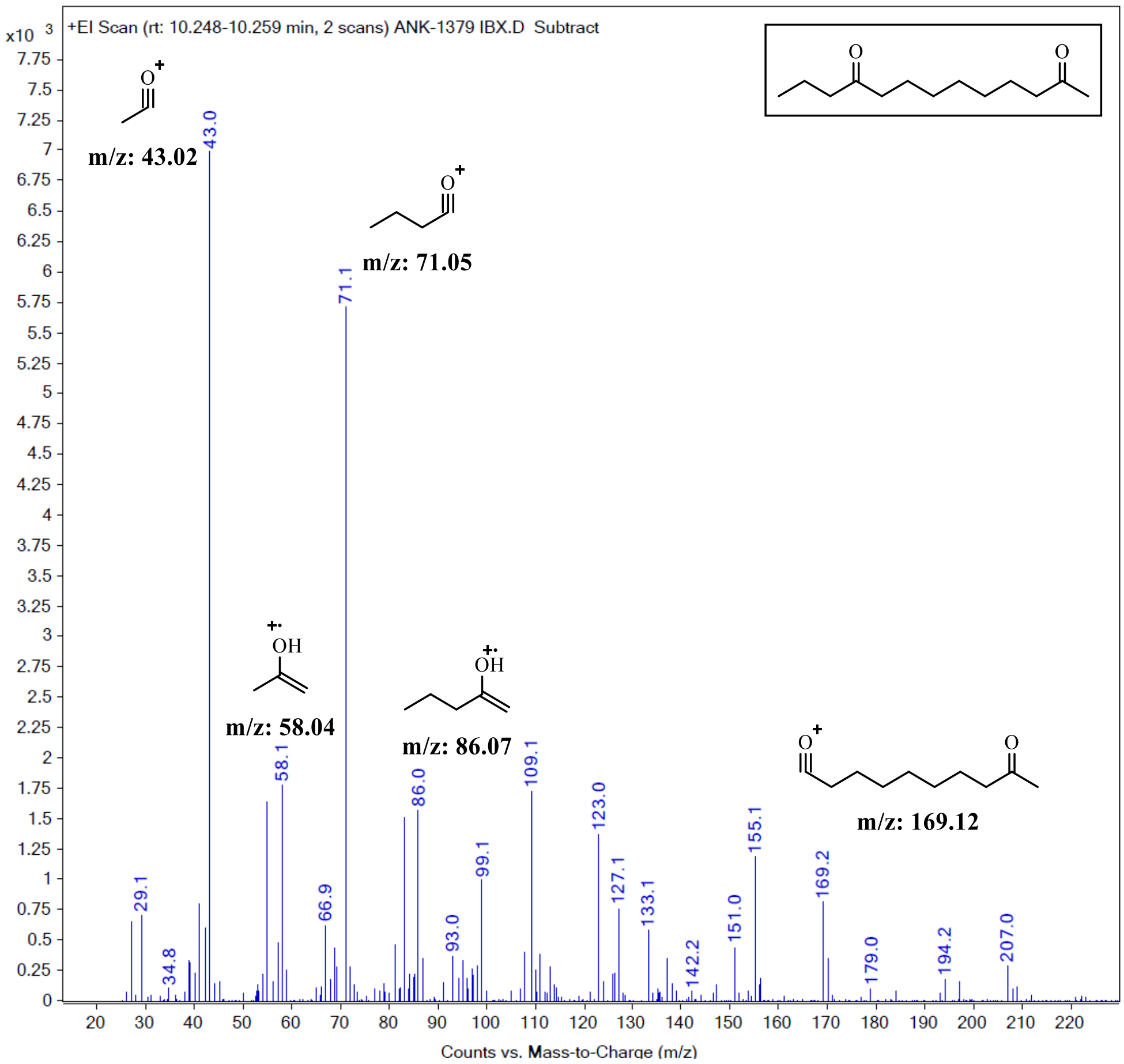

+EI Scan (rt: 10.248-10.259 min, 2 scans) ANK-1379 IBX.D Subtract
m/z: 43.02
m/z: 71.05
m/z: 58.04
m/z: 86.07
m/z: 169.12
Counts vs. Mass-to-Charge (m/z)

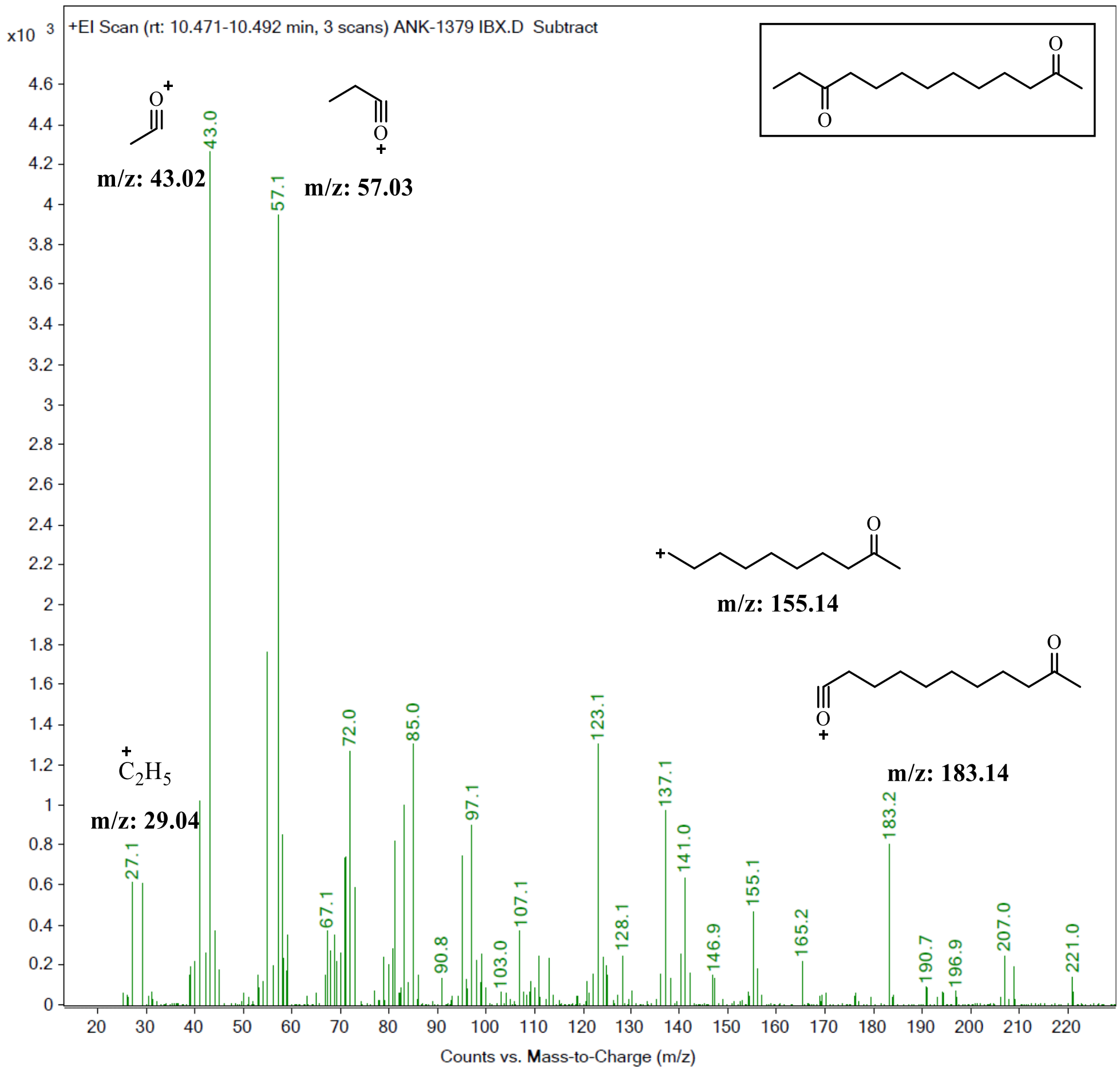
+EI Scan (rt: 10.471-10.492 min, 3 scans) ANK-1379 IBX.D Subtract
x10 3
m/z: 43.02
m/z: 57.03
m/z: 29.04
m/z: 155.14
m/z: 183.14
43.0
57.1
27.1
67.1
72.0
85.0
90.8
97.1
103.0
107.1
123.1
128.1
137.1
141.0
146.9
155.1
165.2
183.2
190.7
196.9
207.0
221.0
Counts vs. Mass-to-Charge (m/z)

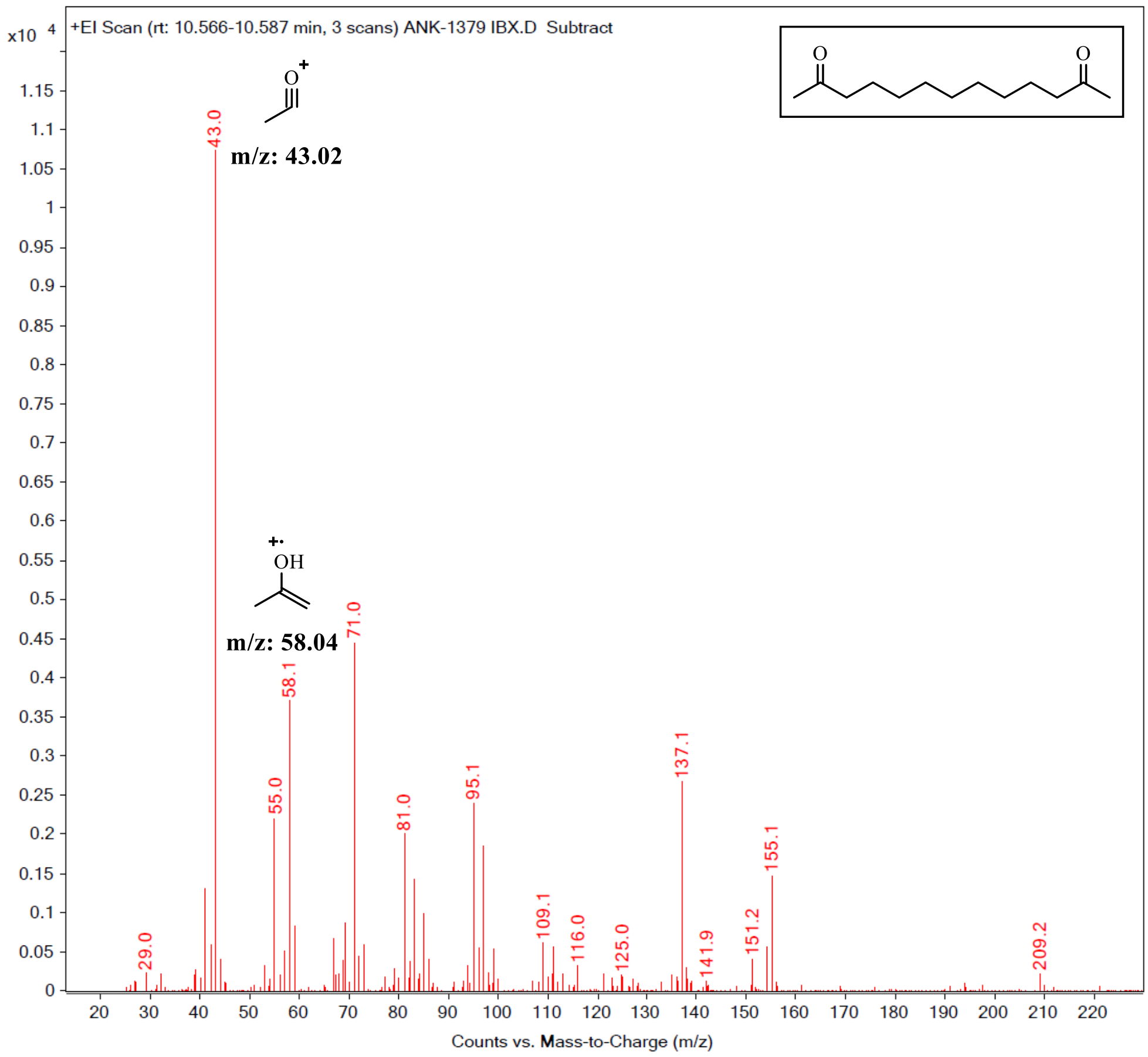
+EI Scan (rt: 10.566-10.587 min, 3 scans) ANK-1379 IBX.D Subtract
x10 4
m/z: 43.02
m/z: 58.04
43.0
29.0
55.0
58.1
71.0
81.0
95.1
109.1
116.0
125.0
137.1
141.9
151.2
155.1
209.2
Counts vs. Mass-to-Charge (m/z)

2-Decylisoindoline-1,3-dione (S10) oxidation

The oxidation was performed using 2-decylisoindoline-1,3-dione (5.32 mg).
The following temperature-program was used: Rtx-5; 60 °C for 3 min, 15 °C/min to 250 °C and 250 °C for 5 min.

GC analysis of the **Mn(mcp)** catalyzed oxidation of 2-decylisoindoline-1,3-dione:

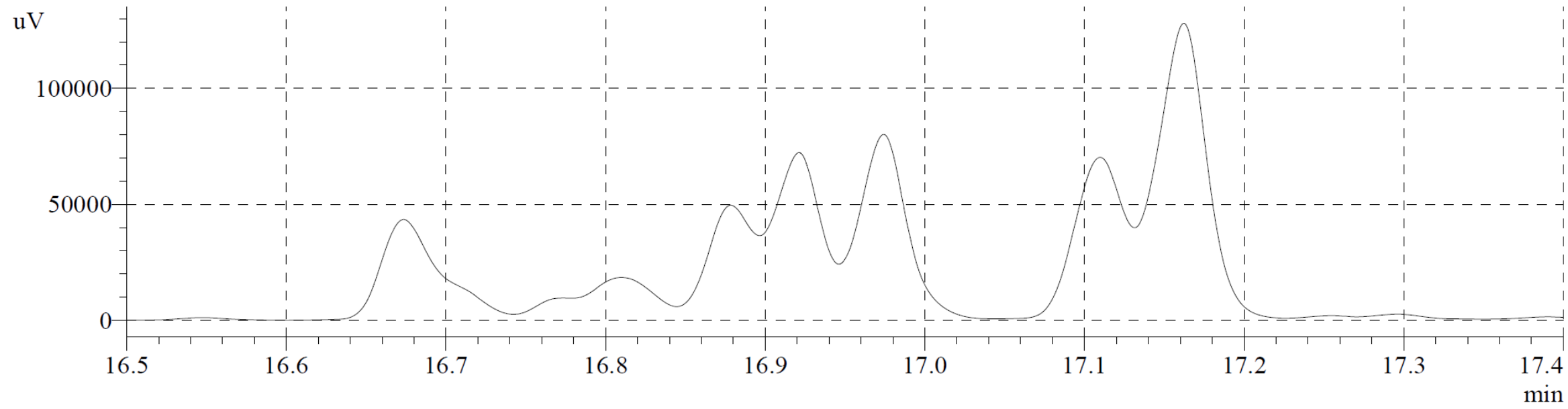

RRF(substrate): 1.33 RRF(ketone): 1.25

i	Retention time, min	Area (A)	Percentage (P), %	Percentage with RRF (P^{RRF}), %	Selectivity, %
-	15.606 (substrate)	579434	37.798	28.3	
-	sum of peaks 16.612-17.230	1016434	66.304	53.0	
4	16.974	168944	11.020	8.8	16.6
	9.030 (biphenyl)	306599	TON = 53; TOF = $5.9 \cdot 10^{-2}$ s^{-1}		
	Conversion: 71.7		Total product yield: 53.0		

GC analysis of the **5**-catalyzed oxidation of 2-decylisoindoline-1,3-dione:

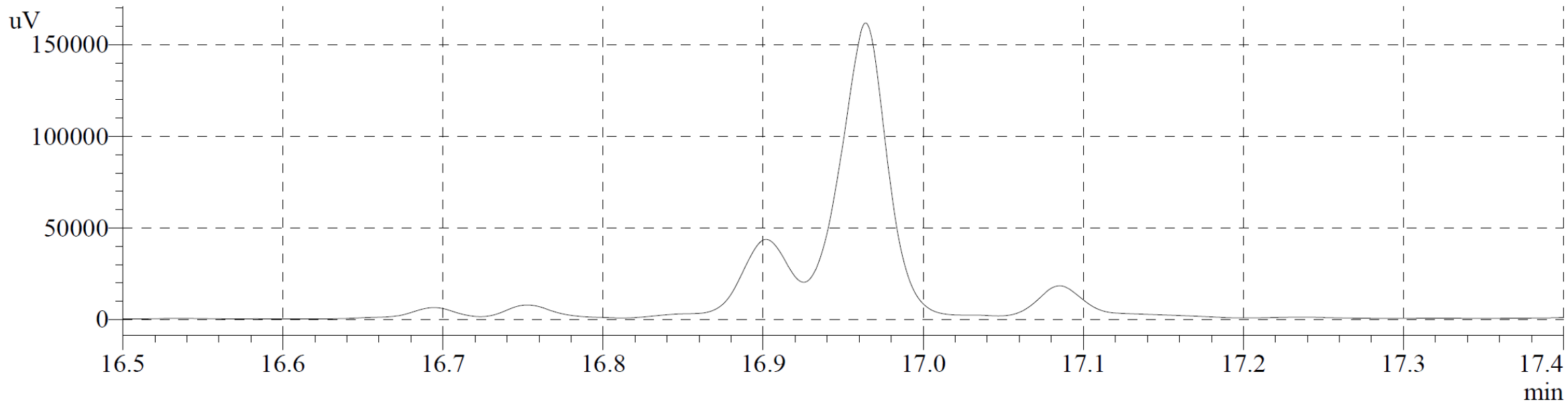

RRF(substrate): 1.33 RRF(ketone): 1.25

i	Retention time, min	Area (A)	Percentage (P), %	Percentage with RRF (P^{RRF}), %	Selectivity, %
-	15.589 (substrate)	102224	7.2692	5.4	
-	sum of peaks 16.624-17.198	1114532	79.255	63.4	
4	16.979	739209	52.565	42.0	66.3
	9.018 (biphenyl)	281253	TON = 32; TOF = $3.5 \cdot 10^{-2}$ s^{-1}		
	Conversion: 94.6		Total product yield: 63.4		

The main oxidation product of the **5**-catalyzed oxidation was isolated and characterized following a scaled-up synthesis.

Scaled up-synthesis (×10)
TFE (1.70 mL), substrate **S10** (53.2 mg, 185 µmol), 2,2-dimethylpropanoic acid (18.9 mg, 185 µmol) and 200 µL of TfOH solution in TFE (2.5 mol% of TfOH in relation to the substrate) were added to an oven-dried 12 mL vial charged with catalyst **5** (2.79 mg, 1.85 µmol) and a stirring bar. The mixture was cooled to 0 °C. A solution of an 50% aqueous H_2O_2 diluted in TFE (370 µL; stock solution: 110 µL 50 % aqueous H_2O_2 in 1.90 mL TFE) was slowly added over 30 minutes (12.3 µL/min) under stirring via a syringe pump. After the addition, the mixture was left stirring for another 30 min.

Work-up and IBX Oxidation: after the time indicated, H_2O (6.0 mL) was added and the mixture was extracted with HPLC grade EtOAc (2.0 mL × 3), washed with sat. $NaHCO_3$ (2.5 mL × 2), filtered through a silica plug (glass filter filled with 6.5 g of silica gel and 6.0 g of Na_2SO_4 on top) into a 100 mL one-neck round-bottom flask to remove catalyst **5** and rinsed with EtOAc (30 mL). Organic solvents were removed *in vacuo*, EtOAc (30 mL) was added and the flask was equipped with a stirring bar. IBX (124 mg, 444 µmol) was added and the mixture was heated to 66 °C (oil bath temperature) overnight. The mixture was allowed to cool down to room temperature, filtered through a silica plug (glass filter filled with 7.0 g of silica gel) into a 100 mL one-neck round-bottom flask to remove excess of IBX and byproducts and rinsed with EtOAc (20 mL). Organic solvents were evaporated to give crude material.

Isolation of the product: the product was isolated by preparative HPLC using the Reprospher 100 Si (particle size: 5 µm; dimensions: 250 × 25 mm) Dr. Maisch column. The crude was dissolved in 1 mL cyclohexane, filtered and eluted by cyclohexane/EtOAc using isocratic program with 9% EtOAc (flow rate: 15 mL/min, λ = 300 nm). Obtained fractions were checked by GC, in order to identify pure fractions of C4-oxidized compound **S10**. The mixed fractions after the HPLC first run containing the desired product were combined and subjected to HPLC one more time. That gave the C4-oxidized compound **S10** as white solid.

GC analysis of the pure isolated product:

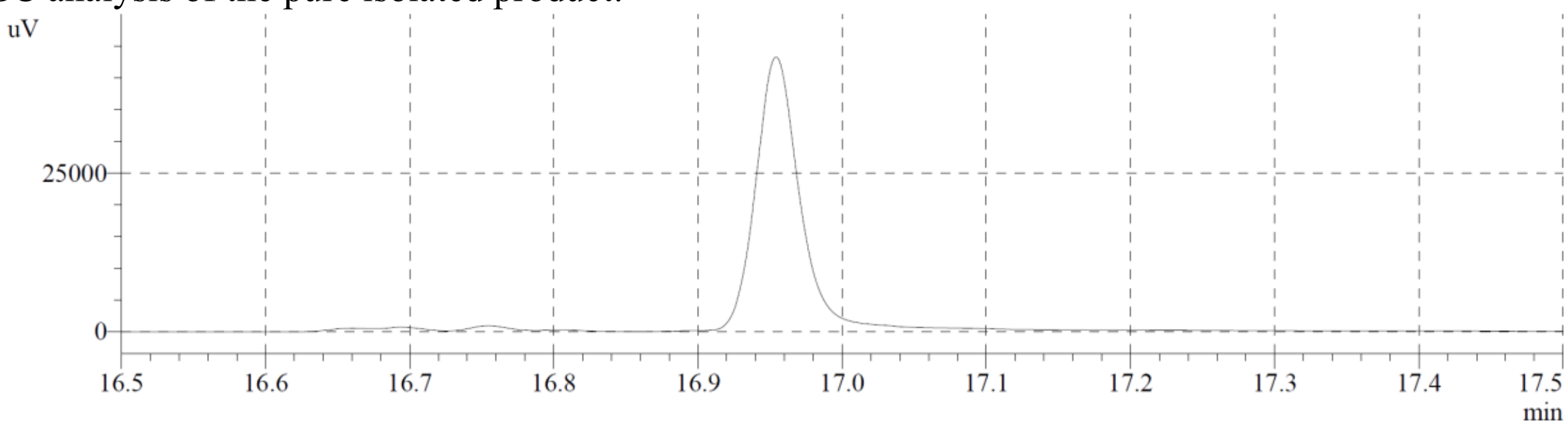

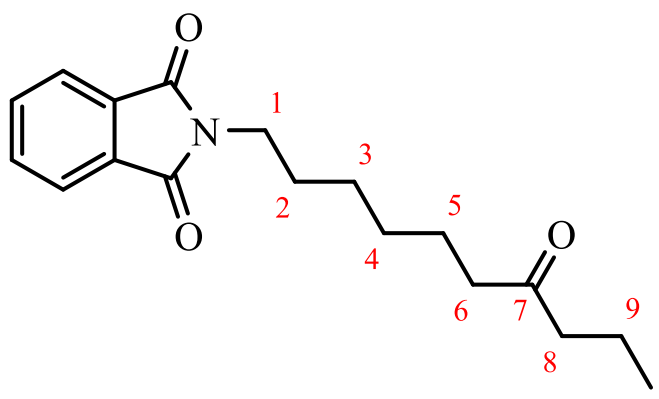

Chemical Formula: $C_{18}H_{23}NO_3$
Molecular Weight: 301.39

Yield: 20 mg, 36% (95% GC purity).

Mp.: 38 – 39 °C.

IR (ID7, cm^{-1}) 2931, 2860, 1772, 1701, 1463, 1433, 1397, 1366, 1036, 960, 886, 792, 717, 624, 530.

^{1}H NMR (500 MHz, $CDCl_3$) δ 7.84 (dd, ^3J = 5.4, ^4J = 3.0 Hz, 2H, 2×CH, Ar), 7.71 (dd, ^3J = 5.4, ^4J = 3.0 Hz, 2H, 2×CH, Ar), 3.67 (t, ^3J = 7.3 Hz, 2H, >NCH_2), 2.38 (t, ^3J = 7.5 Hz, 2H, C(6)H_2), 2.36 (t, ^3J = 7.5 Hz, 2H, C(8)H_2), 1.69 – 1.63 (m, 2H, C(2)H_2), 1.62 – 1.52 (m, 4H, C(5)H_2 + C(9)H_2), 1.38 – 1.28 (m, 4H, C(3)H_2 + C(4)H_2), 0.90 (t, ^3J = 7.4 Hz, 3H, Me).

^{13}C NMR (125 MHz, $CDCl_3$) δ 211.3 (C(7)=O), 168.5 (2×C=O), 133.9 (2×C, Ar), 132.1 (2×CH, Ar), 123.2 (2×CH, Ar), 44.7 (C(8)H_2), 42.6 (C(6)H_2), 37.9 (C(1)H_2), 28.8 (C(4)H_2), 28.4 (C(2)H_2), 26.6 (C(3)H_2), 23.6 (C(5)H_2), 17.3 (C(9)H_2), 13.8 (Me).

HRMS (ESI$^+$) *m/z* calcd for $C_{18}H_{23}NO_3Na$ $[M + Na]^+$: 324.1570; found 324.1576.

N-Decyl-4-methylbenzenesulfonamide (S11) oxidation

The oxidation was performed using *N*-decyl-4-methylbenzenesulfonamide (5.76 mg).
The following temperature-program was used: Rtx-5; 60 °C for 3 min, 15 °C/min to 250 °C and 250 °C for 5 min.

GC analysis of the **Mn(mcp)** catalyzed oxidation of *N*-decyl-4-methylbenzenesulfonamide:

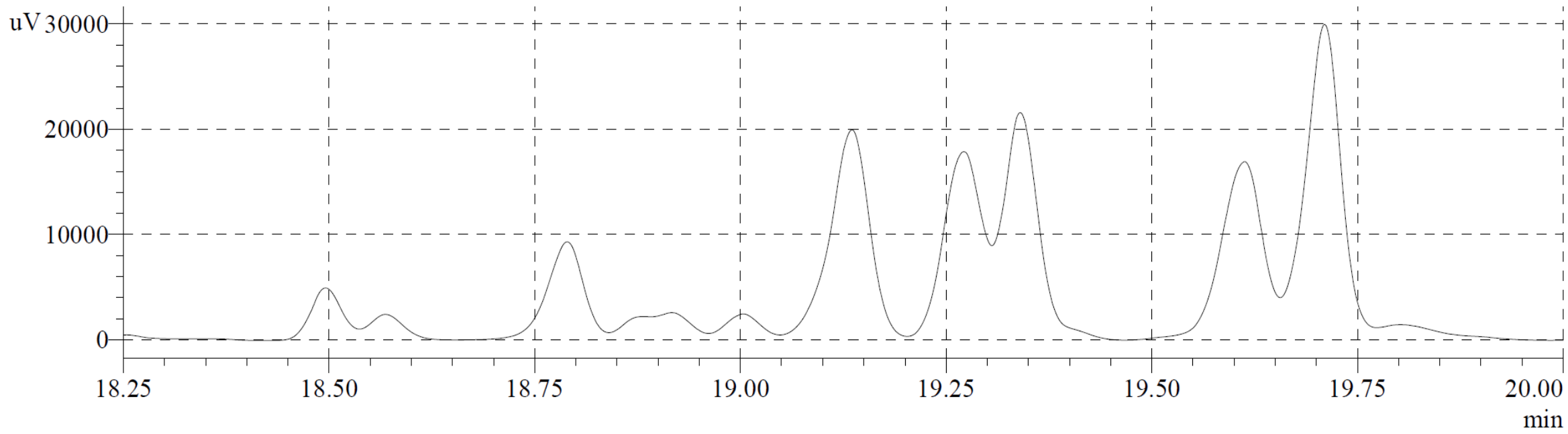

RRF(substrate): 1.417 RRF(ketone): 1.333

i	Retention time, min	Area (A)	Percentage (P), %	Percentage with RRF (P^{RRF}), %	Selectivity, %
-	17.268 (substrate)	880886	56.850	40.1	
-	sum of peaks 18.441-19.776	425590	27.466	20.6	
4	19.340	65307	4.2147	3.2	15.4
	9.030 (biphenyl)	309898	TON = 21; TOF = $2.3\cdot10^{-2}$ s^{-1}		
	Conversion: 59.9		Total product yield: 20.6		

GC analysis of the **5**-catalyzed oxidation of *N*-decyl-4-methylbenzenesulfonamide:

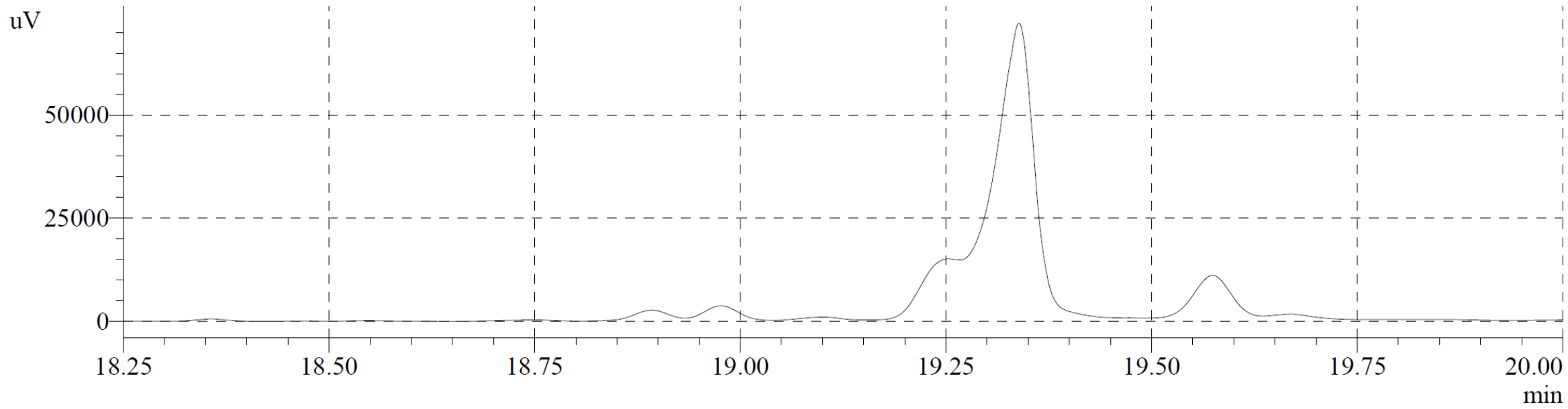

RRF(substrate): 1.417 RRF(ketone): 1.333

i	Retention time, min	Area (A)	Percentage (P), %	Percentage with RRF (P^{RRF}), %	Selectivity, %
-	17.230 (substrate)	410649	28.268	20.0	
-	sum of peaks 18.815-19.789	941614	64.819	48.6	
4	19.340	631321	43.459	32.6	67.0
	9.017 (biphenyl)	290537	TON = 24; TOF = $2.7\cdot10^{-2}$ s^{-1}		
	Conversion: 80.0		Total product yield: 48.6		

The main oxidation product of the **5**-catalyzed oxidation was isolated and characterized following a scaled-up synthesis.

Scaled-up synthesis (×10)
TFE (1.70 mL), substrate **S11** (57.6 mg, 185 µmol), 2,2-dimethylpropanoic acid (18.9 mg, 185 µmol) and 200 µL of TfOH solution in TFE (2.5 mol% of TfOH in relation to the substrate) were added to an oven-dried 12 mL vial charged with catalyst **5** (2.79 mg, 1.85 µmol) and a stirring bar. The mixture was cooled to 0 °C. A solution of an 50% aqueous H_2O_2 diluted in TFE (370 µL; stock solution: 110 µL 50 % aqueous H_2O_2 in 1.90 mL TFE) was slowly added over 30 minutes (12.3 µL/min) under stirring via a syringe pump. After the addition, the mixture was left stirring for another 30 min.

Work-up and IBX Oxidation: after the time indicated, H_2O (6.0 mL) was added and the mixture was extracted with HPLC grade EtOAc (2.0 mL × 3), washed with sat. $NaHCO_3$ (2.5 mL × 2), filtered through a silica plug (glass filter filled with 6.5 g of silica gel and 6.0 g of Na_2SO_4 on top) into a 100 mL one-neck round-bottom flask to remove catalyst **5** and rinsed with EtOAc (30 mL). Organic solvents were removed *in vacuo*, EtOAc (30 mL) was added and the flask was equipped with a stirring bar. IBX (124 mg, 444 µmol) was added and the mixture was heated to 66 °C (oil bath temperature) overnight. The mixture was allowed to cool down to room temperature, filtered through a silica plug (glass filter filled with 7.0 g of silica gel) into a 100 mL one-neck round-bottom flask to remove excess of IBX and byproducts and rinsed with EtOAc (20 mL). Organic solvents were evaporated to give crude material.

Isolation of the product: the product was isolated by preparative HPLC using the Reprospher 100 Si (particle size: 5 µm; dimensions: 250 × 25 mm) Dr. Maisch column. The crude was dissolved in 1.0 mL cyclohexane and 50 µL DCM, filtered and eluted by cyclohexane/EtOH using isocratic program with 1% EtOH (flow rate: 15 mL/min, λ = 220 nm). Obtained fractions were checked by GC, in order to identify pure fractions of C4-oxidized compound **S11**. The mixed fractions after the HPLC first run containing the desired product were combined and subjected to HPLC one more time. That gave the C4-oxidized compound **S11** as white solid.

GC analysis of the pure isolated product:

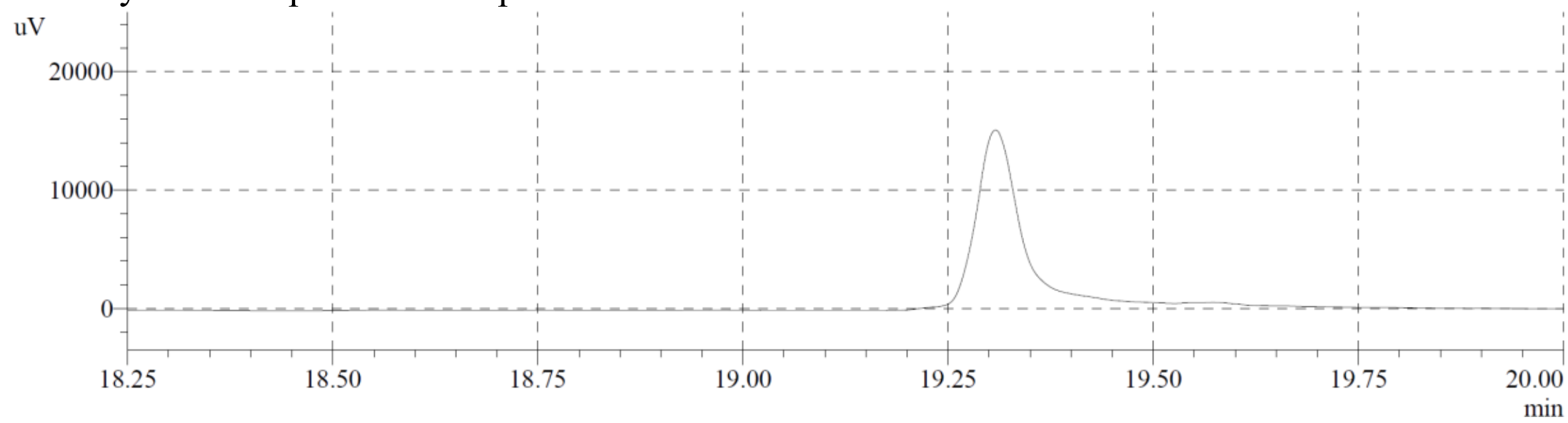

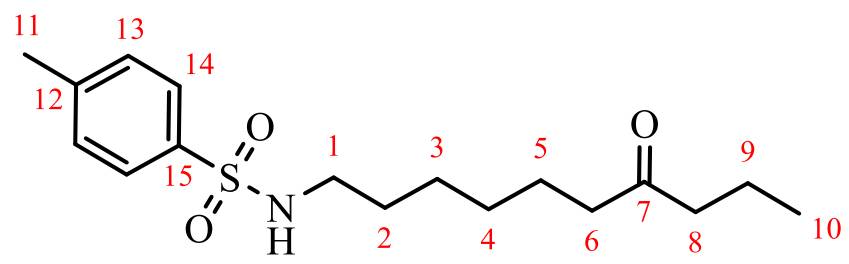

Chemical Formula: $C_{17}H_{27}NO_3S$
Molecular Weight: 325.47

Yield: 15 mg, 25% (99% GC purity).

Mp.: 62 – 63 °C.

IR (ID7, cm^{-1}) 3278 (m), 2928 (m), 2865 (w), 1703 (m), 1595 (w), 1465 (w), 1418 (w), 1377 (w), 1317 (m), 1216 (w), 1153 (s), 1089 (w), 1062 (m), 997 (w), 898 (w), 812 (m), 723 (w), 668 (s), 569 (m), 551 (s), 516 (m), 491 (w).

^{1}H NMR (500 MHz, $CDCl_3$) δ 7.74 (d, ^3J = 8.2 Hz, 2H, 2×C(14)H, Ar), 7.30 (d, ^3J = 8.2 Hz, 2H, 2×C(13)H, Ar), 4.53 (br t, ^3J = 6.2 Hz, 1H, NH), 2.42 (s, 3H, Me(11)), 2.91 (dt, ^3J = 7.0 Hz, ^3J = 6.2 Hz, 2H, C(1)H_2), 2.35 (t, ^3J = 7.3 Hz, 2H, C(8)H_2), 2.34 (t, ^3J = 7.4 Hz, 2H, C(6)H_2), 1.62 – 1.53 (m, 2H, C(9)H_2), 1.53 – 1.47 (m, 2H, C(5)H_2), 1.47 – 1.40 (m, 2H, C(2)H_2), 1.29 – 1.23 (m, 2H, C(3)H_2), 1.23 – 1.17 (m, 2H, C(4)H_2), 0.89 (t, ^3J = 7.4 Hz, 3H, Me(10)).

^{13}C NMR (125 MHz, $CDCl_3$) δ 211.3 (C=O), 143.3 (C(12), Ar), 136.9 (C(15), Ar), 129.7 (2×C(13)H, Ar), 127.1 (2×C(14)H, Ar), 44.7 (C(8)H_2), 43.1 (C(1)H_2), 42.5 (C(6)H_2), 29.3 (C(2)H_2), 28.6 (C(4)H_2), 26.2 (C(3)H_2), 23.4 (C(5)H_2), 21.5 (Me(11)), 17.3 (C(9)H_2), 13.7 (Me(10)).

HRMS (ESI$^+$) *m/z* calcd for $C_{17}H_{28}NO_3S$ $[M + H]^+$: 326.1784; found 326.1789.

N-Decylmethanesulfonamide (S12) oxidation

The oxidation was performed using *N*-decylmethanesulfonamide (4.35 mg).
The following temperature-program was used: Rtx-5; 60 °C for 3 min, 15 °C/min to 250 °C and 250 °C for 5 min.

GC analysis of the **Mn(mcp)** catalyzed oxidation of *N*-decylmethanesulfonamide:

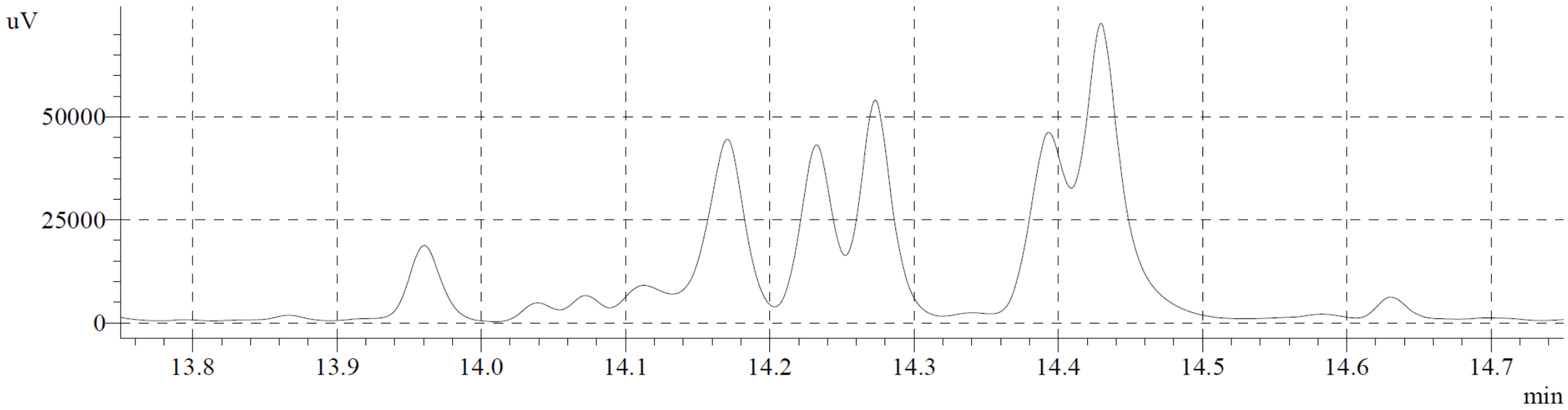

RRF(substrate): 0.917 RRF(ketone): 0.833

i	Retention time, min	Area (A)	Percentage (P), %	Percentage with RRF (P^{RRF}), %	Selectivity, %
-	13.068 (substrate)	469212	30.459	33.2	
-	sum of peaks 13.909-14.523	523970	34.013	40.8	
4	14.273	89351	5.8002	7.0	17.0
	9.030 (biphenyl)	308098	TON = 41; TOF = $4.5 \cdot 10^{-2}$ s^{-1}		
	Conversion: 66.8		Total product yield: 40.8		

GC analysis of the **5**-catalyzed oxidation of *N*-decylmethanesulfonamide:

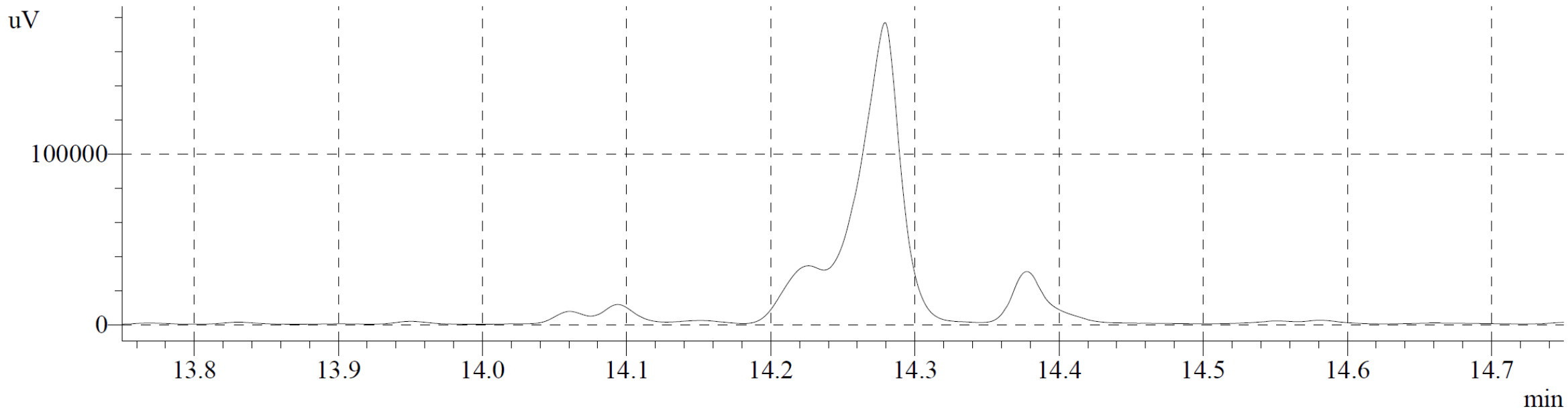

RRF(substrate): 0.917 RRF(ketone): 0.833

i	Retention time, min	Area (A)	Percentage (P), %	Percentage with RRF (P^{RRF}), %	Selectivity, %
-	13.036 (substrate)	101853	7.2878	8.0	
-	sum of peaks 14.006-14.468	508831	36.408	43.7	
4	14.279	355580	25.442	30.5	70.0
	9.017 (biphenyl)	279516	TON = 22; TOF = $2.4 \cdot 10^{-2}$ s^{-1}		
	Conversion: 92.0		Total product yield: 43.7		

The main oxidation product of the **5**-catalyzed oxidation was isolated and characterized following a scaled-up synthesis.

Scaled-up synthesis (×10)
TFE (1.70 mL), substrate **S12** (43.5 mg, 185 µmol), 2,2-dimethylpropanoic acid (18.9 mg, 185 µmol) and 200 µL of TfOH solution in TFE (2.5 mol% of TfOH in relation to the substrate) were added to an oven-dried 12 mL vial charged with catalyst **5** (2.79 mg, 1.85 µmol) and a stirring bar. The mixture was cooled to 0 °C. A solution of an 50% aqueous H_2O_2 diluted in TFE (370 µL; stock solution: 110 µL 50 % aqueous H_2O_2 in 1.90 mL TFE) was slowly added over 30 minutes (12.3 µL/min) under stirring via a syringe pump. After the addition, the mixture was left stirring for another 30 min.

Work-up and IBX Oxidation: after the time indicated, H_2O (6.0 mL) was added and the mixture was extracted with HPLC grade EtOAc (2.0 mL × 3), washed with sat. $NaHCO_3$ (2.5 mL × 2), filtered through a silica plug (glass filter filled with 6.5 g of silica gel and 6.0 g of Na_2SO_4 on top) into a 100 mL one-neck round-bottom flask to remove catalyst **5** and rinsed with EtOAc (30 mL). Organic solvents were removed *in vacuo*, EtOAc (30 mL) was added and the flask was equipped with a stirring bar. IBX (124 mg, 444 µmol) was added and the mixture was heated to 66 °C (oil bath temperature) overnight. The mixture was allowed to cool down to room temperature, filtered through a silica plug (glass filter filled with 7.0 g of silica gel) into a 100 mL one-neck round-bottom flask to remove excess of IBX and byproducts and rinsed with EtOAc (20 mL). Organic solvents were evaporated to give crude material.

Isolation of the product: the product was isolated by preparative HPLC using the Reprospher 100 Si (particle size: 5 µm; dimensions: 250 × 25 mm) Dr. Maisch column. The crude was dissolved in 1.0 mL cyclohexane and 0.10 mL DCM, filtered and eluted by cyclohexane/EtOH using isocratic program with 5% EtOH (flow rate: 15 mL/min, λ = 270 nm). Obtained fractions were checked by GC, in order to identify pure fractions of C4-oxidized compound **S12**. The mixed fractions after the HPLC first run containing the desired product were combined and subjected to HPLC one more time. That gave the C4-oxidized compound **S12** as white solid.

GC analysis of the pure isolated product:

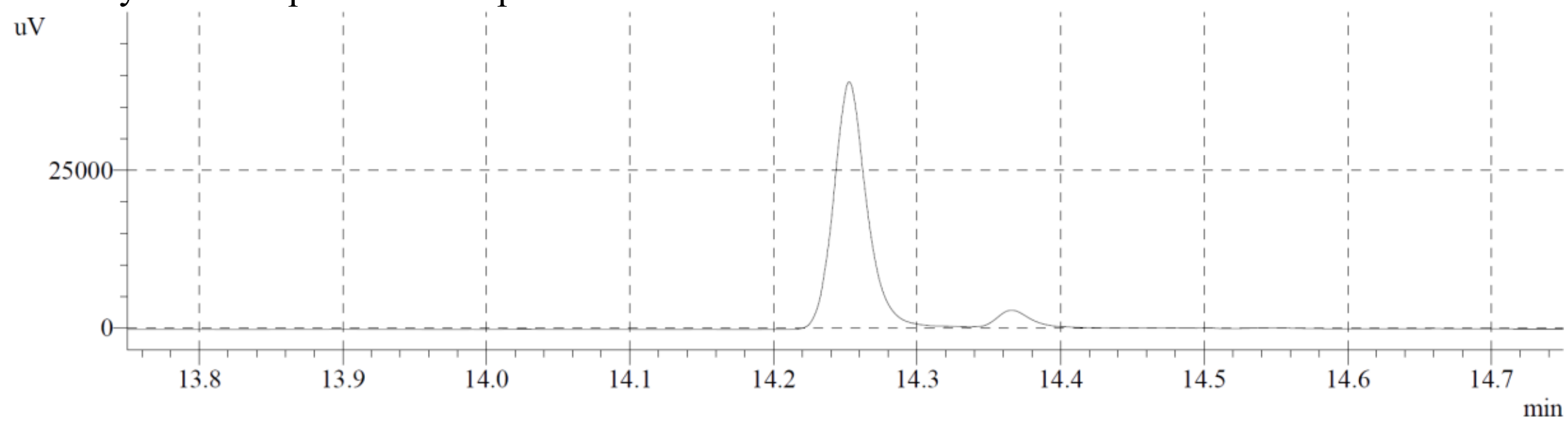

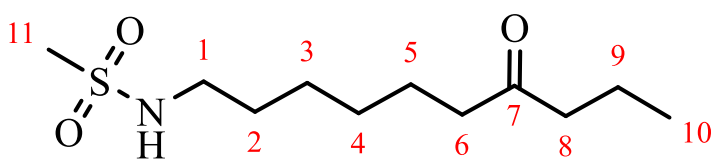

Chemical Formula: $C_{11}H_{23}NO_3S$
Molecular Weight: 249.37

Yield: 14 mg, 30% (94% GC purity).

Mp.: 58 – 59 °C.

IR (ID7, cm^{-1}) 3263 (m), 2926 (m), 2856 (w), 1699 (s), 1467 (w), 1430 (w), 1414 (m), 1374 (w), 1327 (m), 1301 (s), 1216 (w), 1138 (s), 1062 (s), 997 (m), 980 (m), 890 (w), 764 (m), 727 (w), 633 (w), 522 (s), 510 (m), 423 (w).

^{1}H NMR (500 MHz, $CDCl_3$) δ 4.39 (br t, ^3J = 5.9 Hz, 1H, NH), 3.11 (dt, ^3J = 7.1 Hz, ^3J = 5.9 Hz, 2H, C(6)H_2), 2.95 (s, 3H, Me(11)), 2.39 (t, ^3J = 7.3 Hz, 2H, C(6)H_2), 2.36 (t, ^3J = 7.4 Hz, 2H, C(8)H_2), 1.64 – 1.52 (m, 6H, C(2)H_2 + C(5)H_2+ C(9)H_2), 1.39 – 1.32 (m, 2H, C(3)H_2), 1.32 – 1.27 (m, 2H, C(4)H_2), 0.90 (t, ^3J = 7.4 Hz, 3H, Me(10)).

^{13}C NMR (125 MHz, $CDCl_3$) δ 211.3 (C=O), 44.7 (C(8)H_2), 43.2 (C(1)H_2), 42.5 (C(6)H_2), 40.2 (Me(11)), 29.9 (C(2)H_2), 28.6 (C(4)H_2), 26.27 (C(3)H_2), 23.4 (C(5)H_2), 17.3 (C(9)H_2), 13.7 (Me(10)).

HRMS (ESI$^+$) *m/z* calcd for $C_{11}H_{24}NO_3S$ $[M + H]^+$: 250.1471; found 250.1474.

(-)-Menthyl laurate (S13) oxidation

The oxidation was performed using (-)-menthyl laurate (7.23 µL, 6.26 mg).
The following temperature-program was used: Rtx-5; 60 °C for 3 min, 15 °C/min to 250 °C and 250 °C for 5 min.

GC analysis of the **Mn(mcp)** catalyzed oxidation of (-)-menthyl laurate:

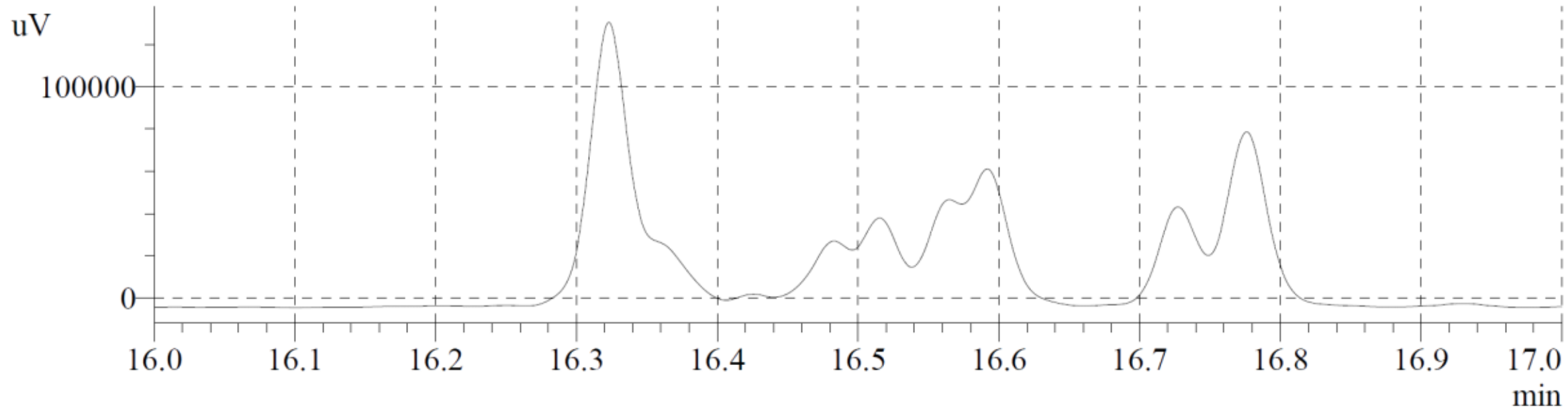

RRF(substrate): 1.75 RRF(ketone): 1.67

i	Retention time, min	Area (A)	Percentage (P), %	Percentage with RRF (P^{RRF}), %	Selectivity, %
-	15.390 (substrate)	913061	61.671	35.2	
-	sum of peaks 16.221-16.876	957677	64.685	38.8	
4	16.592	137068	9.2581	5.6	14.3
	9.027 (biphenyl)	296105	TON = 39; TOF = $4.3\cdot10^{-2}$ s^{-1}		
	Conversion: 64.8		Total product yield: 38.8		

GC analysis of the **5**-catalyzed oxidation of (-)-menthyl laurate:

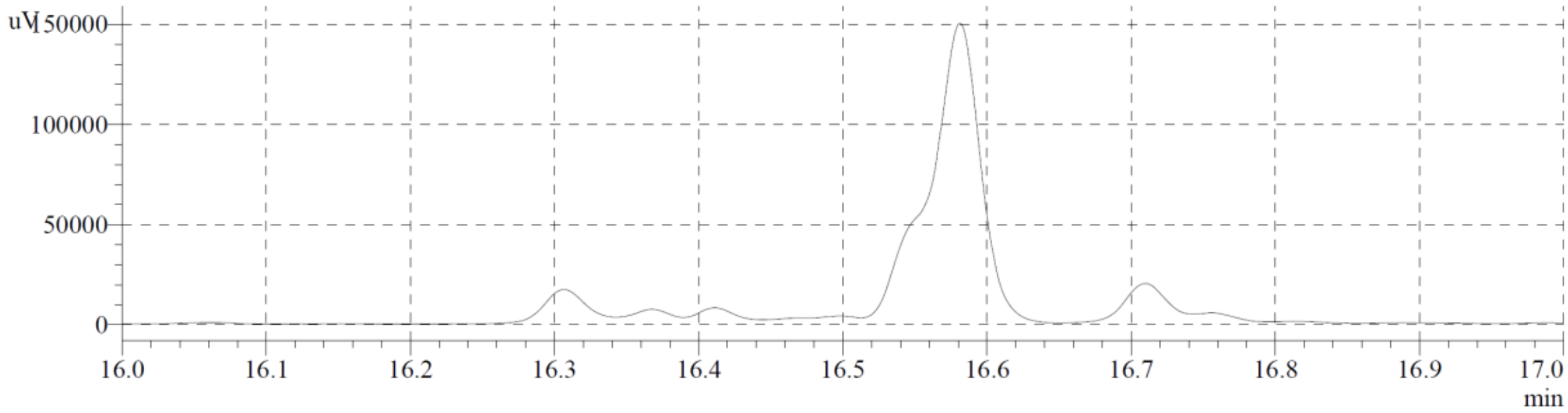

RRF(substrate): 1.75 RRF(ketone): 1.67

i	Retention time, min	Area (A)	Percentage (P), %	Percentage with RRF (P^{RRF}), %	Selectivity, %
-	15.376 (substrate)	393914	28.760	16.4	
-	sum of peaks 16.263-17.852	1065766	77.813	46.7	
4	16.598	638644	46.628	28.0	59.9
	9.018 (biphenyl)	273930	TON = 23; TOF = $2.6\cdot10^{-2}$ s^{-1}		
	Conversion: 83.6		Total product yield: 46.7		

The main oxidation product of the **5**-catalyzed oxidation was isolated and characterized following a scaled-up synthesis.

Scaled-up synthesis (×10)

TFE (1.70 mL), substrate **S13** (72.3 µL, 62.6 mg, 185 µmol), 2,2-dimethylpropanoic acid (18.9 mg, 185 µmol) and 200 µL of TfOH solution in TFE (2.5 mol% of TfOH in relation to the substrate) were added to an oven-dried 12 mL vial charged with catalyst **5** (2.79 mg, 1.85 µmol) and a stirring bar. The mixture was cooled to 0 °C. A solution of an 50% aqueous H_2O_2 diluted in TFE (370 µL; stock solution: 110 µL 50 % aqueous H_2O_2 in 1.90 mL TFE) was slowly added over 30 minutes (12.3 µL/min) under stirring via a syringe pump. After the addition, the mixture was left stirring for another 30 min.

Work-up and IBX Oxidation: after the time indicated, H_2O (6.0 mL) was added and the mixture was extracted with HPLC grade EtOAc (2.0 mL × 3), washed with sat. $NaHCO_3$ (2.5 mL × 2), filtered through a silica plug (glass filter filled with 6.5 g of silica gel and 6.0 g of Na_2SO_4 on top) into a 100 mL one-neck round-bottom flask to remove catalyst **5** and rinsed with EtOAc (30 mL). Organic solvents were removed *in vacuo*, EtOAc (30 mL) was added and the flask was equipped with a stirring bar. IBX (124 mg, 444 µmol) was added and the mixture was heated to 66 °C (oil bath temperature) overnight. The mixture was allowed to cool down to room temperature, filtered through a silica plug (glass filter filled with 7.0 g of silica gel) into a 100 mL one-neck round-bottom flask to remove excess of IBX and byproducts and rinsed with EtOAc (20 mL). Organic solvents were evaporated to give crude material.

Isolation of the product: the product was isolated by preparative HPLC using the Reprospher 100 Si (particle size: 5 µm; dimensions: 250 × 25 mm) Dr. Maisch column. The crude was dissolved in 1.0 mL cyclohexane, filtered and eluted by cyclohexane/EtOAc using isocratic program with 1.5% EtOAc (flow rate: 15 mL/min). Obtained fractions were checked by GC, in order to identify pure fractions of C4-oxidized compound **S13**. Pure fractions were combined, organic solvents were evaporated to give the product as colourless oil.

GC analysis of the pure isolated product:

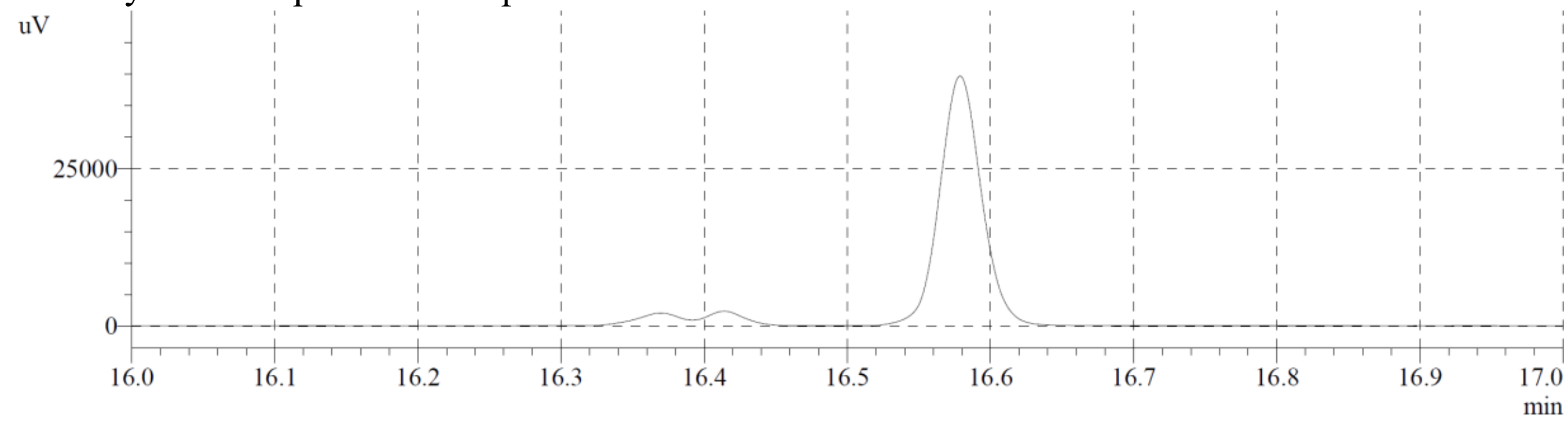

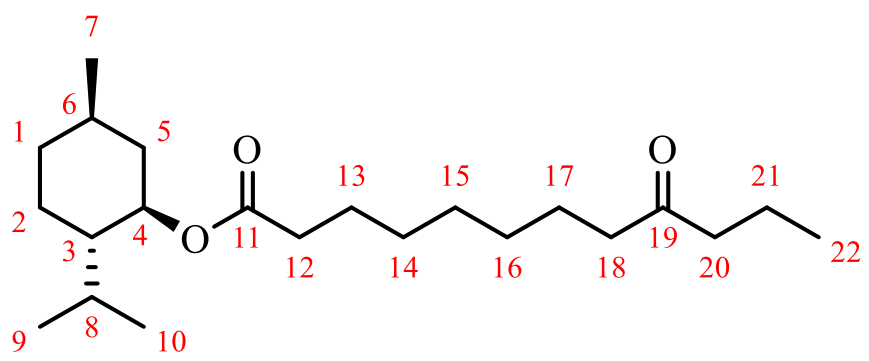

Chemical Formula: $C_{22}H_{40}O_3$
Molecular Weight: 352.56

Yield: 15 mg, 23% (90% GC purity).

IR (ID7, cm^{-1}) 2949 (w), 2924 (m), 2862 (m), 1724 (s), 1714 (s), 1455 (m), 1412 (w), 1366 (m), 1243 (m), 1173 (m), 1095 (w), 1038 (w), 1009 (w), 982 (w), 910 (w), 842 (w), 725 (w), 506 (w), 466 (w).

^{1}H NMR (500 MHz, $CDCl_3$) δ 4.66 (dt, ^3J = 10.9 Hz, ^3J = 4.4 Hz, 1H, C(4)H), 2.37 (t, ^3J = 7.4 Hz, 2H, C(18)H_2), 2.36 (t, ^3J = 7.3 Hz, 2H, C(20)H_2), 2.20 – 2.30 (m, 2H, C(12)H_2), 1.92 – 2.02 (m, 1H, C(5)H_2), 1.79 – 1.91 (m, 1H, C(8)H), 1.63 – 1.70 (m, 2H, C(1)H_2+C(2)H_2), 1.51 – 1.63 (m, 6H, C(13)H_2+C(17)H_2+C(21)H_2), 1.43 – 1.51 (m, 1H, C(6)H), 1.32 – 1.39 (m, 1H, C(3)H), 1.21 – 1.32 (m, 6H, C(14)H_2+C(15)H_2+C(16)H_2), 1.00 – 1.10 (m, 1H, C(2)H_2), 0.80 – 0.99 (m, 11H, C(1)H_2+C(5)H_2+Me(7)+Me(9 or 10)+Me(22)), 0.75 (d, ^3J = 7.0 Hz, 3H, Me(9 or 10)).

^{13}C NMR (125 MHz, $CDCl_3$) δ 211.4 (C(19)=O), 173.4 (C(11)=O), 73.9 (C(4)H), 47.0 (C(3)H), 44.7 (C(20)H_2), 42.7 (C(18)H_2), 40.9 (C(5)H_2), 34.7 (C(12)H_2), 34.3 (C(1)H_2), 31.4 (C(6)H), 29.1 (C(14 or 15 or 16)H_2), 29.0 (C(14 or 15 or 16)H_2), 28.9 (C(14 or 15 or 16)H_2), 26.2 (C(8)H), 25.0 (C(13)H_2), 23.7 (C(17)H_2), 23.4 (C(2)H_2), 22.0 (Me(7)), 20.7 (Me(9 or 10)), 17.3 (C(21)H_2), 16.3 (Me(9 or 10)), 13.8 (Me(22)).

HRMS (ESI$^+$) *m/z* calcd for $C_{22}H_{40}O_3Na$ $[M + Na]^+$: 375.2870; found 375.2873.

2-Methylnonane (S14) oxidation

The oxidation was performed using 2-methylnonane (3.63 µL, 2.63 mg).
The following temperature-program was used: Rtx-5; 60 °C for 3 min, 25 °C/min to 130 °C, 0.5 °C/min to 140 °C, 25 °C/min to 250 °C and 250 °C for 5 min.

GC analysis of the **Mn(mcp)** catalyzed oxidation of 2-methylnonane:

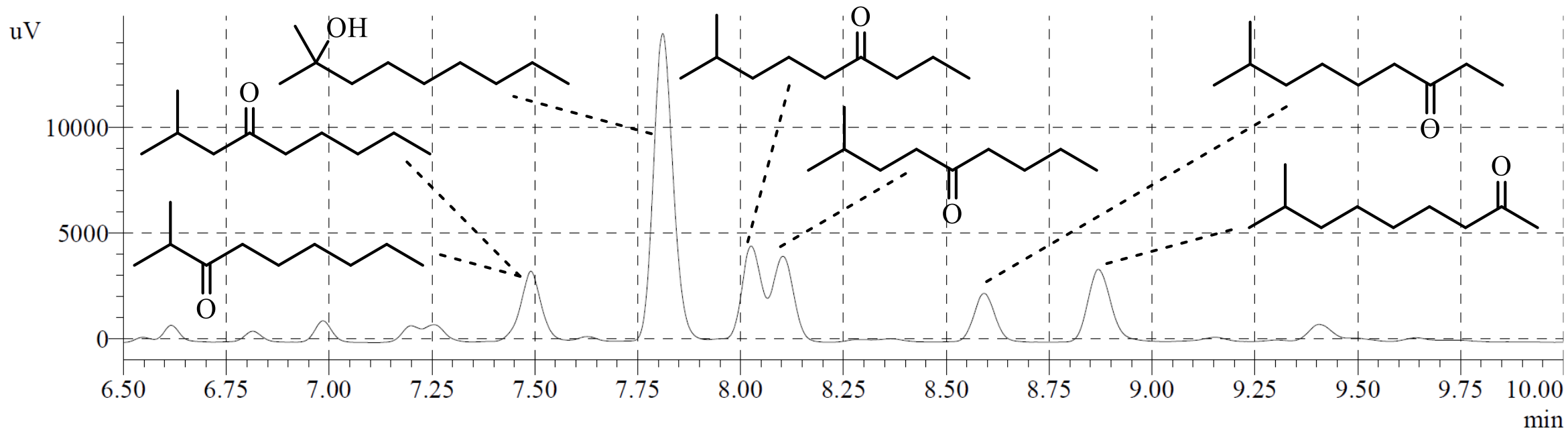

RRF(substrate): 0.833 RRF(ketone): 0.750; RRF(tertiary alcohol): 0.813

i	Retention time, min	Area (A)	Percentage (P), %	Percentage with RRF (P^{RRF}), %	Selectivity, %
-	3.987 (substrate)	541916	33.152	39.8	
6+7	7.490	12458	0.76212	1.0	11.3
8	7.811	49447	3.0249	3.7	41.4
4	8.026	16134	0.98700	1.3	14.6
5	8.103	13997	0.85627	1.1	12.7
3	8.592	8417	0.51491	0.7	7.6
2	8.87	13568	0.83002	1.1	12.3
	24.030 (biphenyl)	326931	TON = 9; TOF = $9.9\cdot10^{-3}$ s^{-1}		
	Conversion: 60.2		Total product yield: 8.9		

GC analysis of the **5**-catalyzed oxidation of 2-methylnonane:

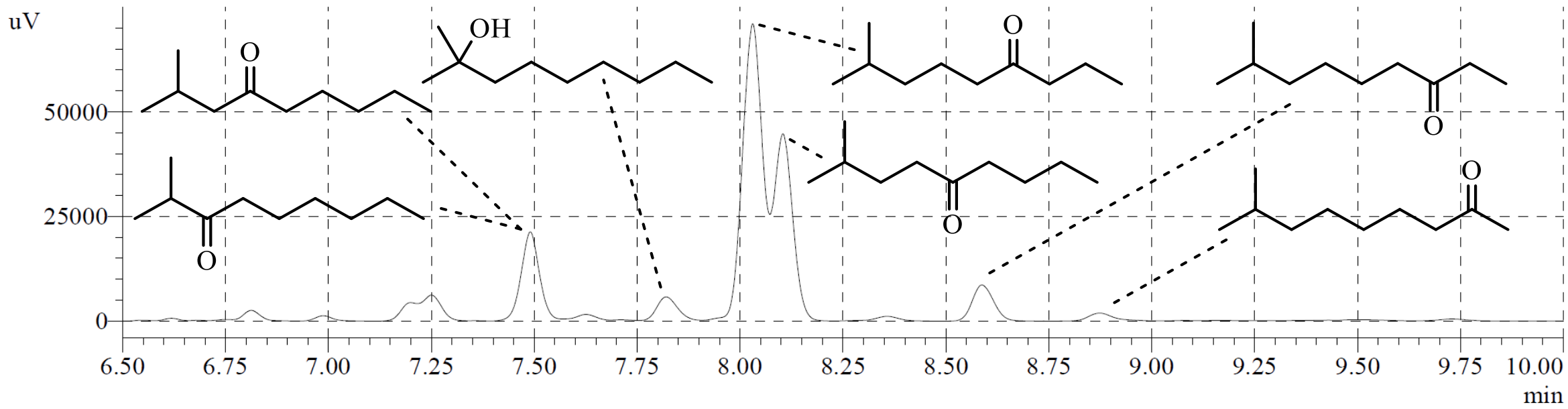

RRF(substrate): 0.833 RRF(ketone): 0.750; RRF(tertiary alcohol): 0.813

i	Retention time, min	Area (A)	Percentage (P), %	Percentage with RRF (P^{RRF}), %	Selectivity, %
-	3.986 (substrate)	160435	9.6555	11.6	
6+7	7.490	63963	3.8495	5.1	12.6
8	7.820	19913	1.1984	1.5	3.6
4	8.031	242853	14.6156	19.5	48.0
5	8.104	140533	8.4577	11.3	27.8
3	8.587	31510	1.8964	2.5	6.2
2	8.873	8688	0.52287	0.7	1.7
	24.026 (biphenyl)	332319	TON = 20; TOF = $2.3\cdot10^{-2}$ s^{-1}		
	Conversion: 88.4		Total product yield: 40.6		

The different oxidation products were assigned according to literature data.[3]

2,7-Dimethyloctane (S15) oxidation

The oxidation was performed using 2,7-dimethyloctane (3.66 µL, 2.63 mg).
The following temperature-program was used: Rtx-5; 60 °C for 3 min, 15 °C/min to 250 °C and 250 °C for 5 min.

GC analysis of the **Mn(mcp)** catalyzed oxidation of 2,7-dimethyloctane:

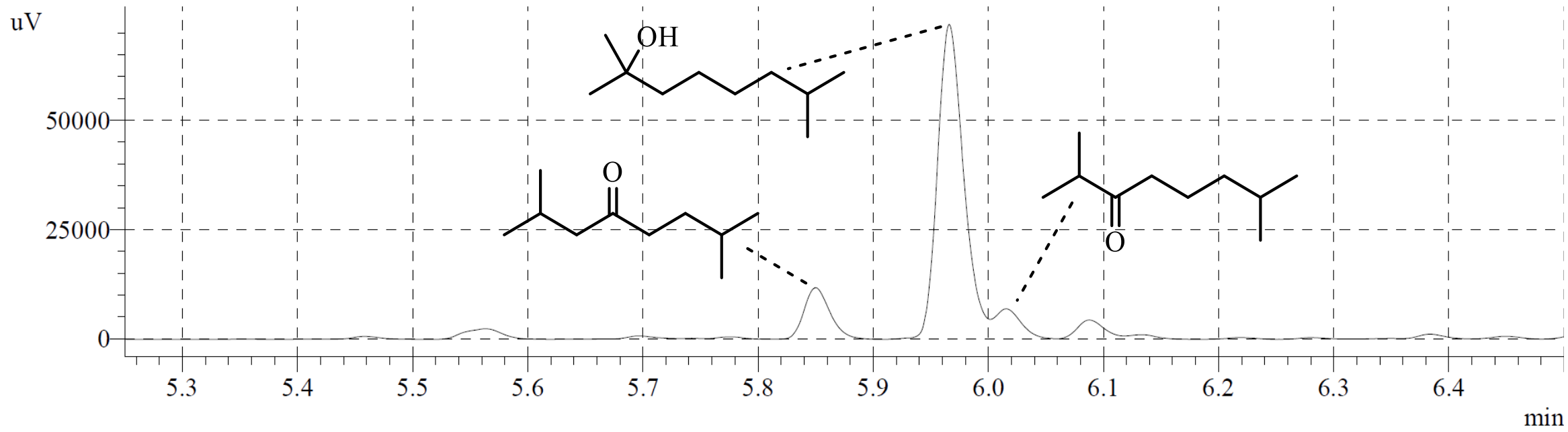

RRF(substrate): 0.833 RRF(ketone): 0.750; RRF(tertiary alcohol): 0.813

i	Retention time, min	Area (A)	Percentage (P), %	Percentage with RRF (P^{RRF}), %	Selectivity, %
-	3.543 (substrate)	506238	32.559	39.1	
4	5.85	18483	1.1887	1.6	13.8
2	5.966	113929	7.3274	9.0	78.4
3	6.016	10539	0.67782	0.9	7.8
	9.031 (biphenyl)	310968	TON = 12; TOF = $1.3\cdot10^{-2}$ s^{-1}		
	Conversion: 60.9		Total product yield: 11.5		

GC analysis of the **5**-catalyzed oxidation of 2,7-dimethyloctane:

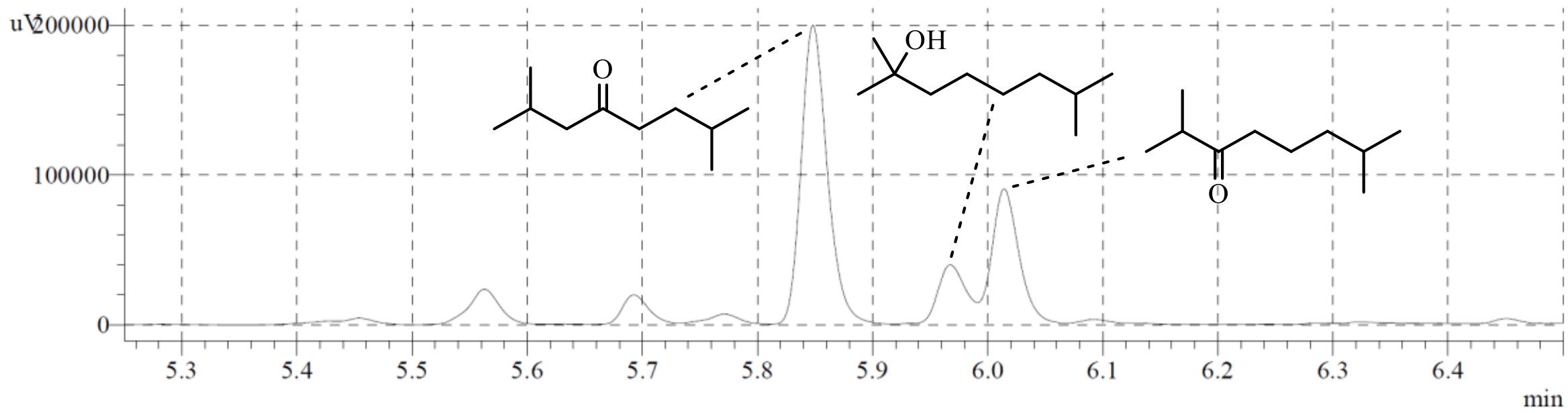

RRF(substrate): 0.833 RRF(ketone): 0.750; RRF(tertiary alcohol): 0.813

i	Retention time, min	Area (A)	Percentage (P), %	Percentage with RRF (P^{RRF}), %	Selectivity, %
-	3.538 (substrate)	58878	3.0804	3.7	
4	5.848	314796	16.469	22.0	60.2
2	5.968	67624	3.5380	4.4	11.9
3	6.014	145774	7.6266	10.2	27.9
	9.029 (biphenyl)	382278	TON = 18; TOF = $2.0\cdot10^{-2}$ s^{-1}		
	Conversion: 96.3		Total product yield: 36.6		

The different oxidation products were assigned according to literature data.[3]

cis-1-Isopropyl-4-methylcyclohexane (S16) oxidation

The oxidation was performed using *cis*-1-isopropyl-4-methylcyclohexane (3.23 µL, 2.59 mg).
The following temperature-program was used: MEGA-DEX DAC β; start at 50 °C, 3 °C/min to 100 °C, 100 °C for 15 min, 25 °C/min to 220 °C and 220 °C for 3 min.

GC analysis of the **Mn(mcp)** catalyzed oxidation of *cis*-1-isopropyl-4-methylcyclohexane:

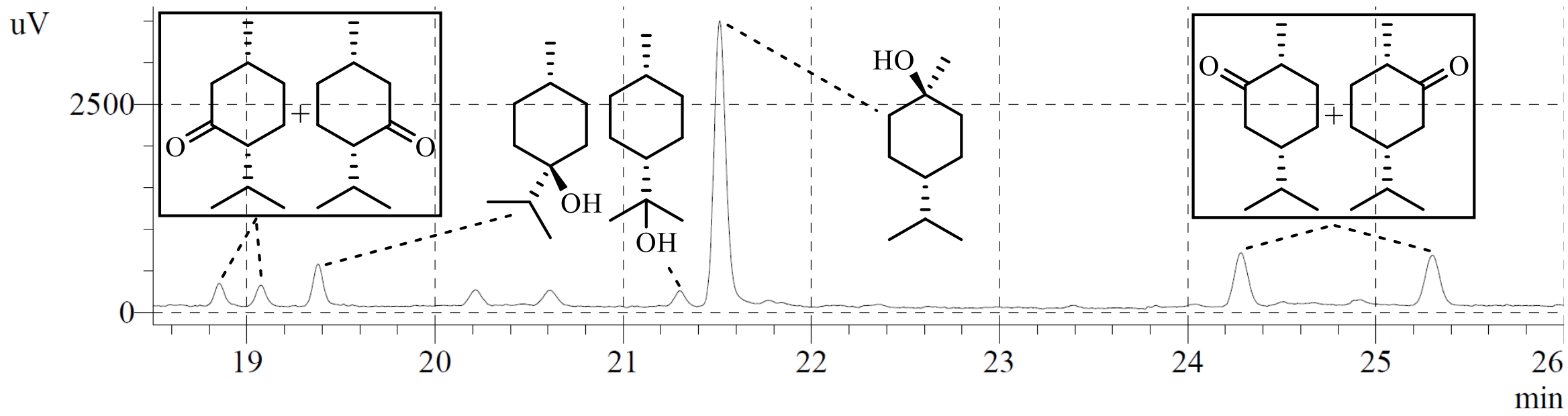

RRF(substrate): 0.833 RRF(ketone): 0.750; RRF(tertiary alcohol): 0.813

i	Retention time, min	Area (A)	Percentage (P), %	Percentage with RRF (P^{RRF}), %	Selectivity, %
-	8.309 (substrate)	76367	24.725	29.7	
3	18.853 + 19.075	2074	0.67149	0.9	8.2
4	19.377	1942	0.62875	0.8	7.1
5	21.301	809	0.26193	0.3	3.0
1	21.512	15262	4.9413	6.1	55.7
2	24.283 + 25.301	6592	2.1343	2.8	26.1
	35.129 (biphenyl)	61773	TON = 11; TOF = $1.2 \cdot 10^{-2}$ s^{-1}		
	Conversion: 70.3		Total product yield: 10.9		

GC analysis of the **5**-catalyzed oxidation of *cis*-1-isopropyl-4-methylcyclohexane:

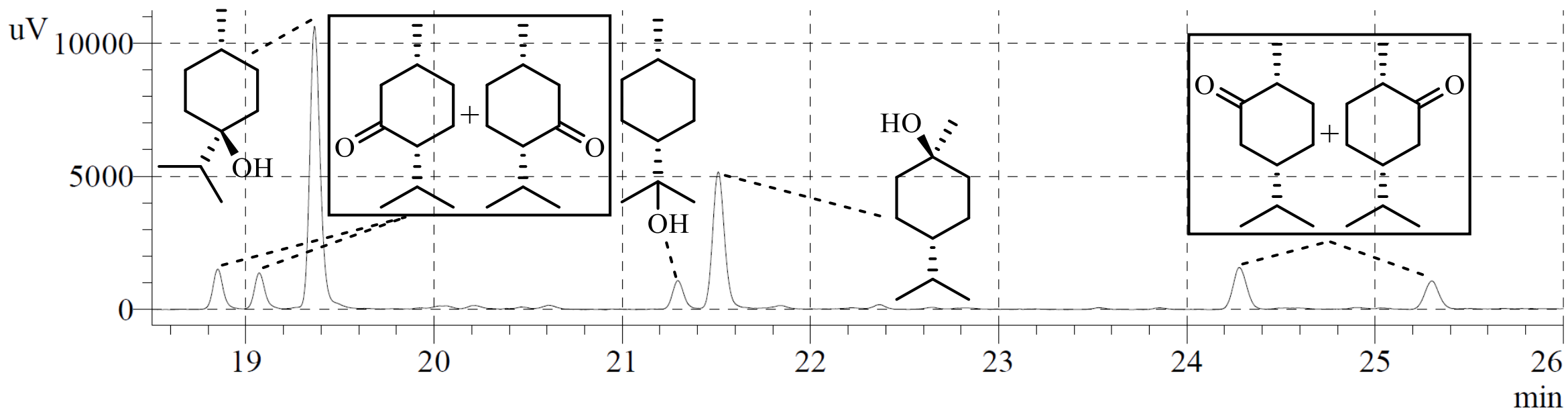

RRF(substrate): 0.833 RRF(ketone): 0.750; RRF(tertiary alcohol): 0.813

i	Retention time, min	Area (A)	Percentage (P), %	Percentage with RRF (P^{RRF}), %	Selectivity, %
-	8.302 (substrate)	24909	7.4568	8.9	
3	18.851 + 19.071	11090	3.3199	4.4	12.5
4	19.365	41919	12.549	15.4	43.6
5	21.295	4483	1.3420	1.6	4.7
1	21.51	22986	6.8811	8.5	23.9
2	24.278 + 25.301	13606	4.0731	5.4	15.3
	35.129 (biphenyl)	66809	TON = 18; TOF = $2.0 \cdot 10^{-2}$ s^{-1}		
	Conversion: 91.1		Total product yield: 35.3		

The different oxidation products were assigned according to standard samples.[3]

Propylcyclohexane (S17) oxidation

The oxidation was performed using propylcyclohexane (2.95 µL, 2.34 mg).
The following temperature-program was used: Rtx-5; 60 °C for 3 min, 15 °C/min to 250 °C and 250 °C for 5 min.

GC analysis of the **Mn(mcp)** catalyzed oxidation of propylcyclohexane:

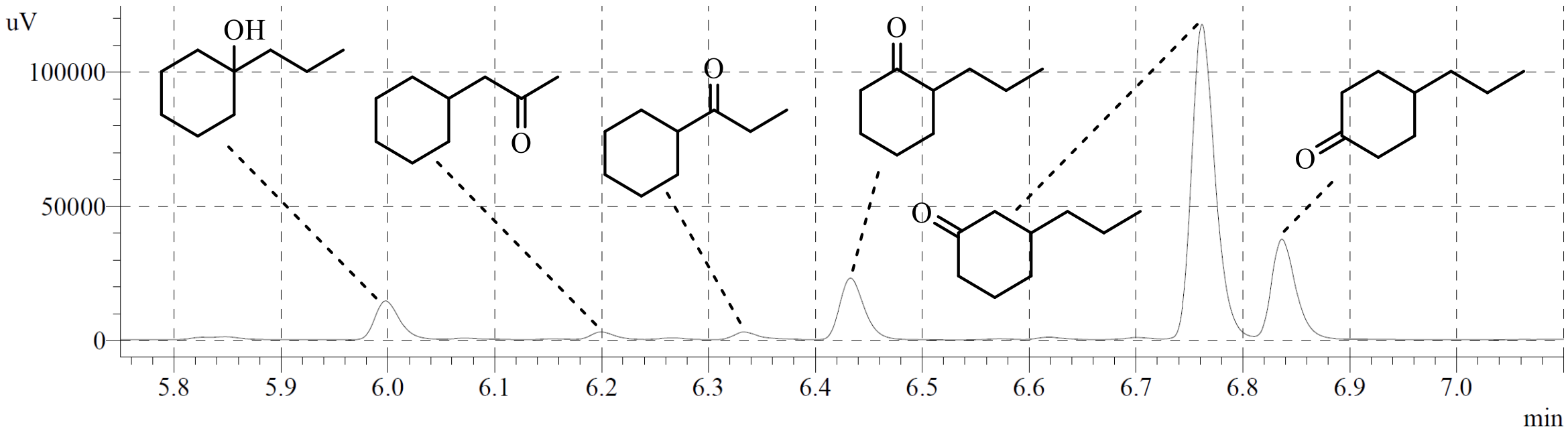

RRF(substrate): 0.750 RRF(ketone): 0.667; RRF(tertiary alcohol): 0.729

i	Retention time, min	Area (A)	Percentage (P), %	Percentage with RRF (P^{RRF}), %	Selectivity, %
-	3.543 (substrate)	391260	27.404	36.5	
4	5.998	23316	1.6331	2.4	7.6
2	6.200	3973	0.27827	0.4	1.3
3	6.333	4816	0.33732	0.5	1.4
5	6.433	36552	2.5601	3.8	11.9
6	6.762	180111	12.615	18.9	58.6
7	6.837	58994	4.1320	6.2	19.2
	9.004 (biphenyl)	285549	TON = 32; TOF = $3.6\cdot10^{-2}$ s^{-1}		
	Conversion: 63.5		Total product yield: 32.2		

GC analysis of the **5**-catalyzed oxidation of propylcyclohexane:

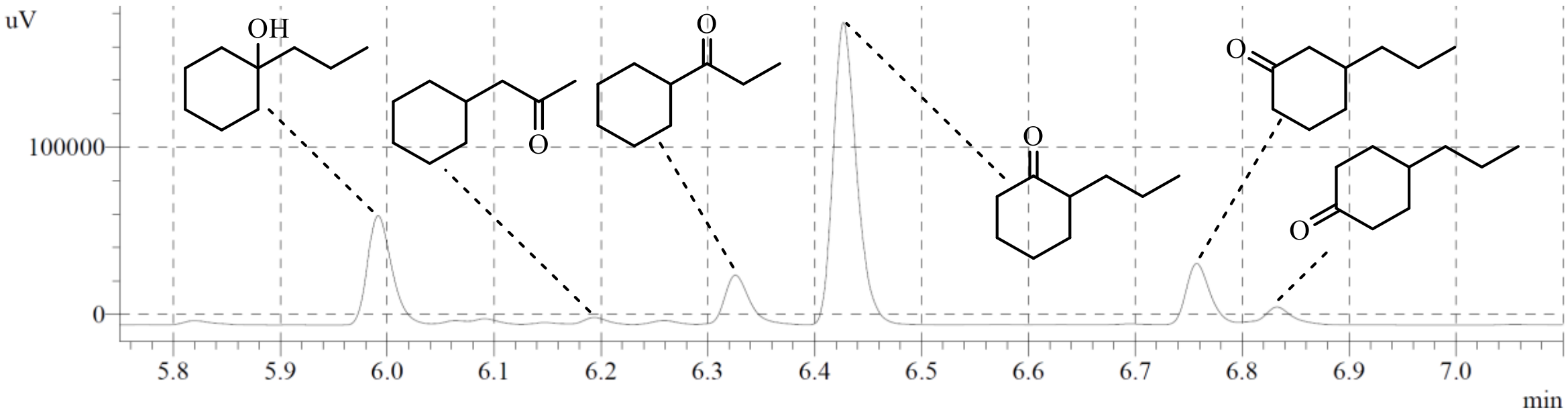

RRF(substrate): 0.750 RRF(ketone): 0.667; RRF(tertiary alcohol): 0.729

i	Retention time, min	Area (A)	Percentage (P), %	Percentage with RRF (P^{RRF}), %	Selectivity, %
-	3.531 (substrate)	141616	10.374	13.8	
4	5.992	105444	7.7245	10.6	19.2
2	6.194	5422	0.39720	0.6	1.1
3	6.326	47238	3.4605	4.7	8.6
5	6.427	276710	20.271	30.4	55.2
6	6.757	58229	4.2656	6.4	11.6
7	6.833	21398	1.5675	2.4	4.3
	9.001 (biphenyl)	273013	TON = 28; TOF = $3.1\cdot10^{-2}$ s^{-1}		
	Conversion: 86.2		Total product yield: 55.1		

GC-MS analyses of the **Mn(mcp)** (top) and **5**-catalyzed (bottom) oxidation of propylcyclohexane. The different oxidation products were assigned according to their fragmentation pattern according to literature data.[18–22]

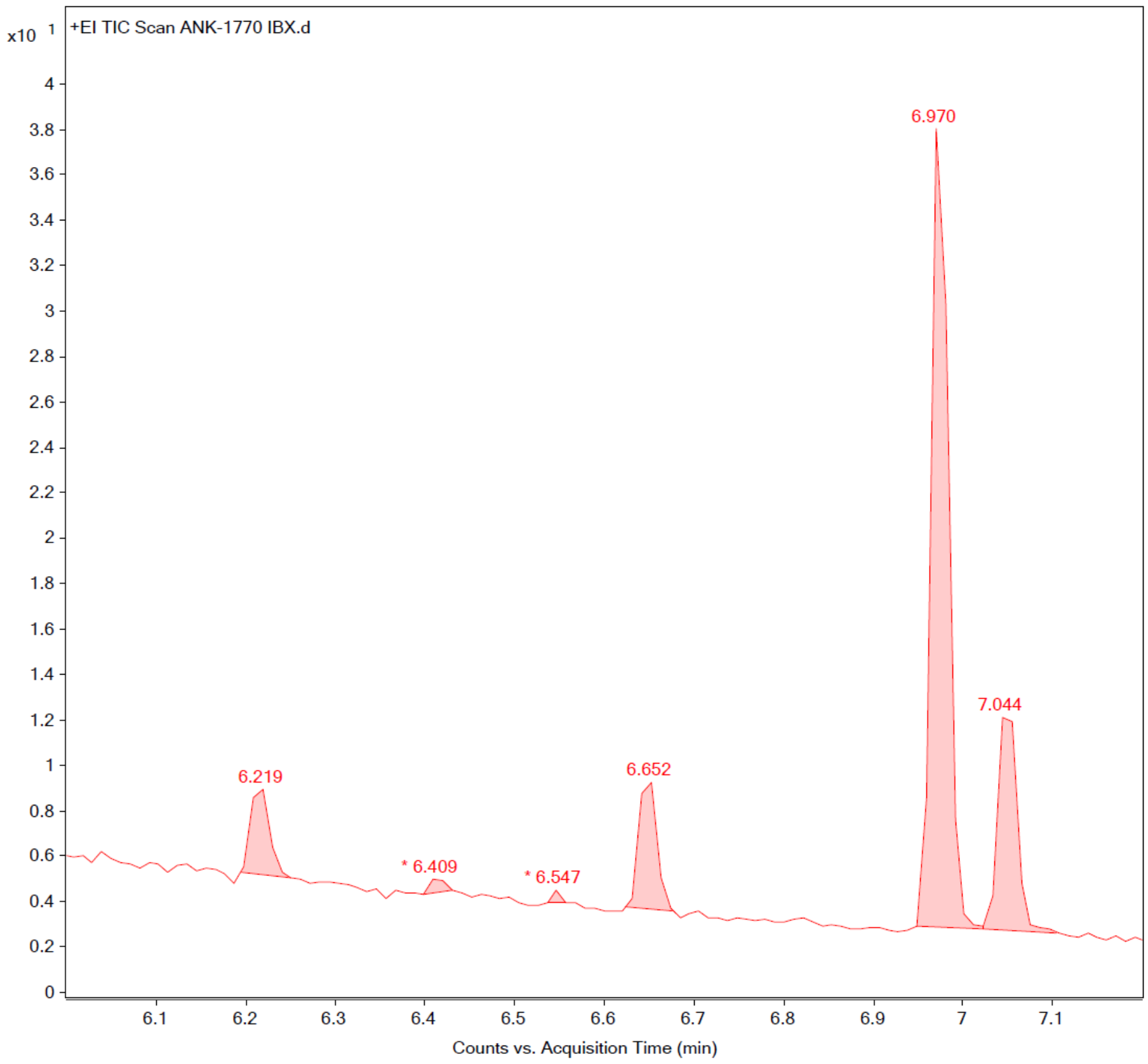

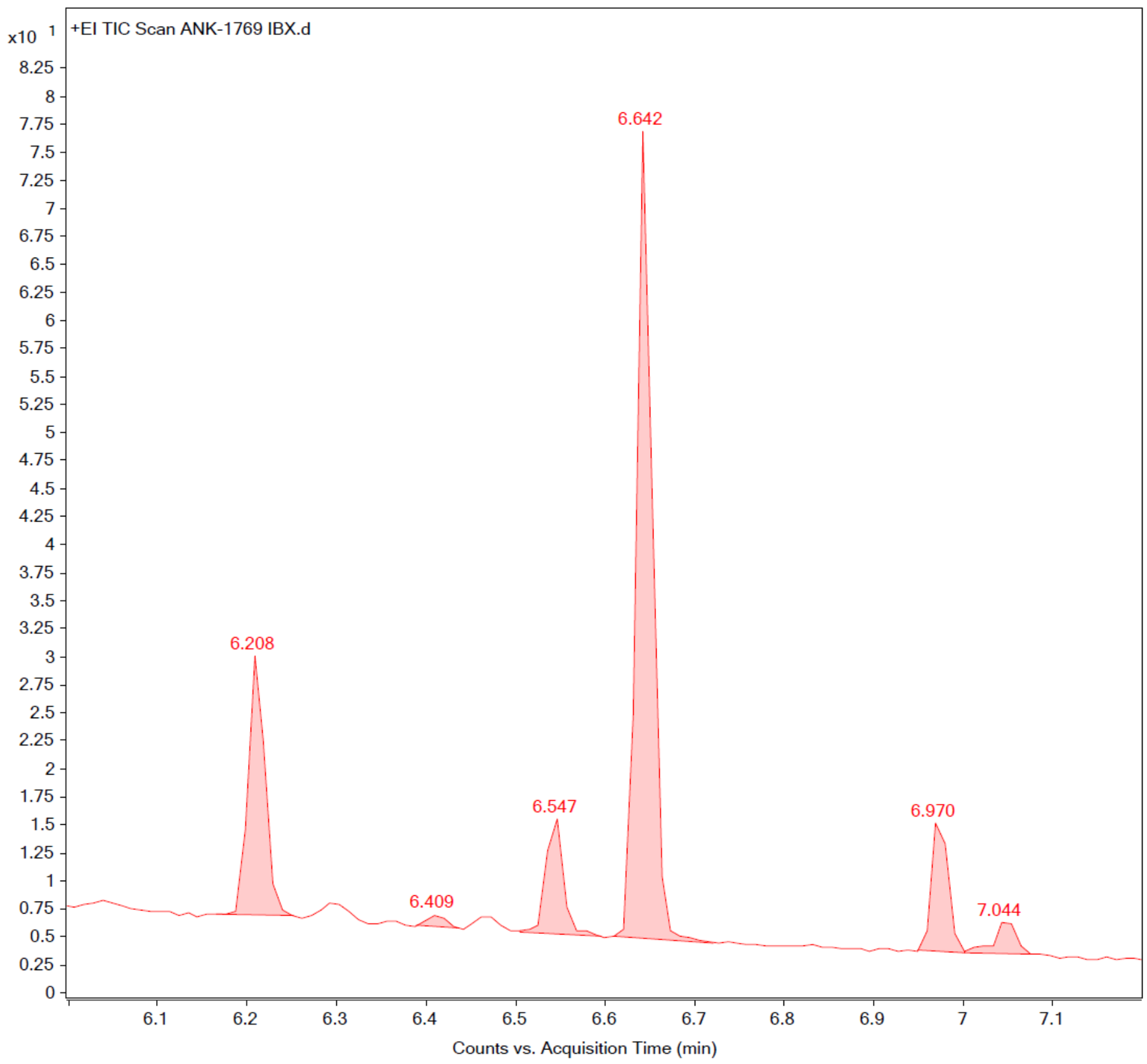

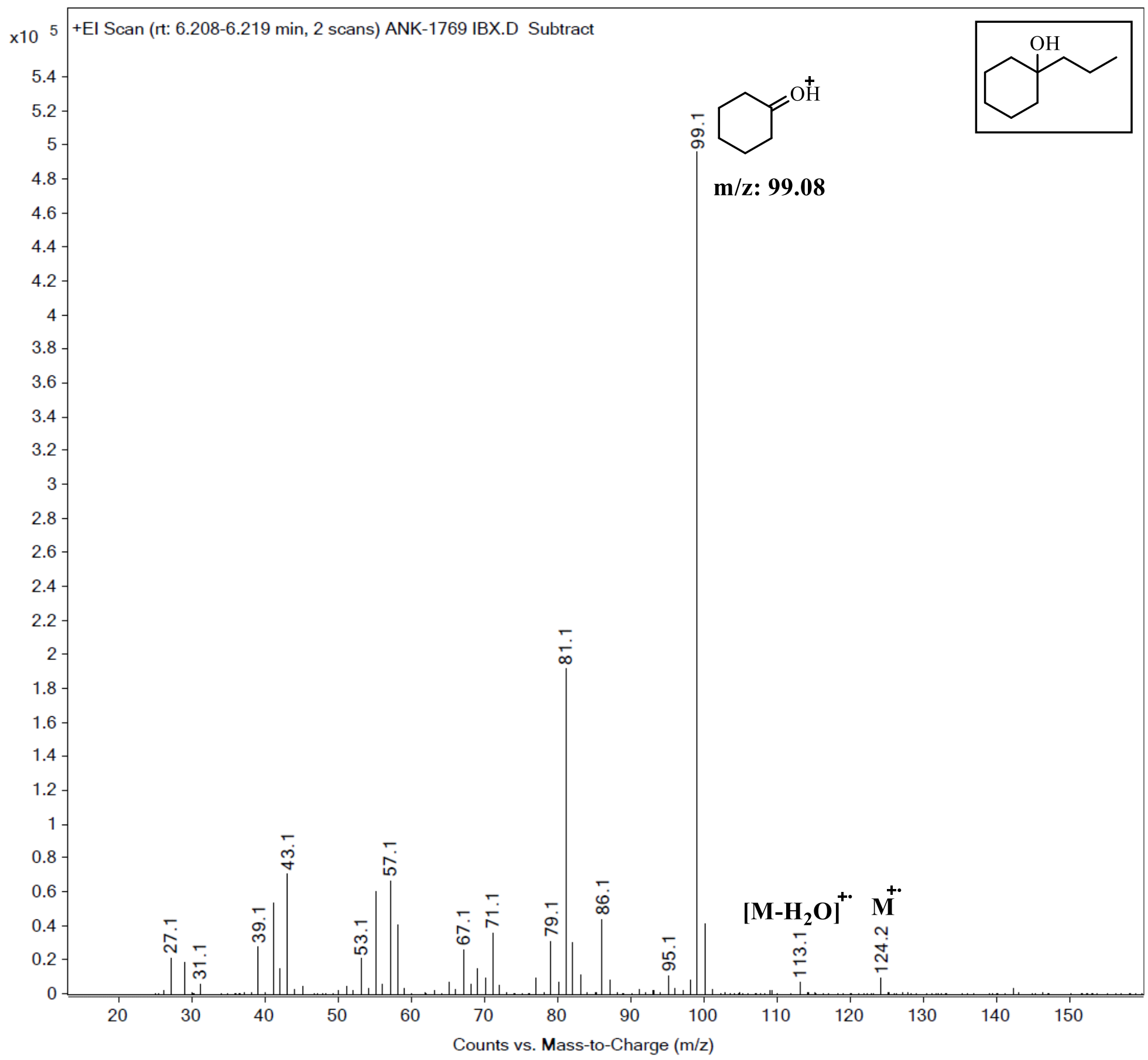

x10 5
+EI Scan (rt: 6.208-6.219 min, 2 scans) ANK-1769 IBX.D Subtract
OH
m/z: 99.08
[M-H2O]+·
M+·
99.1
81.1
43.1
57.1
86.1
71.1
67.1
79.1
39.1
53.1
27.1
31.1
95.1
113.1
124.2
Counts vs. Mass-to-Charge (m/z)

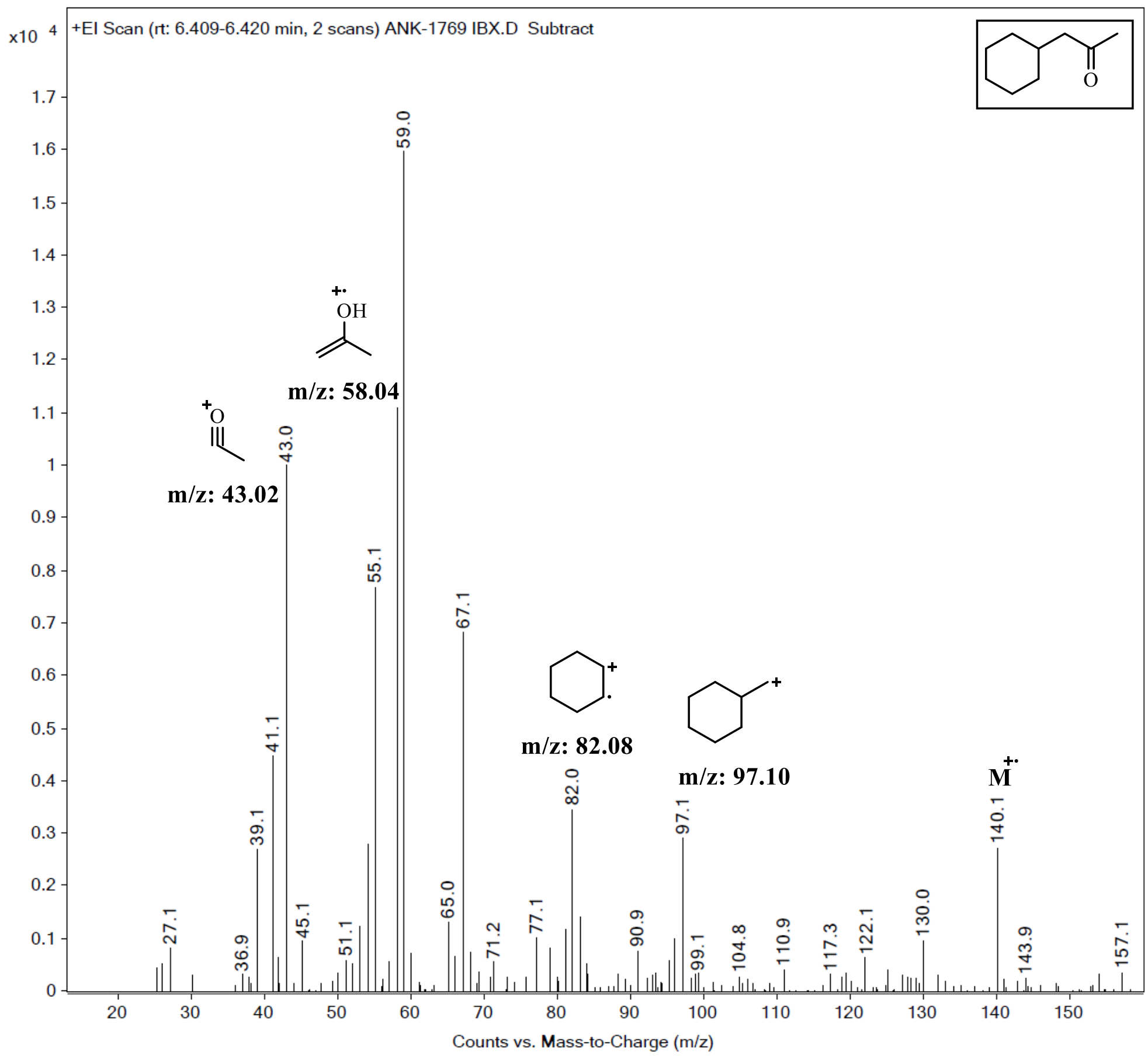

x10 4
+EI Scan (rt: 6.409-6.420 min, 2 scans) ANK-1769 IBX.D Subtract
O
OH
m/z: 58.04
m/z: 43.02
m/z: 82.08
m/z: 97.10
M+·
59.0
43.0
55.1
67.1
41.1
39.1
82.0
97.1
140.1
27.1
36.9
45.1
51.1
65.0
71.2
77.1
90.9
99.1
104.8
110.9
117.3
122.1
130.0
143.9
157.1
Counts vs. Mass-to-Charge (m/z)

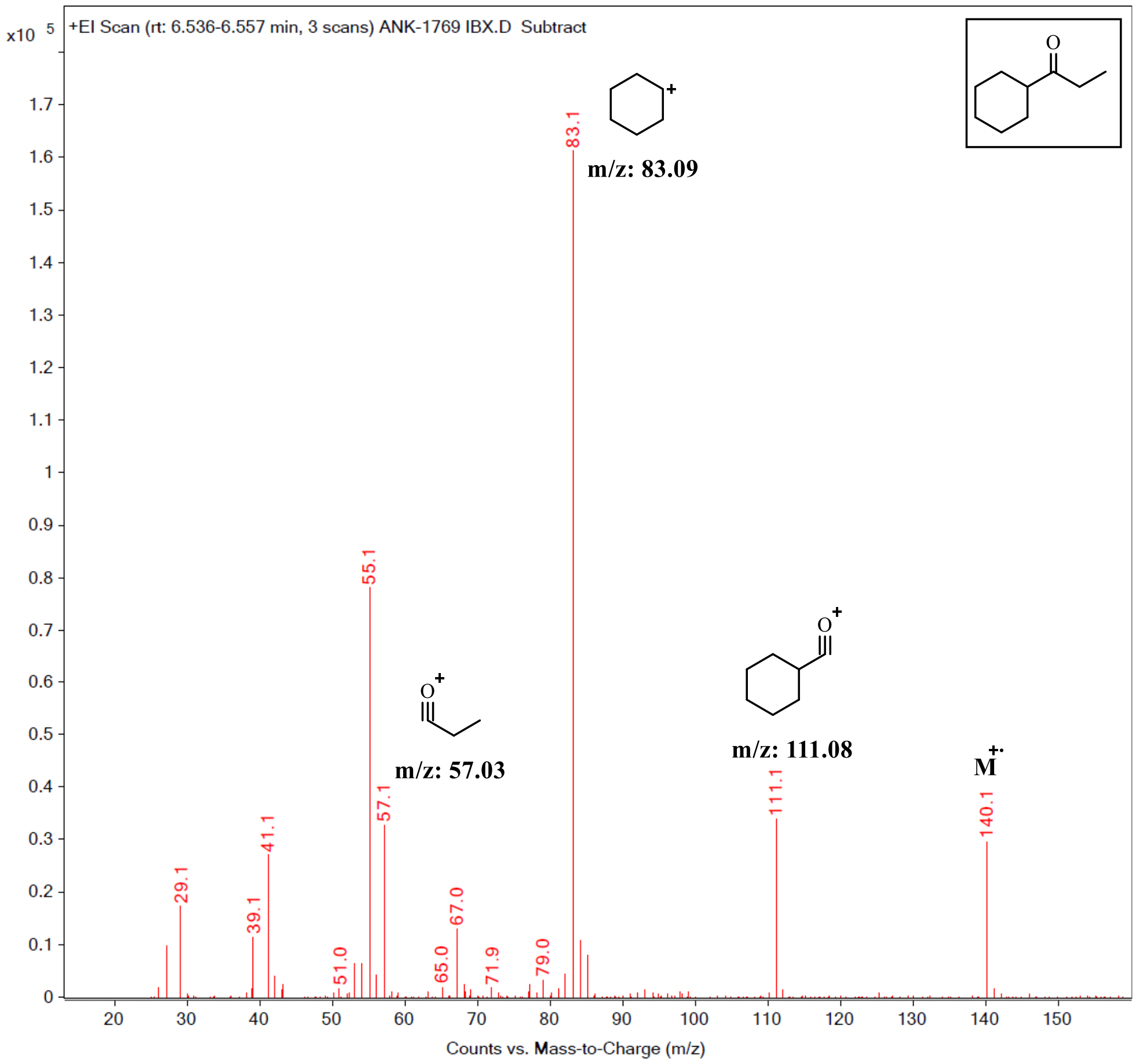

+EI Scan (rt: 6.536-6.557 min, 3 scans) ANK-1769 IBX.D Subtract
x10 5
m/z: 83.09
m/z: 57.03
m/z: 111.08
M
Counts vs. Mass-to-Charge (m/z)

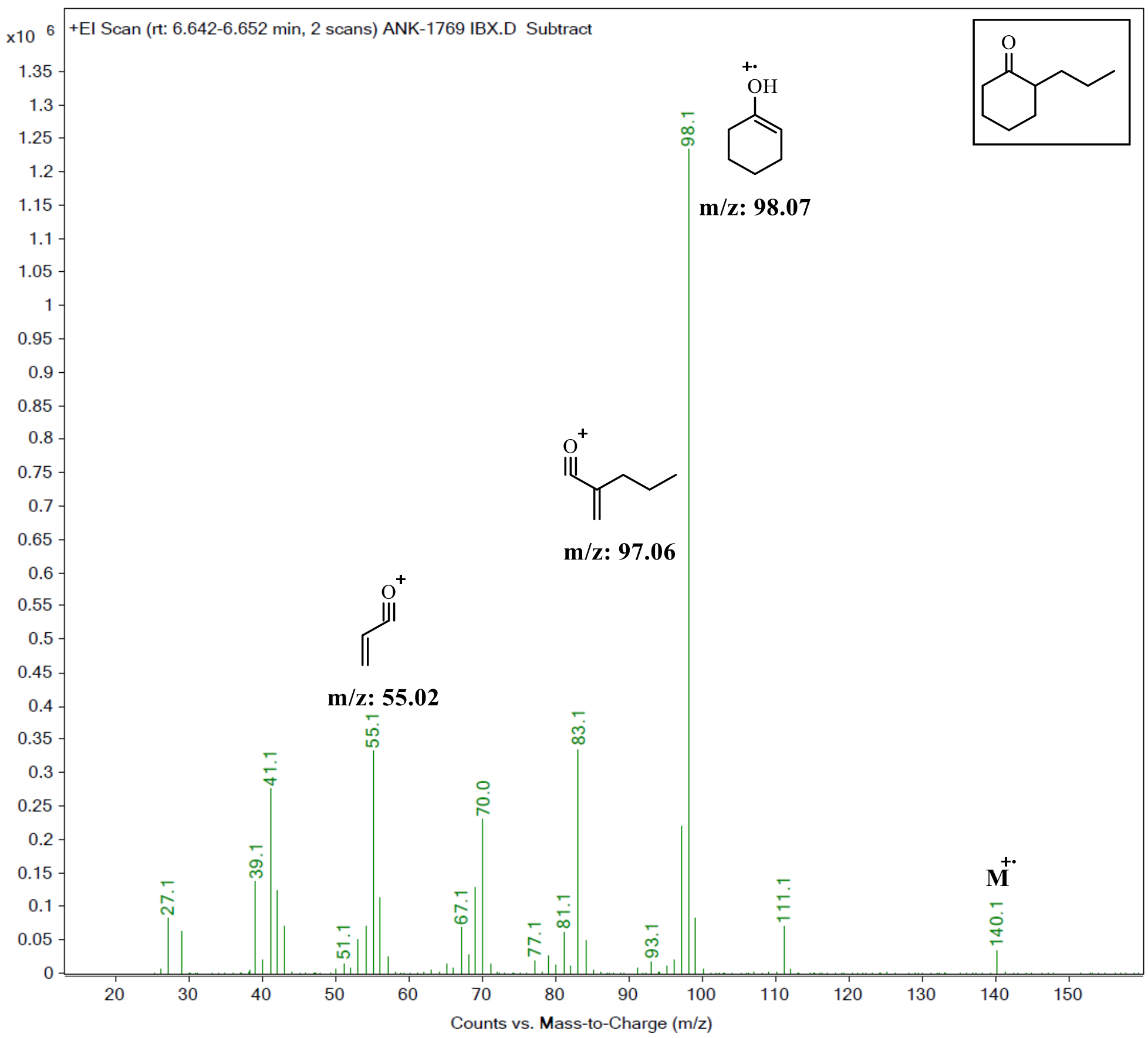

+EI Scan (rt: 6.642-6.652 min, 2 scans) ANK-1769 IBX.D Subtract
x10 6
OH
m/z: 98.07
m/z: 97.06
m/z: 55.02
M
Counts vs. Mass-to-Charge (m/z)

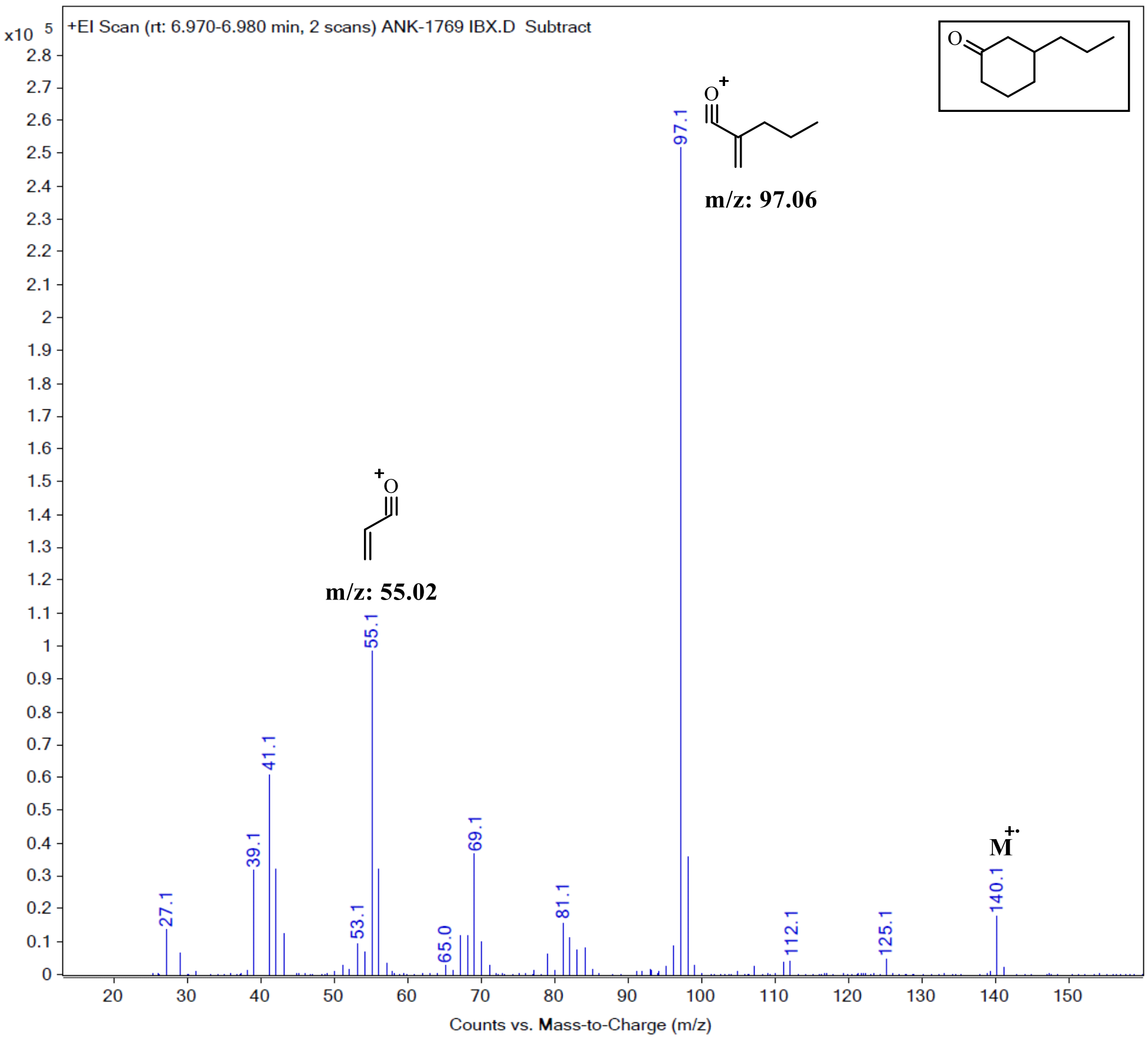
x10 5
+EI Scan (rt: 6.970-6.980 min, 2 scans) ANK-1769 IBX.D Subtract
m/z: 97.06
m/z: 55.02
M+·
27.1
39.1
41.1
53.1
55.1
65.0
69.1
81.1
97.1
112.1
125.1
140.1
Counts vs. Mass-to-Charge (m/z)

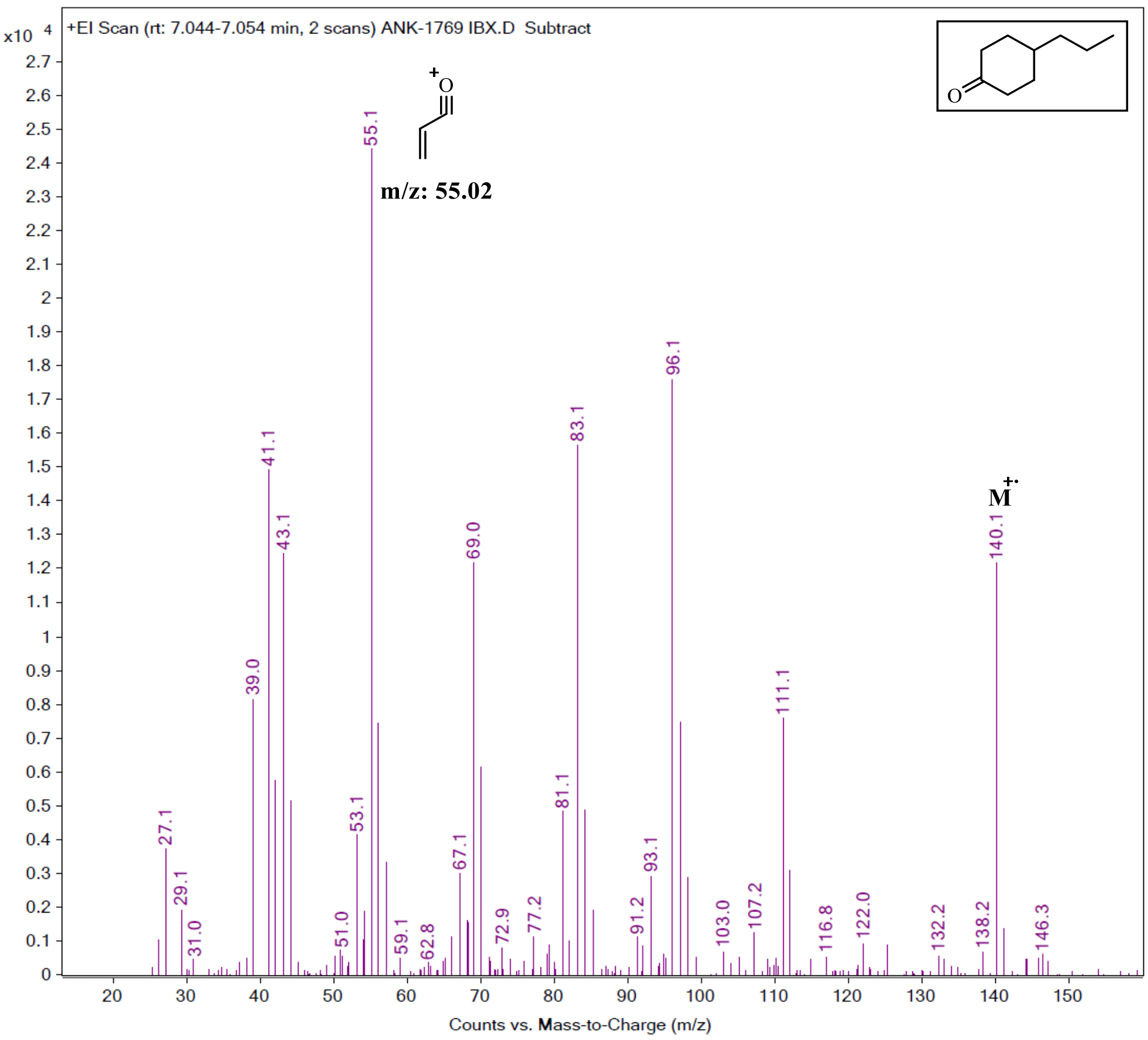
x10 4
+EI Scan (rt: 7.044-7.054 min, 2 scans) ANK-1769 IBX.D Subtract
m/z: 55.02
M+·
27.1
29.1
31.0
39.0
41.1
43.1
51.0
53.1
55.1
59.1
62.8
67.1
69.0
72.9
77.2
81.1
83.1
91.2
93.1
96.1
103.0
107.2
111.1
116.8
122.0
132.2
138.2
140.1
146.3
Counts vs. Mass-to-Charge (m/z)

Octyl (2,3,4,6-tetra-*O*-acetyl)-β-D-glucopyranoside (S18) oxidation

The oxidation was performed using octyl (2,3,4,6-tetra-*O*-acetyl)-β-D-glucopyranoside (8.52 mg). The following temperature-program was used: Rtx-5; 60 °C for 3 min, 15 °C/min to 250 °C and 250 °C for 5 min.

GC analysis of the **Mn(mcp)** catalyzed oxidation of octyl (2,3,4,6-tetra-*O*-acetyl)-β-D-glucopyranoside:

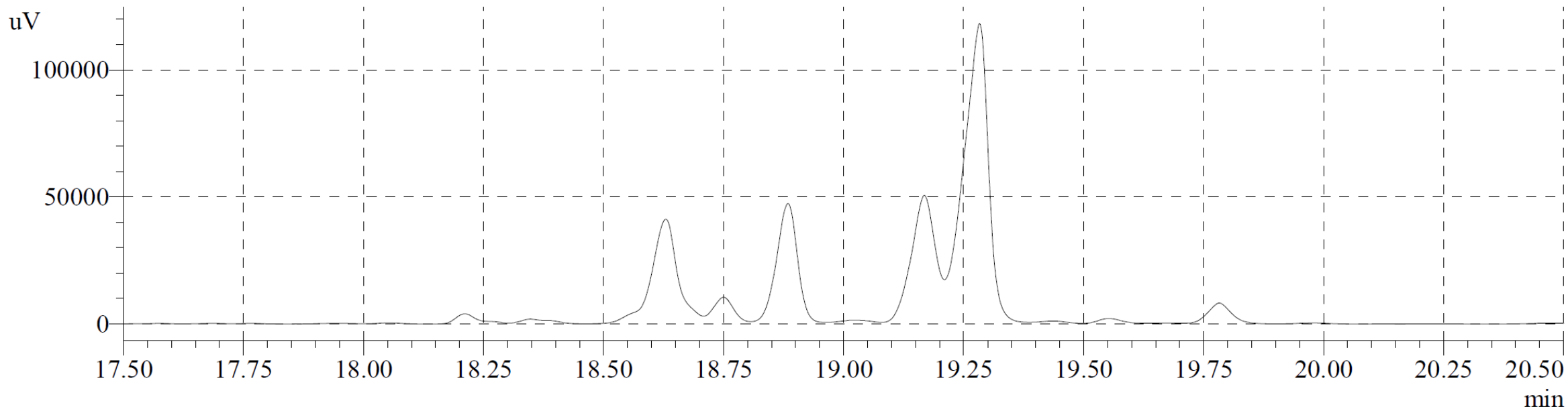

RRF(substrate): 1.333 RRF(ketone): 1.250

i	Retention time, min	Area (A)	Percentage (P), %	Percentage with RRF (P^{RRF}), %	Selectivity, %
-	17.125 (substrate)	665637	42.501	31.9	
-	sum of peaks 18.483-19.430	929380	59.342	47.5	
4	18.884	143102	9.1372	7.3	15.4
	9.027 (biphenyl)	313230	TON = 48; TOF = $5.3\cdot10^{-2}$ s^{-1}		
	Conversion: 68.1		Total product yield: 47.5		

GC analysis of the **5**-catalyzed oxidation of octyl (2,3,4,6-tetra-*O*-acetyl)-β-D-glucopyranoside:

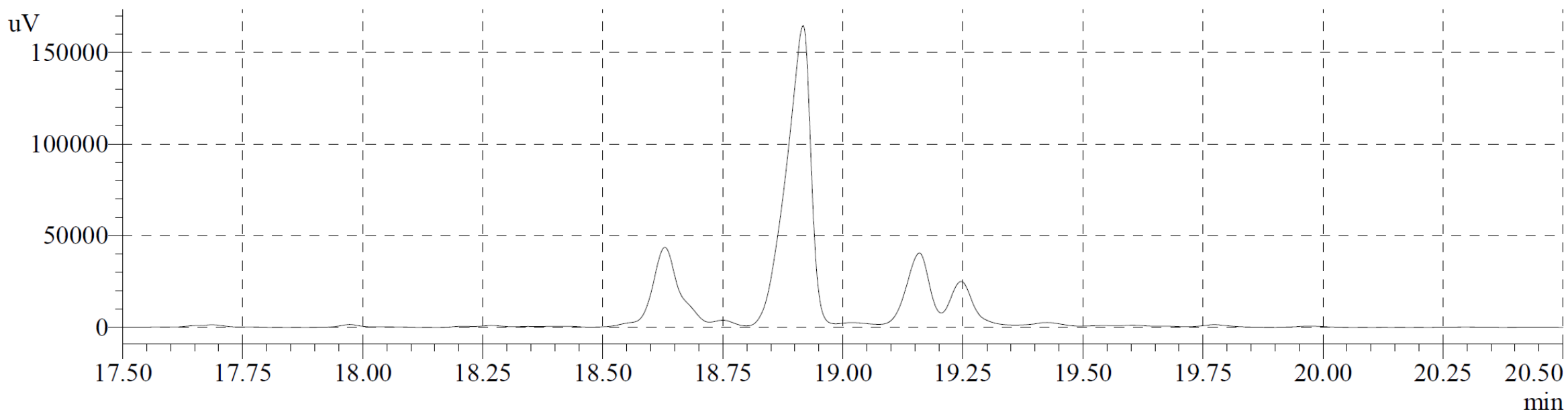

RRF(substrate): 1.333 RRF(ketone): 1.250

i	Retention time, min	Area (A)	Percentage (P), %	Percentage with RRF (P^{RRF}), %	Selectivity, %
-	17.111 (substrate)	445676	30.336	22.8	
-	sum of peaks 18.462-19.363	989438	67.348	53.9	
4	18.917	589031	40.094	32.0	59.5
	9.025 (biphenyl)	293826	TON = 27; TOF = $3.0\cdot10^{-2}$ s^{-1}		
	Conversion: 77.2		Total product yield: 53.9		

The main oxidation product of the **5**-catalyzed oxidation was isolated and characterized following a scaled-up synthesis.

Scaled-up synthesis (×10)

TFE (2.00 mL), substrate **S18** (85.2 mg, 185 µmol), 2,2-dimethylpropanoic acid (18.9 mg, 185 µmol) and 200 µL of TfOH solution in TFE (2.5 mol% of TfOH in relation to the substrate) were added to an oven-dried 12 mL vial charged with catalyst **5** (2.79 mg, 1.85 µmol) and a stirring bar. The mixture was cooled to 0 °C. A solution of an 50% aqueous H_2O_2 diluted in TFE (206 µL; stock solution: 100 µL 50 % aqueous H_2O_2 in 1.90 mL TFE) was slowly added over 15 minutes (13.7 µL/min) under stirring via

a syringe pump and after that the reaction mixture was stirred for additional 15 minutes. Additional portion of catalyst **5** (2.79 mg, 1.85 µmol) in TFE (500 µL). The addtion of H_2O_2 diluted in TFE was repeated and after that the reaction mixture was stirred for additional 15 minutes.

Work-up and IBX Oxidation: after the time indicated, H_2O (6.0 mL) was added and the mixture was extracted with HPLC grade EtOAc (2.0 mL × 3), washed with sat. $NaHCO_3$ (2.5 mL × 2), filtered through a silica plug (glass filter filled with 6.5 g of silica gel and 6.0 g of Na_2SO_4 on top) into a 100 mL one-neck round-bottom flask to remove catalyst **5** and rinsed with EtOAc (30 mL). Organic solvents were removed *in vacuo*, EtOAc (30 mL) was added and the flask was equipped with a stirring bar. IBX (124 mg, 444 µmol) was added and the mixture was heated to 66 °C (oil bath temperature) overnight. The mixture was allowed to cool down to room temperature, filtered through a silica plug (glass filter filled with 7.0 g of silica gel) into a 100 mL one-neck round-bottom flask to remove excess of IBX and byproducts and rinsed with EtOAc (20 mL). Organic solvents were evaporated to give crude material.

Isolation of the product: the product was isolated by preparative HPLC using the Reprospher 100 Si (particle size: 5 µm; dimensions: 250 × 25 mm) Dr. Maisch column. The crude was dissolved in 1 mL of cyclohexane : DCM = 10:1 mixture, filtered and eluted by cyclohexane/EtOH using isocratic program with 3% EtOH (flow rate: 15 mL/min, λ = 220 nm). Obtained fractions were checked by GC, in order to identify pure fractions of C4-oxidized compound **S18**. Pure fractions were combined, organic solvents were evaporated to give the product as white solid.

GC analysis of the pure isolated product:

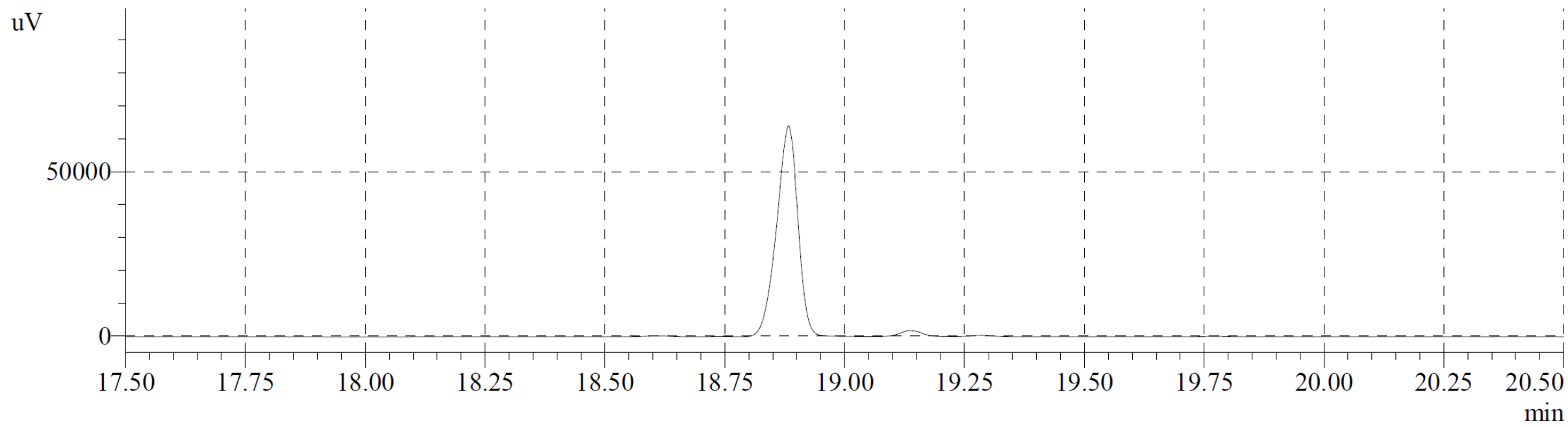

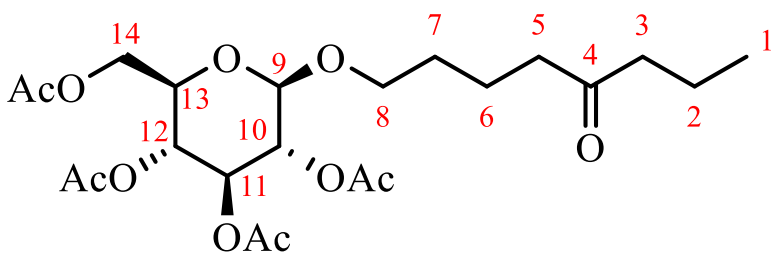

Chemical Formula: $C_{22}H_{34}O_{11}$
Molecular Weight: 474.50

Yield: 26 mg, 29% (96% GC purity).

Mp.: 59 – 60 °C.

IR (ID7, cm^{-1}) 2962 (w), 2939 (w), 2886 (w), 1754 (s), 1741 (s), 1708 (m), 1447 (w), 1428 (w), 1369 (m), 1241 (s), 1212 (s), 1169 (m), 1103 (w), 1064 (m), 1025 (s), 938 (w), 916 (w), 908 (w), 893 (w), 848 (w), 801 (w), 700 (w), 683 (w), 648 (w), 636 (w), 607 (w), 597 (w), 562 (w), 543 (w), 484 (w).

^{1}H NMR (500 MHz, $CDCl_3$) δ 5.19 (dd, ^3J = 9.7, ^3J = 9.3 Hz, 1H, C(11)H), 5.07 (dd, ^3J = 10.0, ^3J = 9.3 Hz, 1H, C(12)H), 4.97 (dd, ^3J = 9.7, ^3J = 8.0 Hz, 1H, C(10)H), 4.48 (d, ^3J = 8.0 Hz, 1H, C(9)H), 4.25 (dd, ^2J = 12.3, ^3J = 4.7 Hz, 1H, C(14)H_2), 4.13 (dd, ^2J = 12.3, ^3J = 2.5 Hz, 1H, C(14)H_2), 3.87 (dt, ^2J = 9.5, ^3J = 5.8 Hz, 1H, C(8)H_2), 3.68 (ddd, ^3J = 10.0, ^3J = 4.7, ^3J = 2.5 Hz, 1H, C(13)H), 3.47 (dt, ^2J = 9.5, ^3J = 6.4 Hz, 1H, C(8)H_2), 2.40 (t, ^3J = 6.8 Hz, 2H, C(5)H_2), 2.36 (t, ^3J = 7.3 Hz, 2H, C(3)H_2), 2.08 (s, 3H, MeCO), 2.04 (s, 3H, MeCO), 2.02 (s, 3H, MeCO), 2.00 (s, 3H, MeCO), 1.64 – 1.51 (m, 6H, C(2)H_2+C(6)H_2+C(7)H_2), 0.90 (t, ^3J = 7.4 Hz, 3H, Me(1)).

^{13}C NMR (150 MHz, $CDCl_3$) δ 210.9 (C(4)=O), 170.7 (Me*C*O), 170.3 (Me*C*O), 169.41 (Me*C*O), 169.38 (Me*C*O), 100.8 (C(9)H), 72.8 (C(11)H), 71.7 (C(13)H), 71.2 (C(10)H), 69.8 (C(8)H_2), 68.4 (C(12)H), 61.9 (C(14)H_2), 44.7 (C(3)H_2), 42.2 (C(5)H_2), 28.8 (C(7)H_2), 20.7 (*Me*CO), 20.64 (*Me*CO), 20.61 (*Me*CO), 20.6 (*Me*CO), 20.1 (C(6)H_2), 17.3 (C(2)H_2), 13.7 (Me(1)).

HRMS (ESI^+) *m/z* calcd for $C_{22}H_{38}NO_{11}$ $[M + NH_4]^+$: 492.2439; found 492.2442.

Heptyl 4-(pivaloyloxy)benzoate (S19) oxidation

The oxidation was performed using heptyl 4-(pivaloyloxy)benzoate (5.93 µL, 5.93 mg).
The following temperature-program was used: Rtx-5; 60 °C for 3 min, 15 °C/min to 250 °C and 250 °C for 5 min.

GC analysis of the **Mn(mcp)** catalyzed oxidation of heptyl 4-(pivaloyloxy)benzoate:

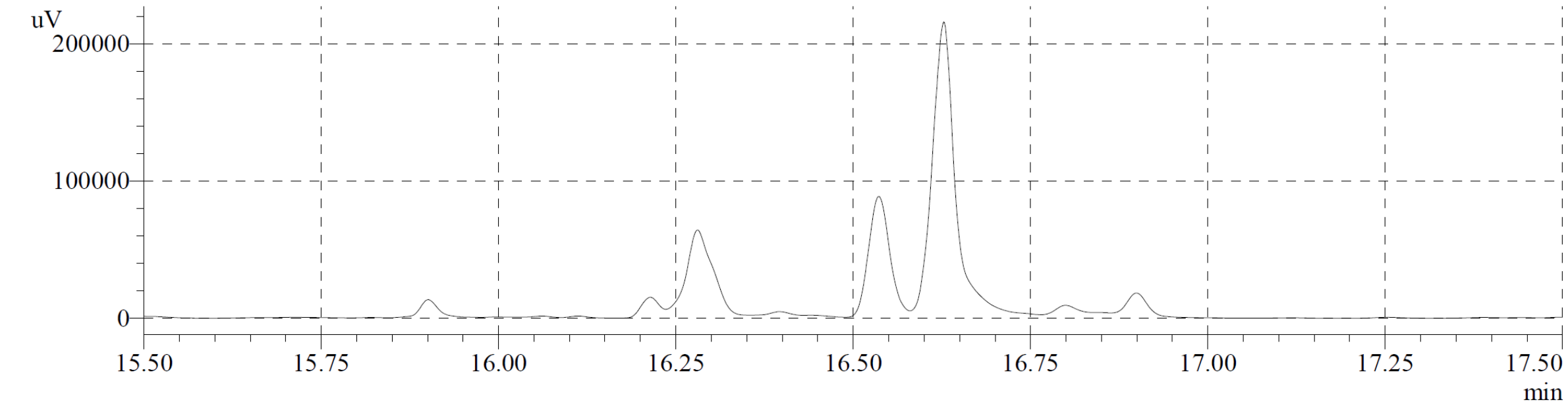

RRF(substrate): 1.417 RRF(ketone): 1.333

i	Retention time, min	Area (A)	Percentage (P), %	Percentage with RRF (P^{RRF}), %	Selectivity, %
-	15.265 (substrate)	792451	55.901	39.4	
-	sum of peaks 16.156-16.765	885461	62.462	46.8	
4	16.281	172163	12.145	9.1	19.4
	9.026 (biphenyl)	283521	TON = 47; TOF = $5.2\cdot10^{-2}$ s^{-1}		
	Conversion: 60.6		Total product yield: 46.8		

GC analysis of the **5**-catalyzed oxidation of heptyl 4-(pivaloyloxy)benzoate:

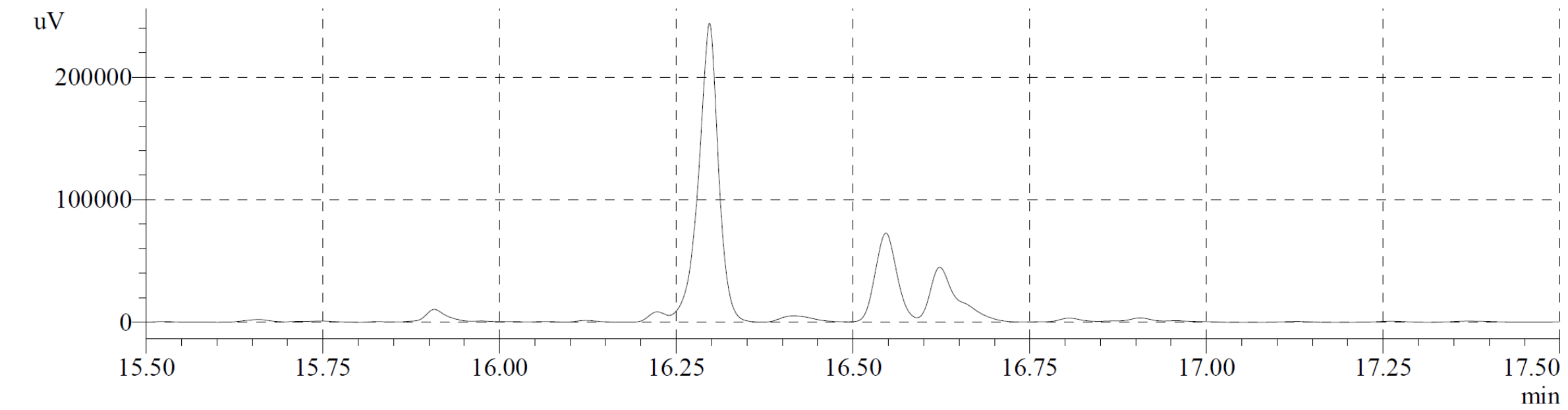

RRF(substrate): 1.417 RRF(ketone): 1.333

i	Retention time, min	Area (A)	Percentage (P), %	Percentage with RRF (P^{RRF}), %	Selectivity, %
-	15.271 (substrate)	821236	56.721	40.0	
-	sum of peaks 16.164-16.765	792095	54.708	41.0	
4	16.297	484227	33.444	25.1	61.1
	9.032 (biphenyl)	289571	TON = 20; TOF = $2.3\cdot10^{-2}$ s^{-1}		
	Conversion: 60.0		Total product yield: 41.0		

The main oxidation product of the **5**-catalyzed oxidation was isolated and characterized following a scaled-up synthesis.

Scaled-up synthesis (×10)

TFE (2.00 mL), substrate **S19** (59.3 µL, 59.3 mg, 185 µmol), 2,2-dimethylpropanoic acid (18.9 mg, 185 µmol) and 200 µL of TfOH solution in TFE (2.5 mol% of TfOH in relation to the substrate) were added to an oven-dried 12 mL vial charged with catalyst **5** (2.79 mg, 1.85 µmol) and a stirring bar. The mixture was cooled to 0 °C. A solution of an 50% aqueous H_2O_2 diluted in TFE (206 µL; stock solution: 100 µL 50 % aqueous H_2O_2 in 1.90 mL TFE) was slowly added over 15 minutes (13.7 µL/min) under stirring via a syringe pump and after that the reaction mixture was stirred for additional 15 minutes. Additional

portion of catalyst **5** (2.79 mg, 1.85 µmol) in TFE (500 µL). The addtion of H_2O_2 diluted in TFE was repeated and after that the reaction mixture was stirred for additional 15 minutes.

Work-up and IBX Oxidation: after the time indicated, H_2O (6.0 mL) was added and the mixture was extracted with HPLC grade EtOAc (2.0 mL × 3), washed with sat. $NaHCO_3$ (2.5 mL × 2), filtered through a silica plug (glass filter filled with 6.5 g of silica gel and 6.0 g of Na_2SO_4 on top) into a 100 mL one-neck round-bottom flask to remove catalyst **5** and rinsed with EtOAc (30 mL). Organic solvents were removed *in vacuo*, EtOAc (30 mL) was added and the flask was equipped with a stirring bar. IBX (124 mg, 444 µmol) was added and the mixture was heated to 66 °C (oil bath temperature) overnight. The mixture was allowed to cool down to room temperature, filtered through a silica plug (glass filter filled with 7.0 g of silica gel) into a 100 mL one-neck round-bottom flask to remove excess of IBX and byproducts and rinsed with EtOAc (20 mL). Organic solvents were evaporated to give crude material.

Isolation of the product: the product was isolated by preparative HPLC using the Reprospher 100 Si (particle size: 5 µm; dimensions: 250 × 25 mm) Dr. Maisch column. The crude was dissolved in 1 mL of cyclohexane, filtered and eluted by cyclohexane/MTBE using isocratic program with 7.5% MTBE (flow rate: 15 mL/min, λ = 220 nm). Obtained fractions were checked by GC, in order to identify pure fractions of C4-oxidized compound **S19**. Pure fractions were combined, organic solvents were evaporated to give the product as colourless oil.

GC analysis of the pure isolated product:

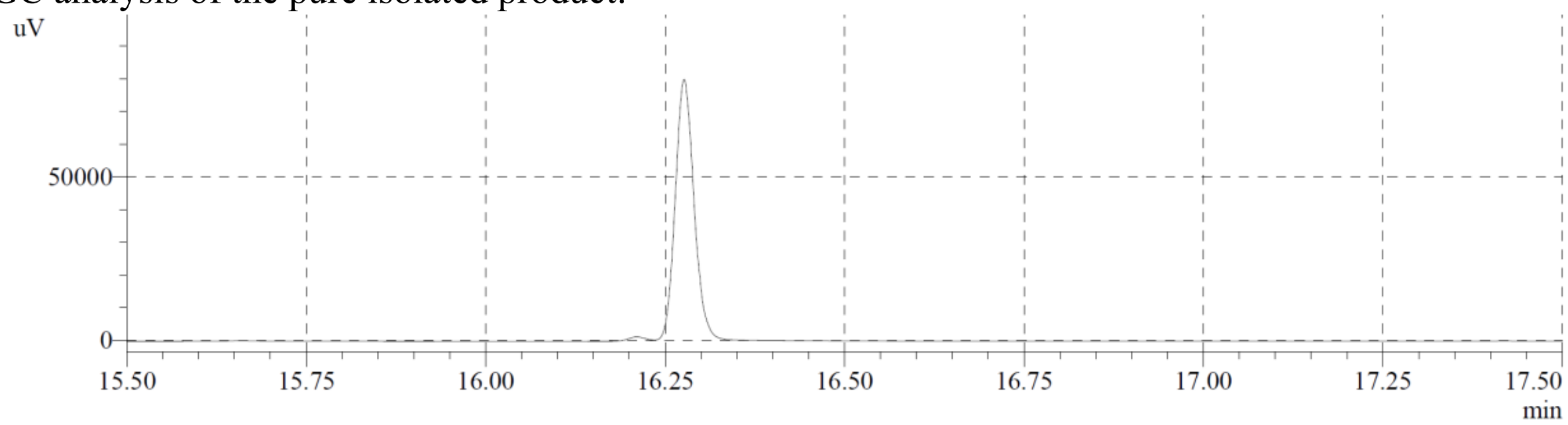

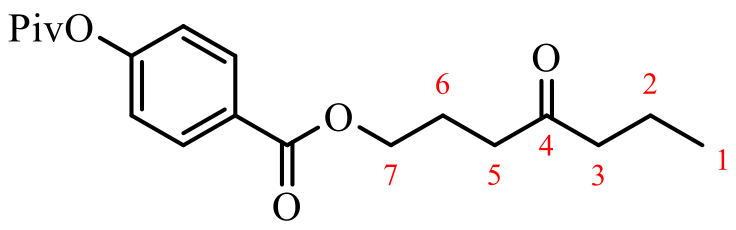

Chemical Formula: $C_{19}H_{26}O_5$
Molecular Weight: 334.41

Yield: 23 mg, 36% (99% GC purity).

IR (ID7, cm^{-1}) 2964 (m), 2935 (w), 2906 (w), 2876 (w), 1752 (m), 1713 (s), 1603 (m), 1505 (w), 1480 (w), 1461 (w), 1412 (w), 1369 (w), 1270 (s), 1202 (s), 1161 (s), 1091 (s), 1015 (s), 900 (m), 860 (w), 757 (m), 692 (w), 650 (w), 585 (w), 504 (w).

^{1}H NMR (500 MHz, $CDCl_3$) δ 8.04 (d, ^3J = 8.7 Hz, 2H, 2×CH, Ar), 7.13 (d, ^3J = 8.7 Hz, 2H, 2×CH, Ar), 4.32 (t, ^3J = 6.4 Hz, 2H, C(7)H_2), 2.56 (t, ^3J = 7.2 Hz, 2H, C(5)H_2), 2.40 (t, ^3J = 7.4 Hz, 2H, C(3)H_2), 2.08 – 2.03 (m, 2H, C(6)H_2), 1.65 – 1.57 (m, 2H, C(2)H_2), 1.36 (s, 9H, Me_3C), 0.91 (t, ^3J = 7.4 Hz, 3H, Me(1)).

^{13}C NMR (125 MHz, $CDCl_3$) δ 209.9 (C(4)=O), 176.5 (C=O), 165.8 (C=O), 154.8 (C, Ar), 131.0 (2×CH, Ar), 127.5 (C, Ar), 121.6 (2×CH, Ar), 64.3 (C(7)H_2), 44.8 (C(3)H_2), 39.2 (Me_3*C*), 38.9 (C(5)H_2), 27.0 (*Me*$_3$C), 22.8 (C(6)H_2), 17.3 (C(2)H_2), 13.7 (Me(1)).

HRMS (ESI$^+$) *m/z* calcd for $C_{19}H_{30}NO_5$ [M + NH_4]$^+$: 352.2118; found 352.2117.

Scaled-up synthesis (×30)

The synthesis was done based on previously tested conditions (section 3.2, entry SI-9).

TFE (6.00 mL), substrate **S19** (178 µL, 178 mg, 555 µmol), 2,2-dimethylpropanoic acid (56.7 mg, 555 µmol) and 600 µL of TfOH solution in TFE (2.5 mol% of TfOH in relation to the substrate) were added to an oven-dried 25 mL flask charged with catalyst **5** (8.36 mg, 5.55 µmol) and a stirring bar. The mixture was cooled to 0 °C. A solution of an 50% aqueous H_2O_2 diluted in TFE (617 µL; stock solution: 100 µL 50 % aqueous H_2O_2 in 1.90 mL TFE) was slowly added over 45 minutes (13.7 µL/min) under

stirring via a syringe pump and after that the reaction mixture was stirred for additional 15 minutes. Additional portion of catalyst **5** (8.36 mg, 5.55 µmol) in TFE (1.50 mL). The addtion of H_2O_2 diluted in TFE was repeated and after that the reaction mixture was stirred for additional 15 minutes.

Work-up and IBX Oxidation: after the time indicated, H_2O (15.0 mL) was added and the mixture was extracted with HPLC grade EtOAc (7.0 mL × 4), washed with sat. $NaHCO_3$ (30 mL × 1) and aqueous pase was extracted with EtOAc (5.0 mL × 2). The organic fractions were filtered through a silica plug (glass filter filled with 6.5 g of silica gel and 6.0 g of Na_2SO_4 on top) into a 250 mL one-neck round-bottom flask to remove catalyst **5** and rinsed with EtOAc (50 mL). Organic solvents were removed *in vacuo*, EtOAc (80 mL) was added and the flask was equipped with a stirring bar. IBX (373 mg, 1.33 mmol) was added and the mixture was heated to 66 °C (oil bath temperature) overnight. The mixture was allowed to cool down to room temperature, filtered through a silica plug (glass filter filled with 7.0 g of silica gel) into a 250 mL one-neck round-bottom flask to remove excess of IBX and byproducts and rinsed with EtOAc (50 mL). Organic solvents were evaporated to give crude material.

Isolation of the product: the product was isolated by preparative HPLC using the Reprospher 100 Si (particle size: 5 µm; dimensions: 250 × 25 mm) Dr. Maisch column. The crude was dissolved in 2 mL of cyclohexane, filtered and eluted by cyclohexane/MTBE using isocratic program with 7.5% MTBE (flow rate: 15 mL/min, λ = 220 nm). Obtained fractions were checked by GC, in order to identify pure fractions of C4-oxidized compound **S19**. Pure fractions were combined, organic solvents were evaporated to give the product as colourless oil.

GC analysis of the pure isolated product:

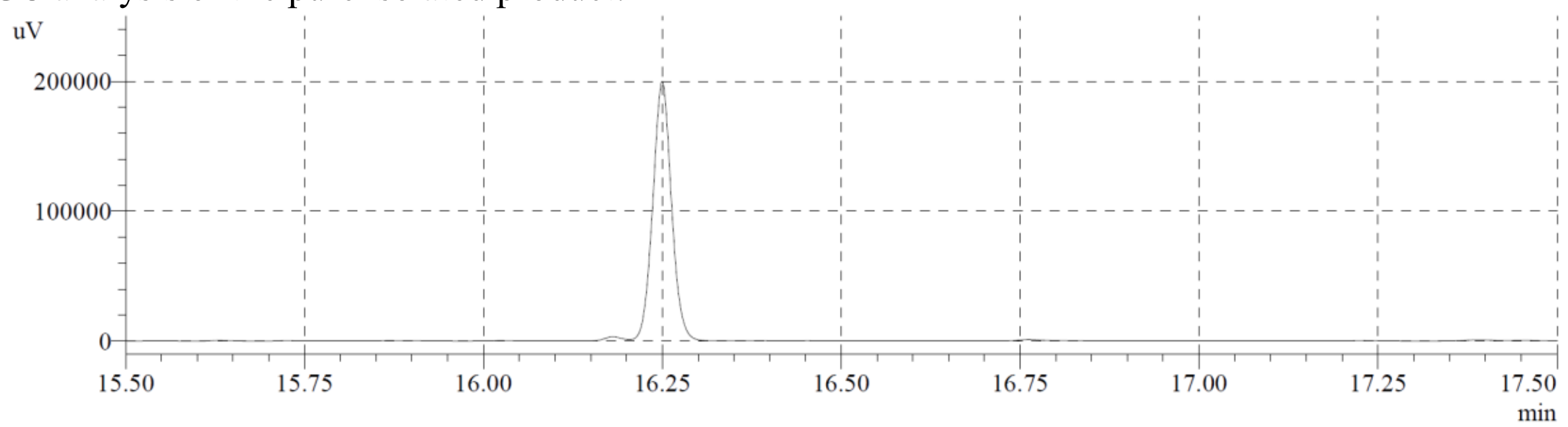

Yield: 51 mg, 28% (98% GC purity).

5-Heptyldihydrofuran-2(3*H*)-one (S20) oxidation

The oxidation was performed using 5-heptyldihydrofuran-2(3*H*)-one (3.62 µL, 3.41 mg).
The following temperature-program was used: Rtx-5; 60 °C for 3 min, 15 °C/min to 250 °C and 250 °C for 5 min.

GC analysis of the **Mn(mcp)** catalyzed oxidation of 5-heptyldihydrofuran-2(3*H*)-one:

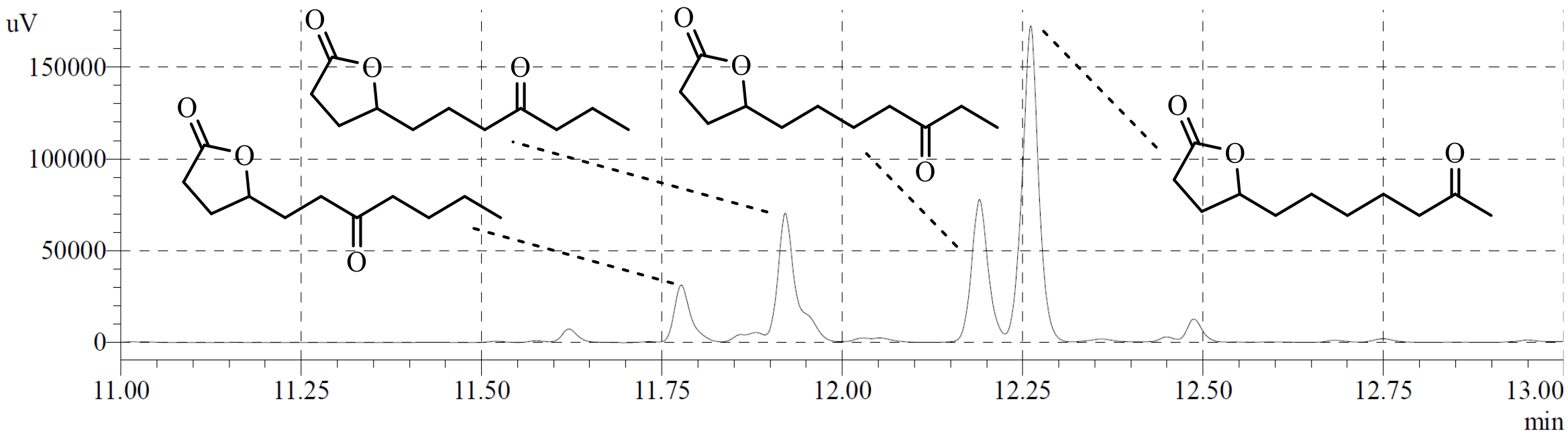

RRF(substrate): 0.833 RRF(ketone): 0.750

i	Retention time, min	Area (A)	Percentage (P), %	Percentage with RRF (P^{RRF}), %	Selectivity, %
-	10.685 (substrate)	389695	27.530	33.0	
5	11.777	50624	3.5763	4.8	9.1
4	11.921	104537	7.3850	9.8	18.8
3	12.190	126673	8.9488	11.9	22.8
2	12.261	274023	19.358	25.8	49.3
	9.026 (biphenyl)	283105	TON = 52; TOF = $5.8 \cdot 10^{-2}$ s^{-1}		
	Conversion: 67.0		Total product yield: 52.3		

GC analysis of the **5**-catalyzed oxidation of 5-heptyldihydrofuran-2(3*H*)-one:

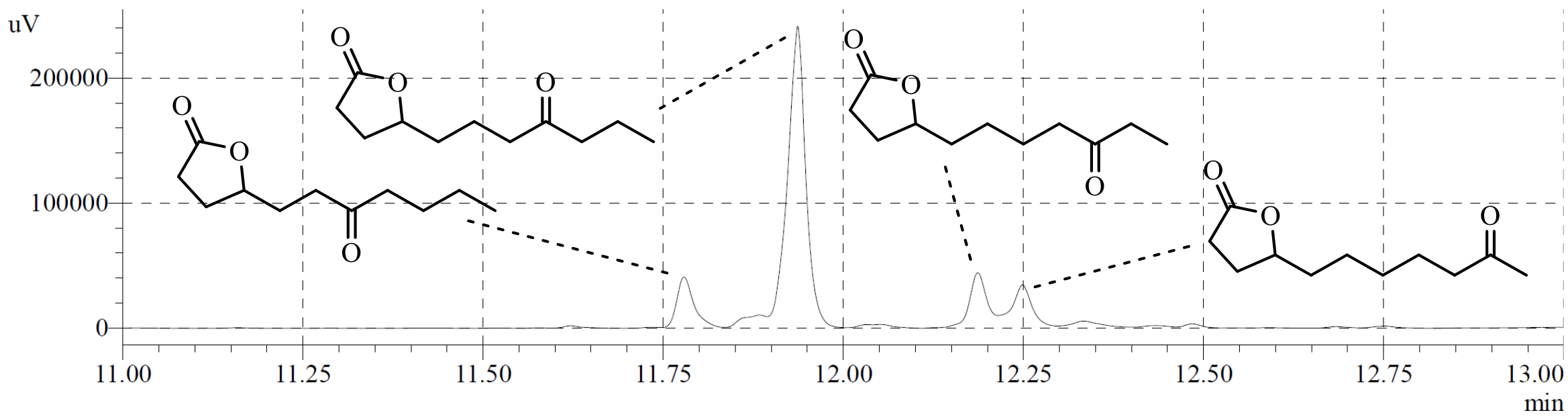

RRF(substrate): 0.833 RRF(ketone): 0.750

i	Retention time, min	Area (A)	Percentage (P), %	Percentage with RRF (P^{RRF}), %	Selectivity, %
-	10.682 (substrate)	267111	19.211	23.0	
5	11.779	69389	4.9906	6.6	11.0
4	11.936	426102	30.646	40.9	67.4
3	12.187	71801	5.1641	6.9	11.4
2	12.249	64960	4.6721	6.2	10.3
	9.027 (biphenyl)	278078	TON = 30; TOF = $3.4 \cdot 10^{-2}$ s^{-1}		
	Conversion: 77.0		Total product yield: 60.6		

GC-MS analyses of the **Mn(mcp)** (top) and **5**-catalyzed (bottom) oxidation of 5-heptyldihydrofuran-2(3*H*)-one. The different oxidation products were assigned according to their fragmentation pattern.

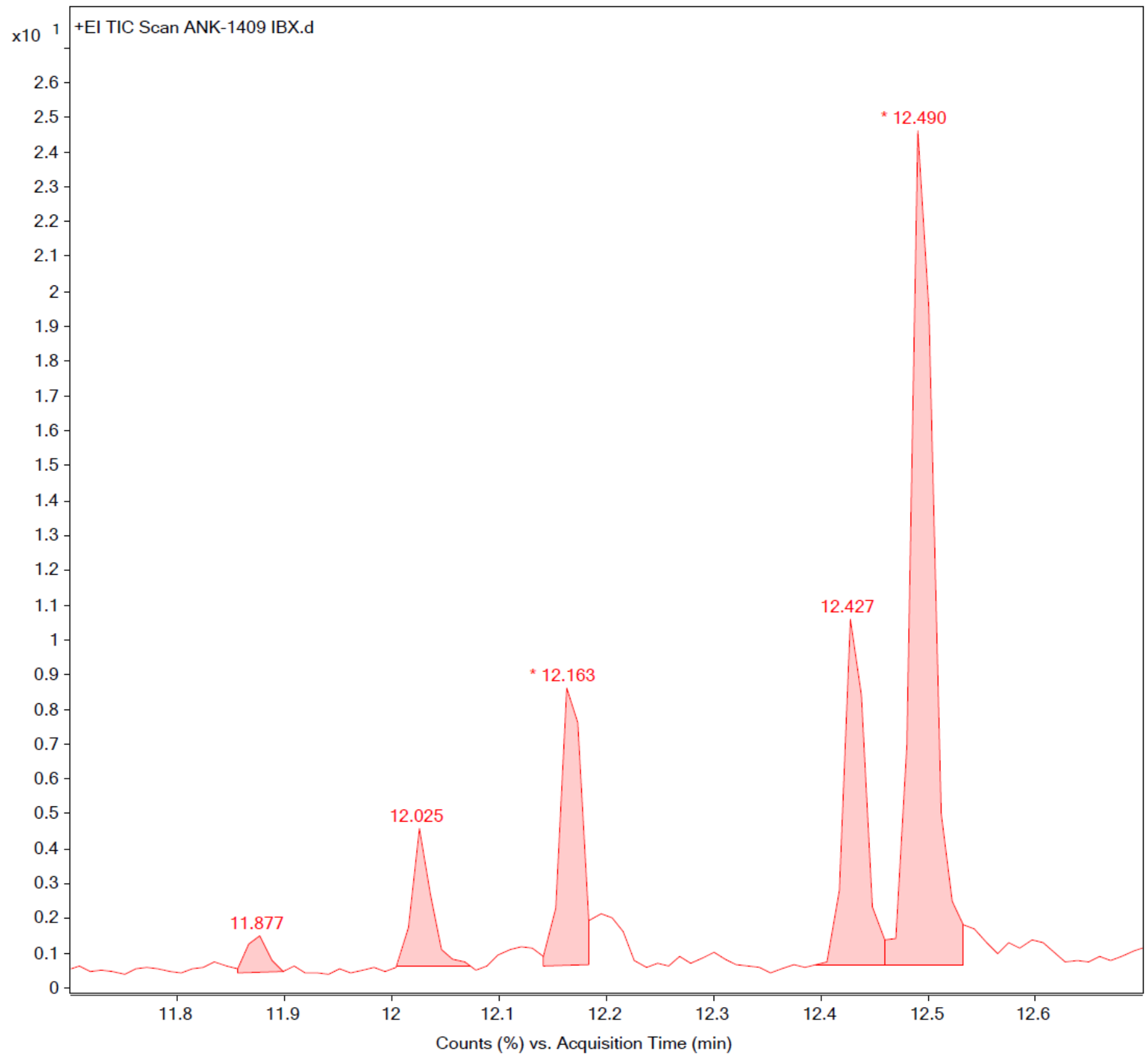

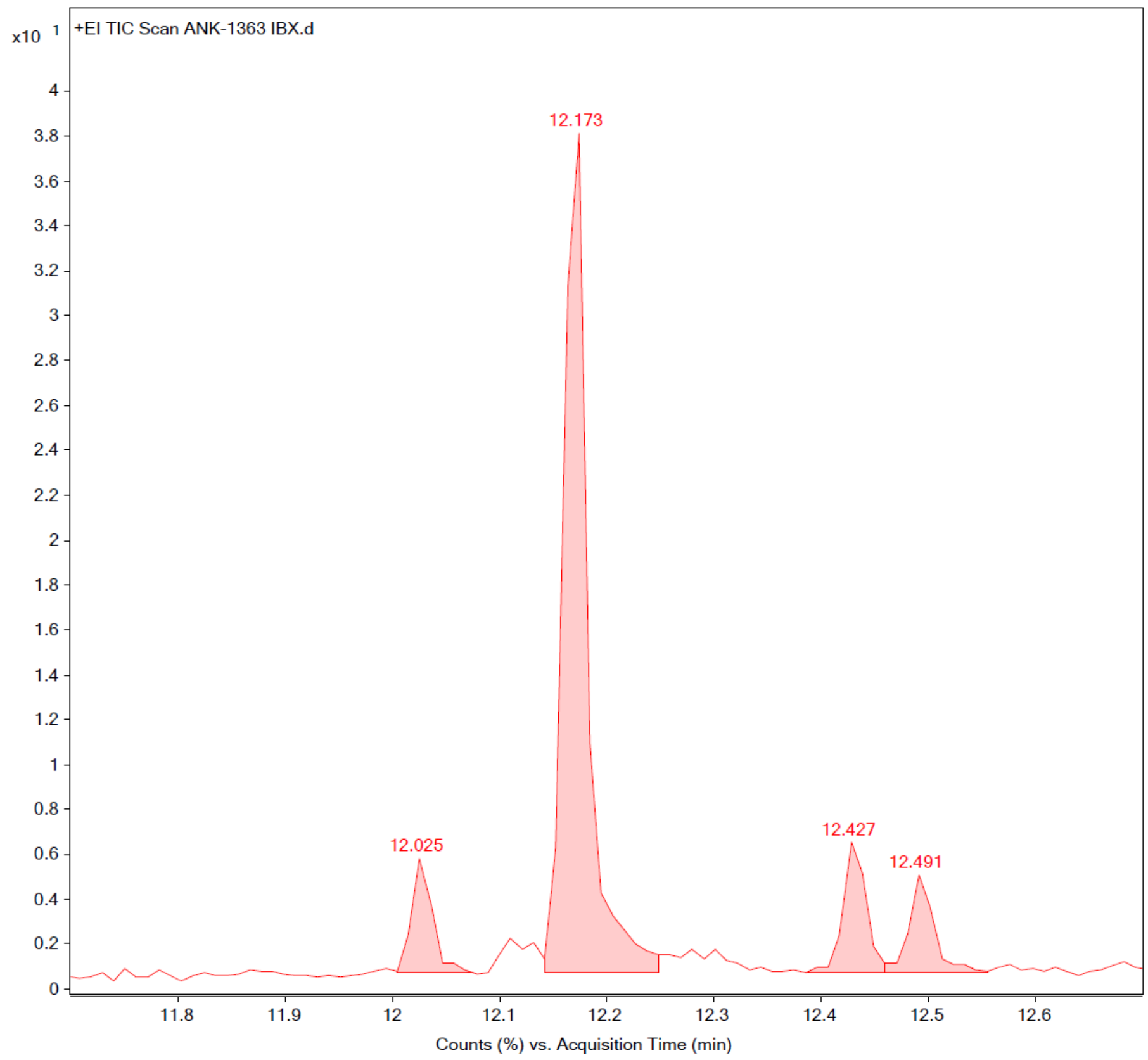

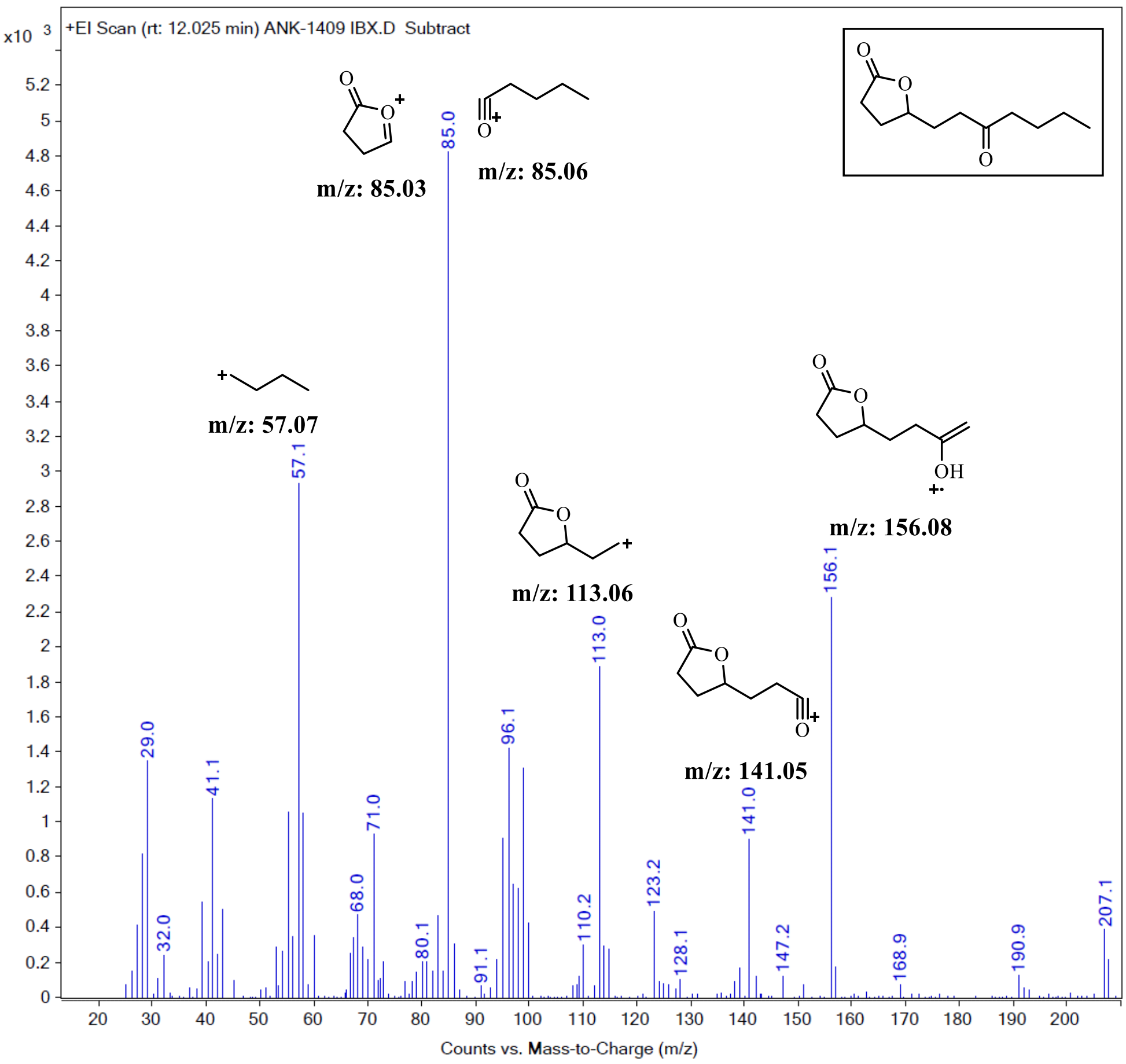

+EI Scan (rt: 12.025 min) ANK-1409 IBX.D Subtract
x10 3
m/z: 85.03
m/z: 85.06
m/z: 57.07
m/z: 113.06
m/z: 156.08
m/z: 141.05
Counts vs. Mass-to-Charge (m/z)

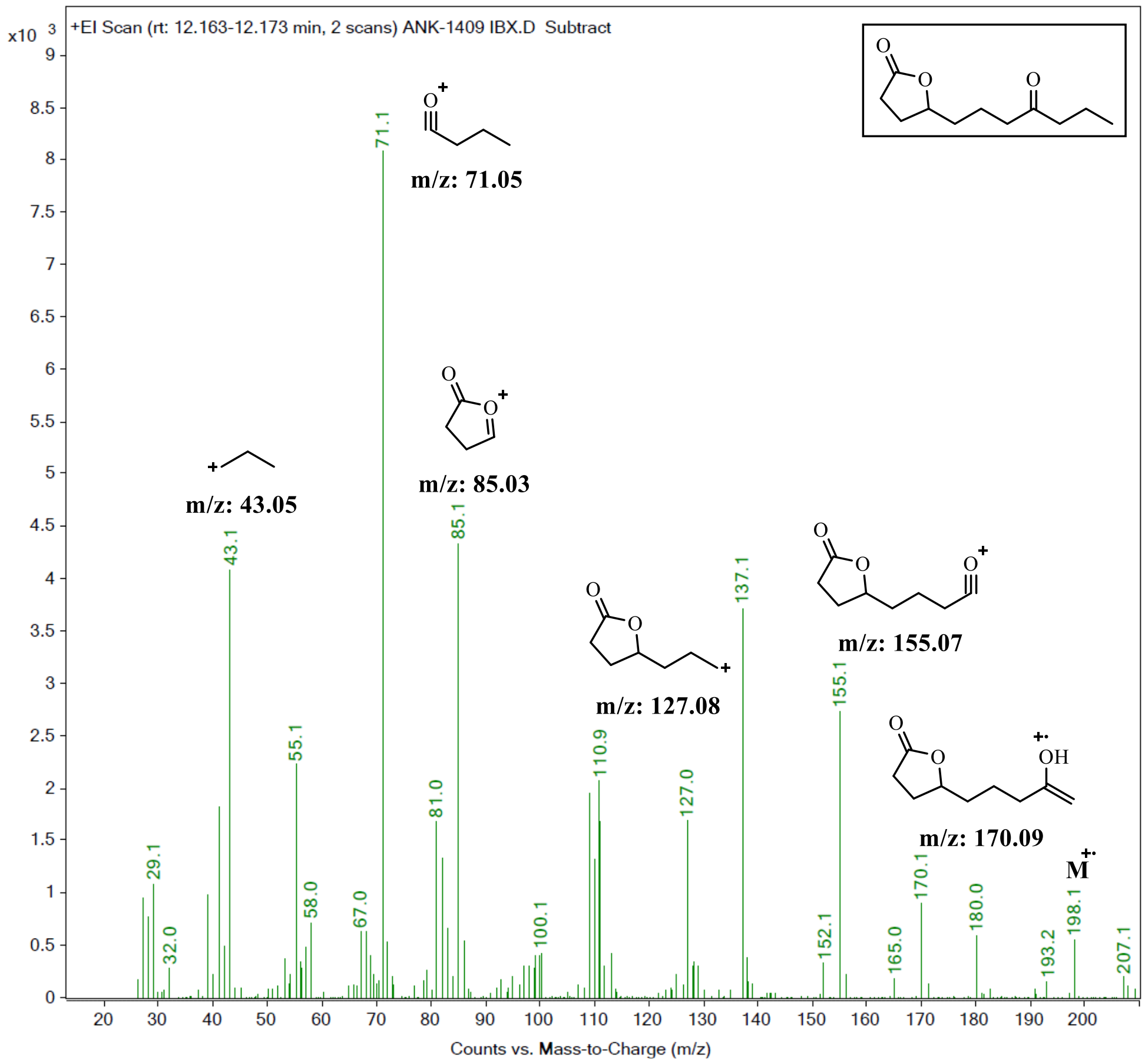

+EI Scan (rt: 12.163-12.173 min, 2 scans) ANK-1409 IBX.D Subtract
x10 3
m/z: 71.05
m/z: 85.03
m/z: 43.05
m/z: 155.07
m/z: 127.08
m/z: 170.09
M
Counts vs. Mass-to-Charge (m/z)

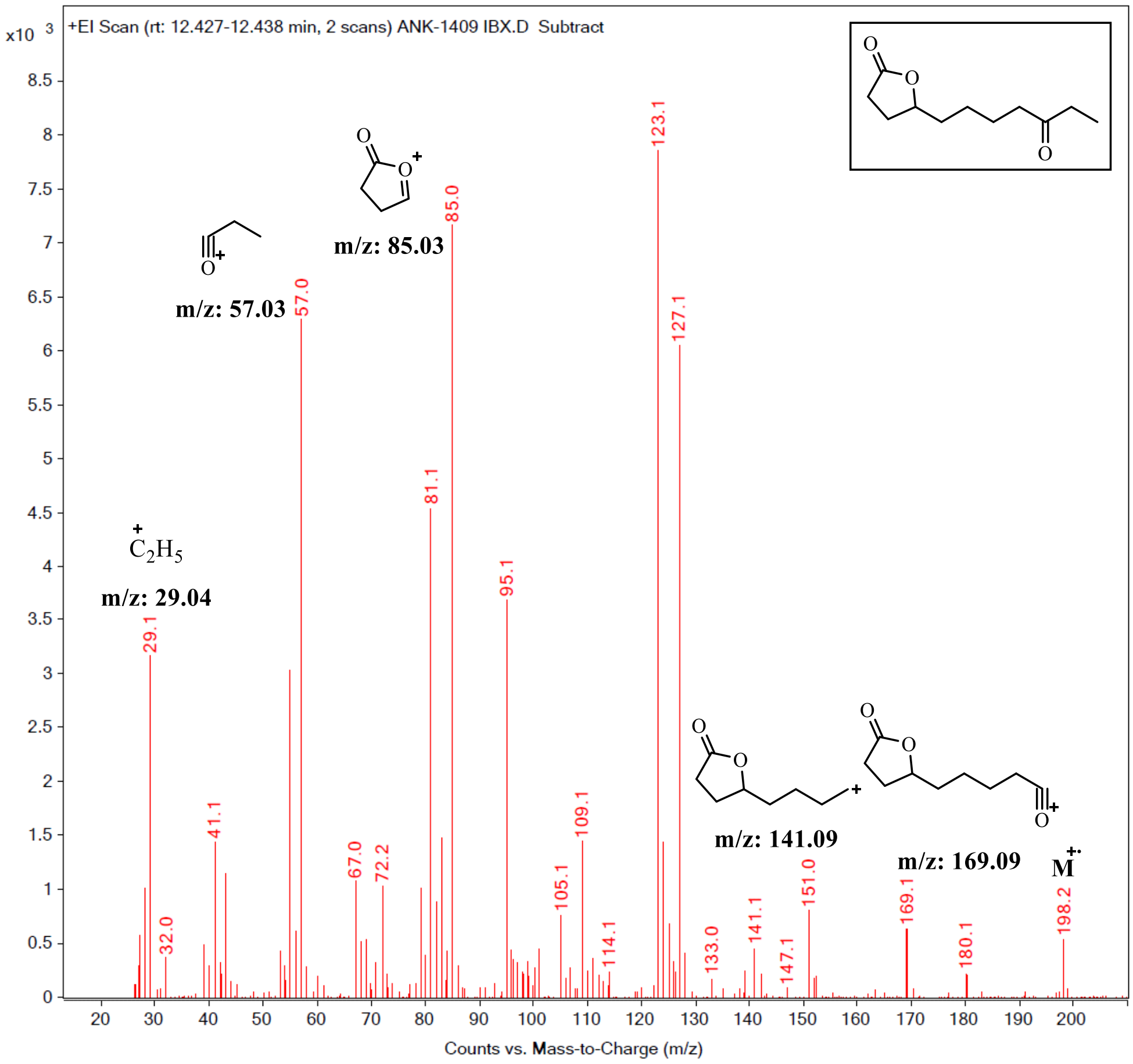
+EI Scan (rt: 12.427-12.438 min, 2 scans) ANK-1409 IBX.D Subtract
x10 3
m/z: 29.04
m/z: 57.03
m/z: 85.03
m/z: 141.09
m/z: 169.09
M+·
Counts vs. Mass-to-Charge (m/z)

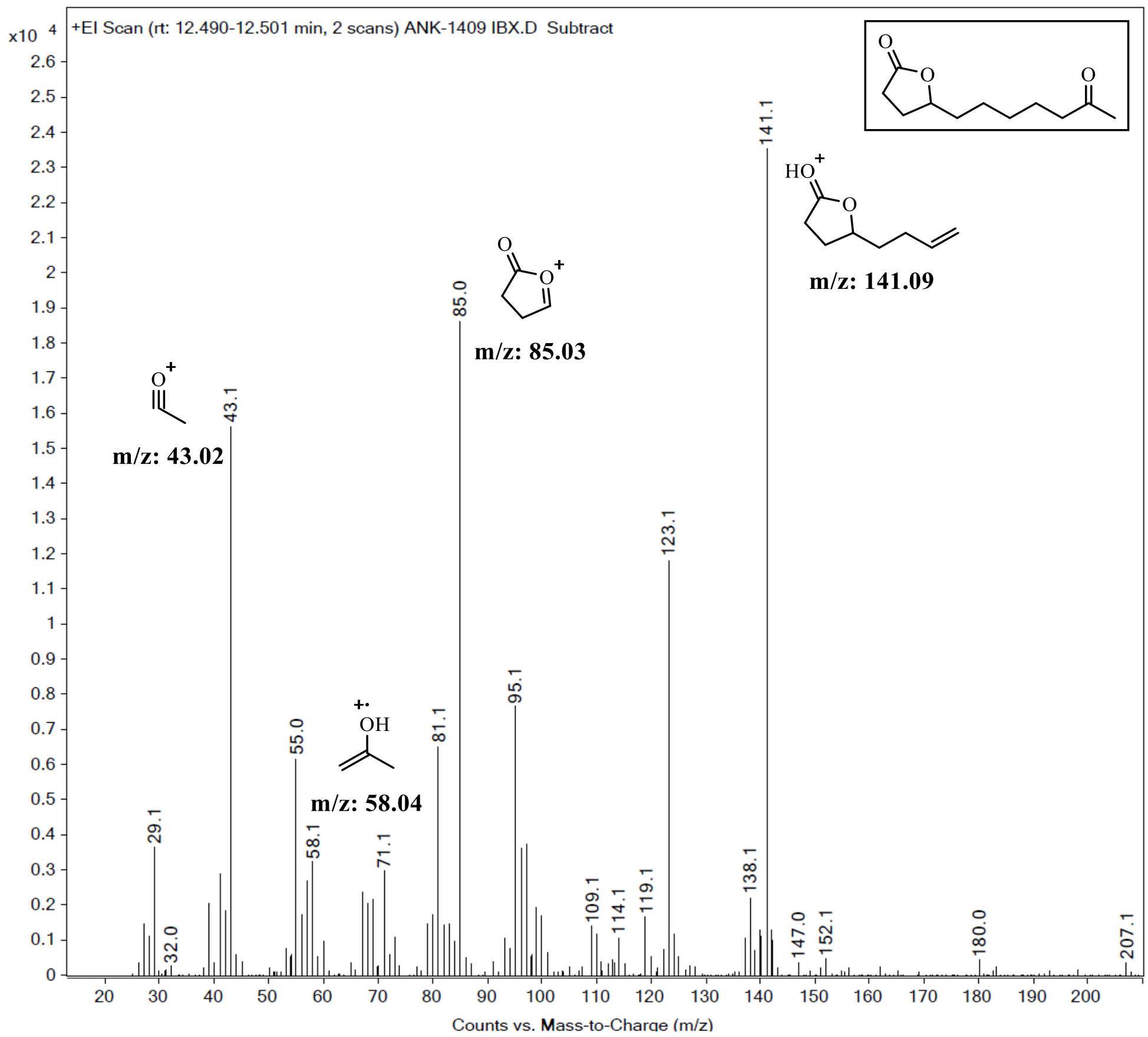
+EI Scan (rt: 12.490-12.501 min, 2 scans) ANK-1409 IBX.D Subtract
x10 4
m/z: 43.02
m/z: 58.04
m/z: 85.03
m/z: 141.09
Counts vs. Mass-to-Charge (m/z)

2-Undecanone (S21) oxidation

The oxidation was performed using 2-undecanone (3.80 µL, 3.15 mg).
The following temperature-program was used: Rtx-5; 60 °C for 3 min, 15 °C/min to 250 °C and 250 °C for 5 min.

GC analysis of the **Mn(mcp)** catalyzed oxidation of 2-undecanone:

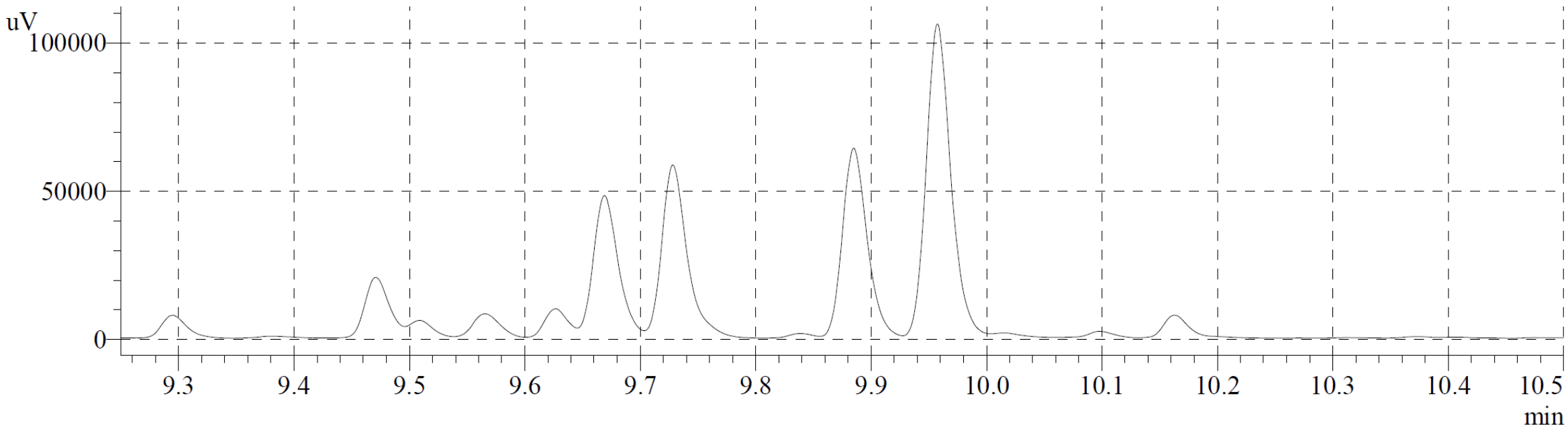

RRF(substrate): 0.833 RRF(ketone): 0.750

i	Retention time, min	Area (A)	Percentage (P), %	Percentage with RRF (P^{RRF}), %	Selectivity, %
-	8.168 (substrate)	435999	30.480	36.6	
-	sum of peaks 9.430-10.061	488715	34.166	45.6	
4	9.728	89241	6.2388	8.3	18.3
	9.015 (biphenyl)	286086	TON = 46; TOF = $5.1 \cdot 10^{-2}$ s^{-1}		
	Conversion: 63.4		Total product yield: 45.6		

GC analysis of the **5**-catalyzed oxidation of 2-undecanone:

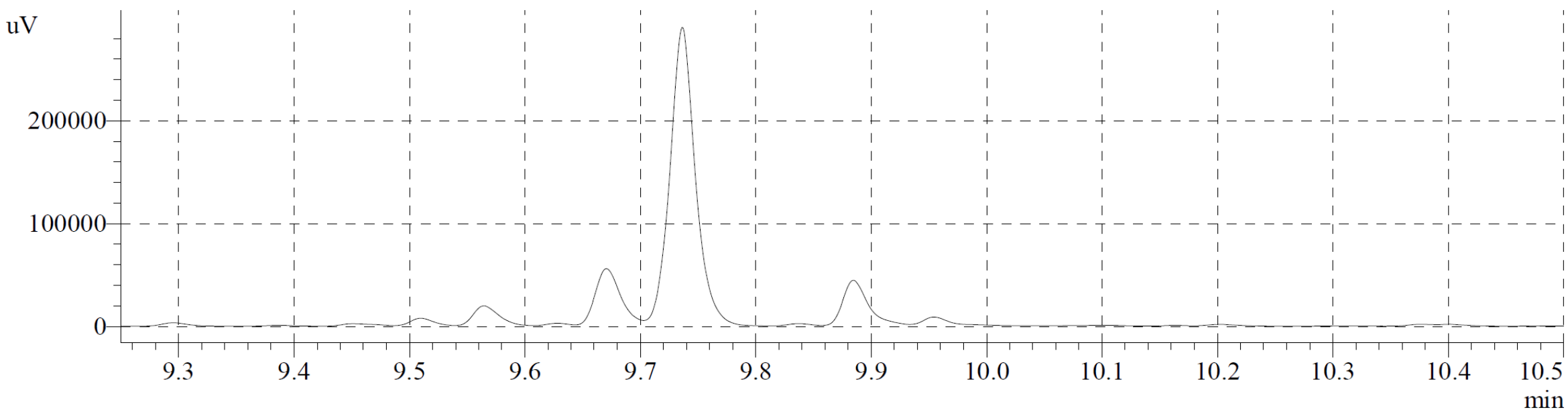

RRF(substrate): 0.833 RRF(ketone): 0.750

i	Retention time, min	Area (A)	Percentage (P), %	Percentage with RRF (P^{RRF}), %	Selectivity, %
-	8.166 (substrate)	202914	14.742	17.7	
-	sum of peaks 9.431-10.063	670907	48.742	65.0	
4	9.736	446857	32.464	43.3	66.6
	9.016 (biphenyl)	275289	TON = 33; TOF = $3.6 \cdot 10^{-2}$ s^{-1}		
	Conversion: 82.3		Total product yield: 65.0		

The main oxidation product of the **5**-catalyzed oxidation was isolated and characterized following a scaled-up synthesis.

Scaled-up synthesis (×10)

TFE (2.00 mL), substrate **S21** (38.0 µL, 31.5 mg, 185 µmol), 2,2-dimethylpropanoic acid (18.9 mg, 185 µmol) and 200 µL of TfOH solution in TFE (2.5 mol% of TfOH in relation to the substrate) were added to an oven-dried 12 mL vial charged with catalyst **5** (2.79 mg, 1.85 µmol) and a stirring bar. The mixture was cooled to 0 °C. A solution of an 50% aqueous H_2O_2 diluted in TFE (206 µL; stock solution: 100 µL 50 % aqueous H_2O_2 in 1.90 mL TFE) was slowly added over 15 minutes (13.7 µL/min) under stirring via a syringe pump and after that the reaction mixture was stirred for additional 15 minutes. Additional

portion of catalyst **5** (2.79 mg, 1.85 µmol) in TFE (500 µL). The addtion of H_2O_2 diluted in TFE was repeated and after that the reaction mixture was stirred for additional 15 minutes.

Work-up and IBX Oxidation: after the time indicated, H_2O (6.0 mL) was added and the mixture was extracted with HPLC grade EtOAc (3.0 mL × 2), washed with sat. $NaHCO_3$ (2.0 mL), dried over Na_2SO_4, filtered through a silica plug and rinsed with EtOAc. The organic phase was transferred into a one-neck round-bottom flask equipped with a stirring bar. IBX (124 mg, 444 µmol) was added and the mixture was heated to 66 °C overnight. The mixture was allowed to cool down to room temperature, filtered through a silica plug and rinsed with EtOAc. Organic solvents were evaporated to give a crude material.

Isolation of the product: the product was isolated by preparative HPLC using the Reprospher 100 Si (particle size: 5 µm; dimensions: 250 × 25 mm) Dr. Maisch column. The crude was dissolved in 1 mL of hexane, filtered and eluted by hexane/MTBE using isocratic program with 20% MTBE (flow rate: 15 mL/min, λ = 220, 256 nm). Obtained fractions were checked by GC, in order to identify pure fractions of C4-oxidized compound **S21**. Pure fractions were combined, organic solvents were evaporated to give the product as white solid.

GC analysis of the pure isolated product:

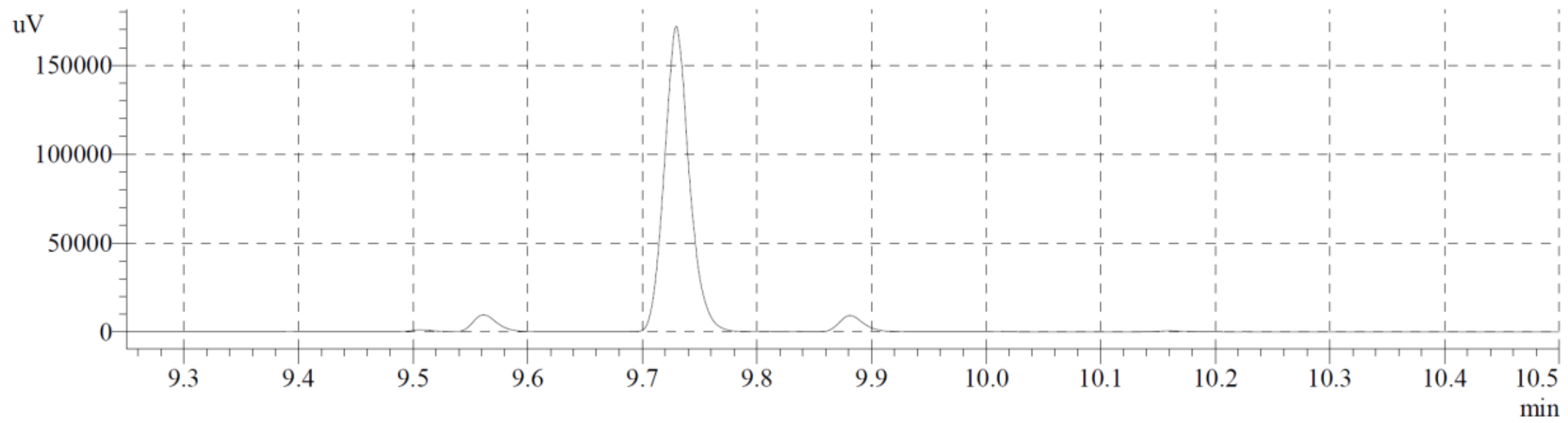

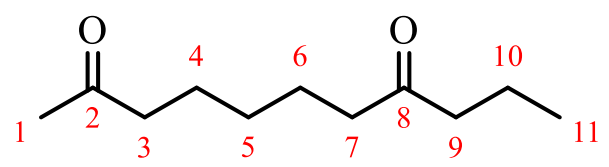

Chemical Formula: $C_{11}H_{20}O_2$
Molecular Weight: 184.28

Yield: 12 mg, 35% (89% GC purity).

Mp.: 38 – 39 °C.

IR (ID7, cm^{-1}) 2960 (m), 2933 (s), 2873 (m), 1702 (s), 1463 (w), 1414 (m), 1358 (s), 1278 (w), 1223 (s), 1167 (s), 1128 (s), 1101 (w), 1052 (w), 1002 (w), 951 (w), 900 (w), 743 (w), 716 (w), 683 (w), 597 (m), 492 (w).

^{1}H NMR (500 MHz, $CDCl_3$) δ 2.42 (t, ^3J = 7.4 Hz, 2H, C(3)H$_2$), 2.38 (t, ^3J = 7.4 Hz, 2H, C(7)H$_2$), 2.36 (t, ^3J = 7.4 Hz, 2H, C(9)H$_2$), 2.12 (s, 3H, Me(1)), 1.63 – 1.53 (m, 6H, C(4)H$_2$+C(6)H$_2$+C(10)H$_2$), 1.31 – 1.23 (m, 2H, C(5)H$_2$), 0.90 (t, ^3J = 7.4 Hz, 3H, Me(11)).

^{13}C NMR (125 MHz, $CDCl_3$) δ 211.2 (C(8)=O), 209.0 (C(2)=O), 44.7 (C(9)H$_2$), 43.4 (C(3)H$_2$), 42.4 (C(7)H$_2$), 29.9 (Me(1)), 28.6 (C(5)H$_2$), 23.5 (C(4)H$_2$), 23.4 (C(6)H$_2$), 17.3 (C(10)H$_2$), 13.7 (Me(11)).

HRMS (ESI$^+$) *m/z* calcd for $C_{11}H_{21}O_2$ [M + H]$^+$: 185.1536; found 185.1538.

Methyl decanoate (S22) oxidation

The oxidation was performed using methyl decanoate (3.95 µL, 3.45 mg).
The following temperature-program was used: Rtx-5; 60 °C for 3 min, 15 °C/min to 250 °C and 250 °C for 5 min.

GC analysis of the **Mn(mcp)** catalyzed oxidation of methyl decanoate:

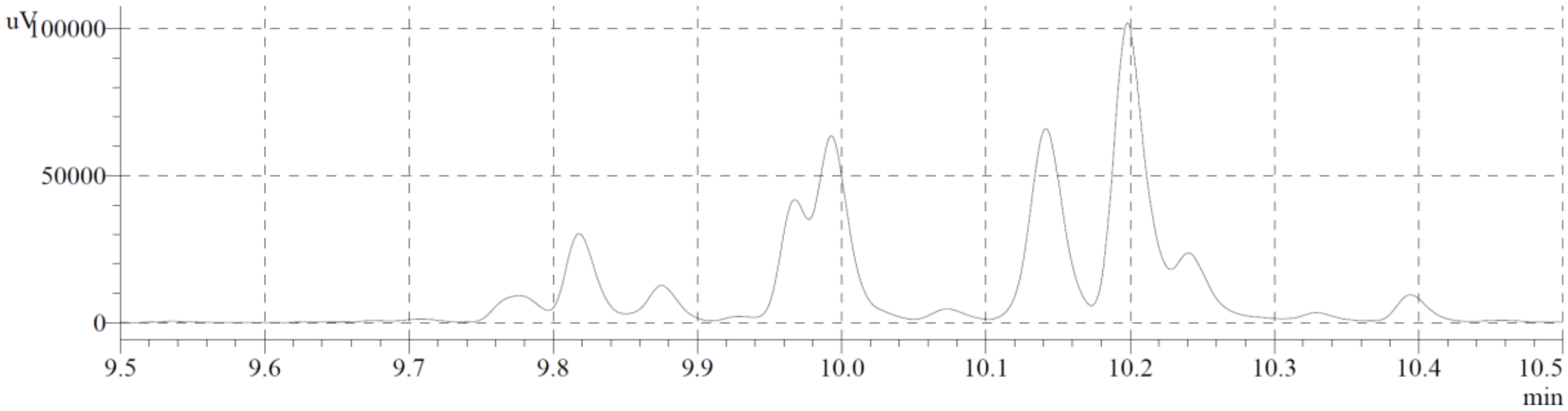

RRF(substrate): 0.833 RRF(ketone): 0.750

i	Retention time, min	Area (A)	Percentage (P), %	Percentage with RRF (P^{RRF}), %	Selectivity, %
-	8.459 (substrate)	333938	22.930	27.5	
-	sum of peaks 9.739-10.304	544424	37.384	49.8	
4	9.993	98479	6.7623	9.0	18.1
	9.015 (biphenyl)	291260	TON = 50; TOF = $5.5\cdot10^{-2}$ s^{-1}		
	Conversion: 72.5		Total product yield: 49.8		

GC analysis of the **5**-catalyzed oxidation of methyl decanoate:

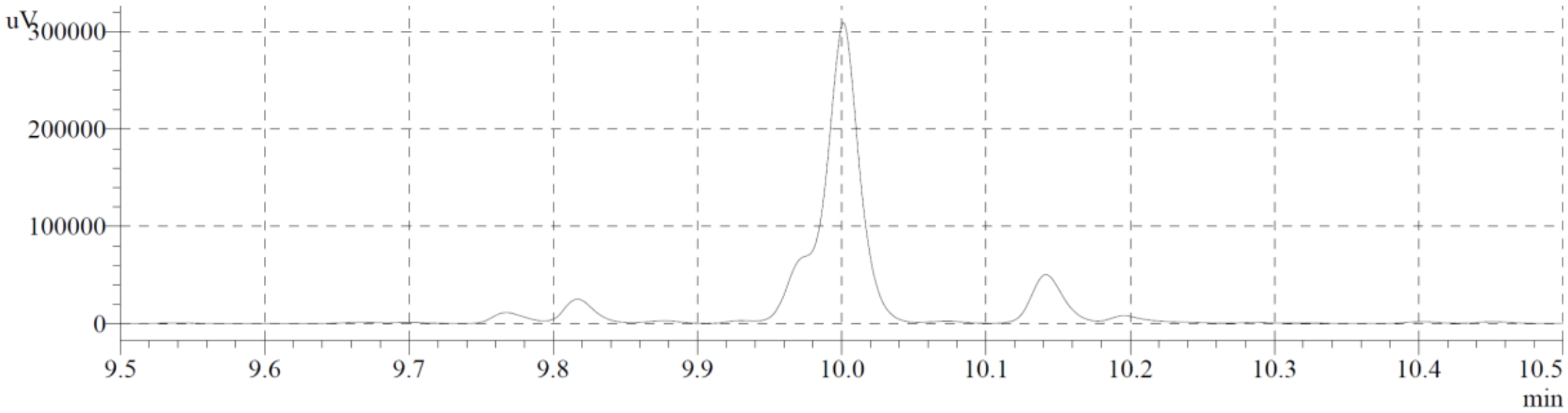

RRF(substrate): 0.833 RRF(ketone): 0.750

i	Retention time, min	Area (A)	Percentage (P), %	Percentage with RRF (P^{RRF}), %	Selectivity, %
-	8.458 (substrate)	158762	11.356	13.6	
-	sum of peaks 9.740-10.304	704924	50.422	67.2	
4	10.001	482030	34.479	46.0	68.4
	9.016 (biphenyl)	279608	TON = 34; TOF = $3.7\cdot10^{-2}$ s^{-1}		
	Conversion: 86.4		Total product yield: 67.2		

The main oxidation product of the **5**-catalyzed oxidation was isolated and characterized following a scaled-up synthesis.

Scaled-up synthesis (×10)
TFE (2.00 mL), substrate **S22** (39.5 µL, 34.5 mg, 185 µmol), 2,2-dimethylpropanoic acid (18.9 mg, 185 µmol) and 200 µL of TfOH solution in TFE (2.5 mol% of TfOH in relation to the substrate) were added to an oven-dried 12 mL vial charged with catalyst **5** (2.79 mg, 1.85 µmol) and a stirring bar. The mixture was cooled to 0 °C. A solution of an 50% aqueous H_2O_2 diluted in TFE (206 µL; stock solution: 100 µL 50 % aqueous H_2O_2 in 1.90 mL TFE) was slowly added over 15 minutes (13.7 µL/min) under stirring via a syringe pump and after that the reaction mixture was stirred for additional 15 minutes. Additional

portion of catalyst **5** (2.79 mg, 1.85 µmol) in TFE (500 µL). The addtion of H_2O_2 diluted in TFE was repeated and after that the reaction mixture was stirred for additional 15 minutes.

Work-up and IBX Oxidation: after the time indicated, H_2O (6.0 mL) was added and the mixture was extracted with HPLC grade EtOAc (3.0 mL × 2), washed with sat. $NaHCO_3$ (2.0 mL), dried over Na_2SO_4, filtered through a silica plug and rinsed with EtOAc. The organic phase was transferred into a one-neck round-bottom flask equipped with a stirring bar. IBX (124 mg, 444 µmol) was added and the mixture was heated to 66 °C overnight. The mixture was allowed to cool down to room temperature, filtered through a silica plug and rinsed with EtOAc. Organic solvents were evaporated to give a crude material.

Isolation of the product: the product was isolated by preparative HPLC using the Reprospher 100 Si (particle size: 5 µm; dimensions: 250 × 25 mm) Dr. Maisch column. The crude was dissolved in 1 mL of hexane, filtered and eluted by hexane/MTBE using isocratic program with 12.5% MTBE (flow rate: 15 mL/min, λ = 220, 256 nm). Obtained fractions were checked by GC, in order to identify pure fractions of C4-oxidized compound **S22**. Pure fractions were combined, organic solvents were evaporated to give the product as colourless oil.

GC analysis of the pure isolated product:

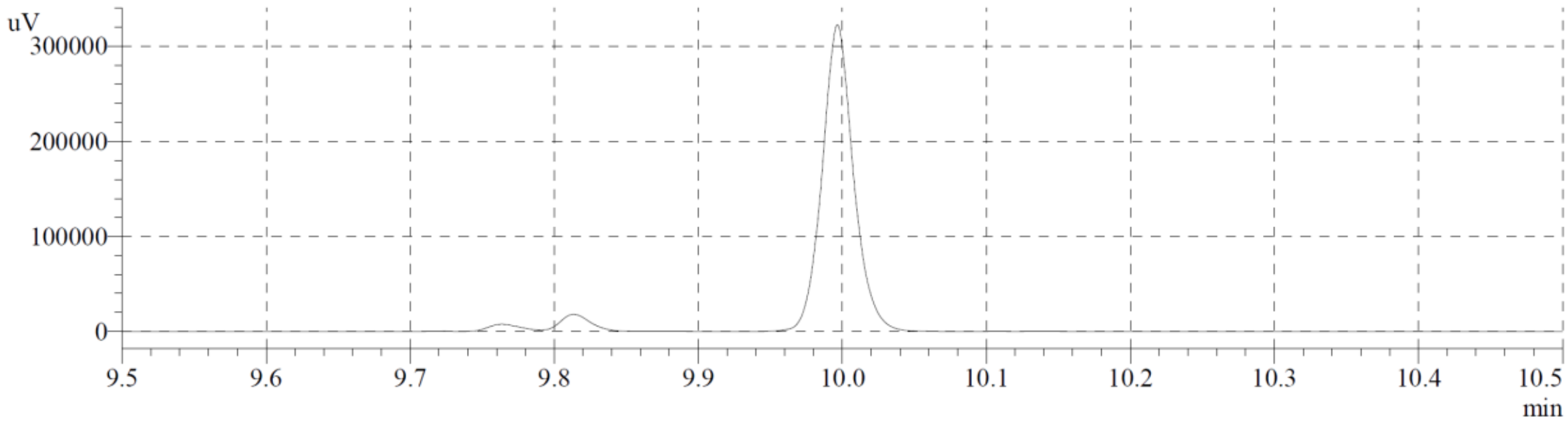

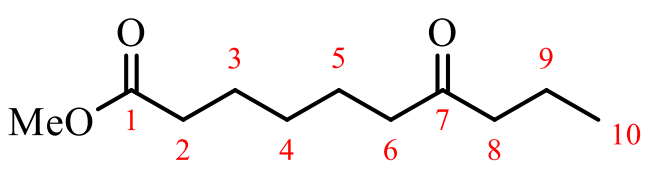

Chemical Formula: $C_{11}H_{20}O_3$
Molecular Weight: 200.28

Yield: 13 mg, 35% (92% GC purity).

IR (ID7, cm^{-1}) 2935 (m), 2873 (w), 1737 (s), 1710 (s), 1457 (s), 1437 (m), 1365 (m), 1251 (w), 1200 (m), 1169 (s), 1097 (w), 1004 (w), 883 (w), 741 (w), 434 (w).

^{1}H NMR (500 MHz, $CDCl_3$) δ 3.66 (s, 3H, OMe), 2.39 (t, ^3J = 7.4 Hz, 2H, C(6)H_2), 2.36 (t, ^3J = 7.4 Hz, 2H, C(8)H_2), 2.30 (t, ^3J = 7.5 Hz, 2H, C(2)H_2), 1.66 – 1.55 (m, 6H, C(3)H_2+C(5)H_2+C(9)H_2), 1.34 – 1.28 (m, 2H, C(4)H_2), 0.91 (t, ^3J = 7.4 Hz, 3H, Me(10)).

^{13}C NMR (125 MHz, $CDCl_3$) δ 211.1 (C(7)=O), 174.1 (C(1)=O), 51.5 (OMe), 44.7 (C(8)H_2), 42.4 (C(6)H_2), 33.8 (C(2)H_2), 28.7 (C(4)H_2), 24.7 (C(3)H_2), 23.3 (C(5)H_2), 17.3 (C(9)H_2), 13.8 (Me(10)).

HRMS (ESI$^+$) *m/z* calcd for $C_{11}H_{21}O_3$ $[M + H]^+$: 201.1485; found 201.1484.

5. Computational part

To elucidate the experimentally observed site-selectivity of octane oxidation, a computational analysis was performed using the recently developed g-xTB method. This semiempirical quantum mechanical approach provides a computationally efficient approximation to ωB97M-V/def2-TZVPPD-level properties and is particularly suited for the investigation of large supramolecular host–guest assemblies.[23]

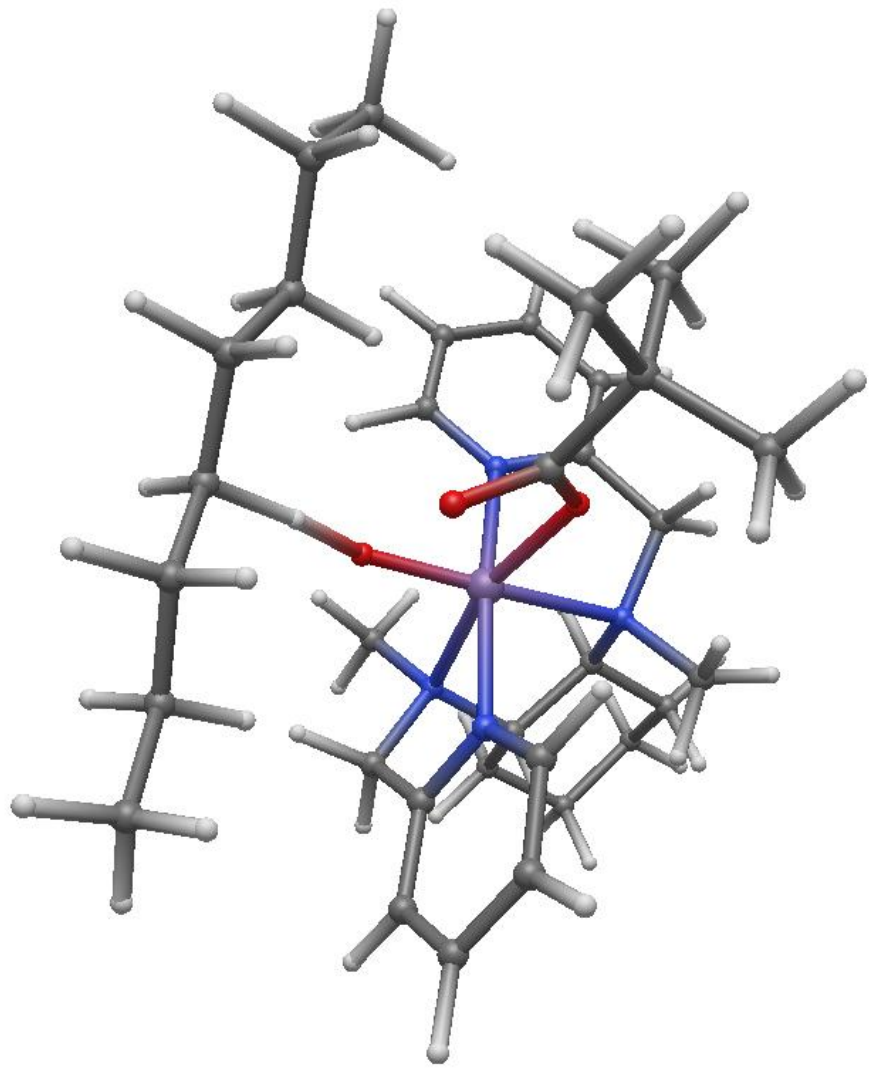

Figure 5. g-xTB-optimized transition state for C4–H abstraction of n-octane by the Mn(V)-oxo species in a reduced model of the dicationic triplet manganese complex.

Initially, a reduced model of the dicationic manganese complex in its triplet electronic state was employed to locate a transition state corresponding to hydrogen atom abstraction from the C4-position of octane by the reactive Mn=O unit (Figure 5). The transition state was optimized using the g-xTB method as implemented in ORCA 6.1.1.[24] The resulting structure exhibits a single imaginary frequency of -507 cm^{-1} corresponding to the C(4)–H···O–Mn hydrogen-transfer mode. The associated geometrical parameters, C(4)–H = 1.23 Å and H···O(Mn) = 1.34 Å, are characteristic of a late-stage hydrogen abstraction process.

To investigate the influence of the supramolecular host environment on the regioselectivity of C–H activation, the complete manganese host–guest complex containing octane was subjected to conformational optimization using the Global Optimizer Algorithm (GOAT)[25] implemented in ORCA. Starting from transition-state-like geometries, only the C–H (1.23 Å) and H···O(Mn) (1.34 Å) distances identified in the localized transition state were constrained, while all remaining degrees of freedom were optimized. Independent global optimization procedures were performed for both C(4)-methylene C–H bonds of octane. In all cases, the resulting global minima feature the alkane substrate deeply embedded within the calix[4]arene cavity, positioning the reactive methylene groups in close proximity to the manganese(V)-oxo center.

The octane conformations obtained from the GOAT searches were subsequently subjected to unconstrained geometry optimization using the g-xTB method. The resulting dicationic triplet host–guest complex (Figure 6) was characterized as a true minimum on the potential energy surface by frequency analysis, which revealed the absence of imaginary frequencies.

To assess the dynamic behavior of the encapsulated substrate and to establish a structural rationale for the experimentally observed regioselectivities, molecular dynamics simulations were performed on the fully optimized host–guest complex. The simulations were carried out with the g-xTB method

implemented in xTB version 6.7.1[26] at 298 K employing a time step of 2 fs and a hydrogen mass of 4 atomic mass units. To prevent dissociation of the host–guest assembly during the simulation, a soft distance constraint (force constant 5.0 kcal/mol/Å^2, corresponding to 0.00223 a.u.) was applied between one terminal methyl carbon atom of octane and an aryl carbon atom of the calix[4]arene host.

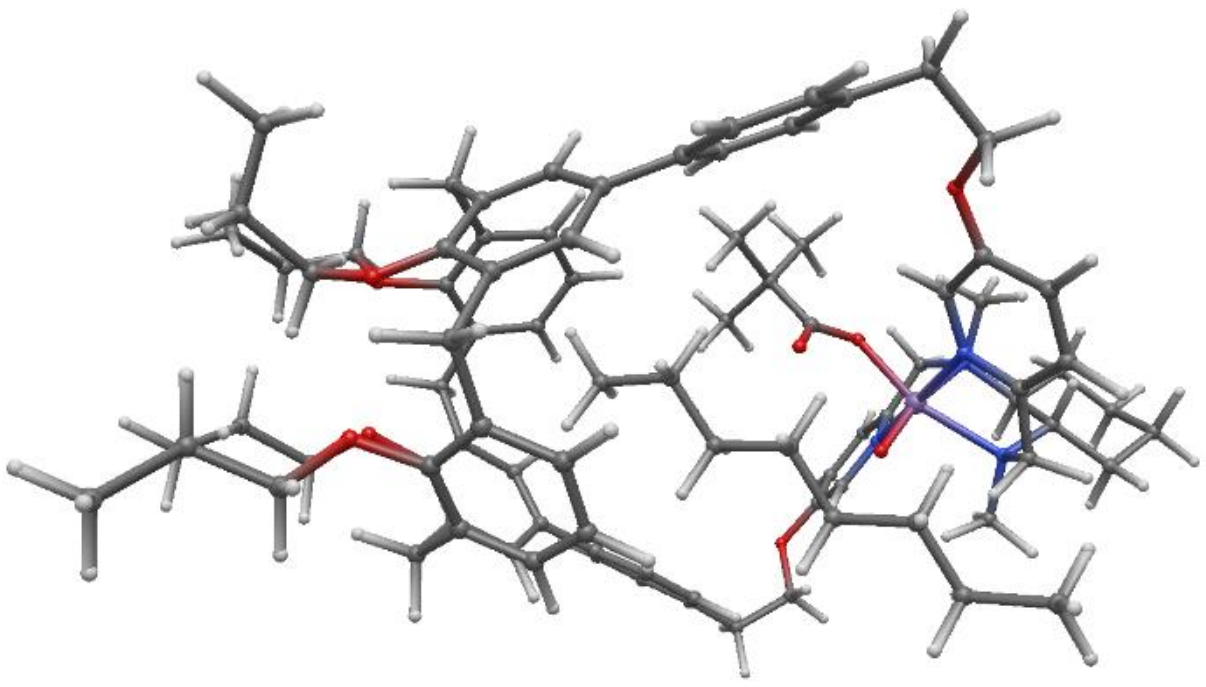

Figure 6. Most stable host–guest complex for octane, obtained by global conformational optimization using the GOAT algorithm implemented in ORCA 6.1.1 at the g-xTB level.

Analysis of geometrical snapshots collected over a 16 ps trajectory provided average distances between the reactive Mn=O unit and the methylene groups of the encapsulated octane substrate. The shortest average distances were observed for the central C4 and C5 methylene positions (3.80 and 3.72 Å, respectively), whereas substantially larger distances were found for the C3/C6 (5.19 and 4.51 Å) and C2/C7 (5.75 and 5.13 Å) positions. These results indicate a pronounced preference for interaction of the manganese(V)-oxo species with the central methylene groups of the substrate.

The dynamic proximity of the Mn=O unit to the C4/C5 positions throughout the MD trajectory provides a structural explanation for the experimentally observed regioselectivity of C–H activation. The significantly shorter average Mn=O···C(4/5)H_2 distances at these sites suggest a higher probability of productive hydrogen abstraction and consequently a greater propensity for oxidation at the central methylene positions of octane.

6. References

(1) Knezevic, M.; Heilmann, M.; Piccini, G. M.; Tiefenbacher, K. Overriding Intrinsic Reactivity in Aliphatic C−H Oxidation: Preferential C3/C4 Oxidation of Aliphatic Ammonium Substrates. *Angew Chem Int Ed* **2020**, *59* (30), 12387–12391. https://doi.org/10.1002/anie.202004242.

(2) Scanlon, J. T.; Willis, D. E. Calculation of Flame Ionization Detector Relative Response Factors Using the Effective Carbon Number Concept. *Journal of Chromatographic Science* **1985**, *23* (8), 333–340. https://doi.org/10.1093/chromsci/23.8.333.

(3) Lu, Y.; Knezevic, M.; Prescimone, A.; Goldfuss, B.; Tiefenbacher, K. Site-Selective C(Sp3)–H Oxidation of Alkyl Substrates Devoid of Functional Handles. *Chem* **2025**, *11* (3), 102442. https://doi.org/10.1016/j.chempr.2025.102442.

(4) Lu, Y.; Anisovich, K.; Tiefenbacher, K. Convergent Synthesis of Tetradentate Aminopyridine C–H Oxidation Catalysts. *ACS Org. Inorg. Au* **2026**, *6* (2), 202–208. https://doi.org/10.1021/acsorginorgau.6c00006.

(5) Gutsche, C. D.; Levine, J. A. Calixarenes. 6. Synthesis of a Functionalizable Calix[4]Arene in a Conformationally Rigid Cone Conformation. *J. Am. Chem. Soc.* **1982**, *104* (9), 2652–2653. https://doi.org/10.1021/ja00373a060.

(6) Dondoni, A.; Marra, A.; Scherrmann, M.; Casnati, A.; Sansone, F.; Ungaro, R. Synthesis and Properties of *O* -Glycosyl Calix[4]Arenes (Calixsugars). *Chemistry A European J* **1997**, *3* (11), 1774–1782. https://doi.org/10.1002/chem.19970031108.

(7) Giovanardi, G.; Cattani, S.; Balestri, D.; Secchi, A.; Cera, G. Iridium-Catalyzed C–H Borylations: Regioselective Functionalizations of Calix[4]Arene Macrocycles. *J. Org. Chem.* **2024**, *89* (12), 8486–8499. https://doi.org/10.1021/acs.joc.4c00419.

(8) Ji, F.; Li, J.; Li, X.; Guo, W.; Wu, W.; Jiang, H. Carbonylation Access to Phthalimides Using Self-Sufficient Directing Group and Nucleophile. *J. Org. Chem.* **2018**, *83* (1), 104–112. https://doi.org/10.1021/acs.joc.7b02433.

(9) Tan, Y.; Wang, S. J.; Wang, Y. T.; Gao, B. X.; Ba, X. W. A Highly Efficient Synthesis of Sulphonamide Derivatives in the Presence of N-Methyl-2-Pyrrolidone. *Synthetic Communications* **2010**, *40* (24), 3648–3653. https://doi.org/10.1080/00397910903470517.

(10) Zeng, R.; Sheng, H.; Zhang, Y.; Feng, Y.; Chen, Z.; Wang, J.; Chen, M.; Zhu, M.; Guo, Q. Heterobimetallic Dinuclear Lanthanide Alkoxide Complexes as Acid–Base Difunctional Catalysts for Transesterification. *J. Org. Chem.* **2014**, *79* (19), 9246–9252. https://doi.org/10.1021/jo5016536.

(11) Matin, M. M.; Bhattacharjee, S. C.; Chakraborty, P.; Alam, M. S. Synthesis, PASS Predication, in Vitro Antimicrobial Evaluation and Pharmacokinetic Study of Novel n-Octyl Glucopyranoside Esters. *Carbohydrate Research* **2019**, *485*, 107812. https://doi.org/10.1016/j.carres.2019.107812.

(12) Cianfanelli, M.; Olivo, G.; Milan, M.; Klein Gebbink, R. J. M.; Ribas, X.; Bietti, M.; Costas, M. Enantioselective C–H Lactonization of Unactivated Methylenes Directed by Carboxylic Acids. *J. Am. Chem. Soc.* **2020**, *142* (3), 1584–1593. https://doi.org/10.1021/jacs.9b12239.

(13) Serrano-Plana, J.; Acuña-Parés, F.; Dantignana, V.; Oloo, W. N.; Castillo, E.; Draksharapu, A.; Whiteoak, C. J.; Martin-Diaconescu, V.; Basallote, M. G.; Luis, J. M.; Que, L.; Costas, M.; Company, A. Acid-Triggered O−O Bond Heterolysis of a Nonheme Fe^{III} (OOH) Species for the Stereospecific Hydroxylation of Strong C−H Bonds. *Chemistry A European J* **2018**, *24* (20), 5331–5340. https://doi.org/10.1002/chem.201704851.

(14) Miao, C.; Wang, B.; Wang, Y.; Xia, C.; Lee, Y.-M.; Nam, W.; Sun, W. Proton-Promoted and Anion-Enhanced Epoxidation of Olefins by Hydrogen Peroxide in the Presence of Nonheme Manganese Catalysts. *J. Am. Chem. Soc.* **2016**, *138* (3), 936–943. https://doi.org/10.1021/jacs.5b11579.

(15) Dantignana, V.; Milan, M.; Cussó, O.; Company, A.; Bietti, M.; Costas, M. Chemoselective Aliphatic C–H Bond Oxidation Enabled by Polarity Reversal. *ACS Cent. Sci.* **2017**, *3* (12), 1350–1358. https://doi.org/10.1021/acscentsci.7b00532.

(16) Smithrud, D. B.; Diederich, F. Strength of Molecular Complexation of Apolar Solutes in Water and in Organic Solvents Is Predictable by Linear Free Energy Relationships: A General Model for Solvation Effects on Apolar Binding. *J. Am. Chem. Soc.* **1990**, *112* (1), 339–343. https://doi.org/10.1021/ja00157a052.

(17) Reichardt, C. Solvatochromic Dyes as Solvent Polarity Indicators. *Chem. Rev.* **1994**, *94* (8), 2319–2358. https://doi.org/10.1021/cr00032a005.
(18) Maruoka, K.; Concepcion, A. B.; Yamamoto, H. Selective Homologation of Ketones and Aldehydes with Diazoalkanes Promoted by Organoaluminum Reagents. *Synthesis* **1994**, *1994* (12), 1283–1290. https://doi.org/10.1055/s-1994-25682.
(19) Mubarak, M. S.; Pagel, M.; Marcus, L. M.; Peters, D. G. Formation of 2-(3‘-Oxocyclohexyl)-2-Cyclohexen-1-One via Reduction of 2-Cyclohexen-1-One with Electrogenerated Nickel(I) Salen. *J. Org. Chem.* **1998**, *63* (4), 1319–1322. https://doi.org/10.1021/jo971538z.
(20) Aureliano Antunes, C. S.; Bietti, M.; Lanzalunga, O.; Salamone, M. Photolysis of 1-Alkylcycloalkanols in the Presence of (Diacetoxyiodo)Benzene and I_2 . Intramolecular Selectivity in the β-Scission Reactions of the Intermediate 1-Alkylcycloalkoxyl Radicals. *J. Org. Chem.* **2004**, *69* (16), 5281–5289. https://doi.org/10.1021/jo049524y.
(21) Jiang, C.; Wu, Y.; Zhang, Y.; Zong, J.; Wang, N.; Liu, G.; Liu, R.; Yu, H. Supramolecular Modulation for Selective Mechanochemical Iron-Catalyzed Olefin Oxidation. *Angew Chem Int Ed* **2025**, *64* (1), e202413901. https://doi.org/10.1002/anie.202413901.
(22) Yu, Q.; Zhao, W.; Cui, Y.; Zhang, C.; Zhang, X.; Nie, L.; Li, Z. Direct Production of Cyclohexanones from Lignin via Chlorine-Mediated Catalytic Hydroprocessing. *Angew Chem Int Ed* **2025**, *64* (39), e202510322. https://doi.org/10.1002/anie.202510322.
(23) Froitzheim, T.; Müller, M.; Hansen, A.; Grimme, S. G-xTB: A General-Purpose Extended Tight-Binding Electronic Structure Method For the Elements H to Lr (Z=1–103). June 23, 2025. https://doi.org/10.26434/chemrxiv-2025-bjxvt.
(24) Neese, F. Software Update: The ORCA Program System—Version 6.0. *WIREs Comput Mol Sci* **2025**, *15* (2), e70019. https://doi.org/10.1002/wcms.70019.
(25) De Souza, B. GOAT: A Global Optimization Algorithm for Molecules and Atomic Clusters. *Angew Chem Int Ed* **2025**, *64* (18), e202500393. https://doi.org/10.1002/anie.202500393.
(26) Bannwarth, C.; Caldeweyher, E.; Ehlert, S.; Hansen, A.; Pracht, P.; Seibert, J.; Spicher, S.; Grimme, S. Extended TIGHT-BINDING Quantum Chemistry Methods. *WIREs Comput Mol Sci* **2021**, *11* (2), e1493. https://doi.org/10.1002/wcms.1493.

Appendix A: NMR spectra of compounds

^{1}H NMR (500 MHz, $CDCl_3$, 298K) of compound **SI-1**

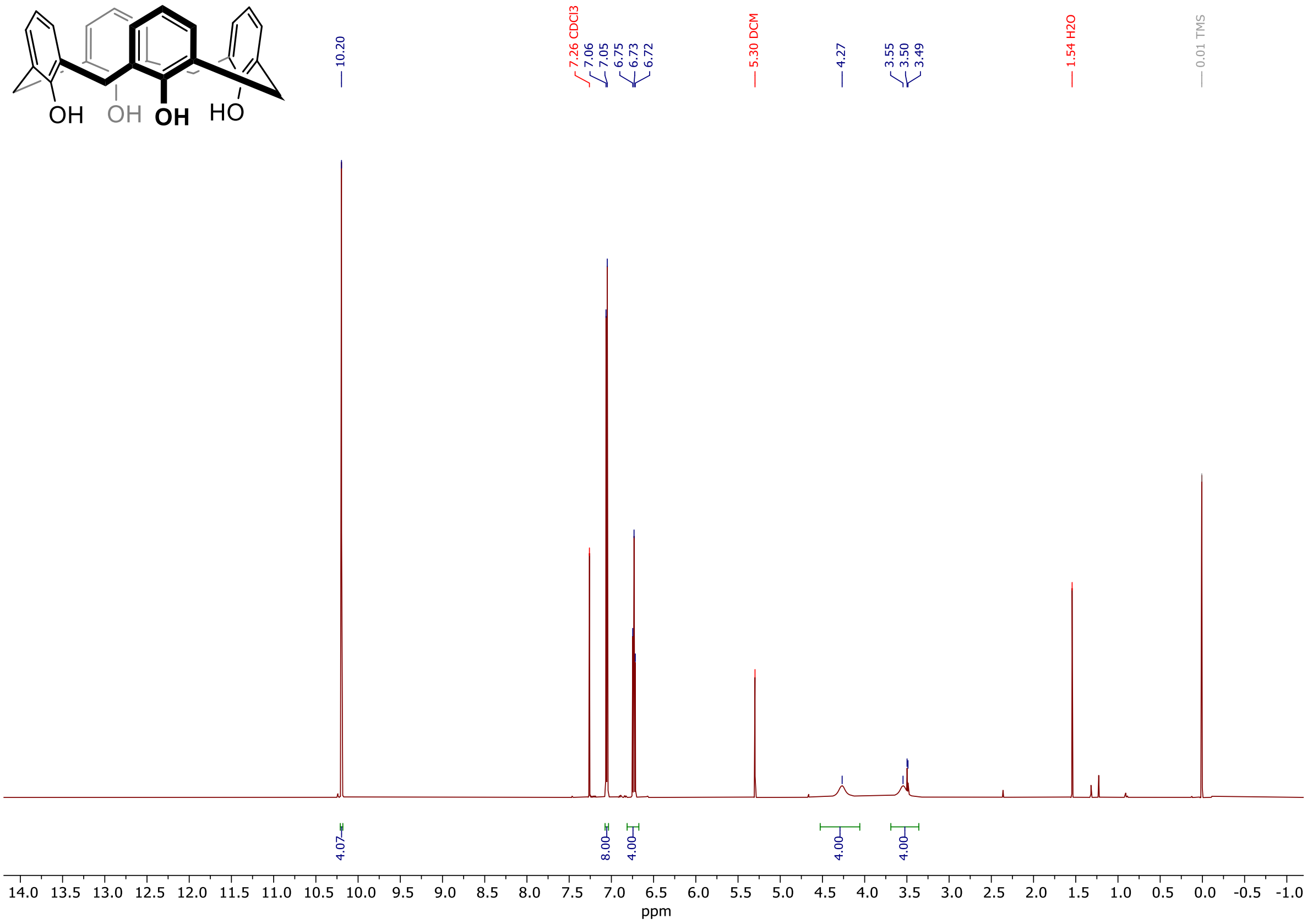

^{13}C NMR (125 MHz, $CDCl_3$, 298K) of compound **SI-1**

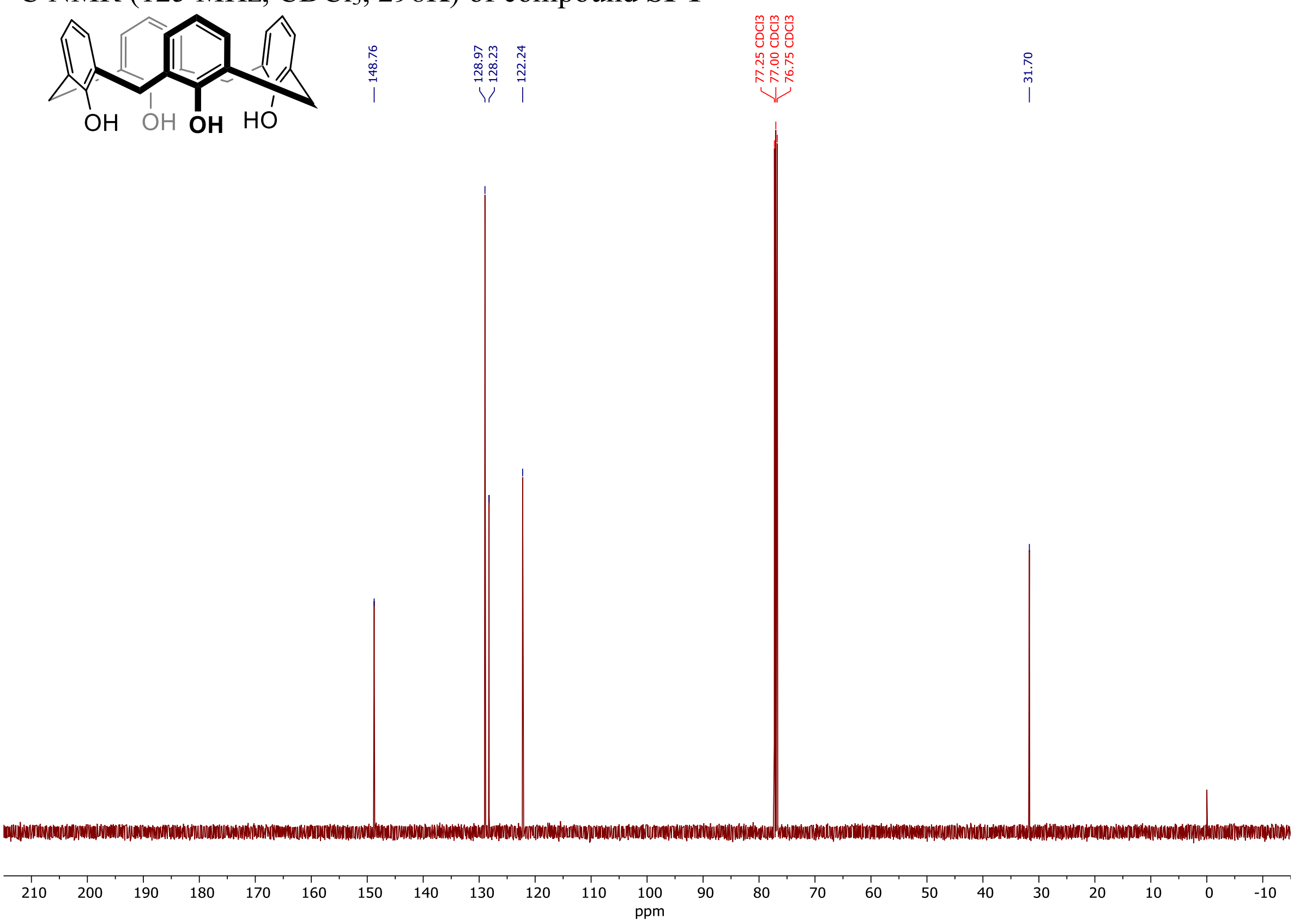

^{1}H NMR (500 MHz, $CDCl_3$, 298K) of compound **6**

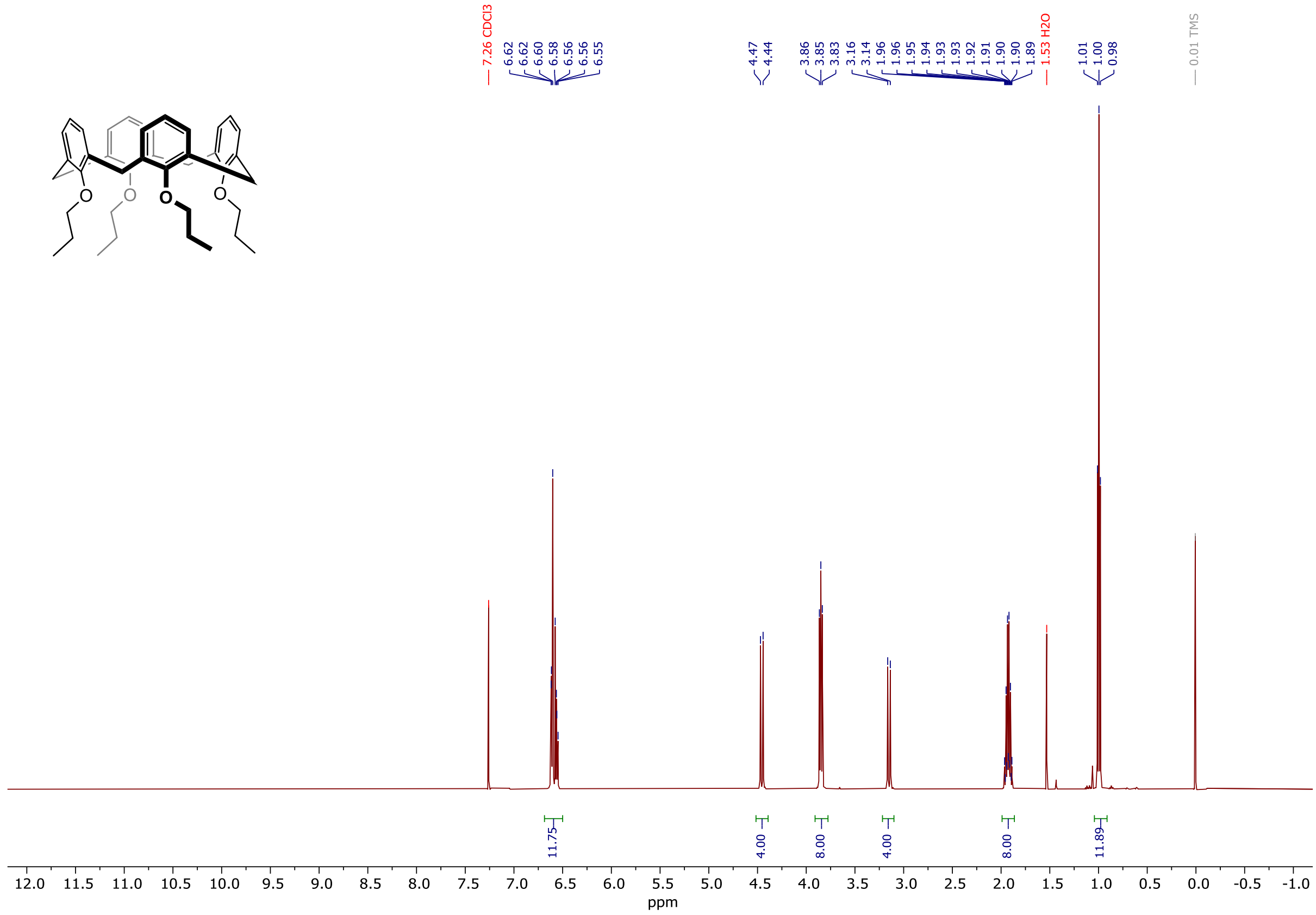

^{13}C NMR (125 MHz, $CDCl_3$, 298K) of compound **6**

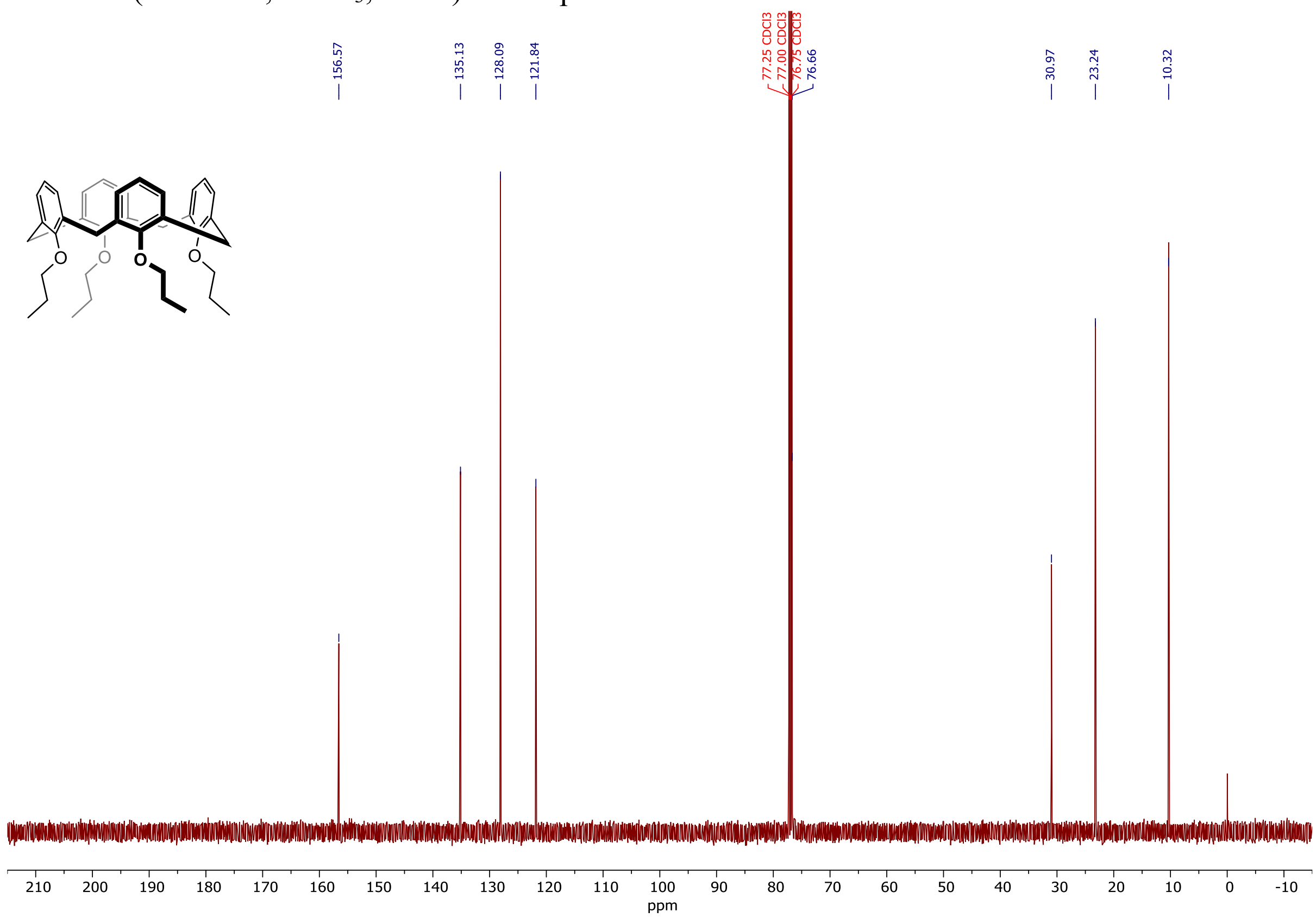

^{1}H NMR (500 MHz, $CDCl_3$, 298K) of compound **7**

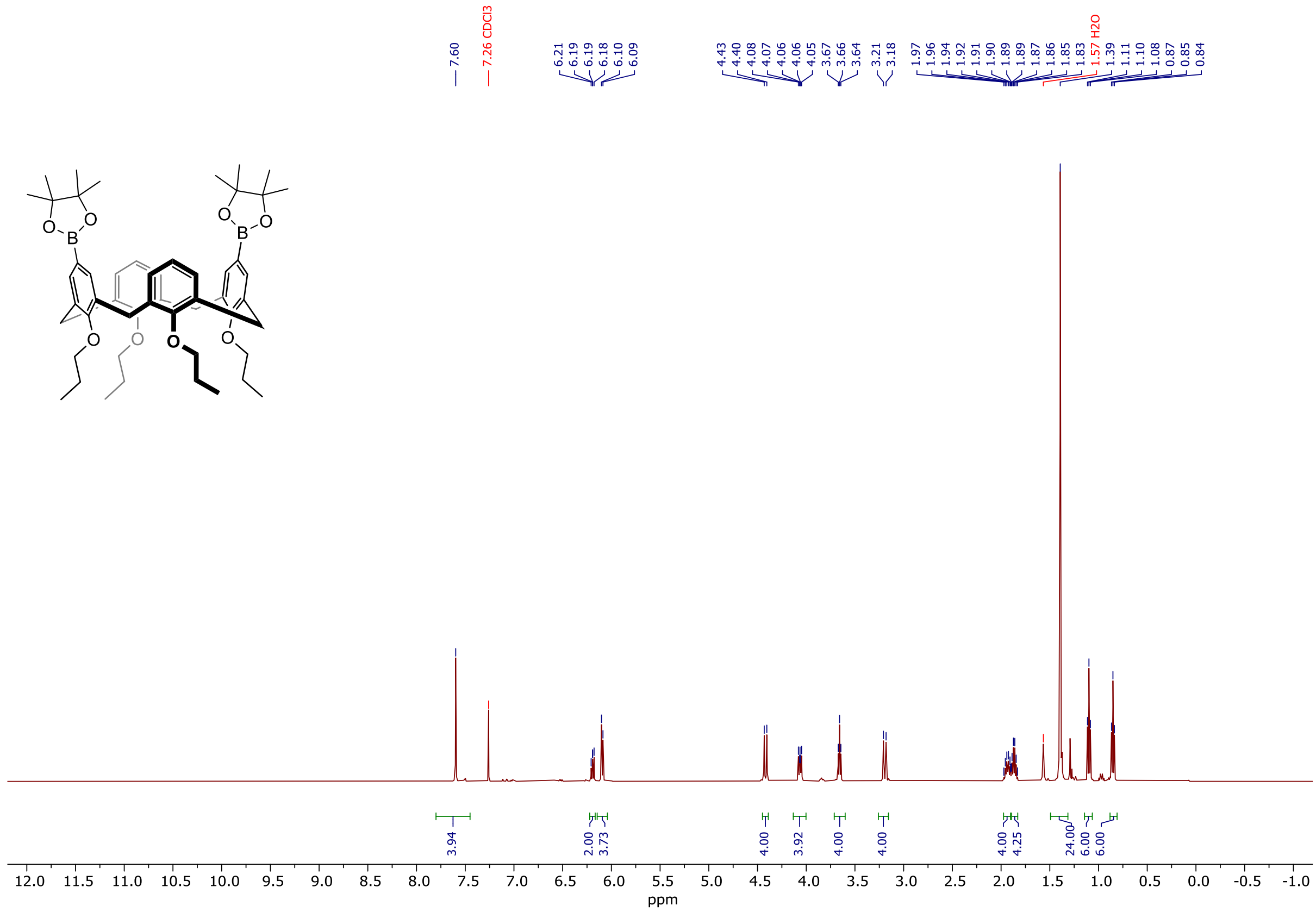

^{13}C NMR (125 MHz, $CDCl_3$, 298K) of compound **7**

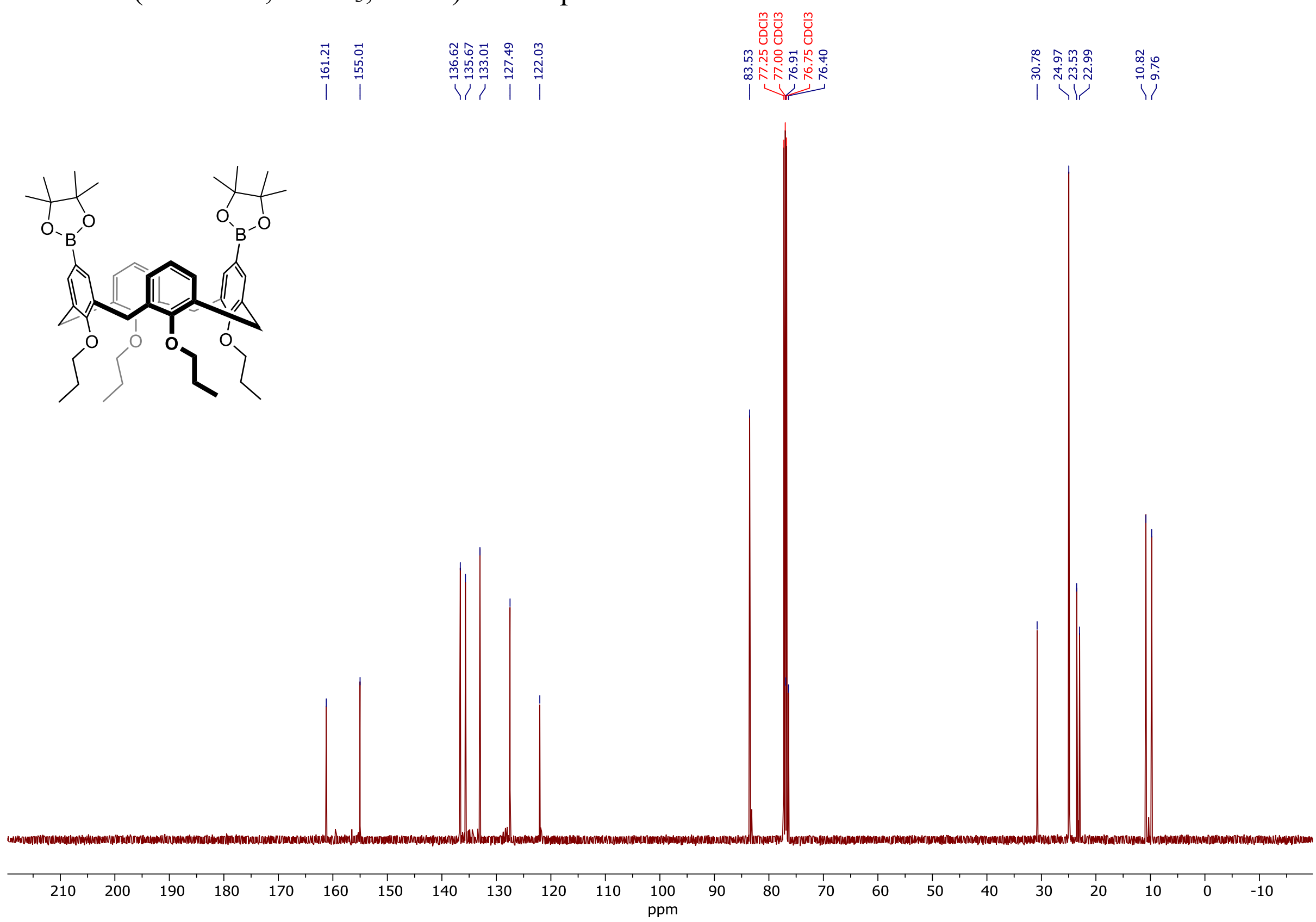

^{1}H NMR (500 MHz, $CDCl_3$, 298K) of compound **10**

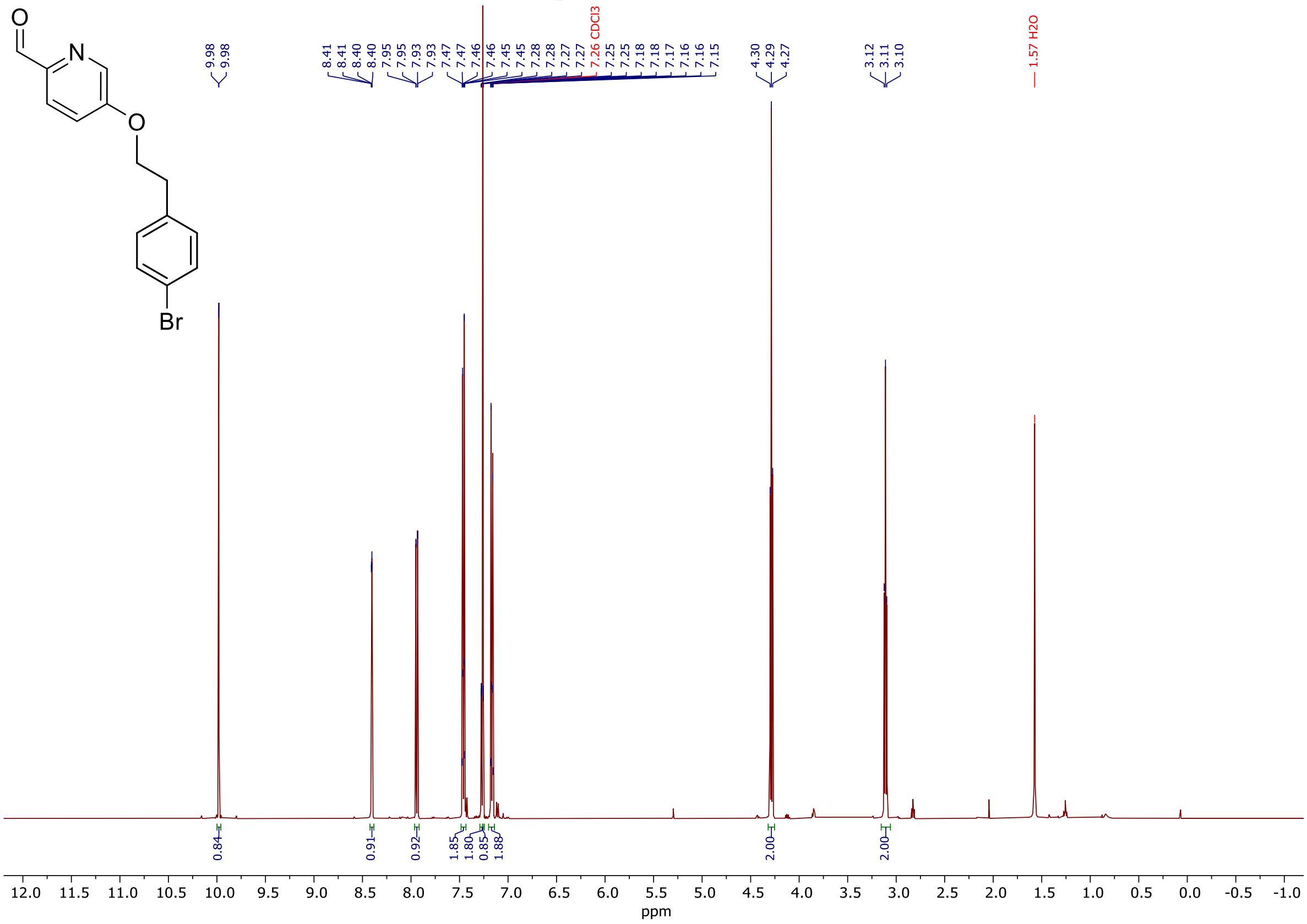

^{13}C NMR (150 MHz, $CDCl_3$, 298K) of compound **10**

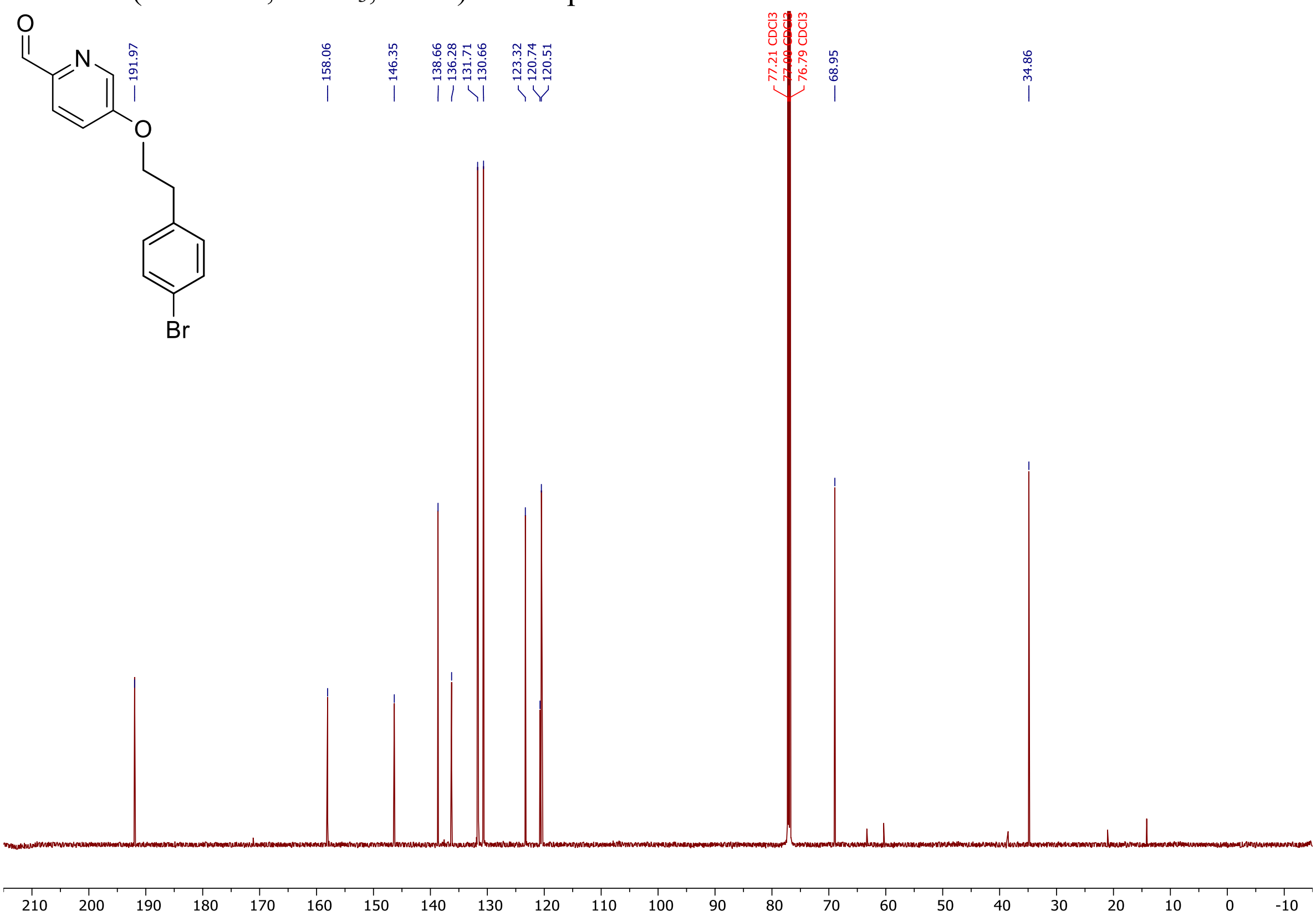

^{1}H NMR (500 MHz, $CDCl_3$, 298K) of compound **SI-2**

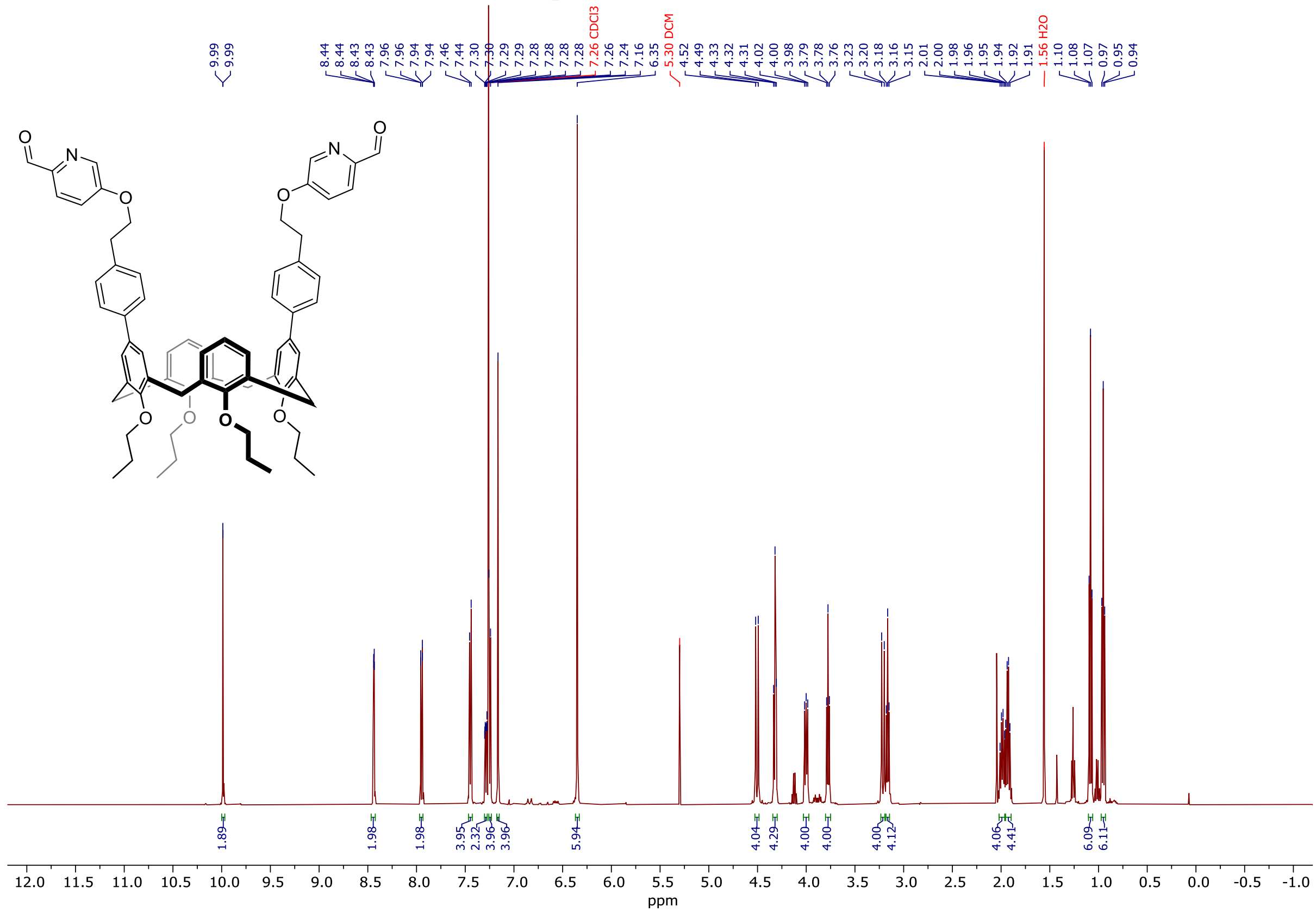

^{13}C NMR (150 MHz, $CDCl_3$, 298K) of compound **SI-2**

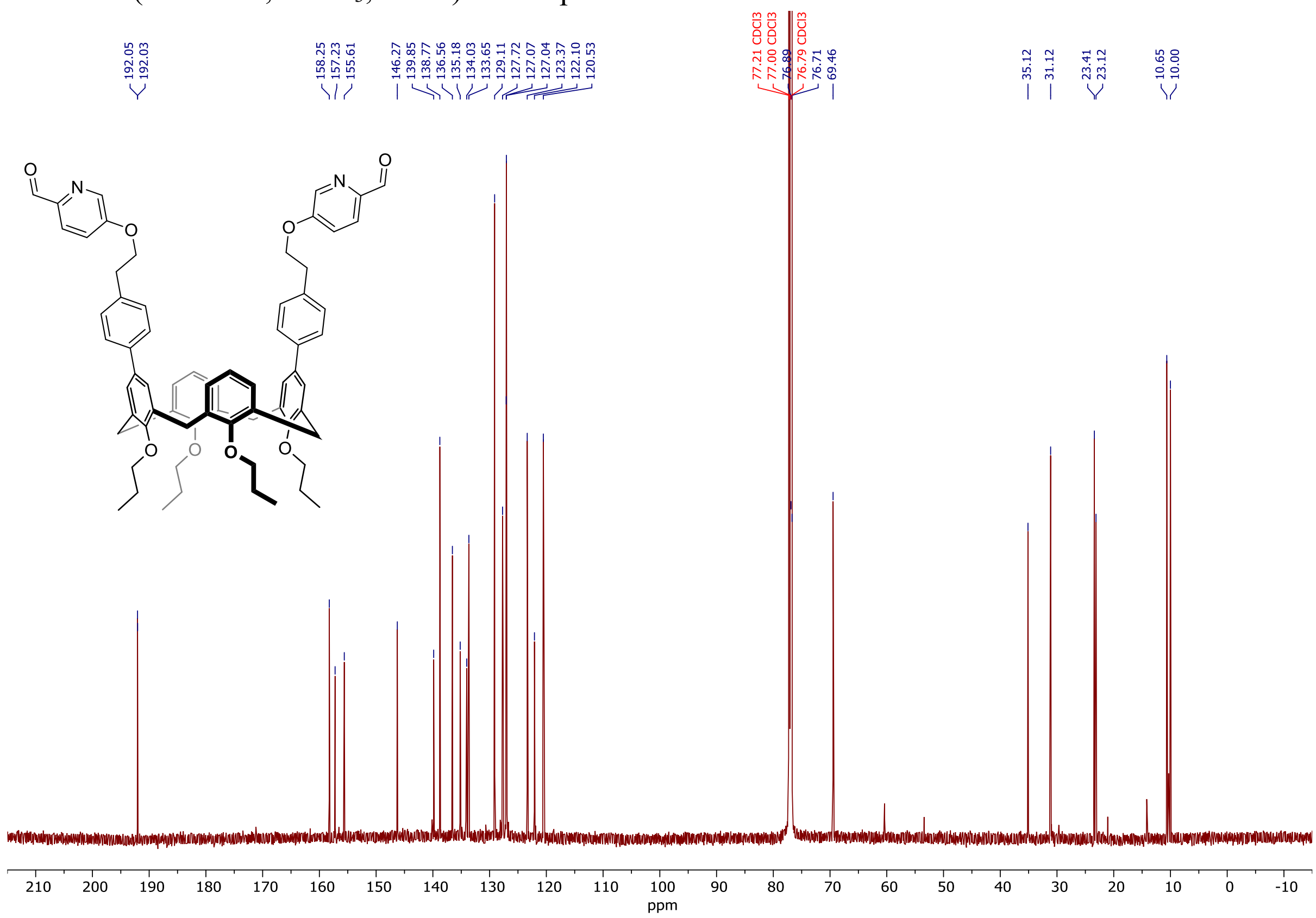

^{1}H NMR (500 MHz, $CDCl_3$, 298K) of compound **11**

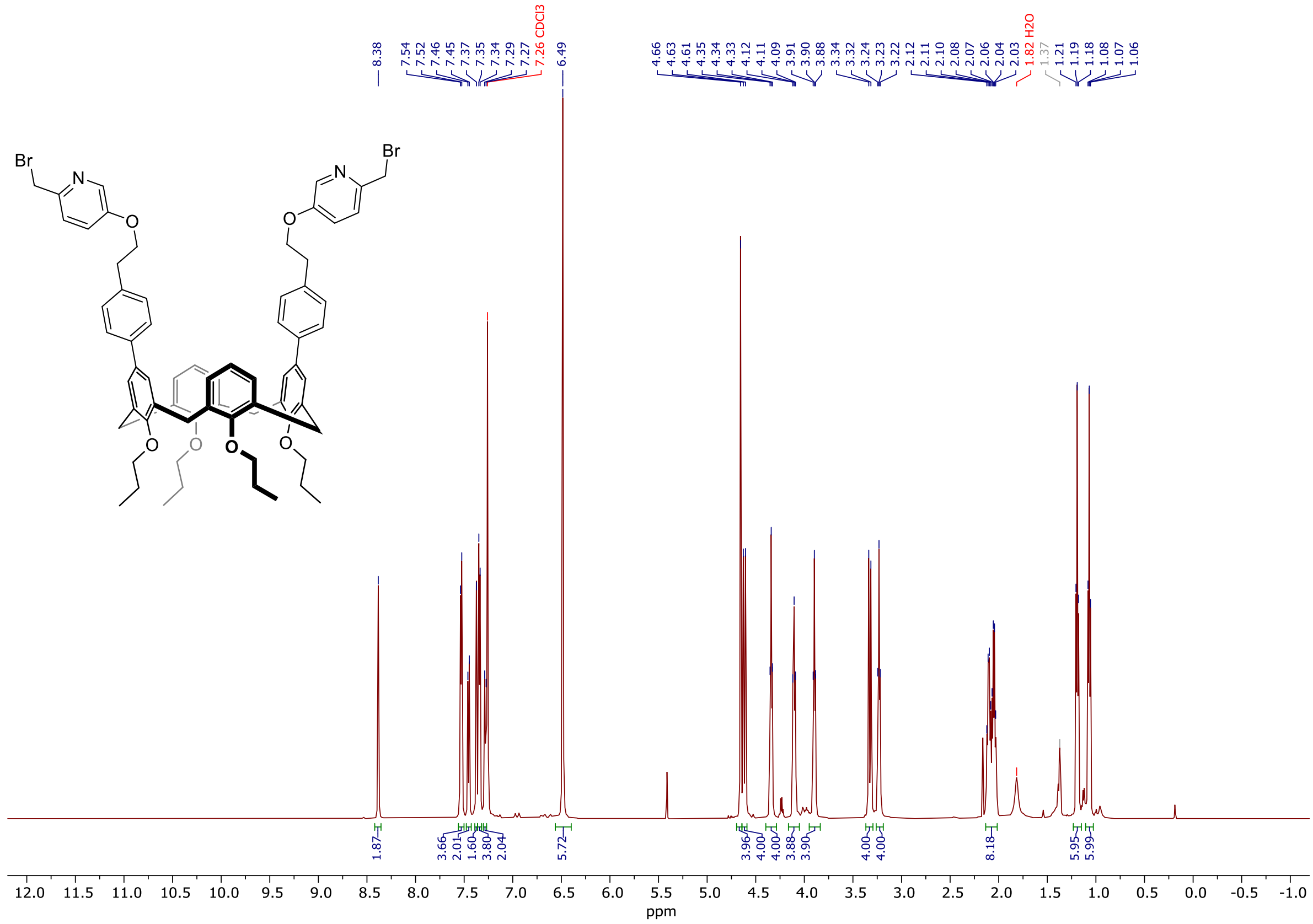

^{13}C NMR (150 MHz, $CDCl_3$, 298K) of compound **11**

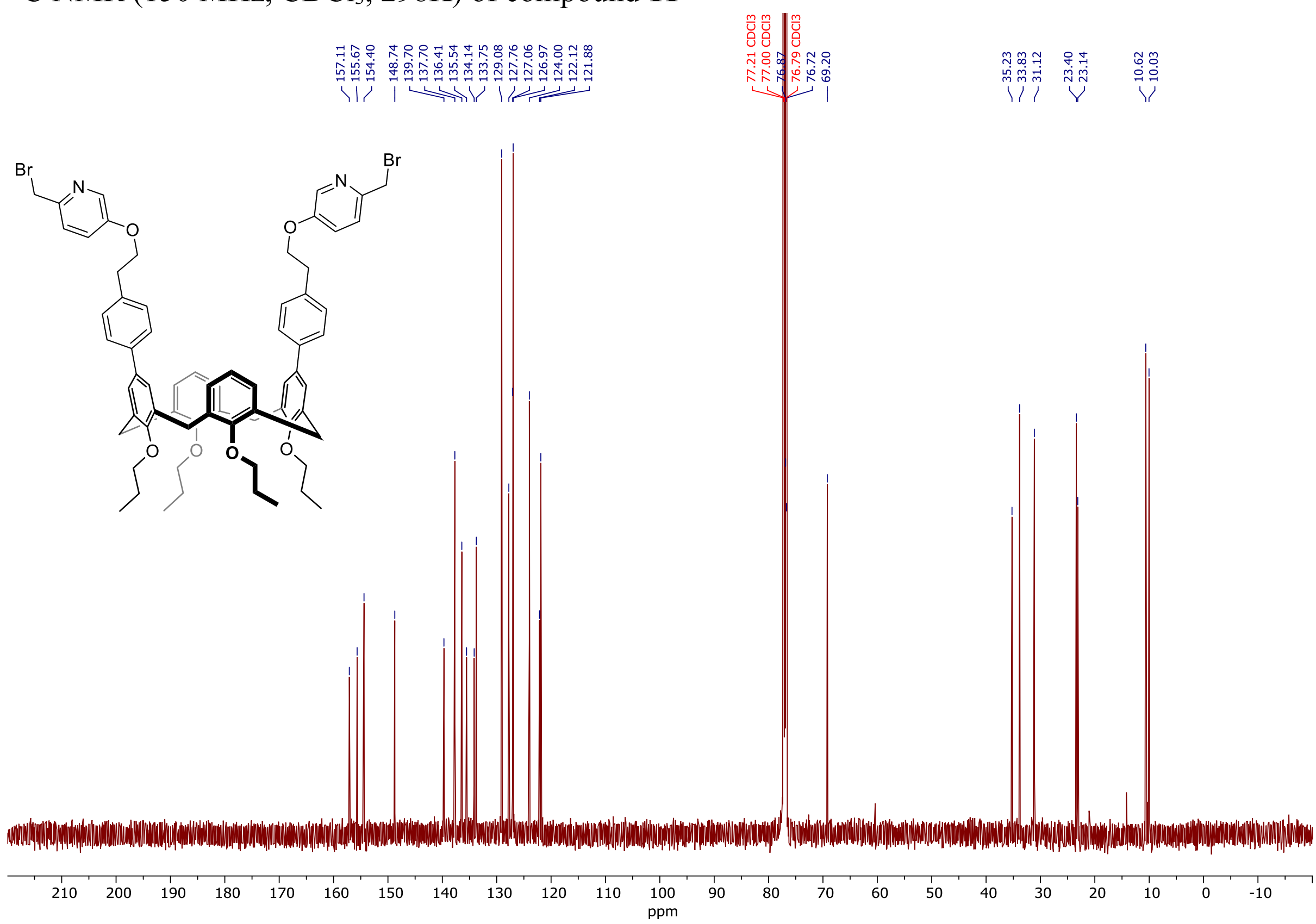

^{1}H NMR (600 MHz, $CDCl_3$, 298K) of compound **12**

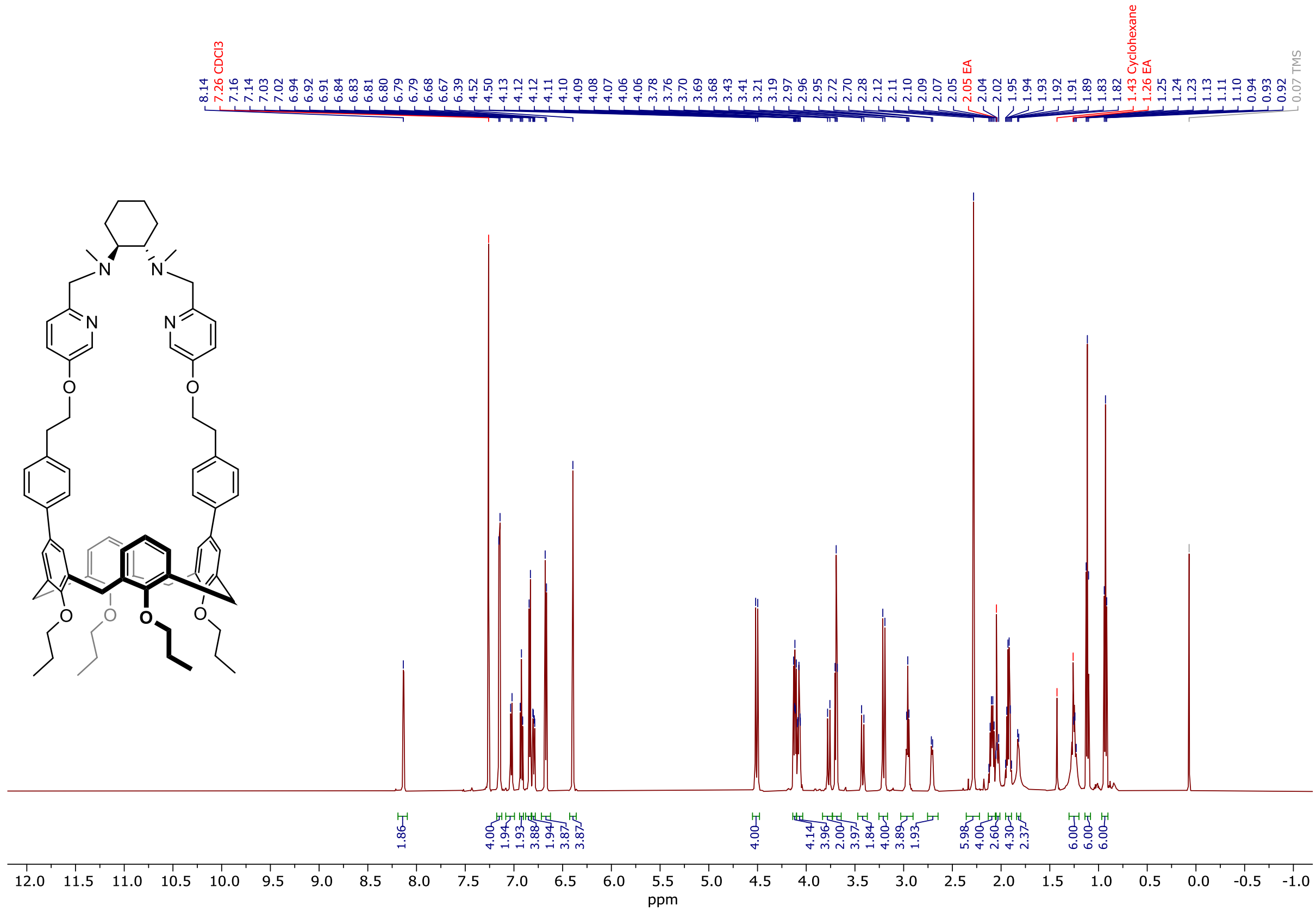

^{13}C NMR (150 MHz, $CDCl_3$, 298K) of compound **12**

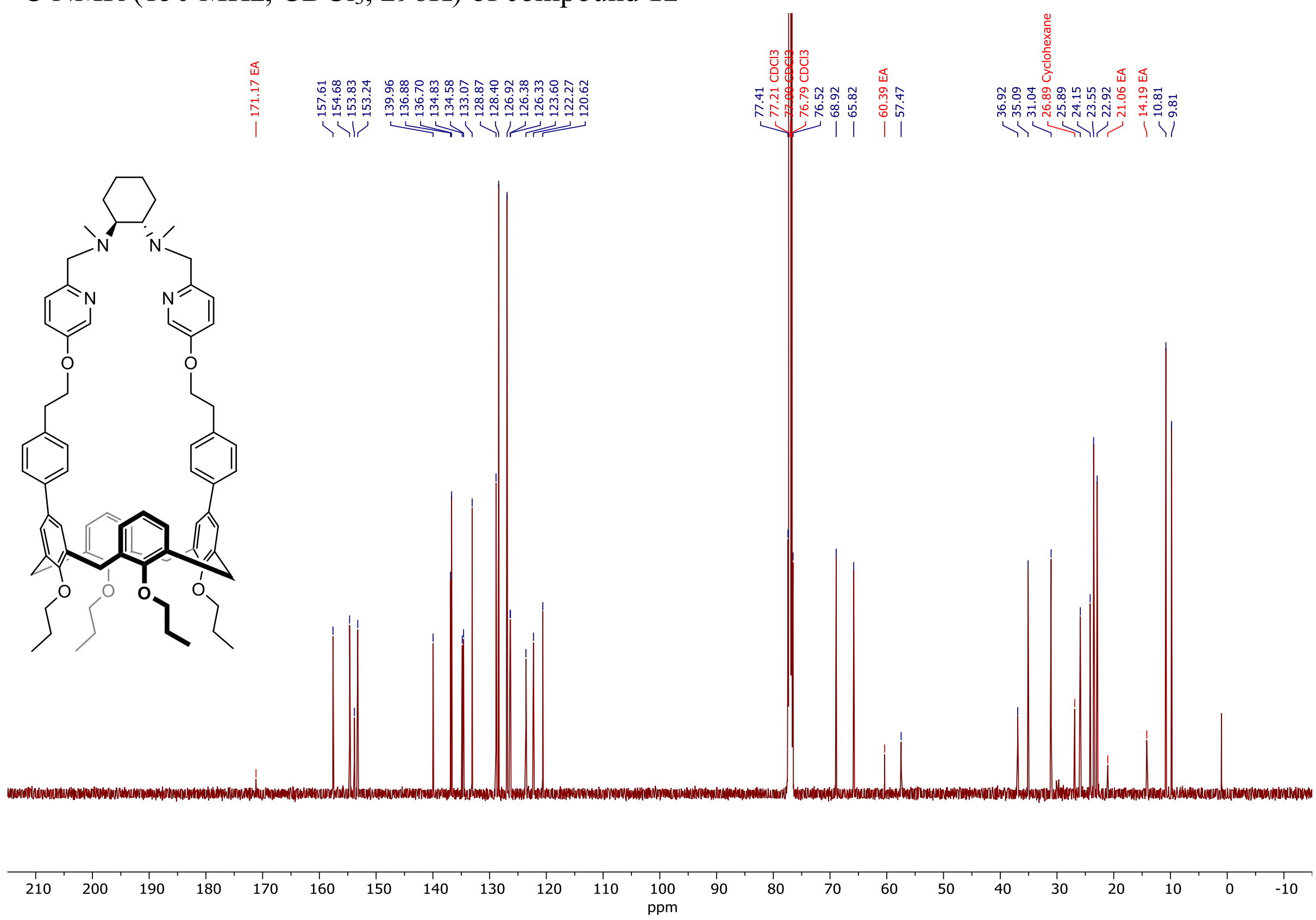

^{1}H NMR (500 MHz, $CDCl_3$, 298K) of compound **S10**

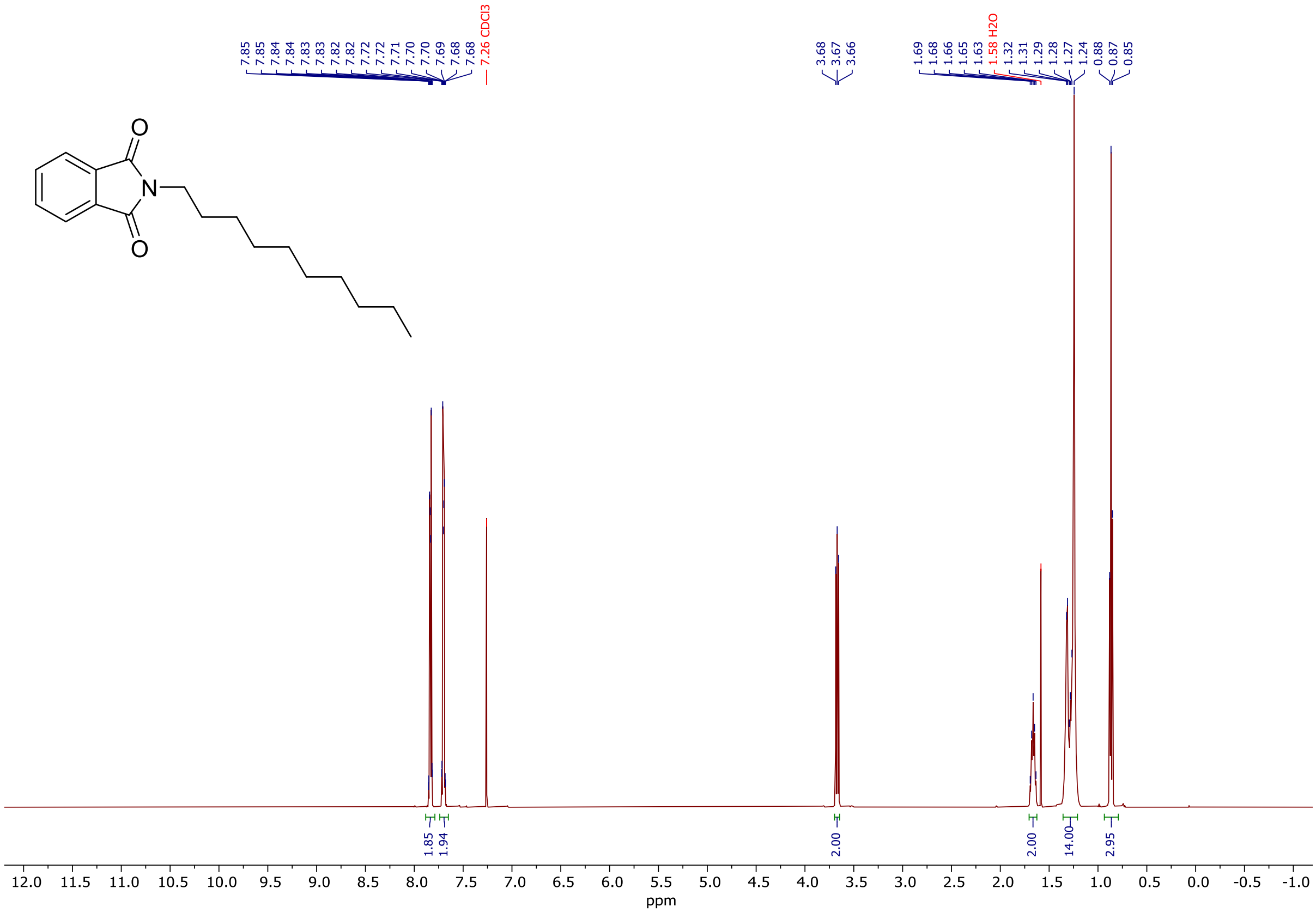

^{13}C NMR (125 MHz, $CDCl_3$, 298K) of compound **S10**

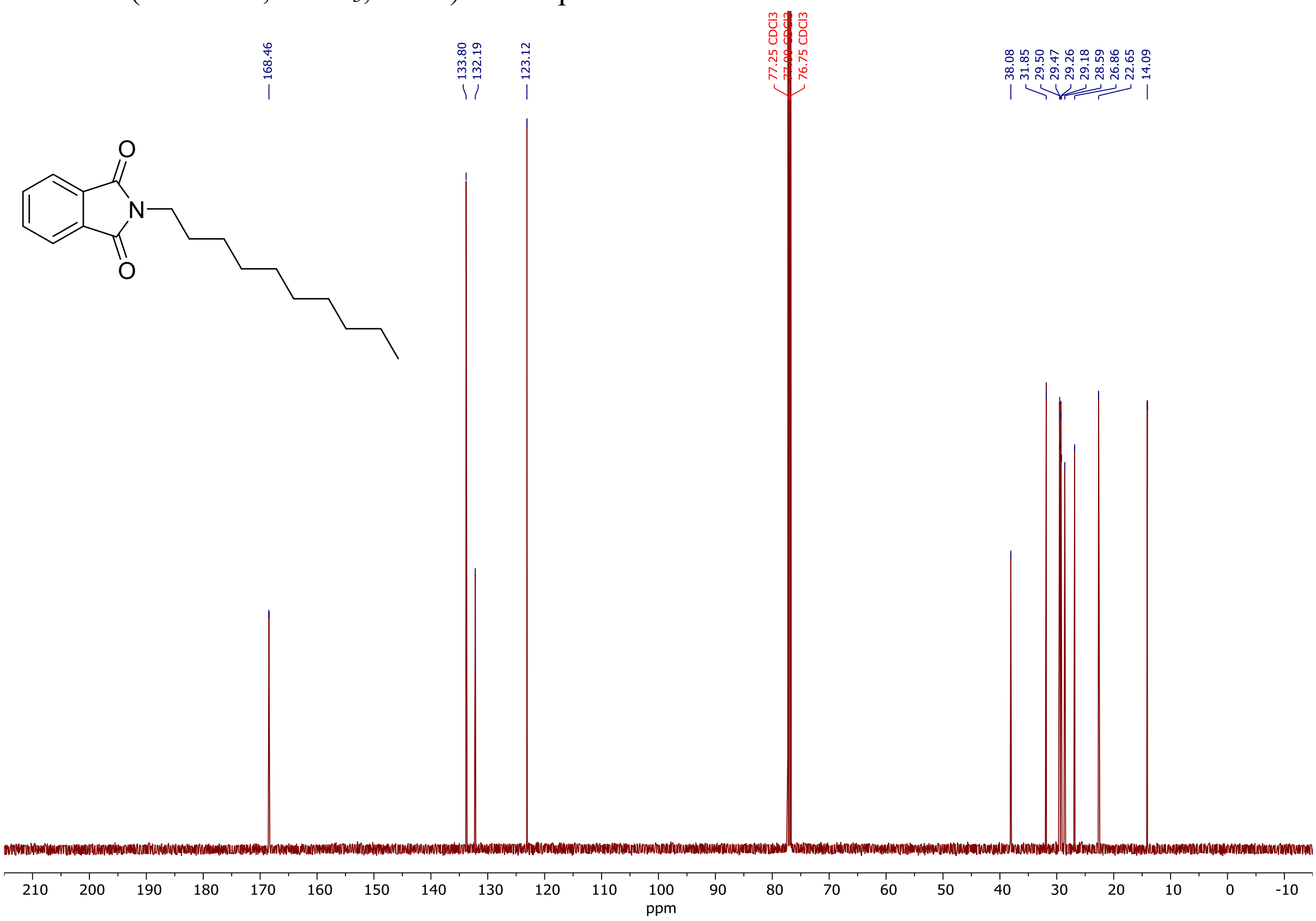

^{1}H NMR (500 MHz, $CDCl_3$, 298K) of compound **S11**

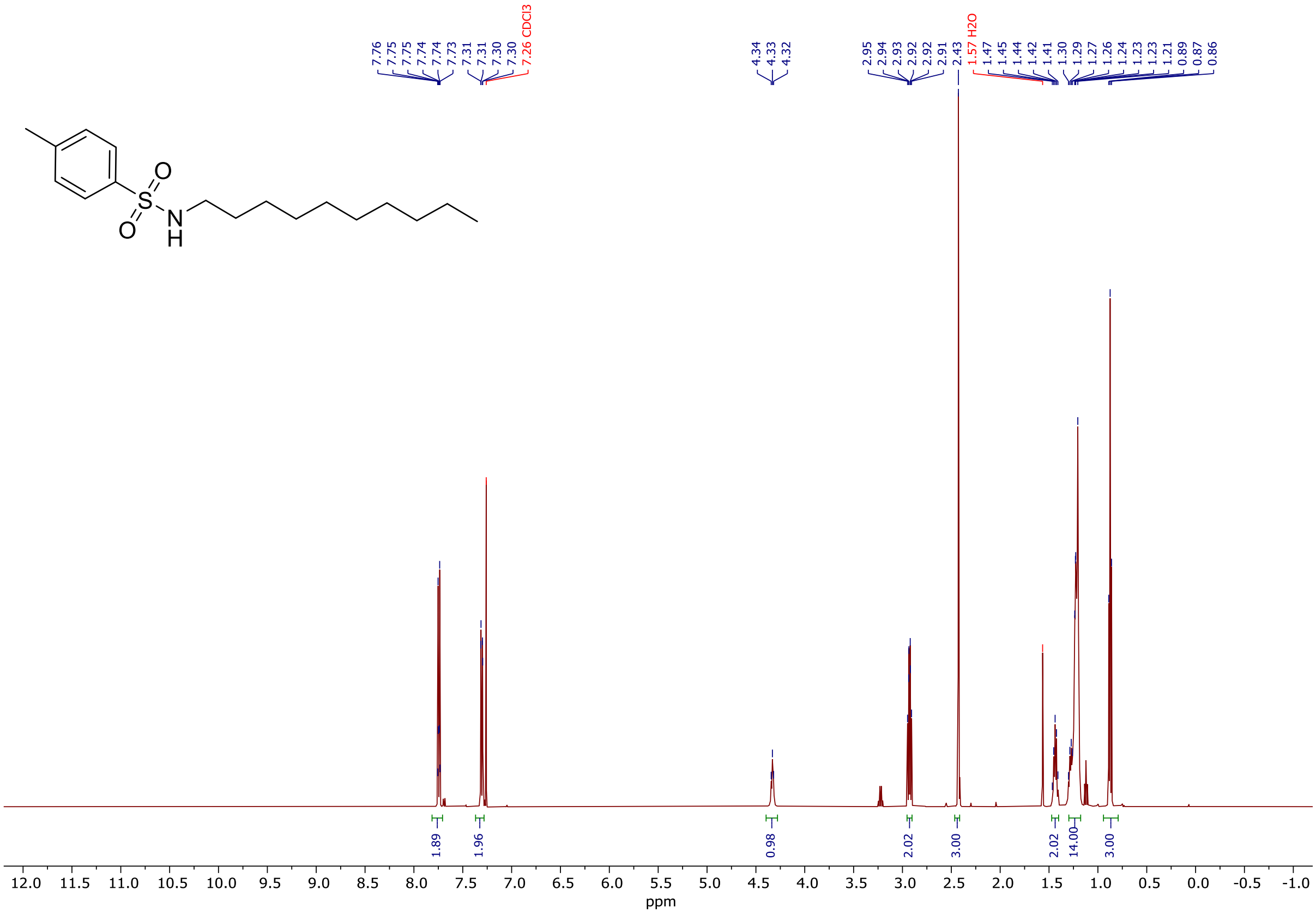

^{13}C NMR (125 MHz, $CDCl_3$, 298K) of compound **S11**

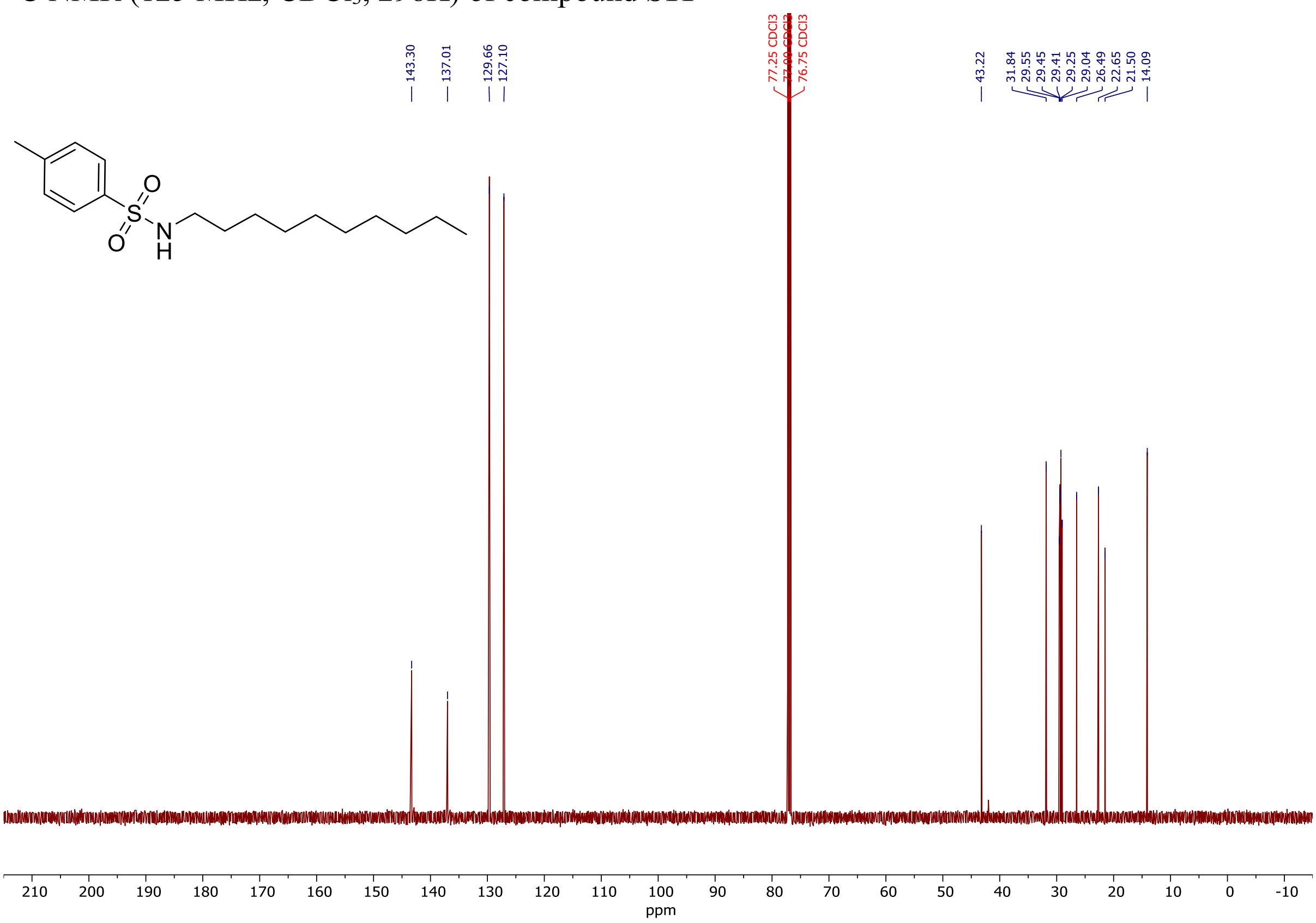

^{1}H NMR (500 MHz, $CDCl_3$, 298K) of compound **S12**

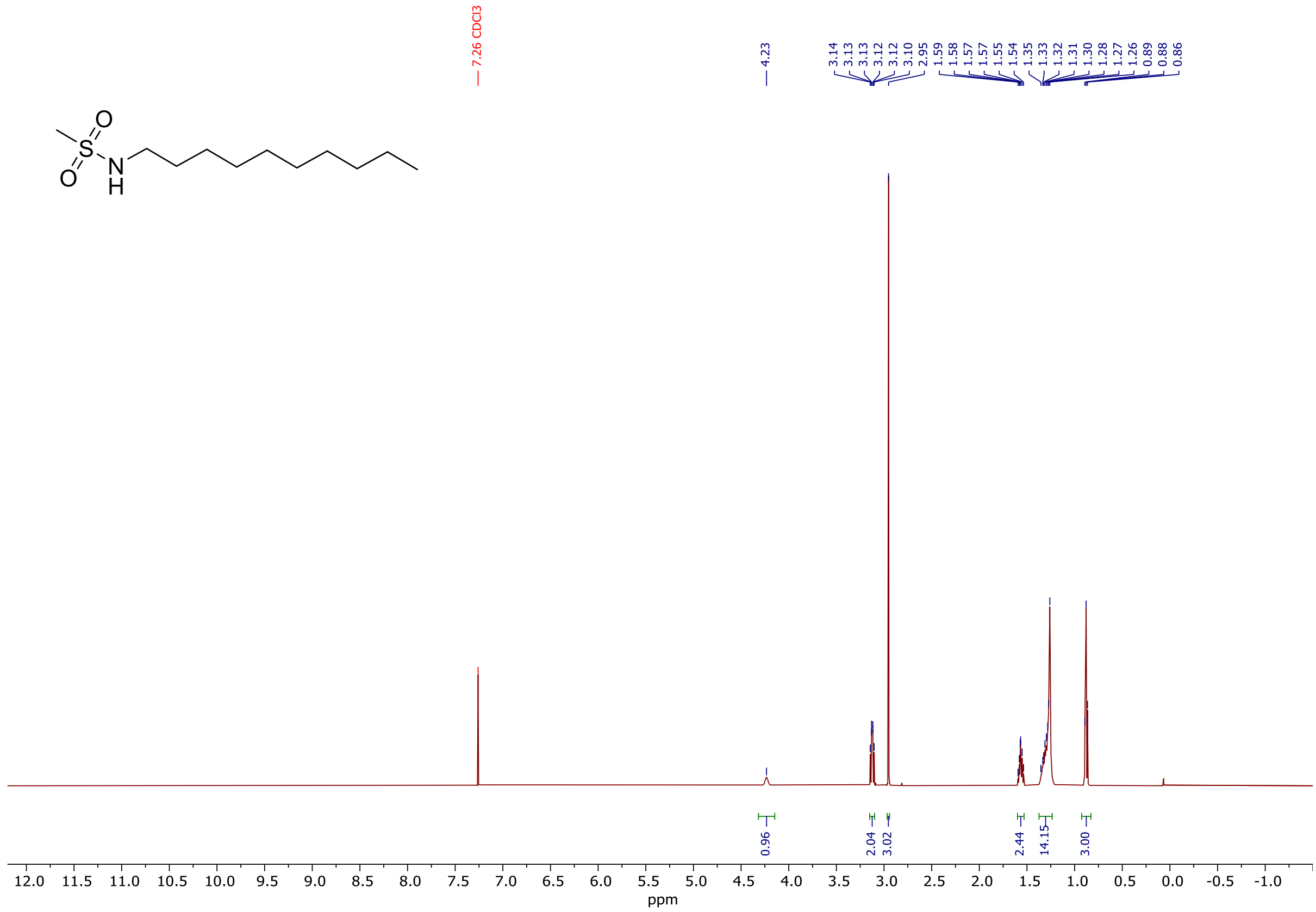

^{13}C NMR (125 MHz, $CDCl_3$, 298K) of compound **S12**

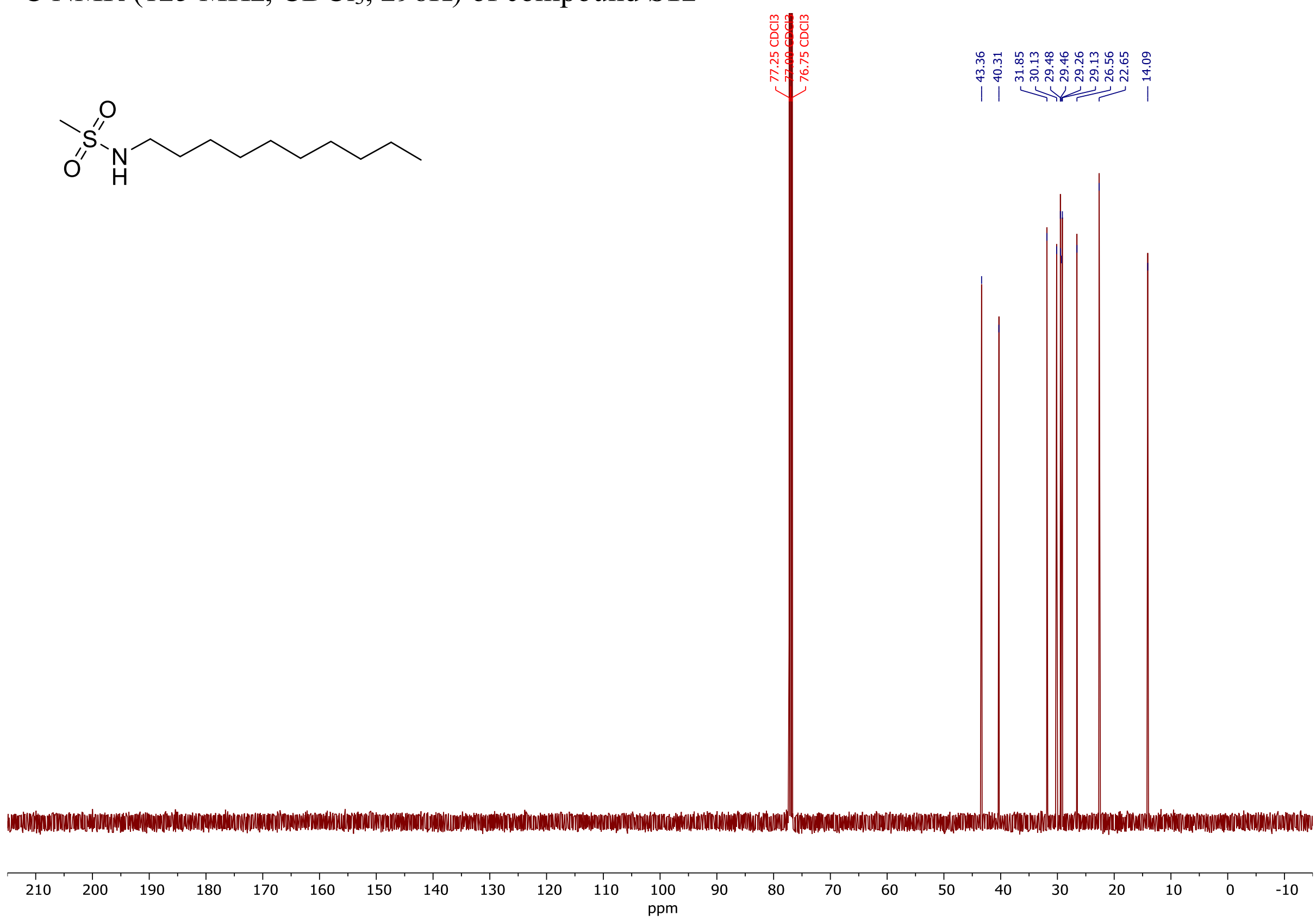

^{1}H NMR (500 MHz, $CDCl_3$, 298K) of compound **S13**

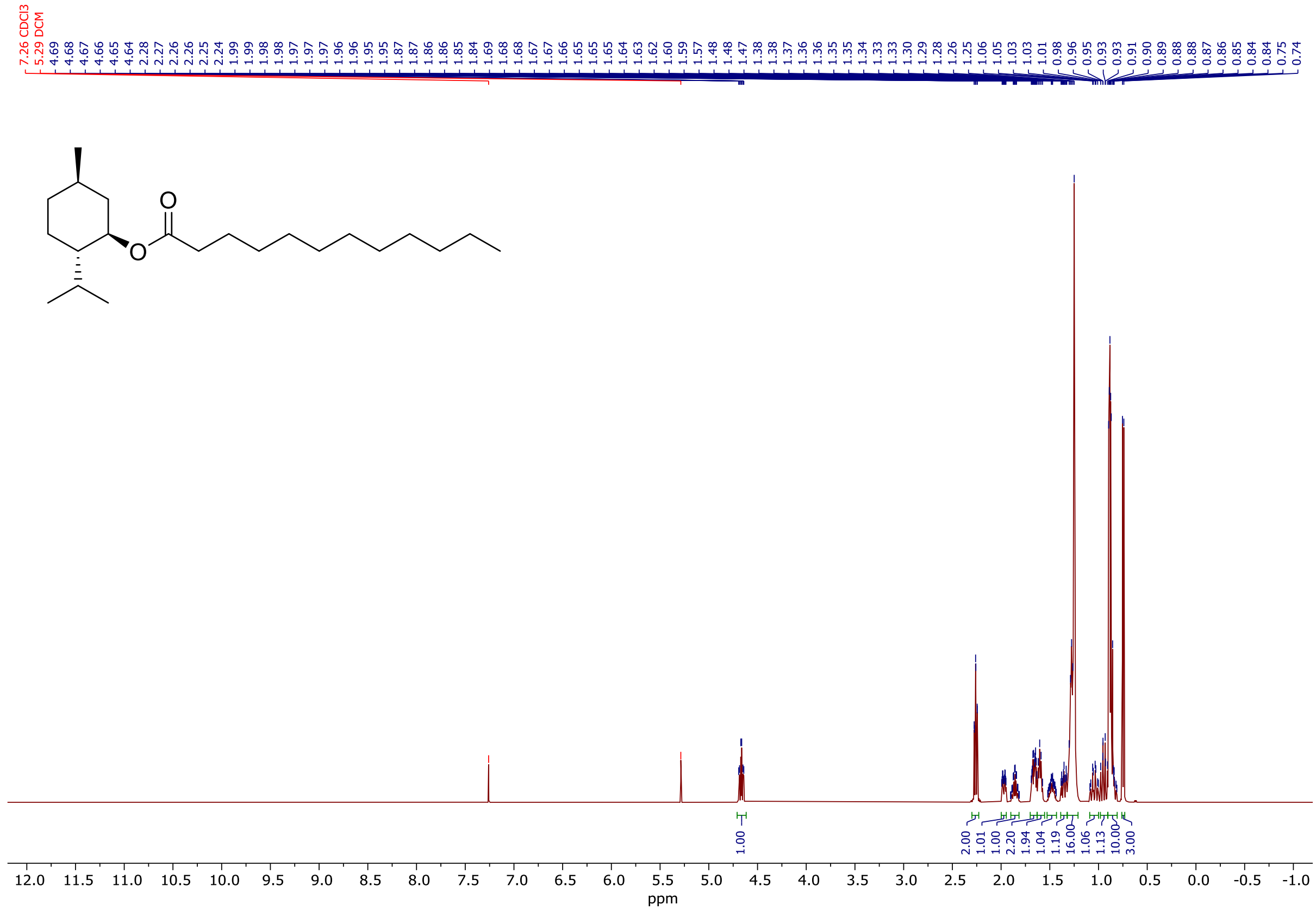

^{13}C NMR (125 MHz, $CDCl_3$, 298K) of compound **S13**

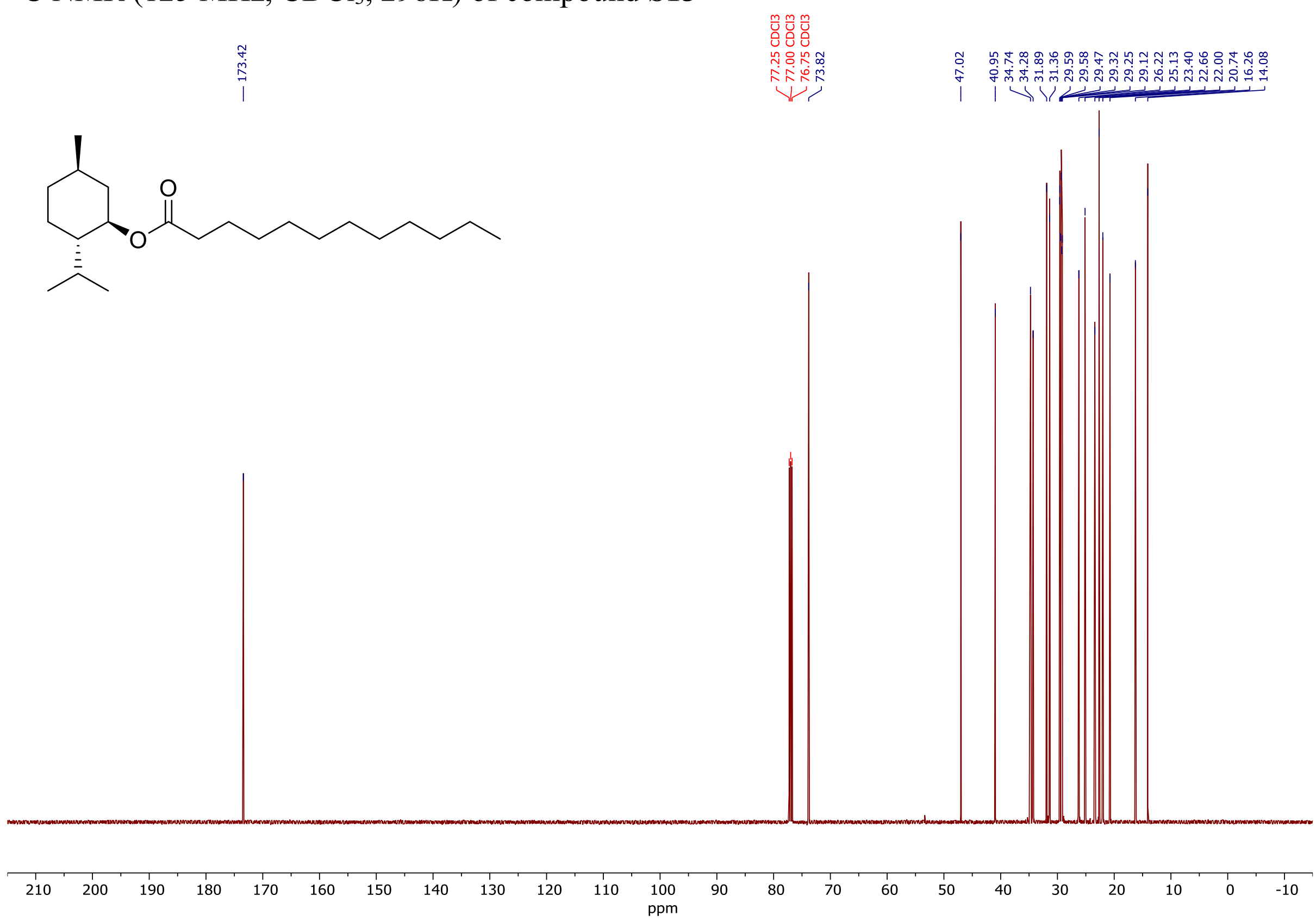

^{1}H NMR (500 MHz, $CDCl_3$, 298K) of compound ***cis*-SI-3**

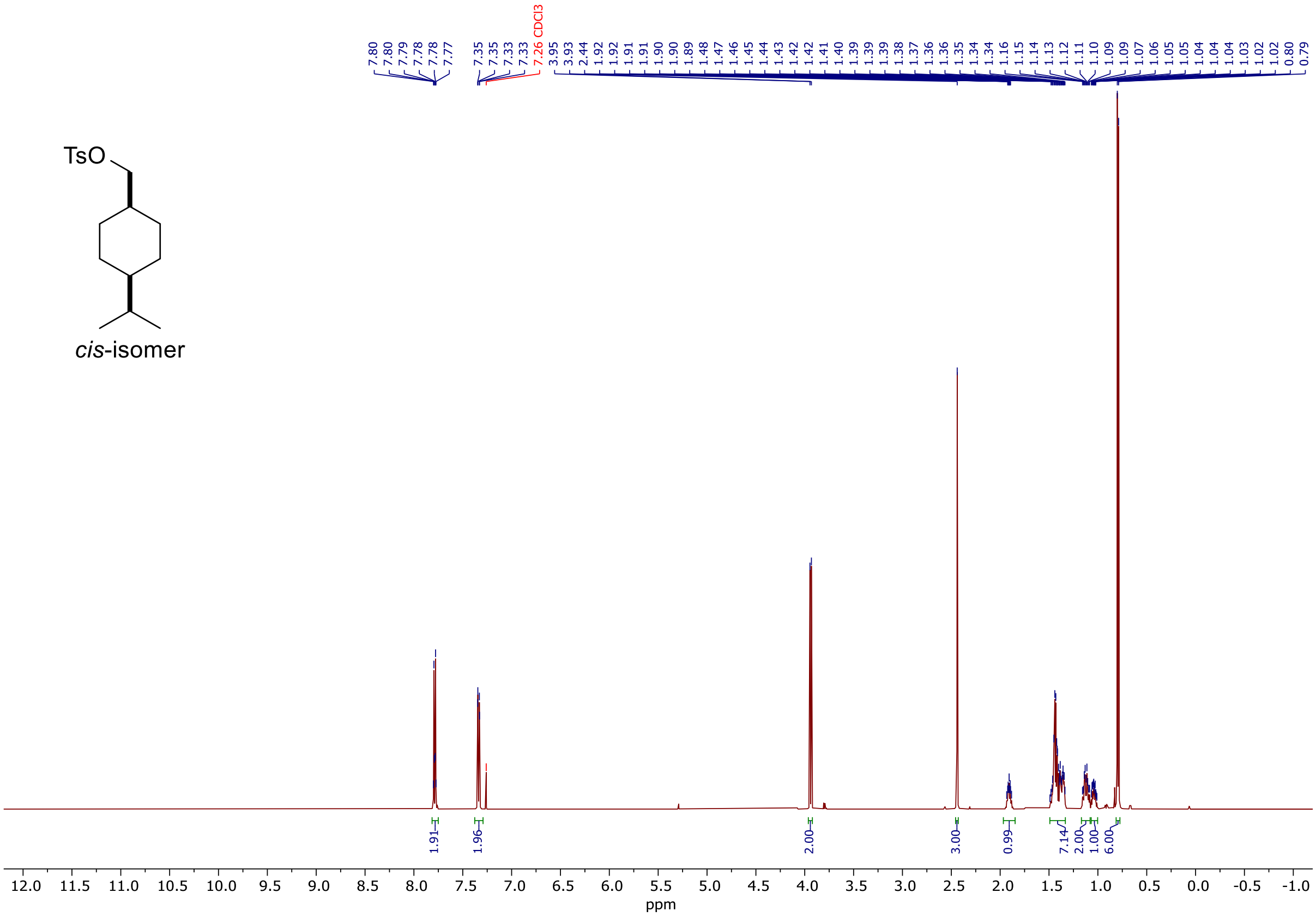

^{13}C NMR (125 MHz, $CDCl_3$, 298K) of compound ***cis*-SI-3**

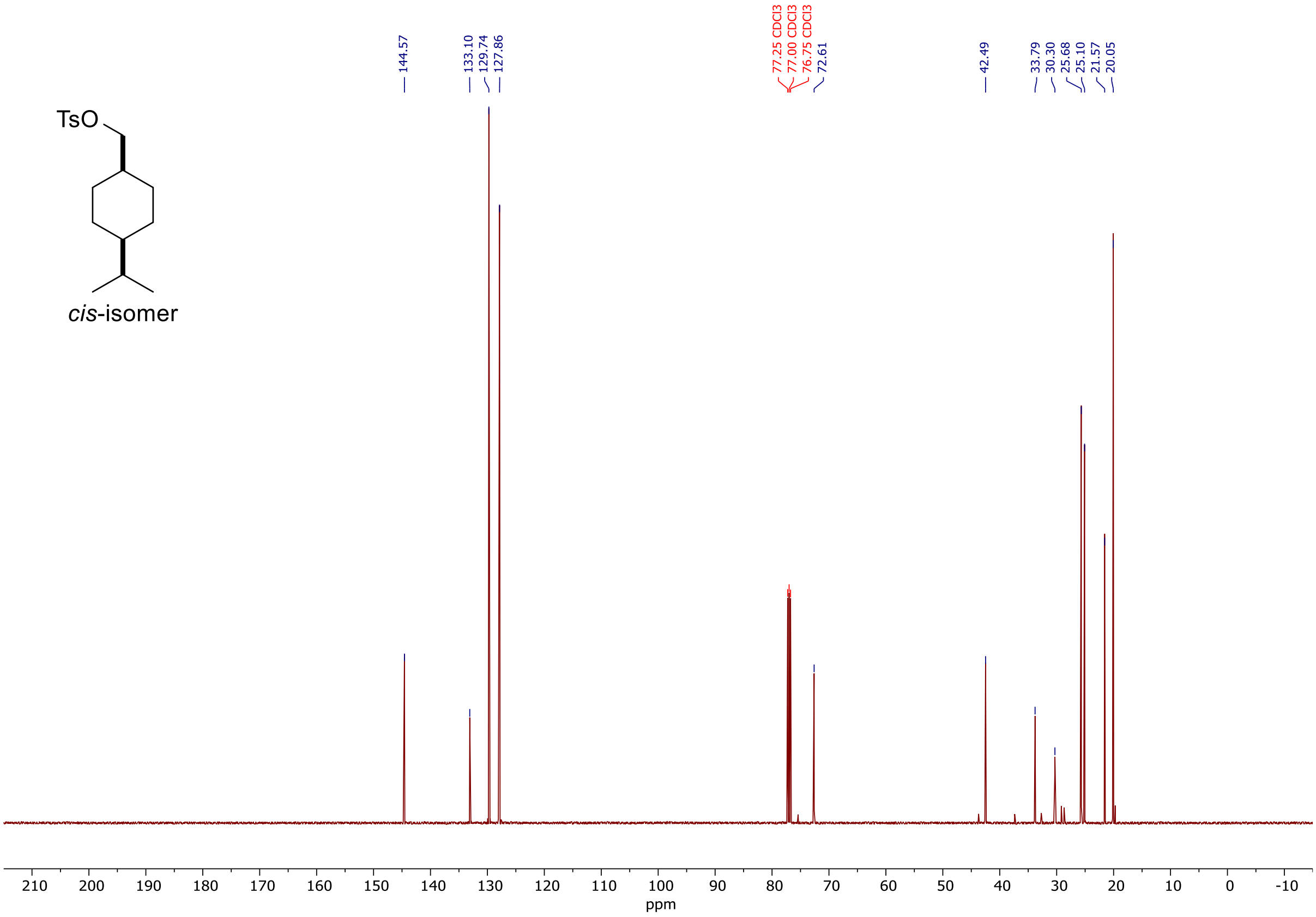

^{1}H NMR (500 MHz, $CDCl_3$, 298K) of compound ***trans*-SI-3**

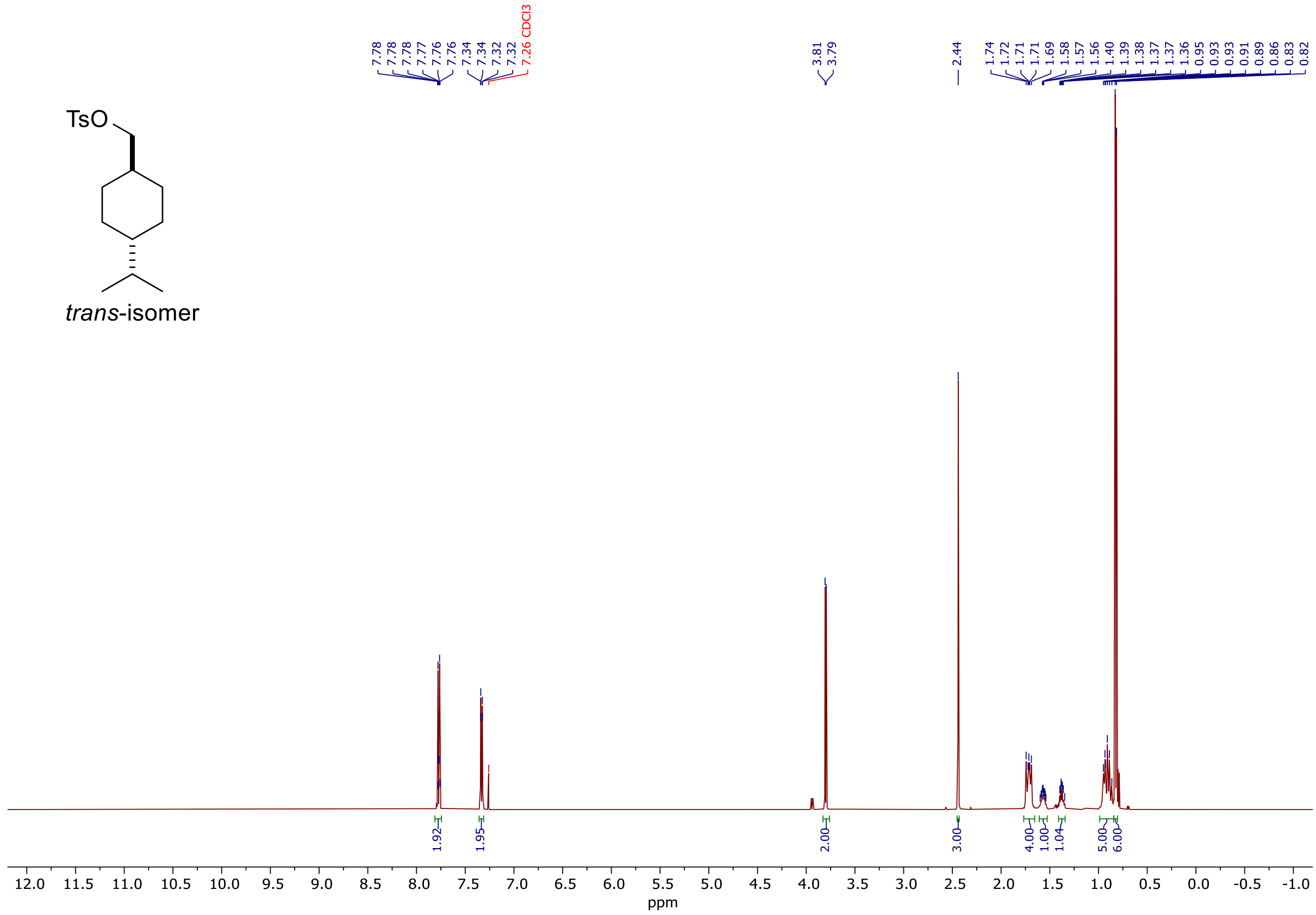

^{13}C NMR (125 MHz, $CDCl_3$, 298K) of compound ***trans*-SI-3**

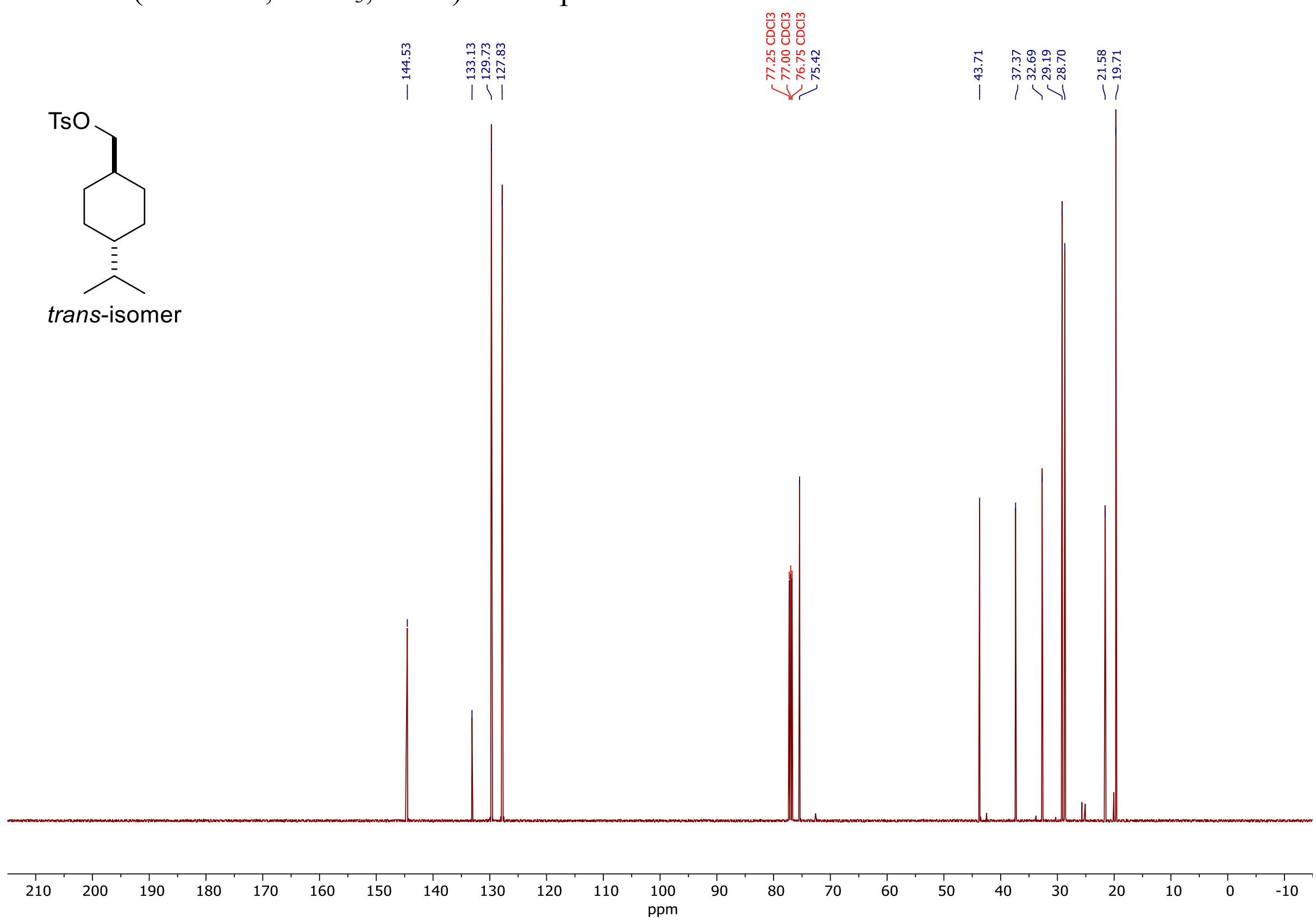

^{1}H NMR (500 MHz, $CDCl_3$, 298K) of compound **S16**

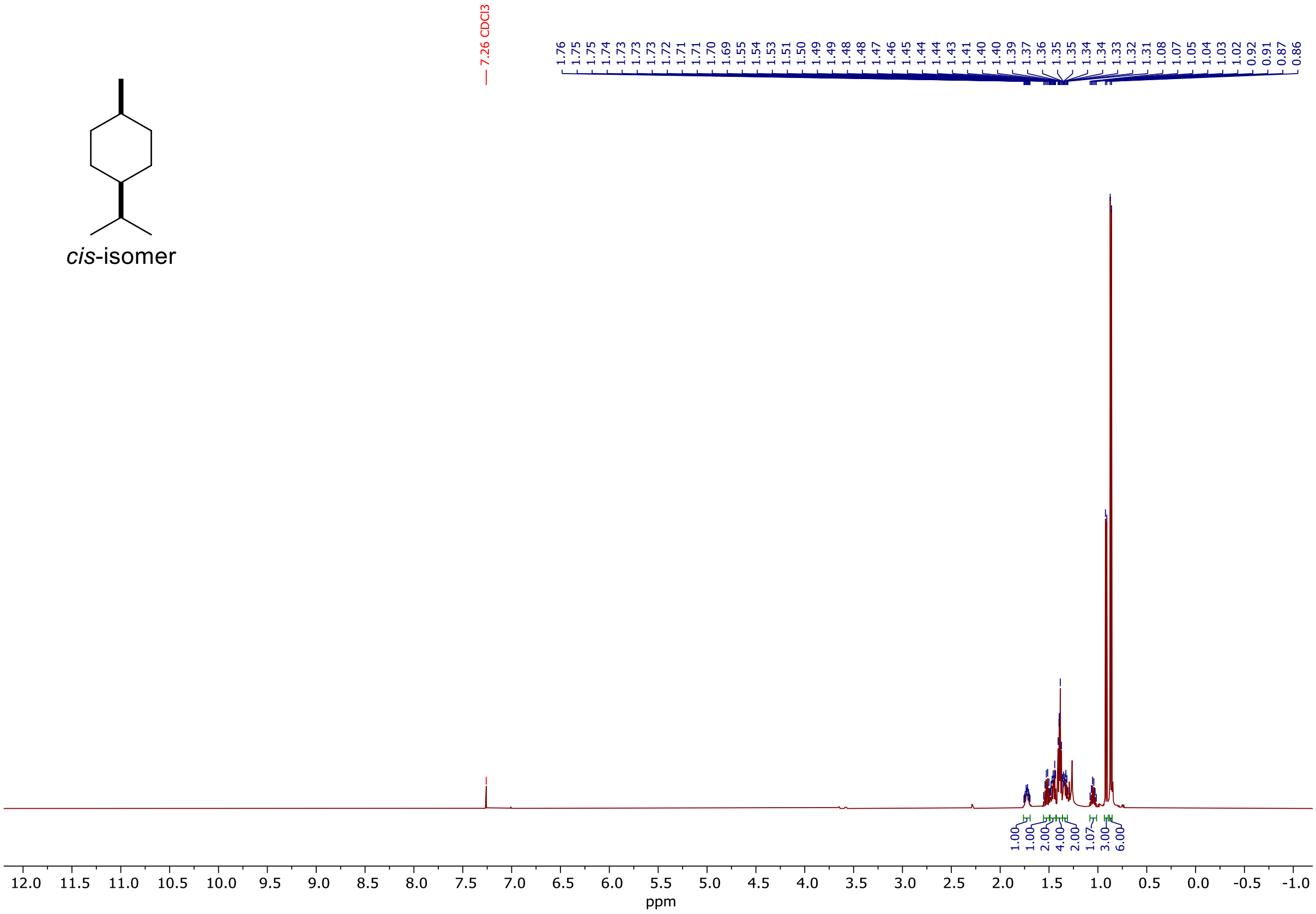

^{13}C NMR (125 MHz, $CDCl_3$, 298K) of compound **S16**

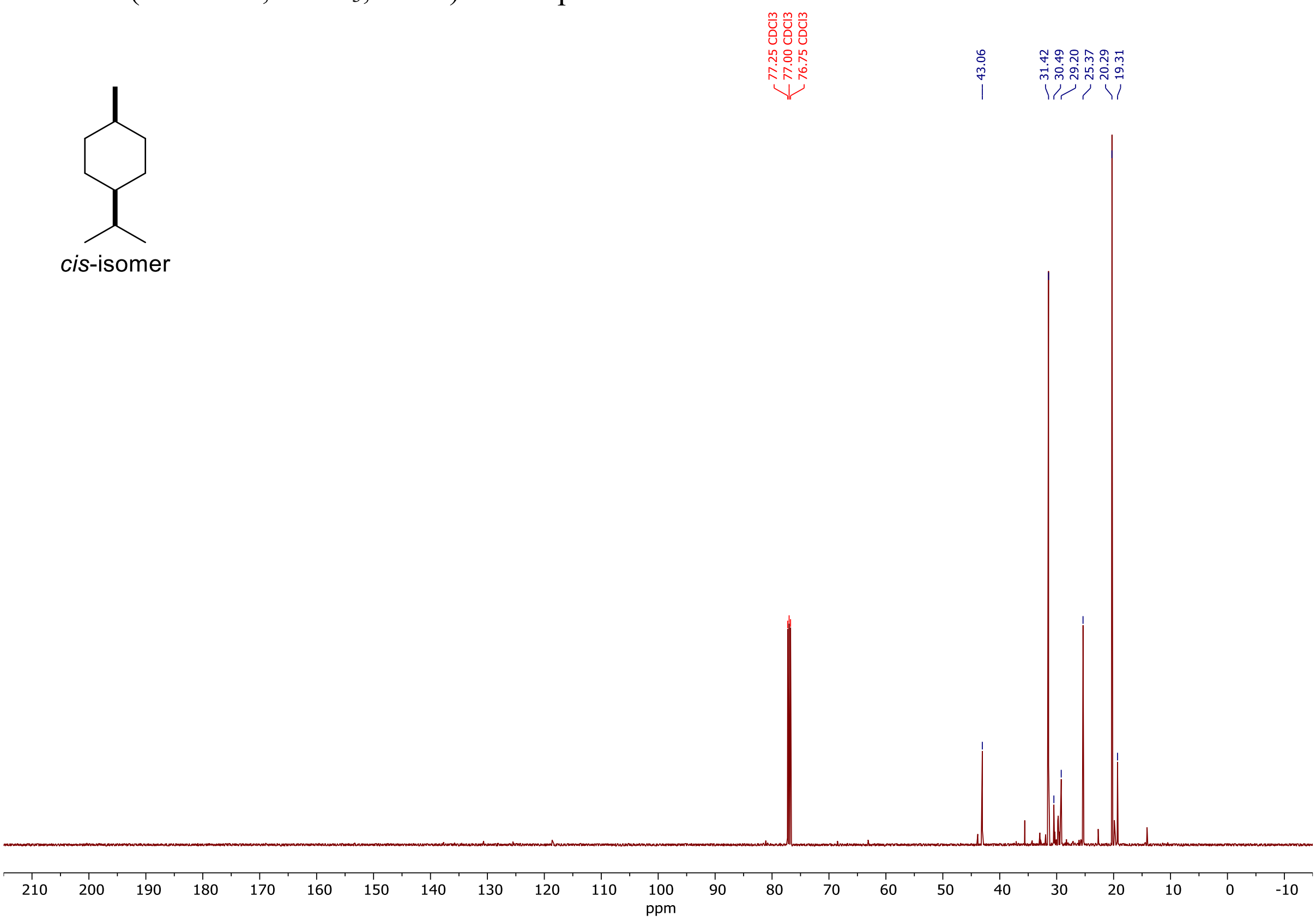

^{1}H NMR (500 MHz, $CDCl_3$, 298K) of compound **S18**

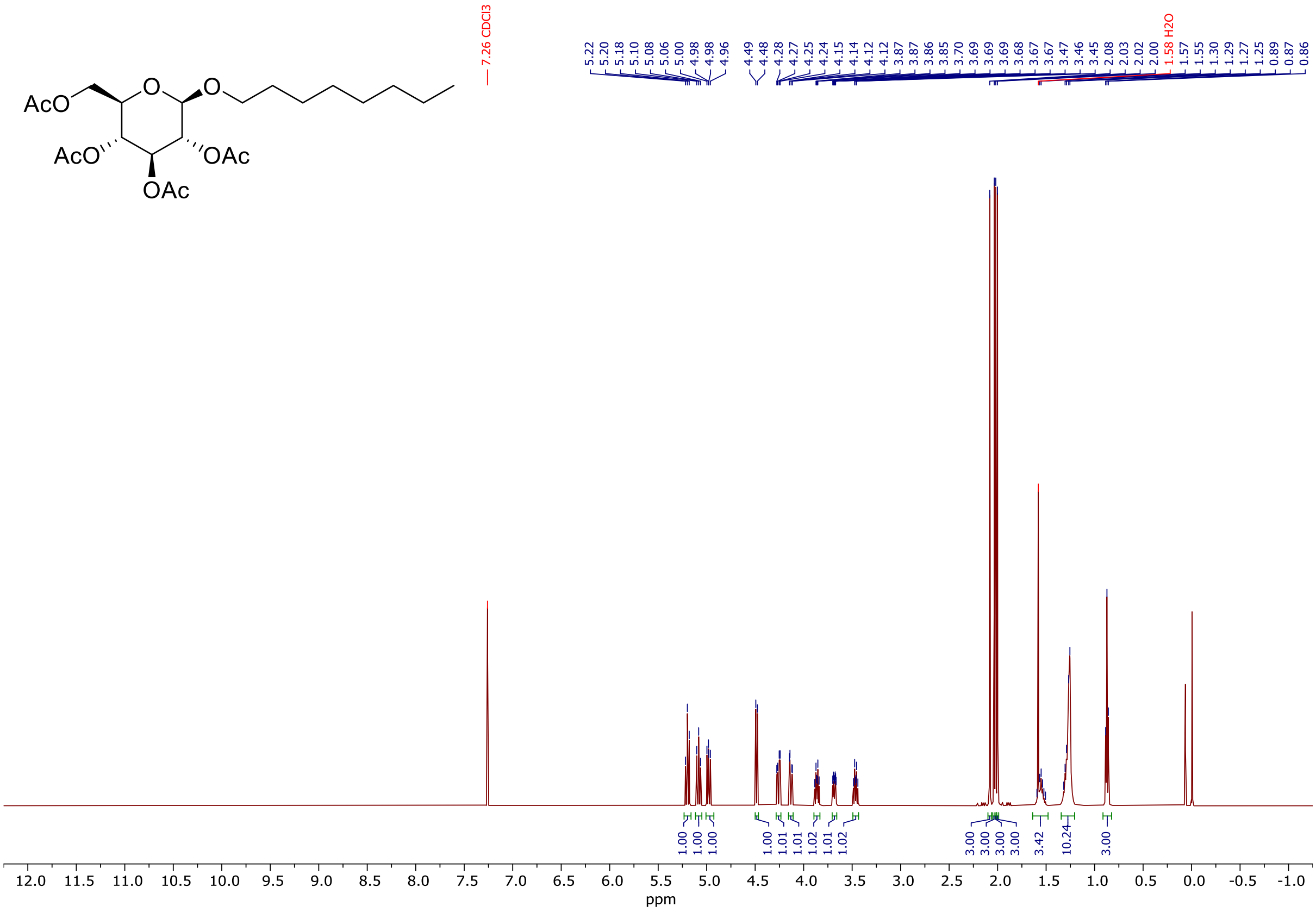

^{13}C NMR (125 MHz, $CDCl_3$, 298K) of compound **S18**

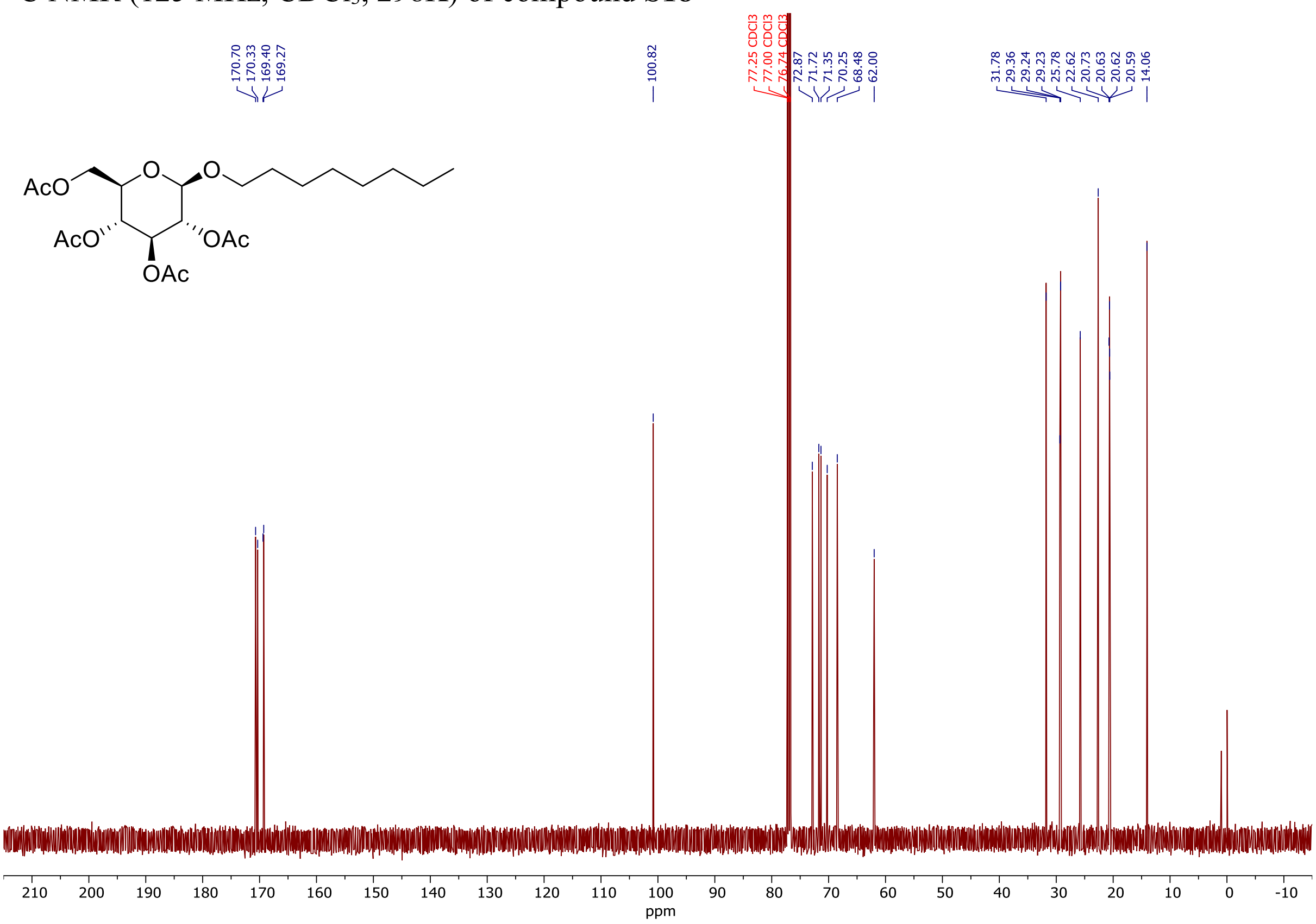

^{1}H-^{13}C HMQC ($CDCl_3$, 298K) of compound **S18**

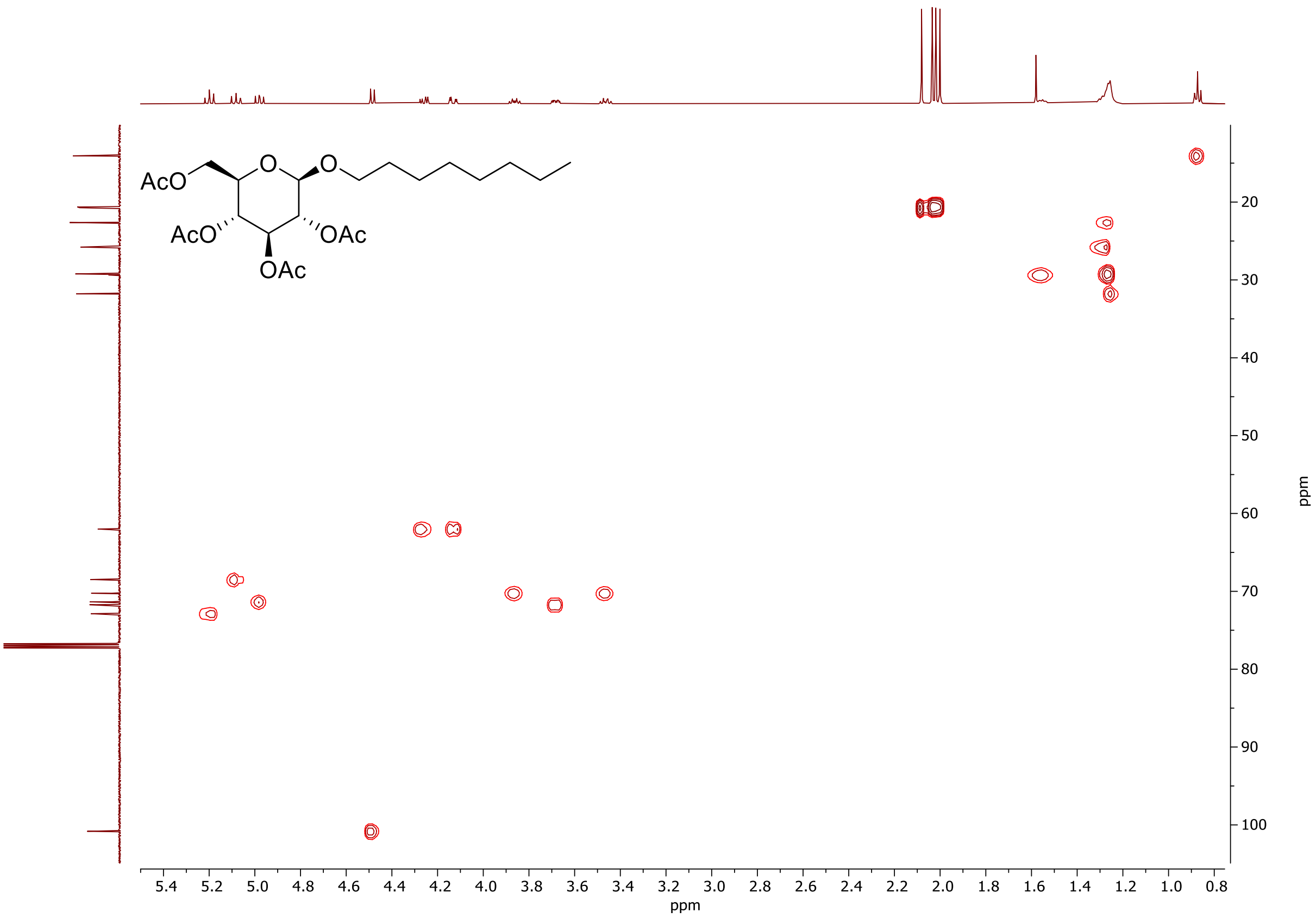

^{1}H NMR (500 MHz, $CDCl_3$, 298K) of compound **S19**

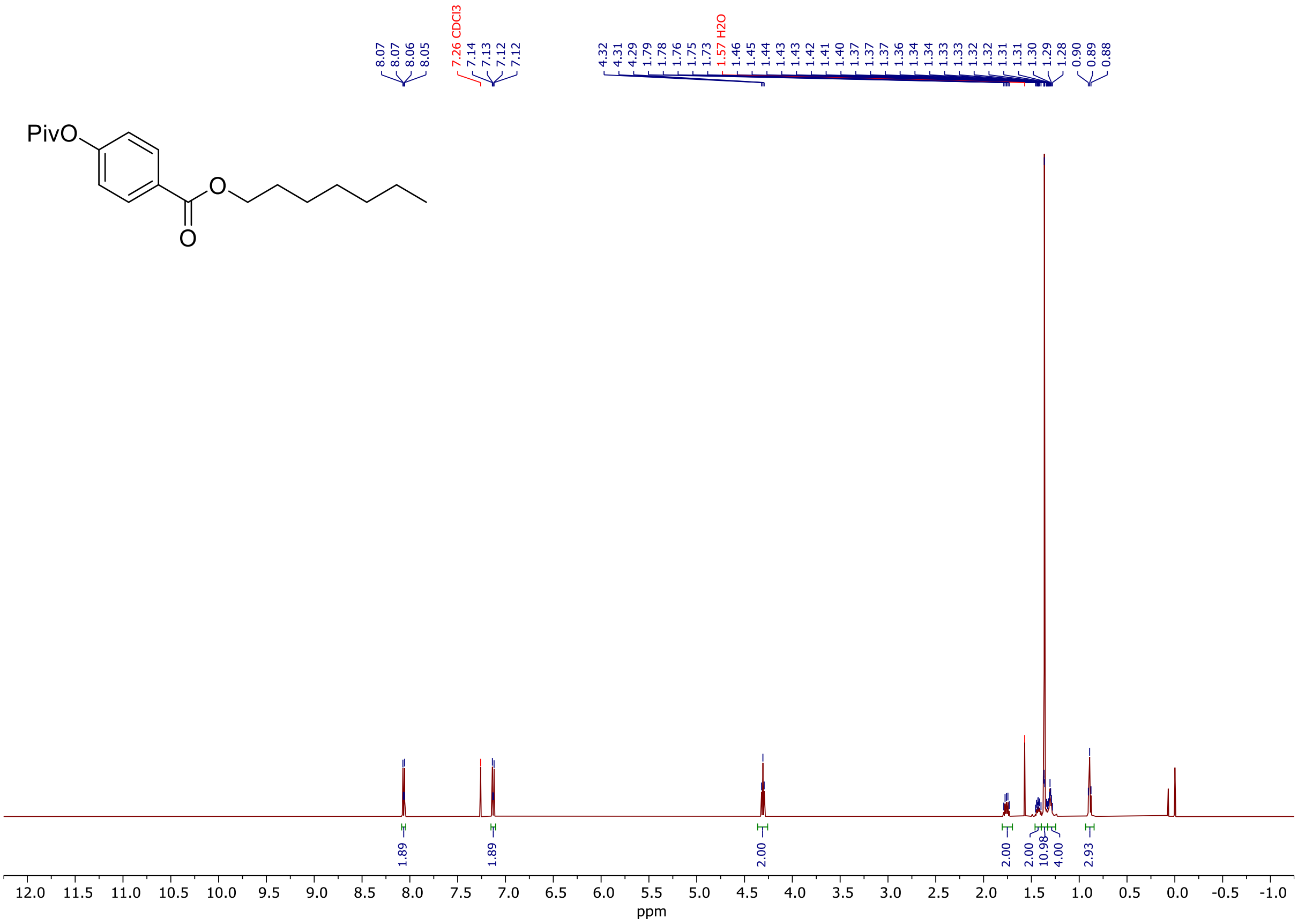

^{13}C NMR (125 MHz, $CDCl_3$, 298K) of compound **S19**

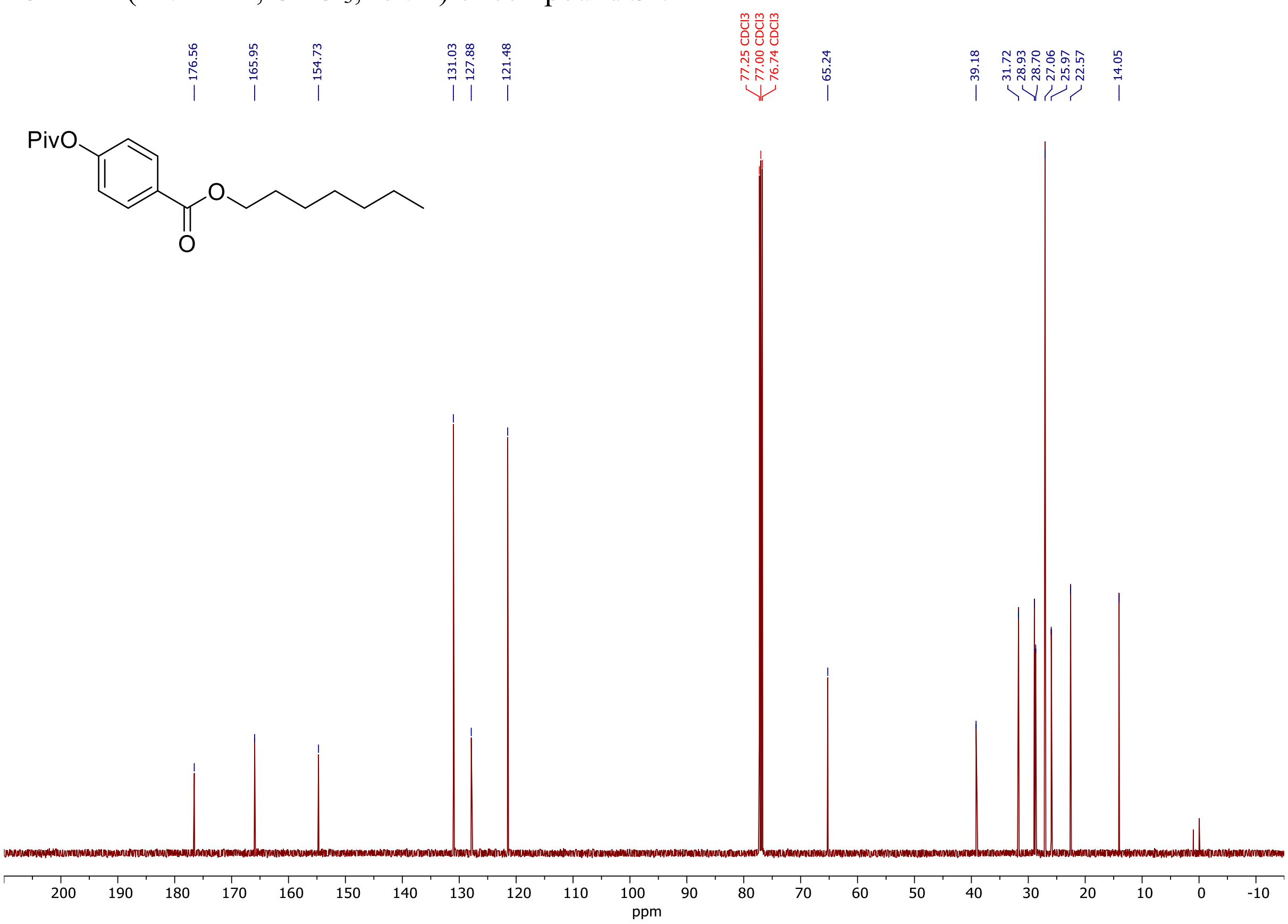

^{1}H-^{13}C HMQC ($CDCl_3$, 298K) of compound **S19**

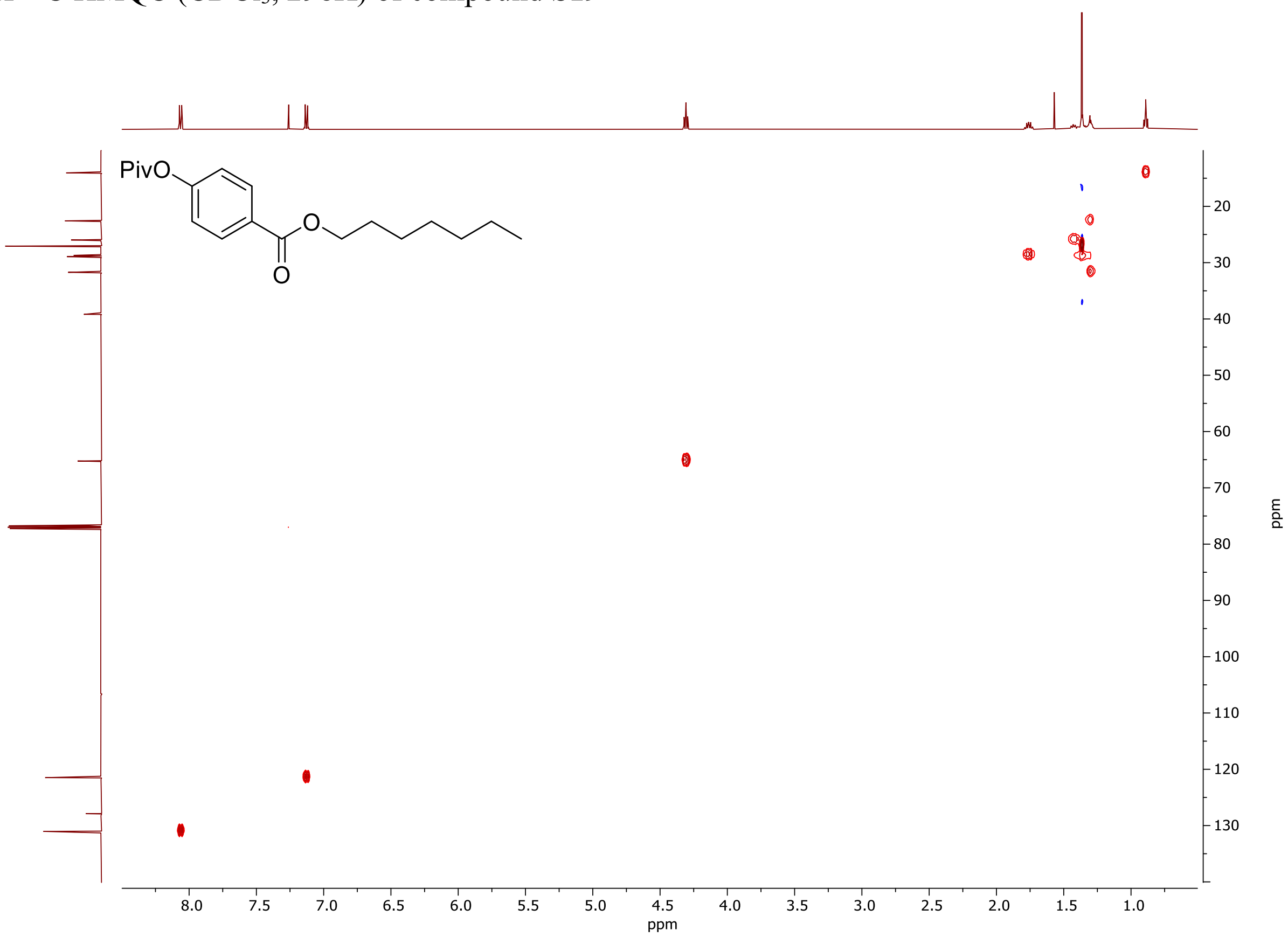

^{1}H NMR (500 MHz, $CDCl_3$, 298K) of C4-oxidized **S10**

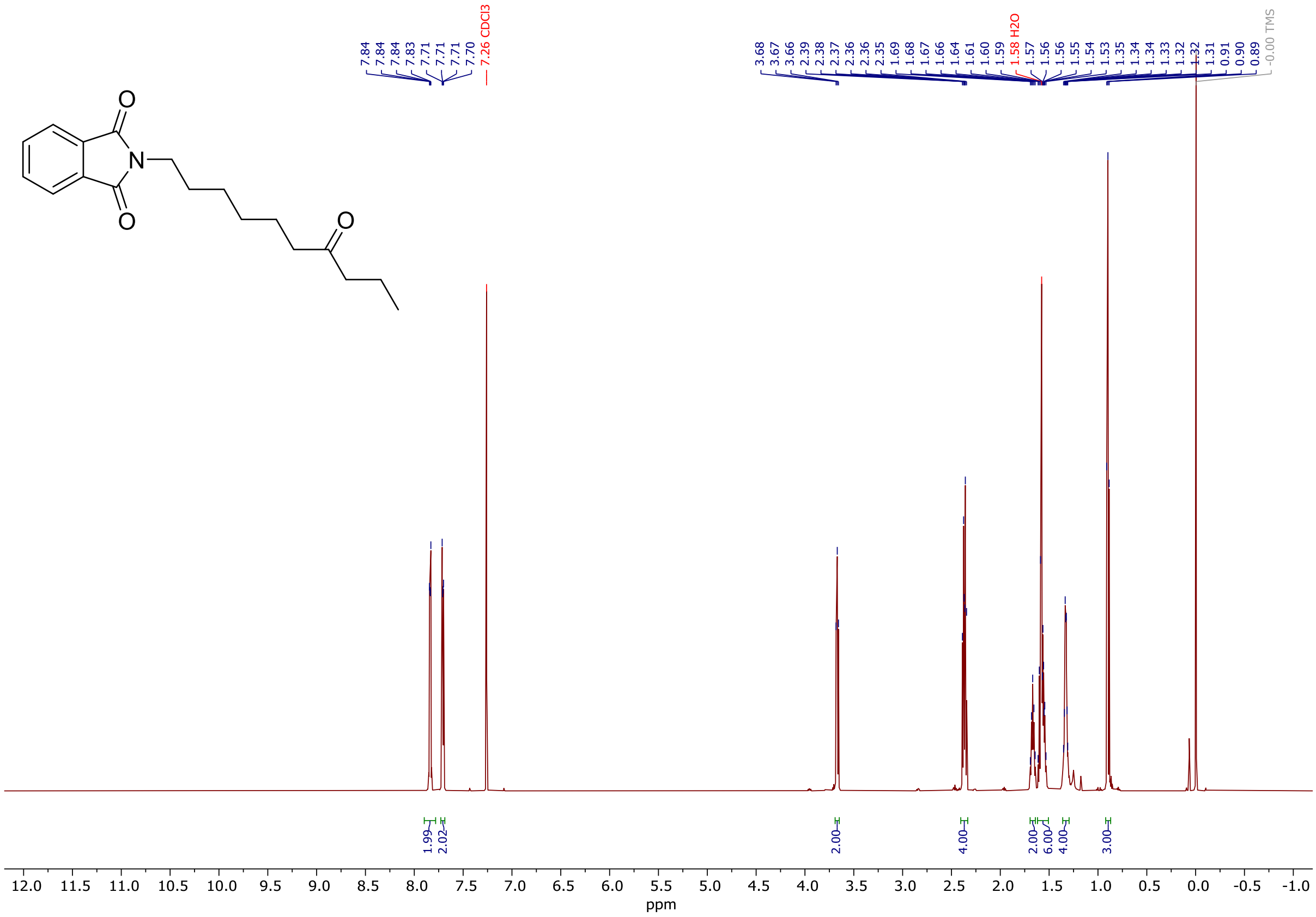

^{13}C NMR (125 MHz, $CDCl_3$, 298K) of C4-oxidized **S10**

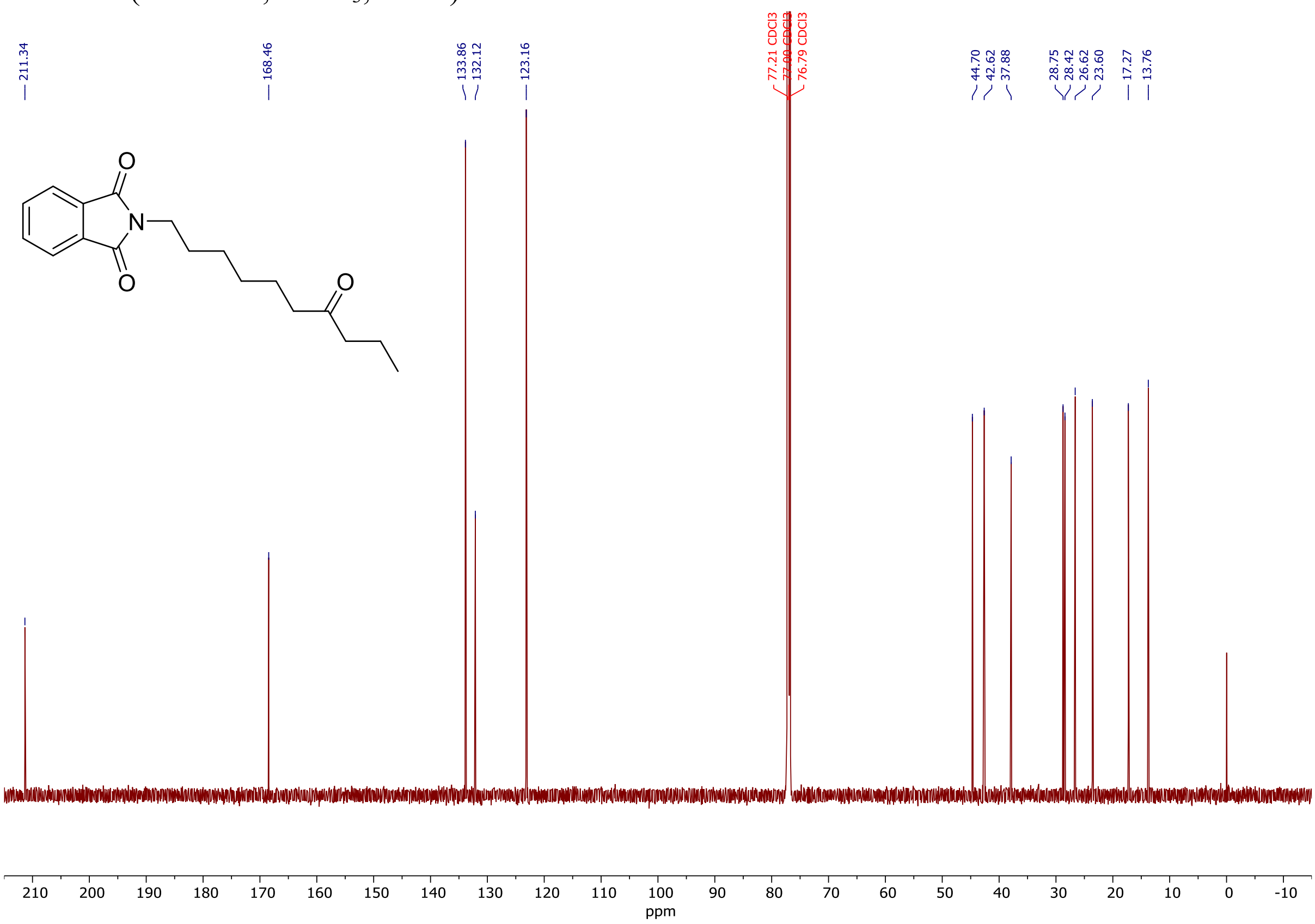

^{1}H-^{13}C HMQC ($CDCl_3$, 298K) of C4-oxidized **S10**

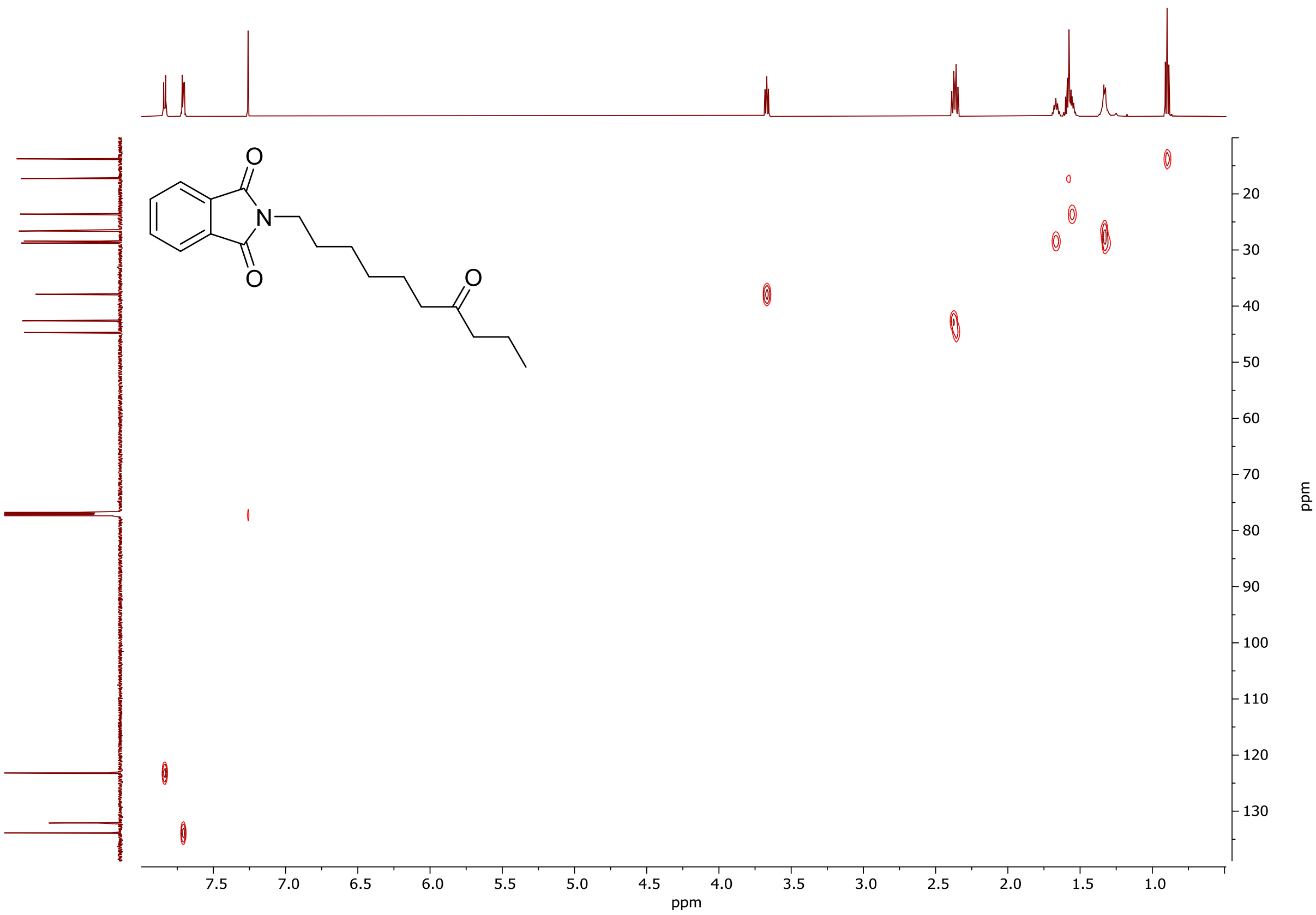

^{1}H-^{13}C HMBC ($CDCl_3$, 298K) of C4-oxidized **S10**

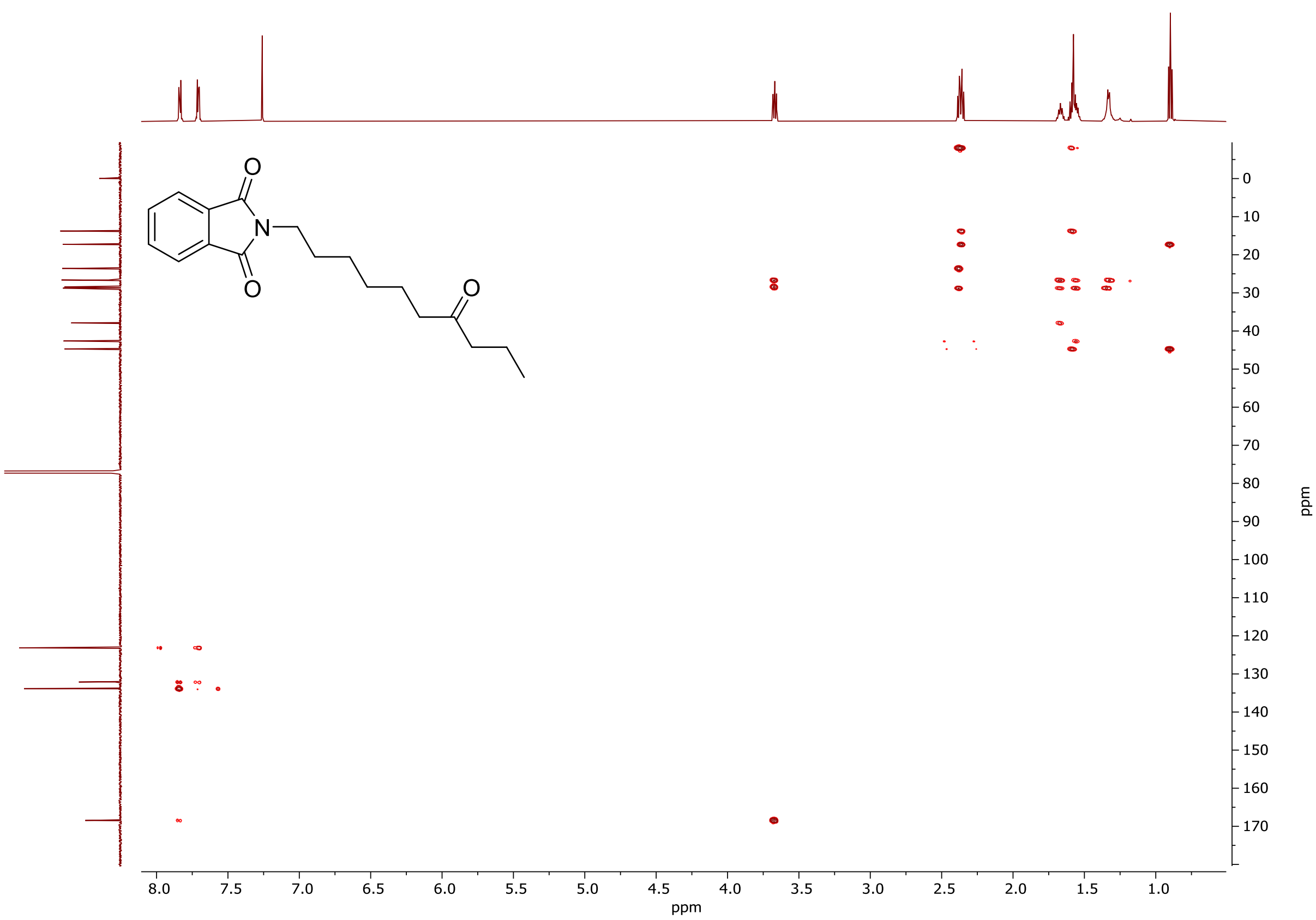

^{1}H NMR (500 MHz, $CDCl_3$, 298K) of C4-oxidized **S11**

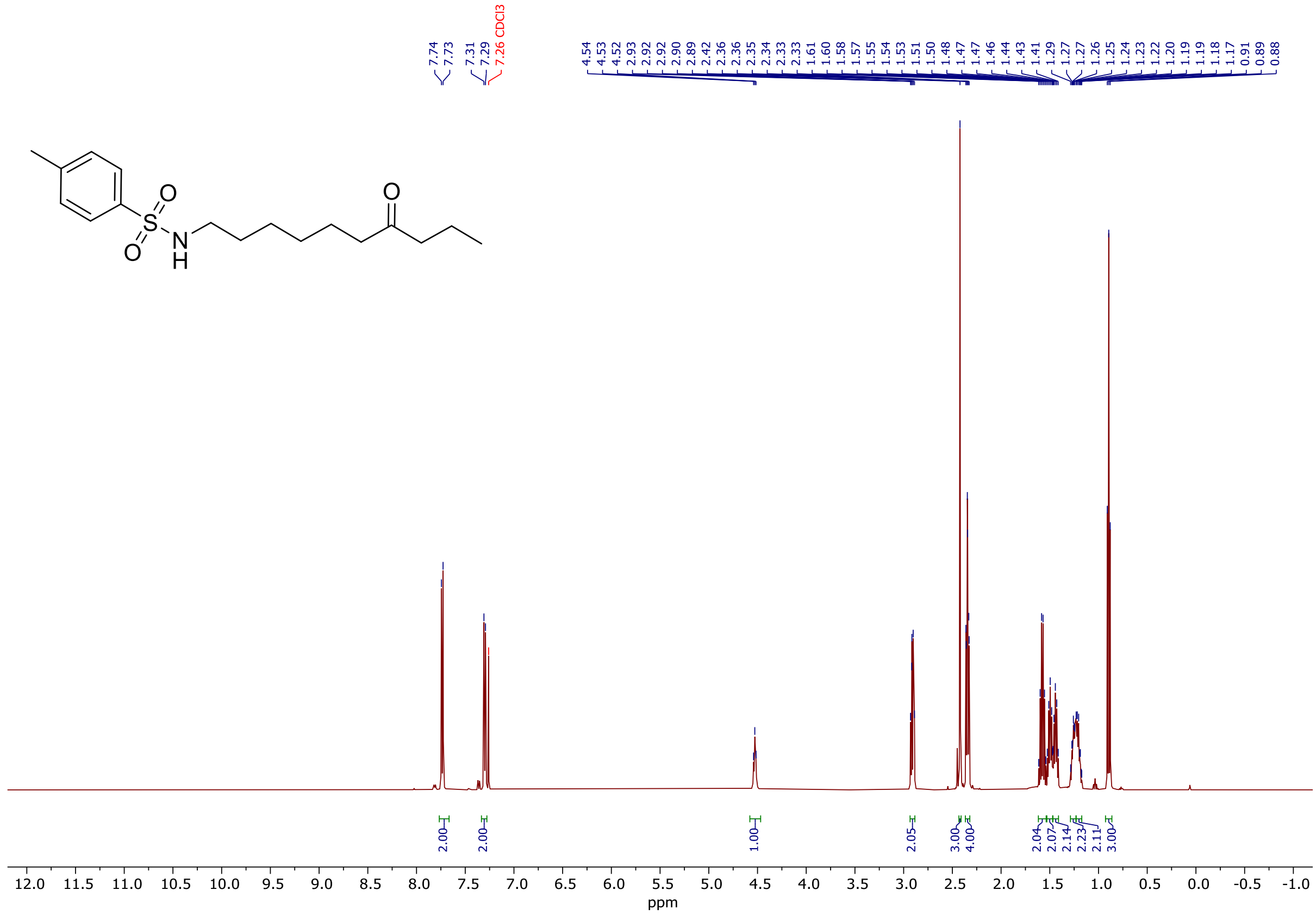

^{13}C NMR (125 MHz, $CDCl_3$, 298K) of C4-oxidized **S11**

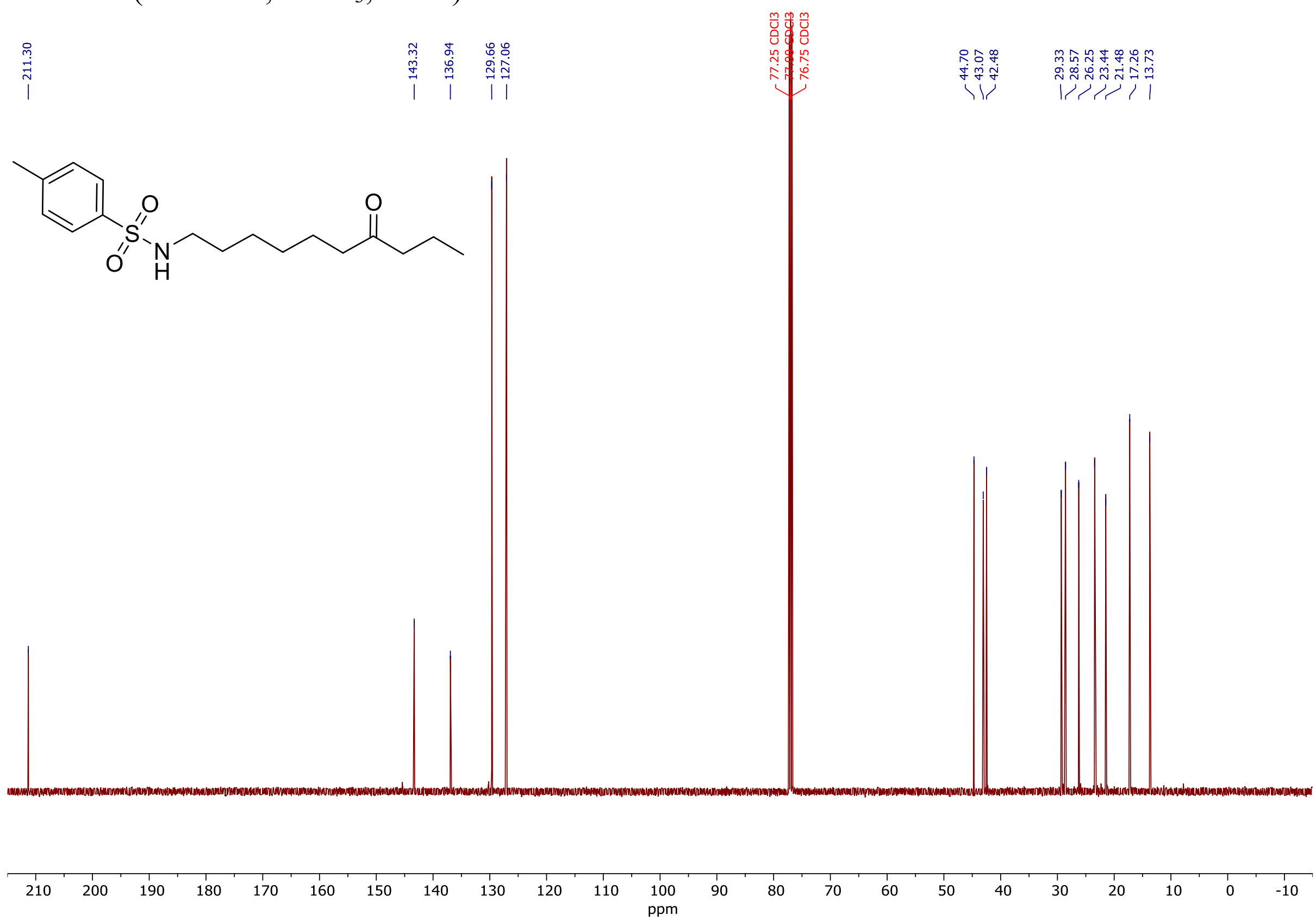

^{1}H-^{13}C HMQC ($CDCl_3$, 298K) of C4-oxidized **S11**

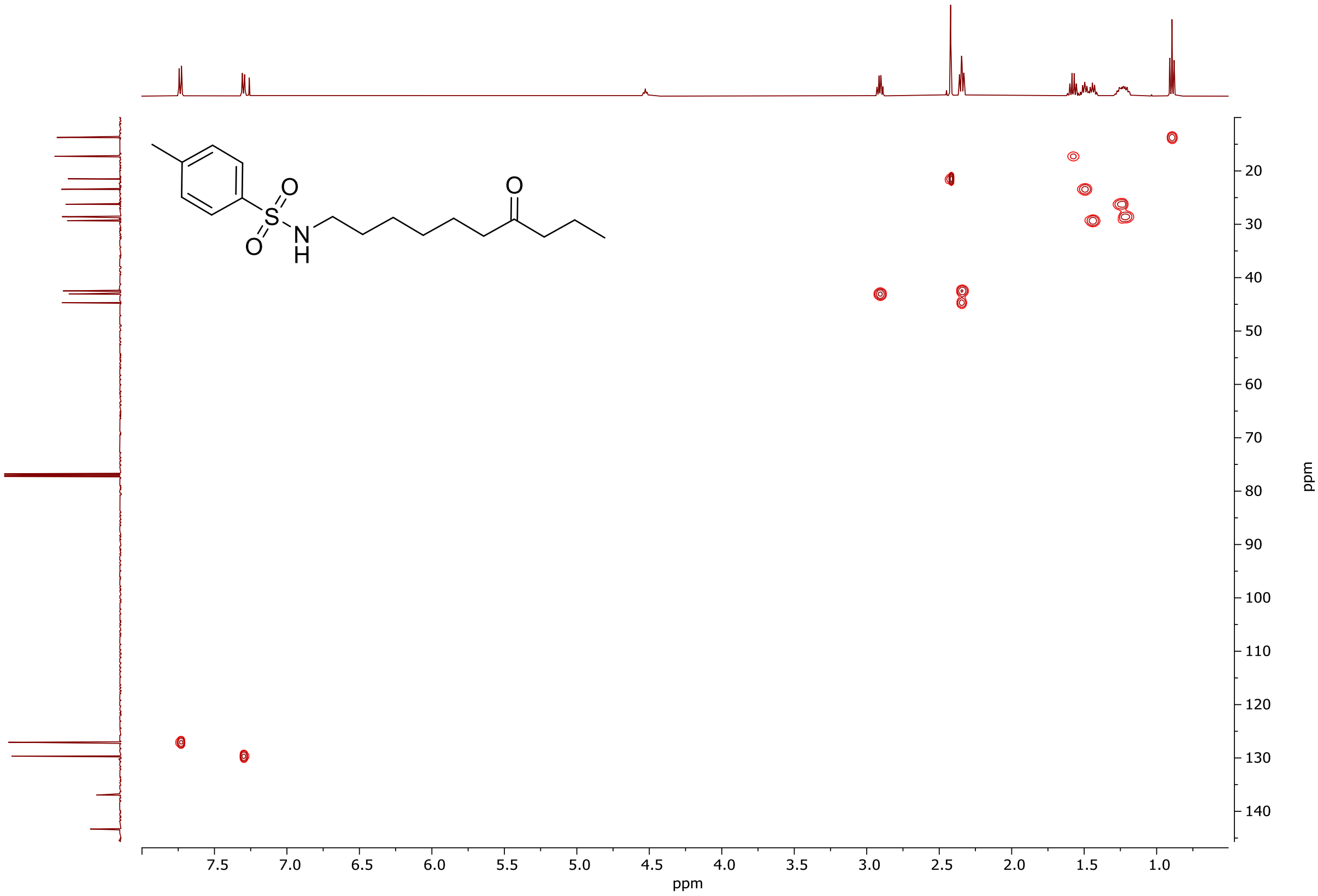

^{1}H-^{13}C HMBC ($CDCl_3$, 298K) of C4-oxidized **S11**

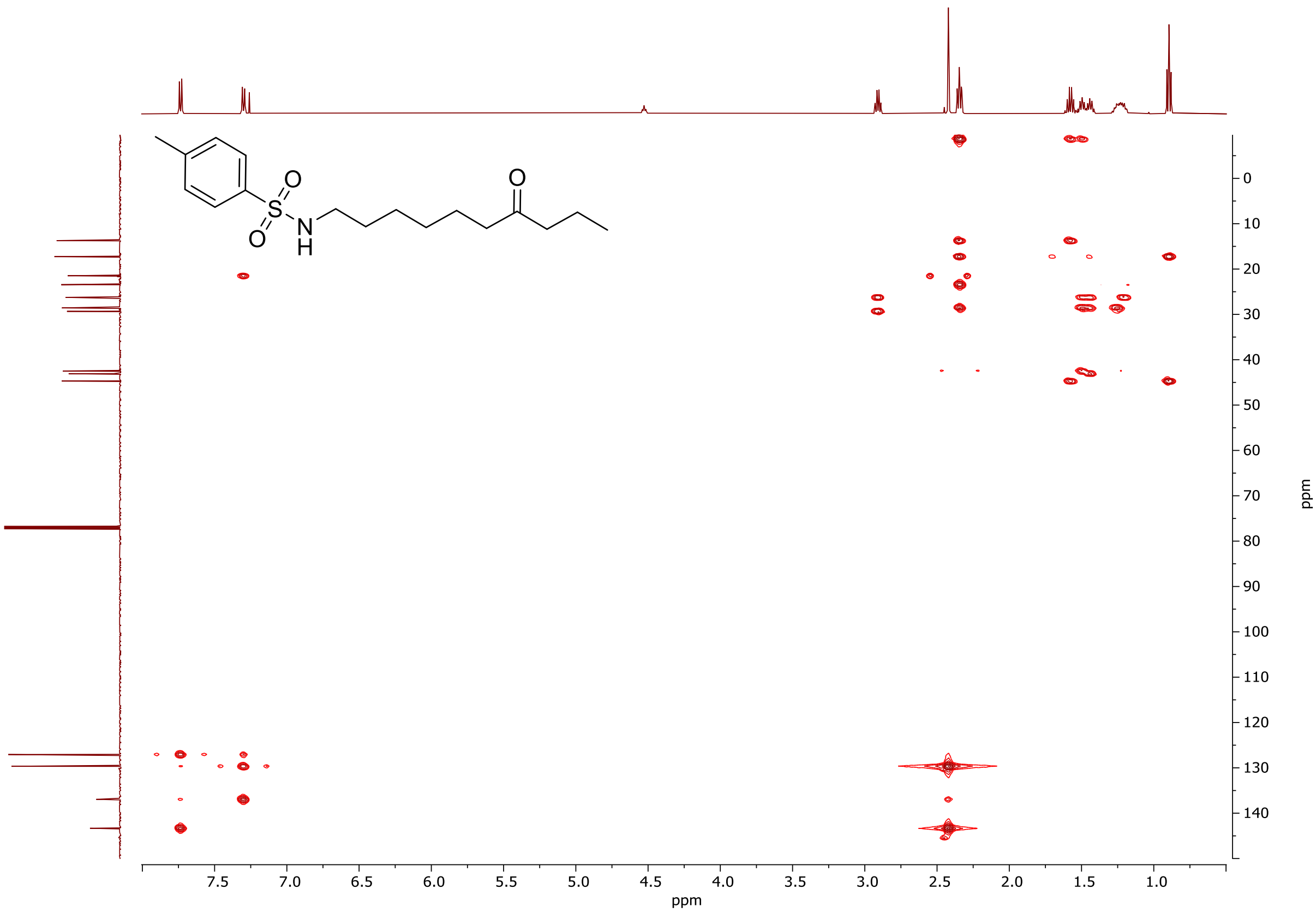

^{1}H NMR (500 MHz, $CDCl_3$, 298K) of C4-oxidized **S12**

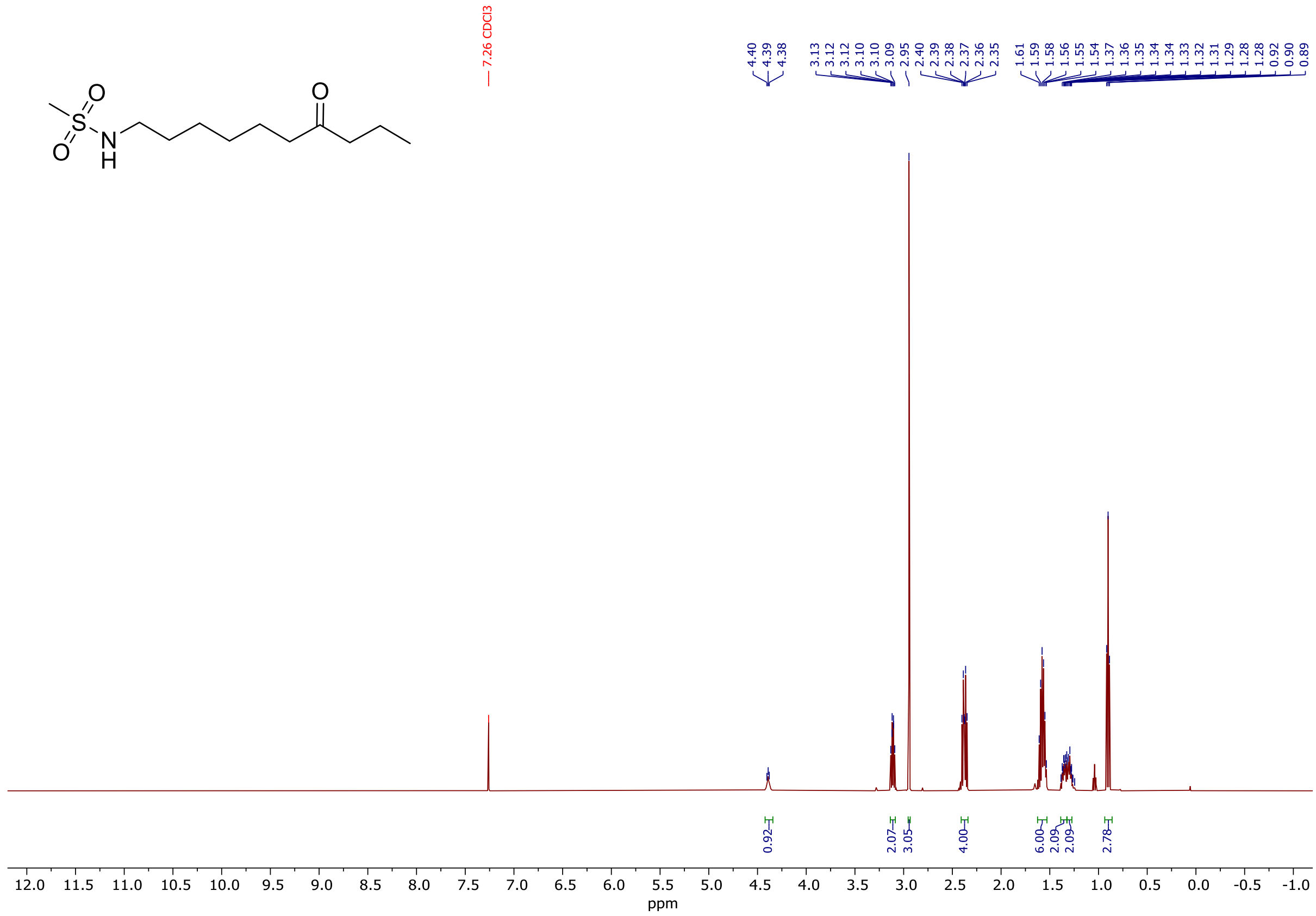

^{13}C NMR (125 MHz, $CDCl_3$, 298K) of C4-oxidized **S12**

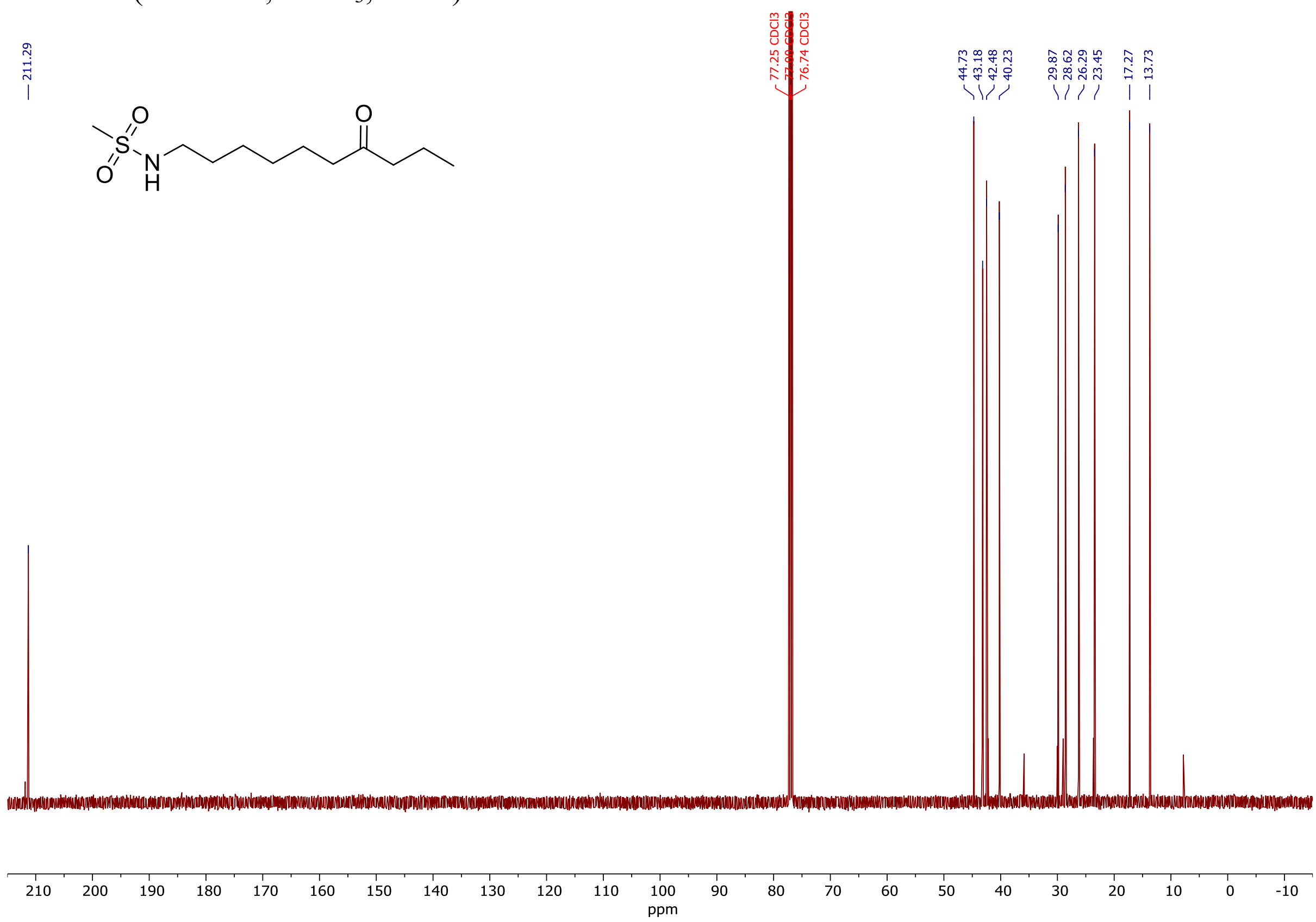

^{1}H-^{13}C HMQC ($CDCl_3$, 298K) of C4-oxidized **S12**

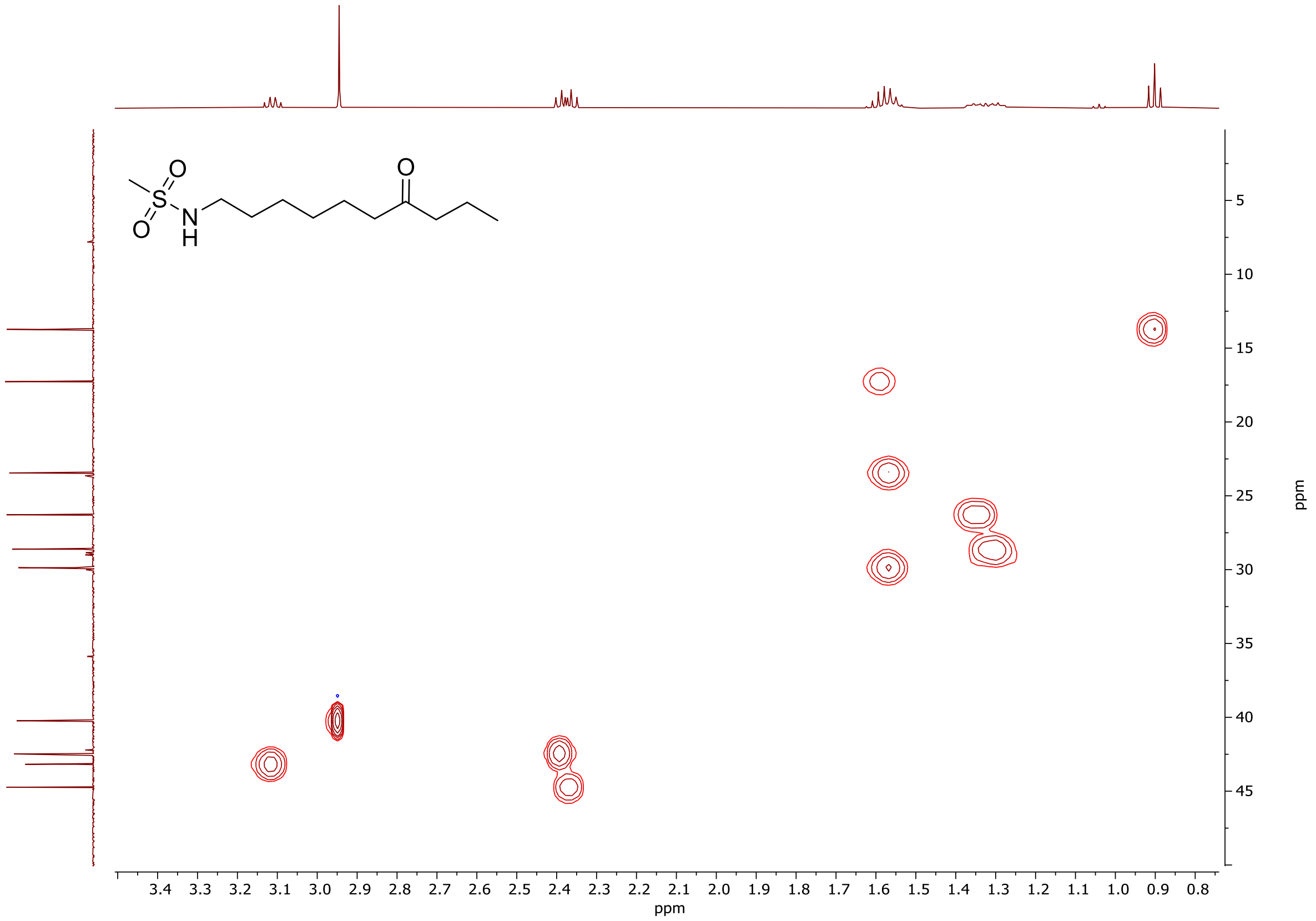

^{1}H-^{13}C HMBC ($CDCl_3$, 298K) of C4-oxidized **S12**

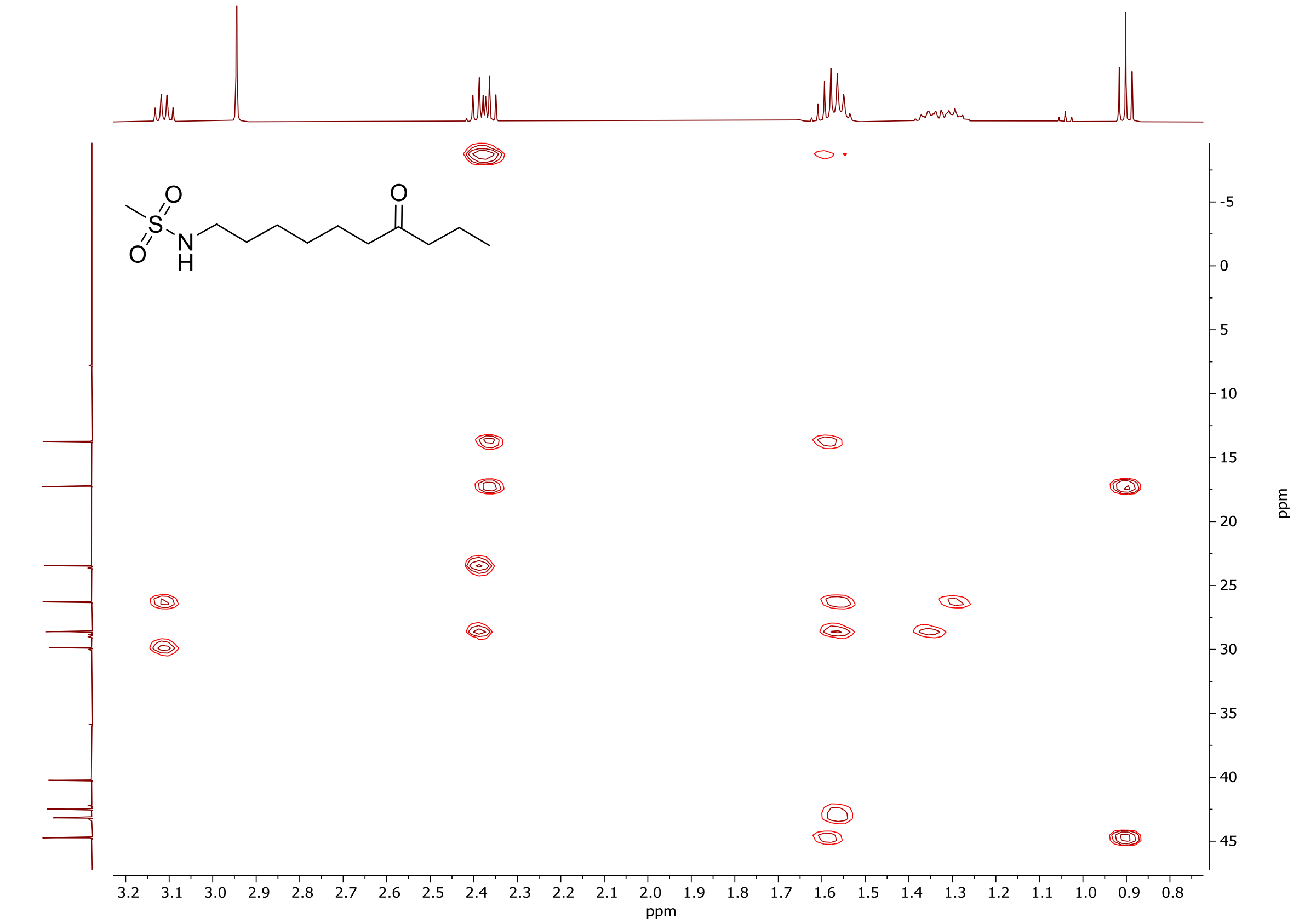

^{1}H NMR (500 MHz, $CDCl_3$, 298K) of C4-oxidized **S13**

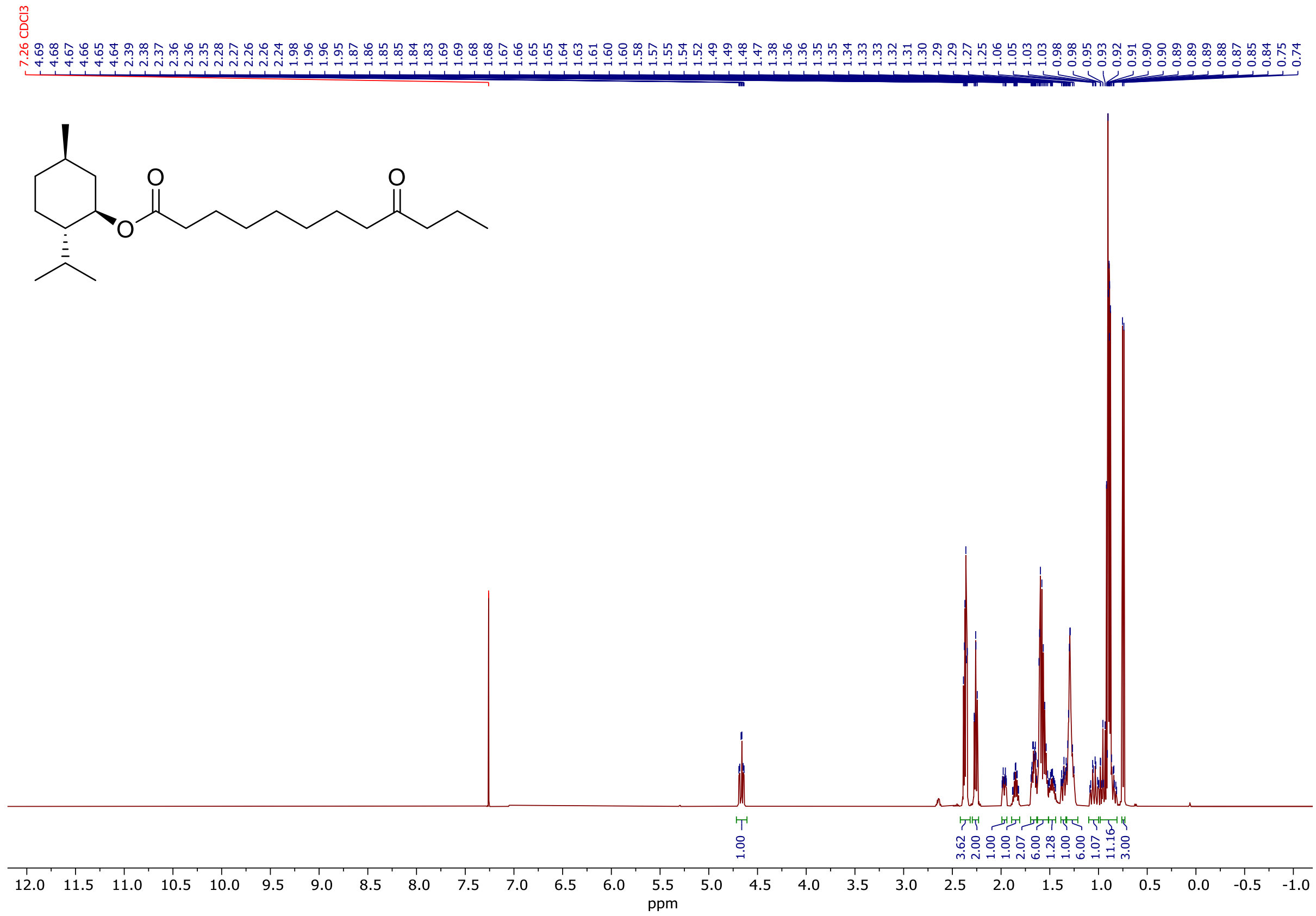

^{13}C NMR (125 MHz, $CDCl_3$, 298K) of C4-oxidized **S13**

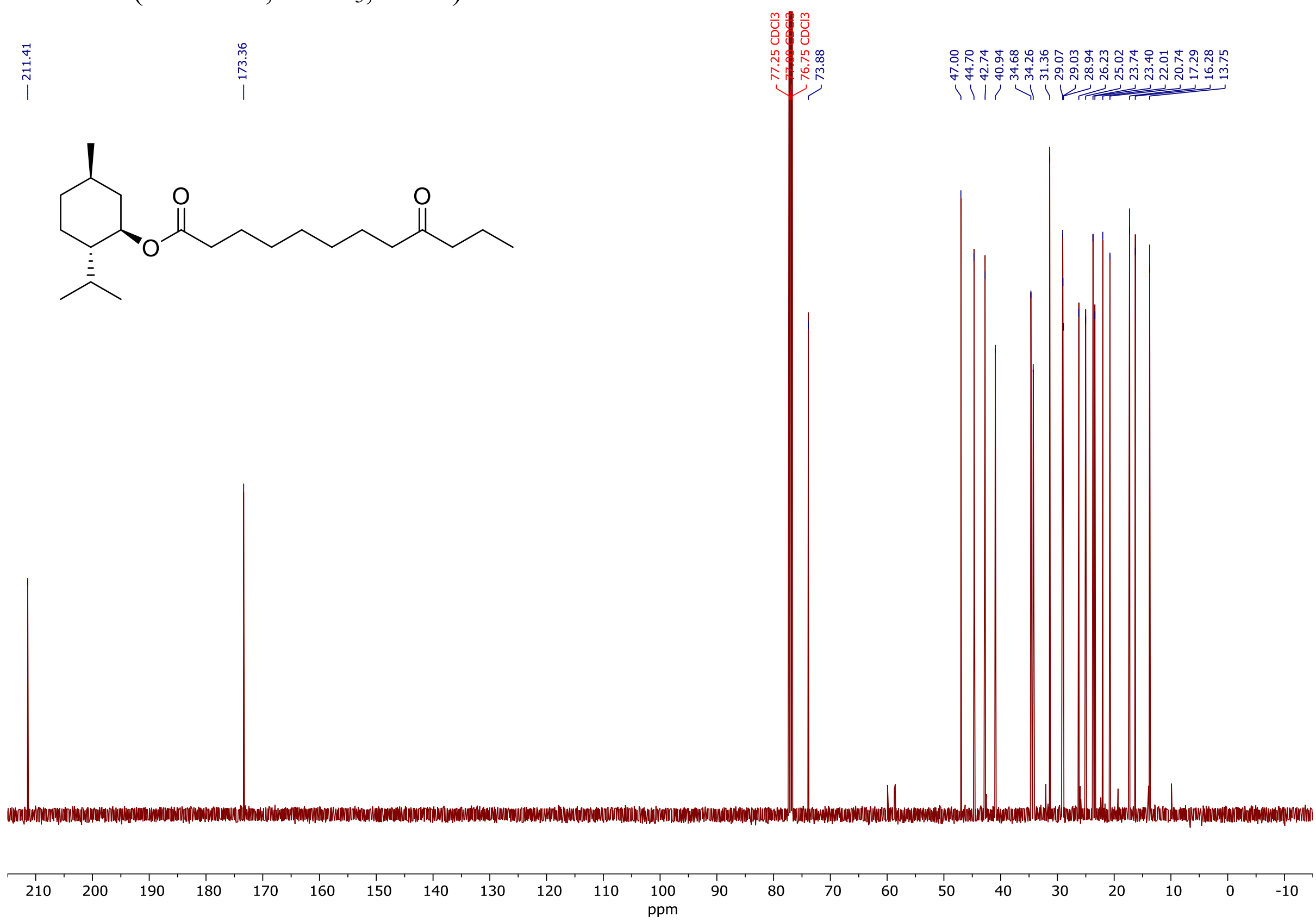

^{1}H-^{13}C HMQC ($CDCl_3$, 298K) of C4-oxidized **S13**

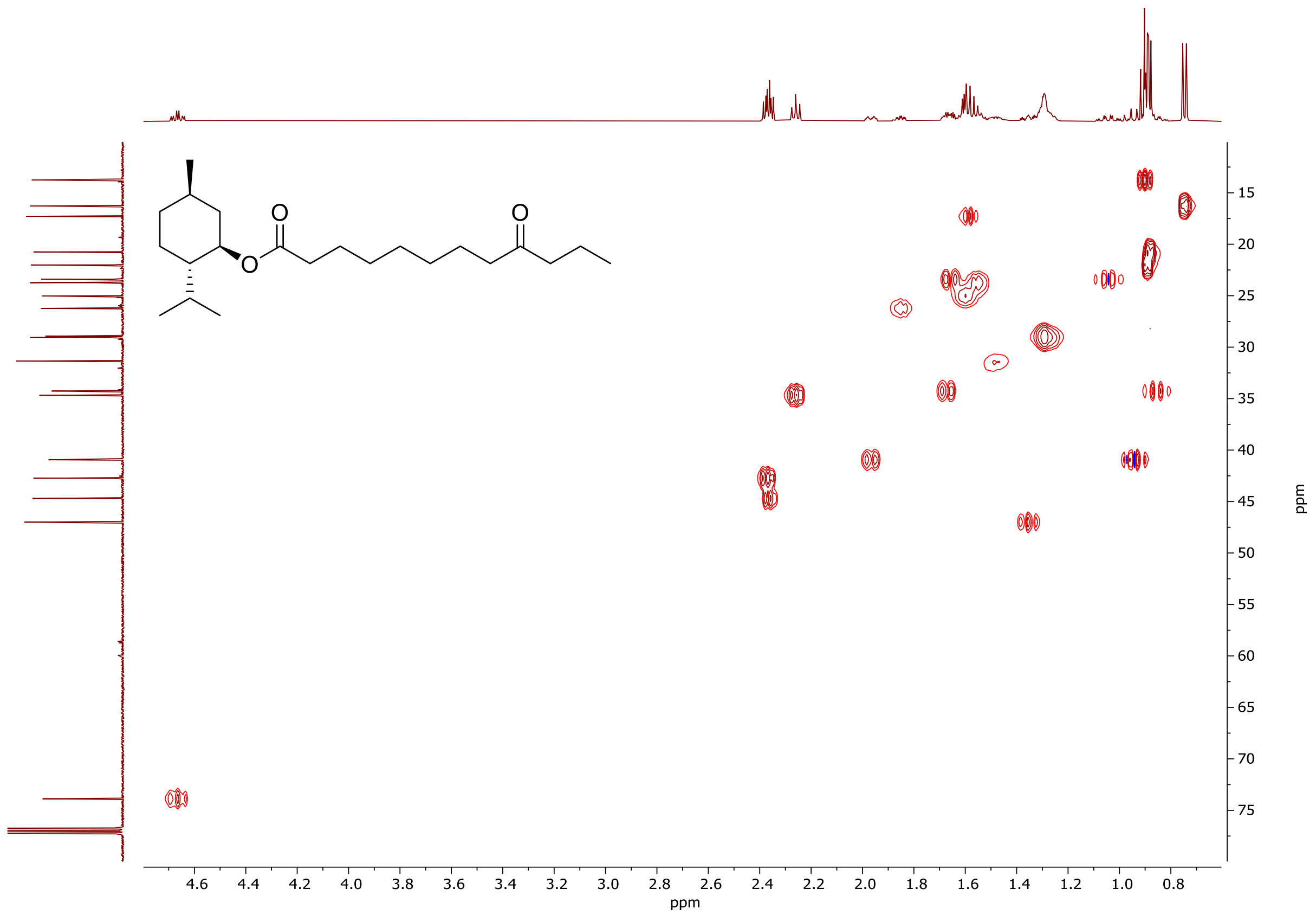

^{1}H-^{13}C HMBC ($CDCl_3$, 298K) of C4-oxidized **S13**

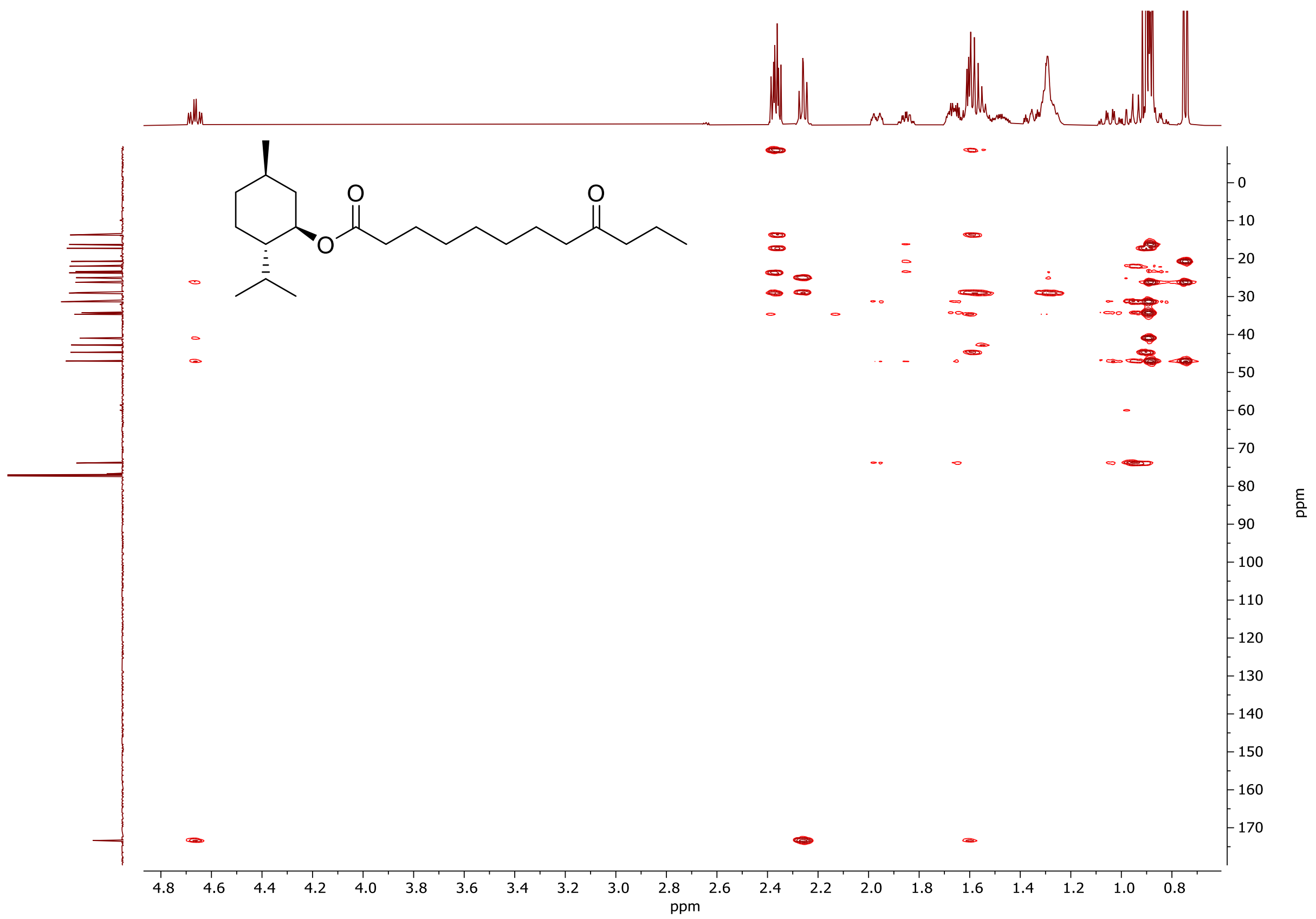

^{1}H NMR (500 MHz, $CDCl_3$, 298K) of C4-oxidized **S18**

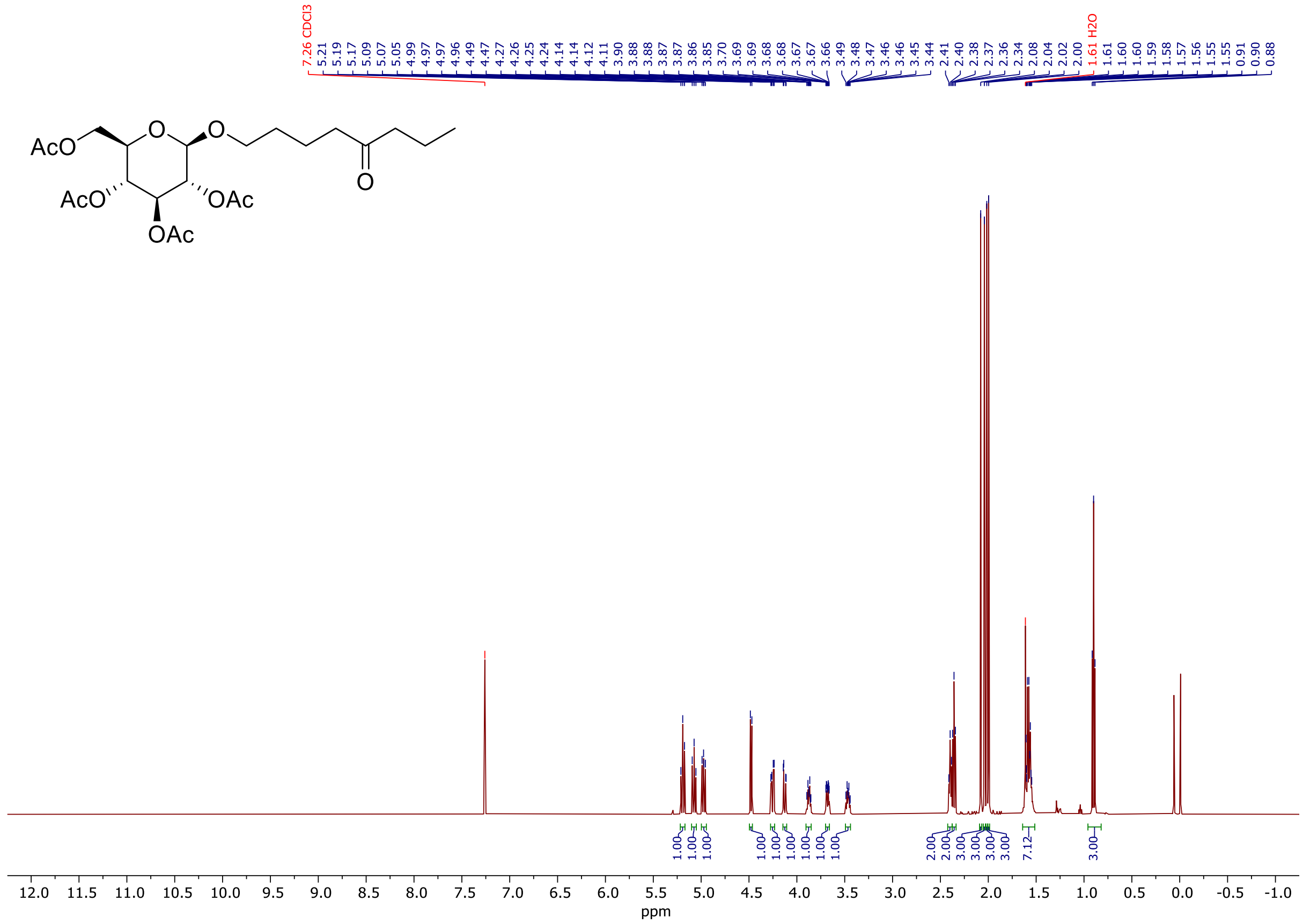

^{13}C NMR (150 MHz, $CDCl_3$, 298K) of C4-oxidized **S18**

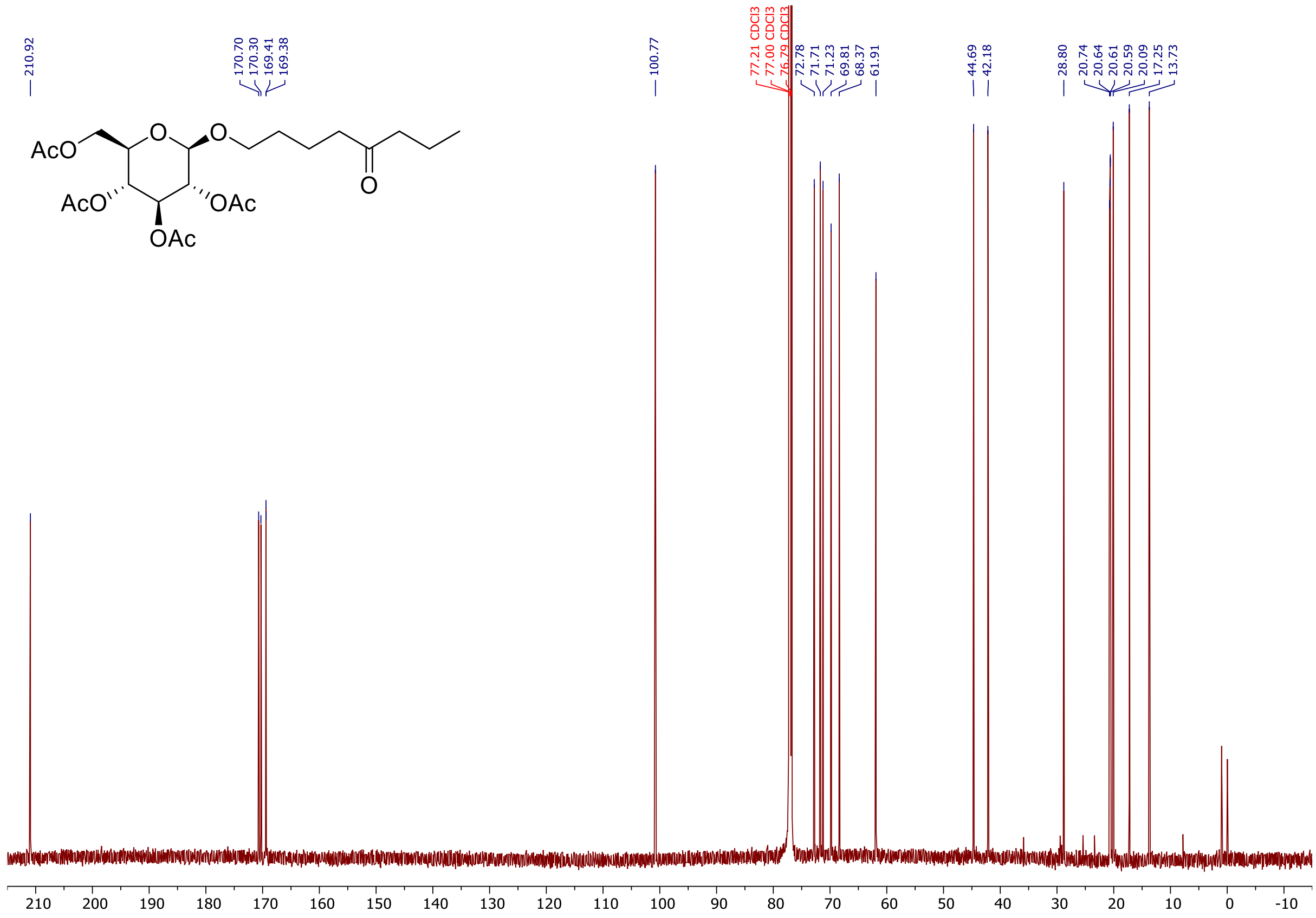

^{1}H-^{13}C HMQC ($CDCl_3$, 298K) of C4-oxidized **S18**

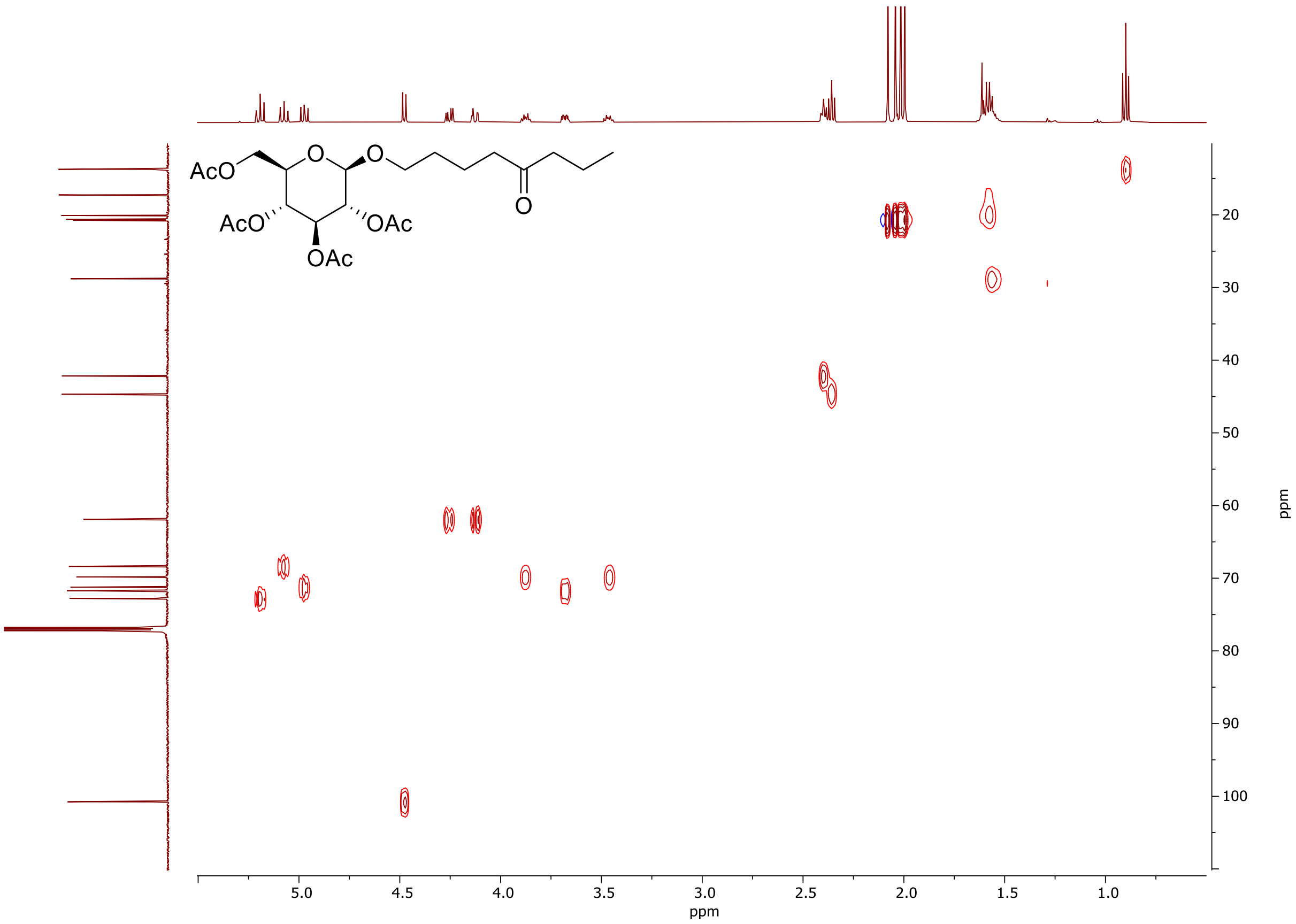

^{1}H-^{13}C HMBC ($CDCl_3$, 298K) of C4-oxidized **S18**

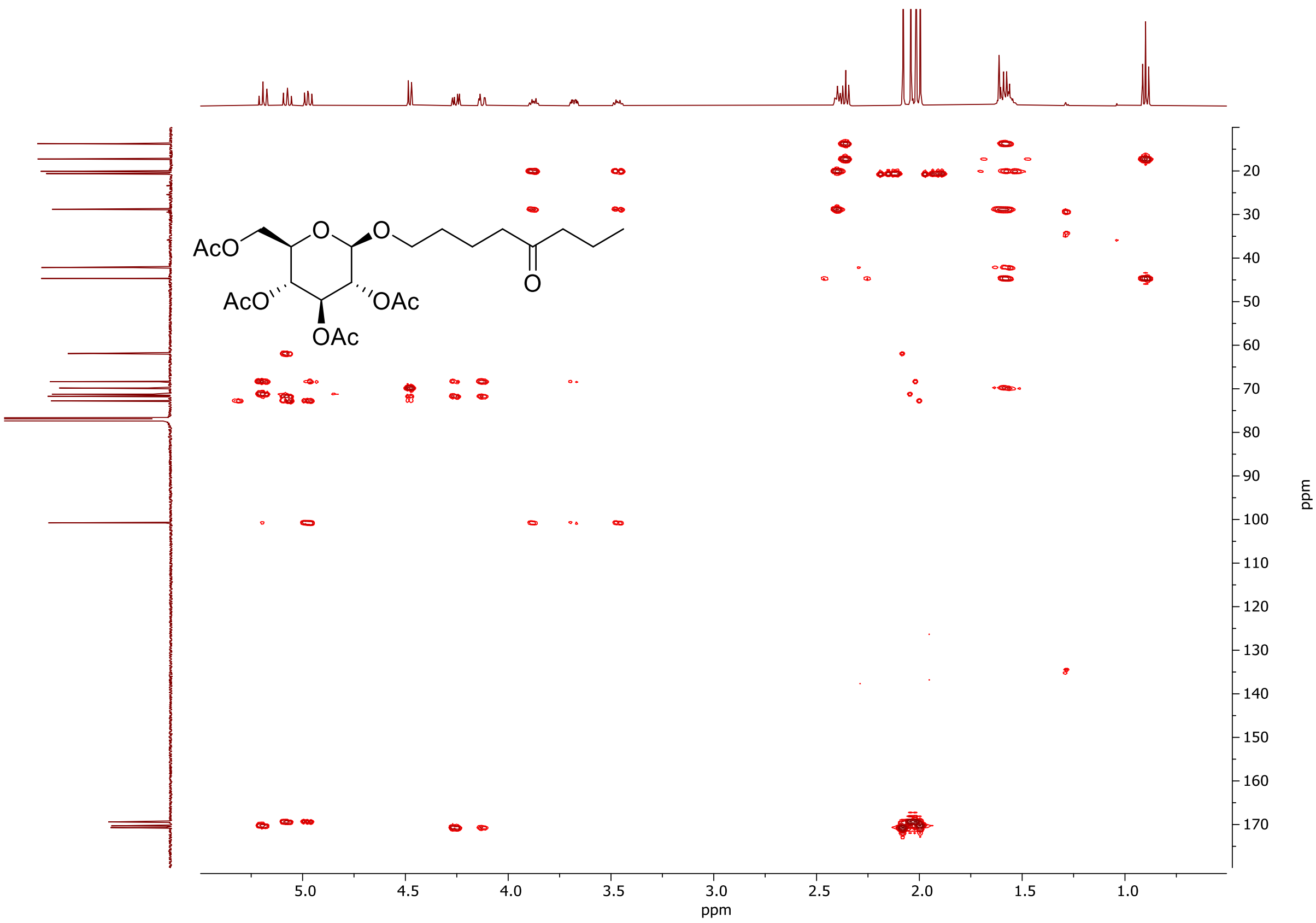

^{1}H NMR (500 MHz, $CDCl_3$, 298K) of C4-oxidized **S19**

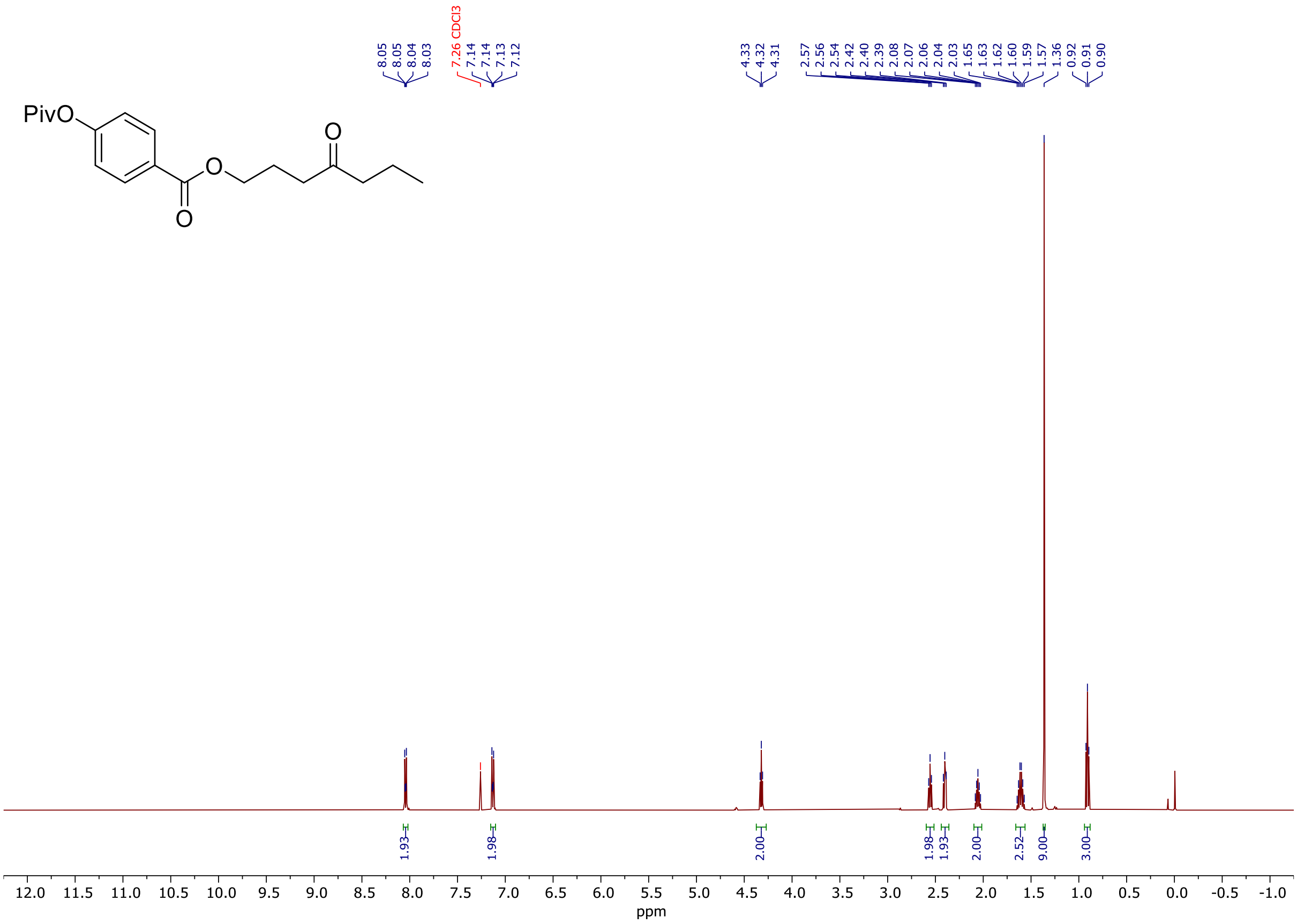

^{13}C NMR (125 MHz, $CDCl_3$, 298K) of C4-oxidized **S19**

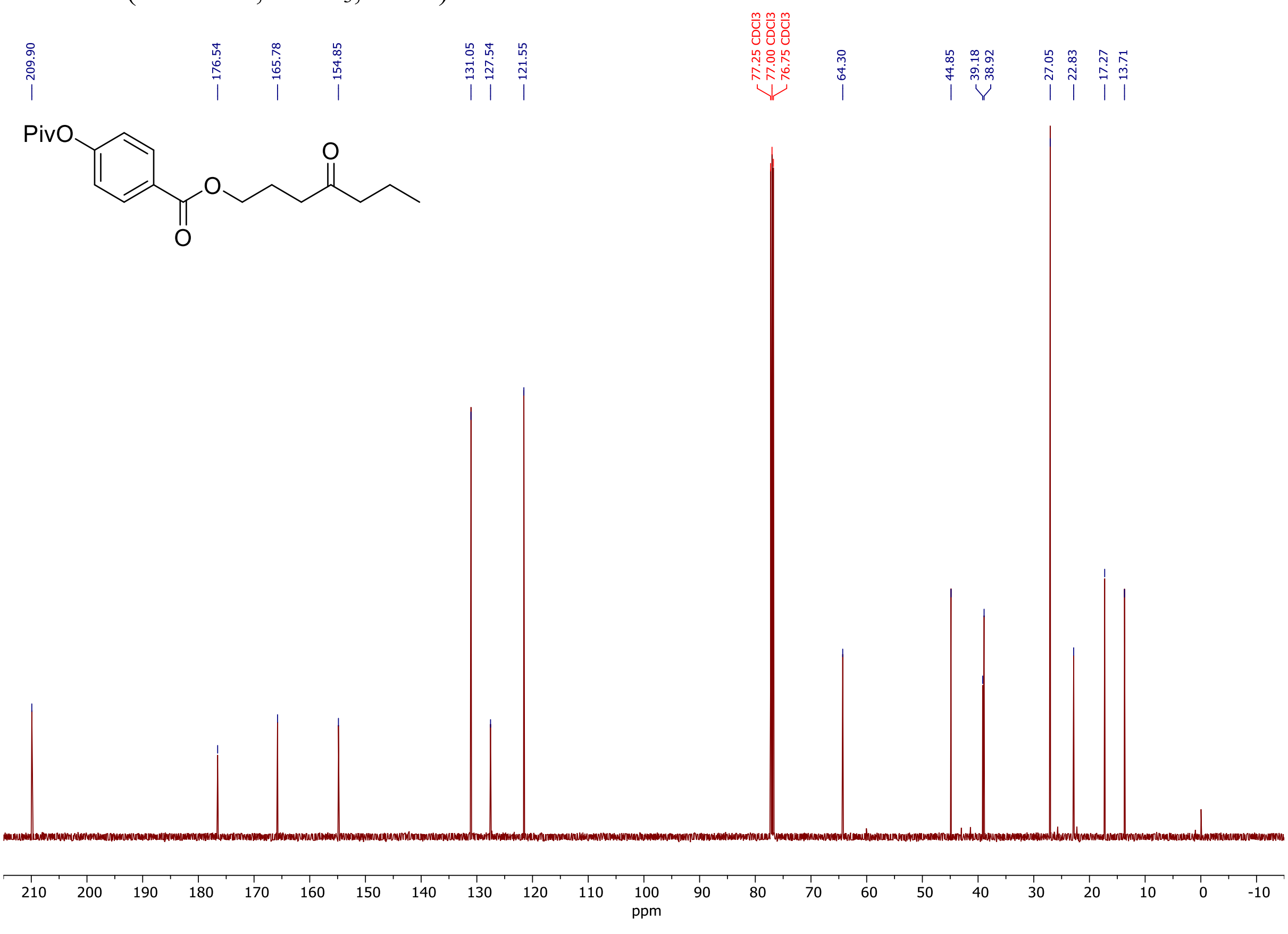

DEPT-135 (125 MHz, $CDCl_3$, 298K) of C4-oxidized **S19**

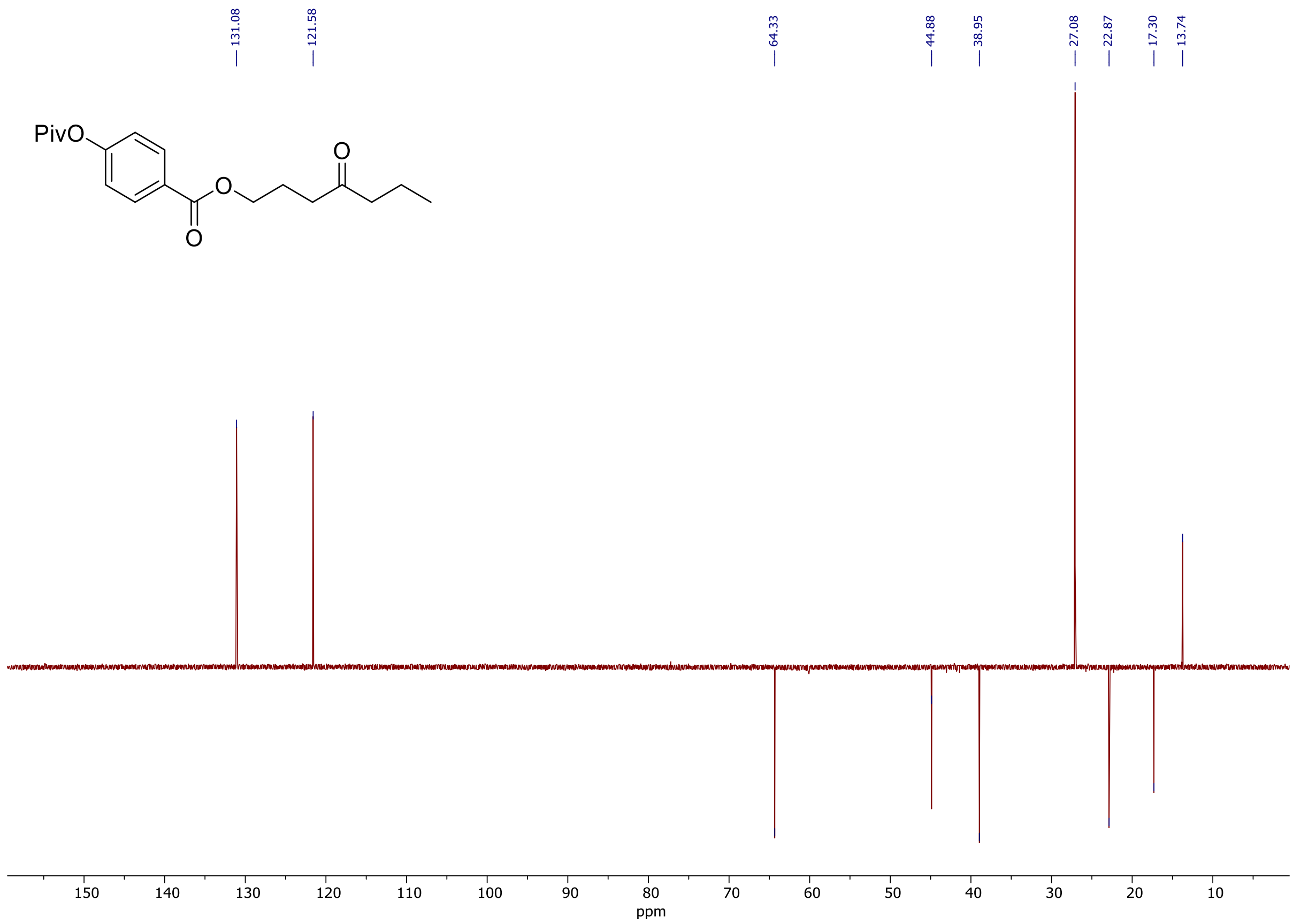

^1H-^{13}C HMQC ($CDCl_3$, 298K) of C4-oxidized **S19**

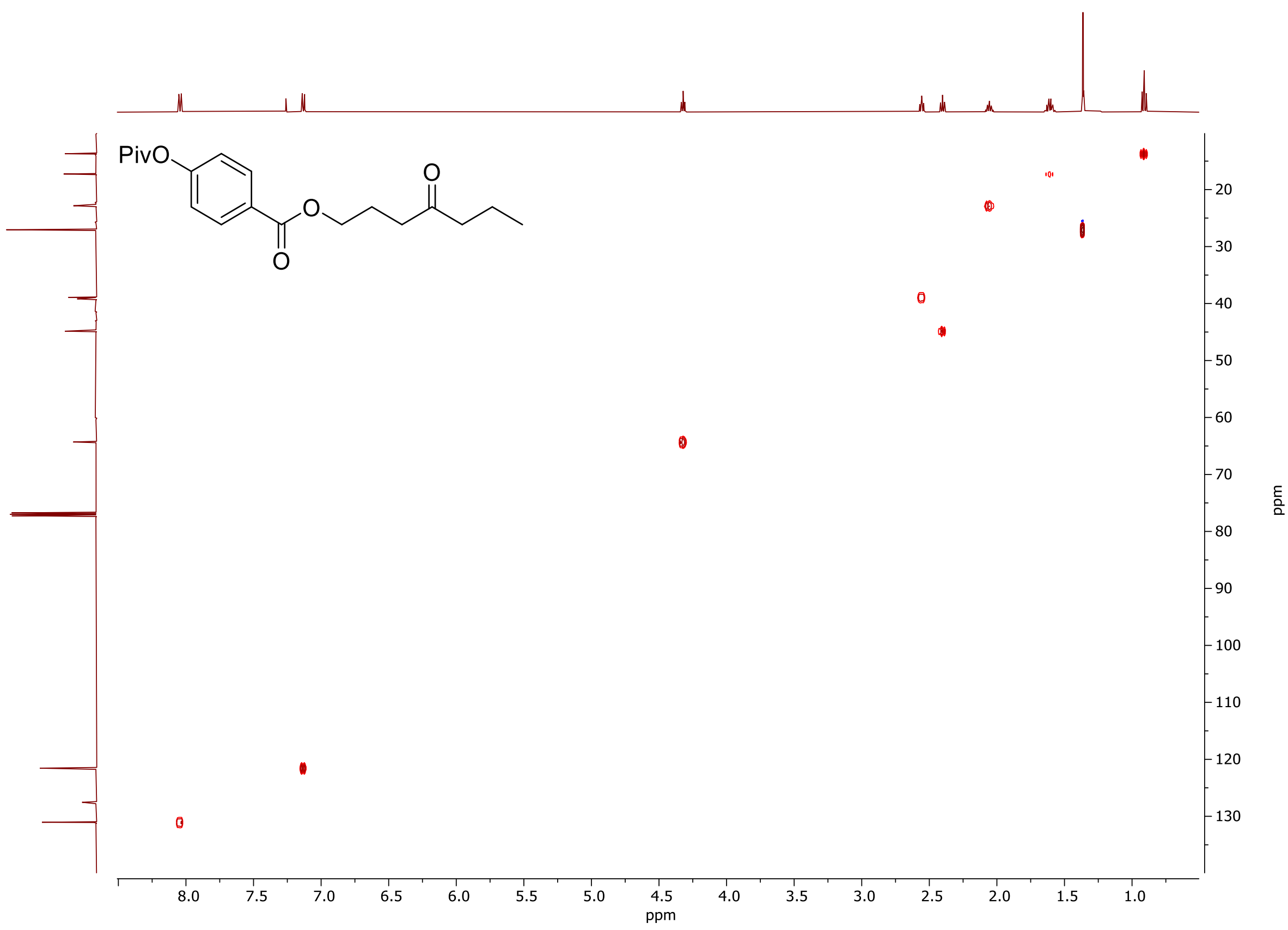

^{1}H-^{13}C HMBC ($CDCl_3$, 298K) of C4-oxidized **S19**

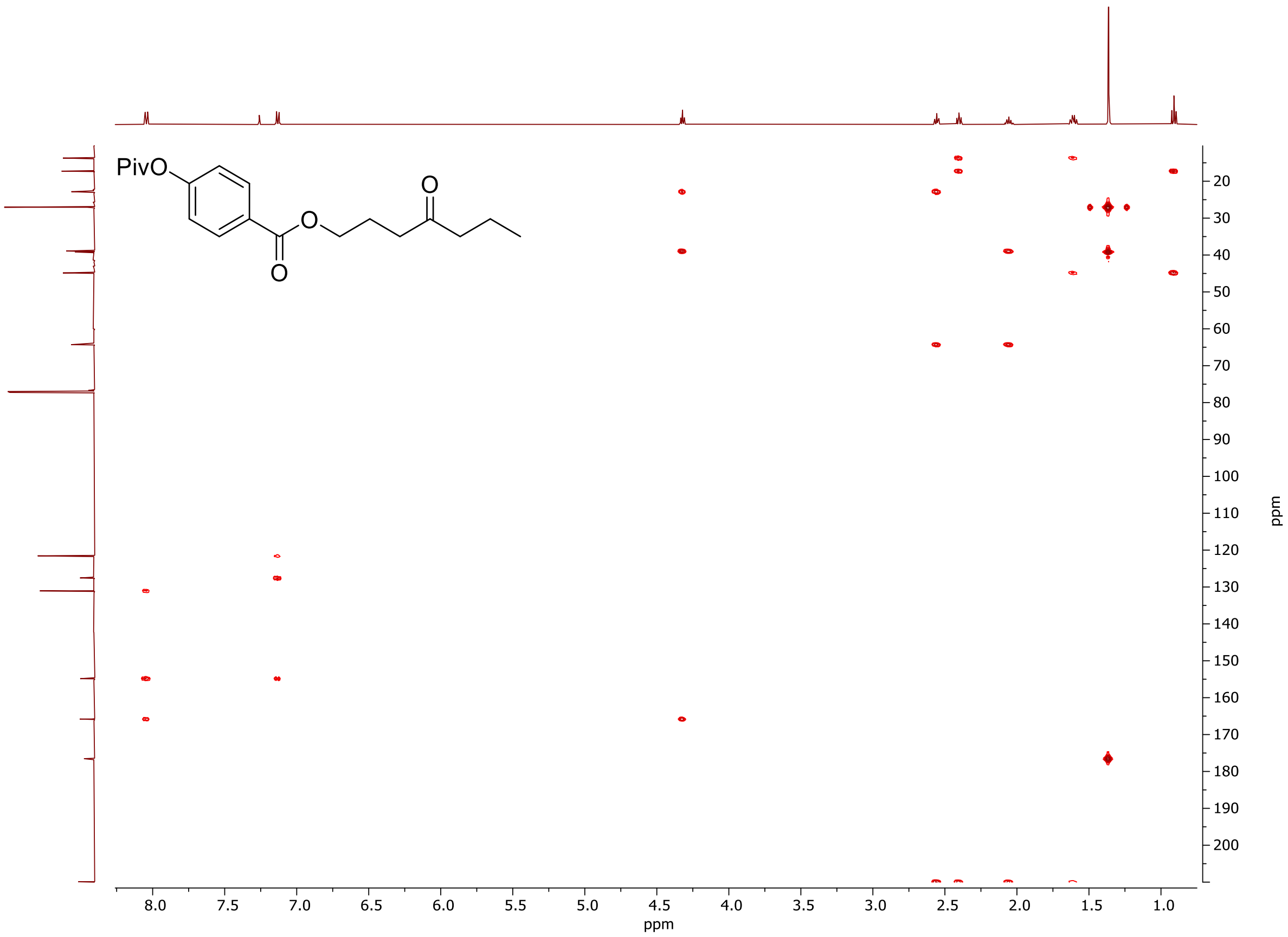

^{1}H NMR (500 MHz, $CDCl_3$, 298K) of C4-oxidized **S21**

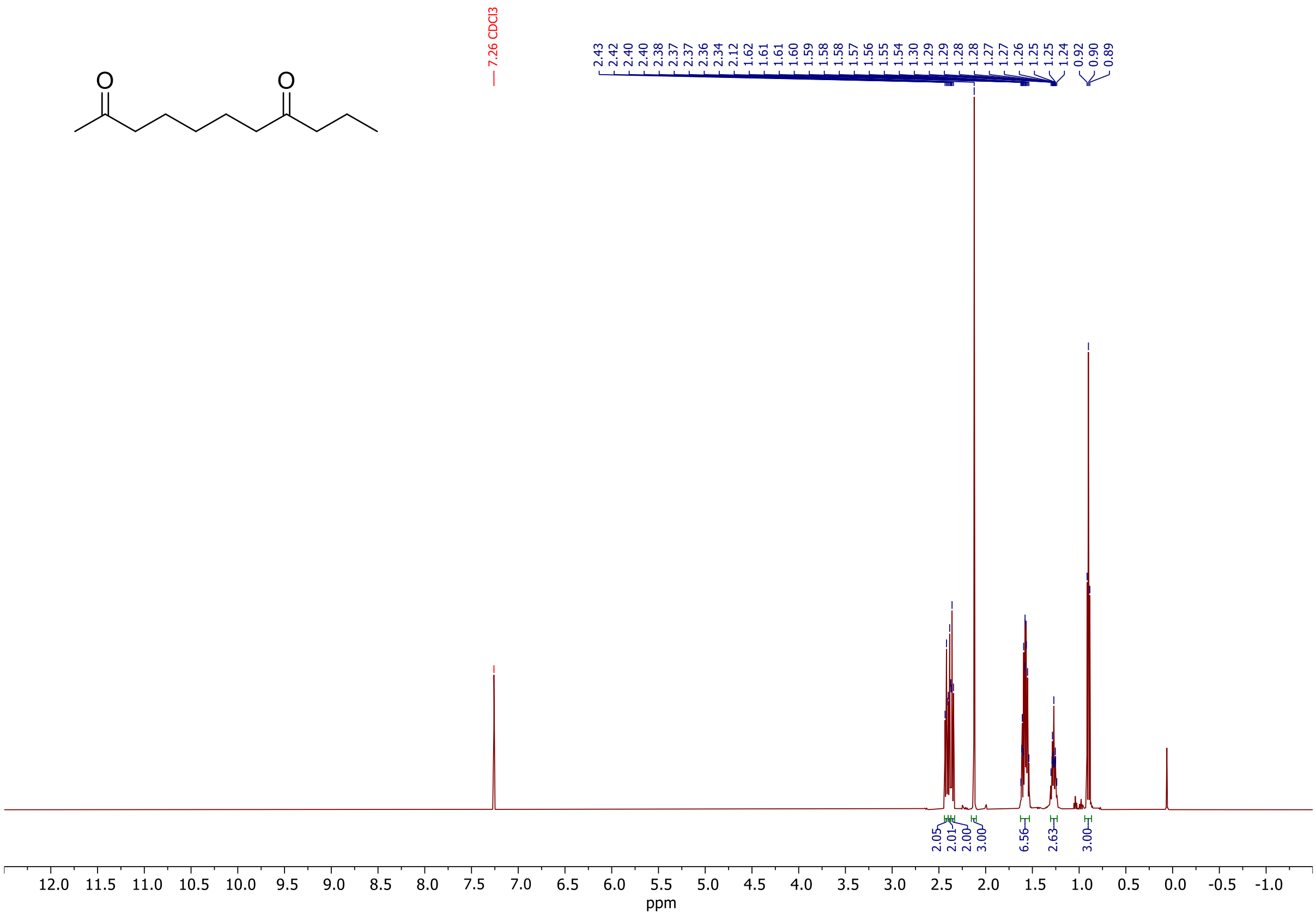

^{13}C NMR (125 MHz, $CDCl_3$, 298K) of C4-oxidized **S21**

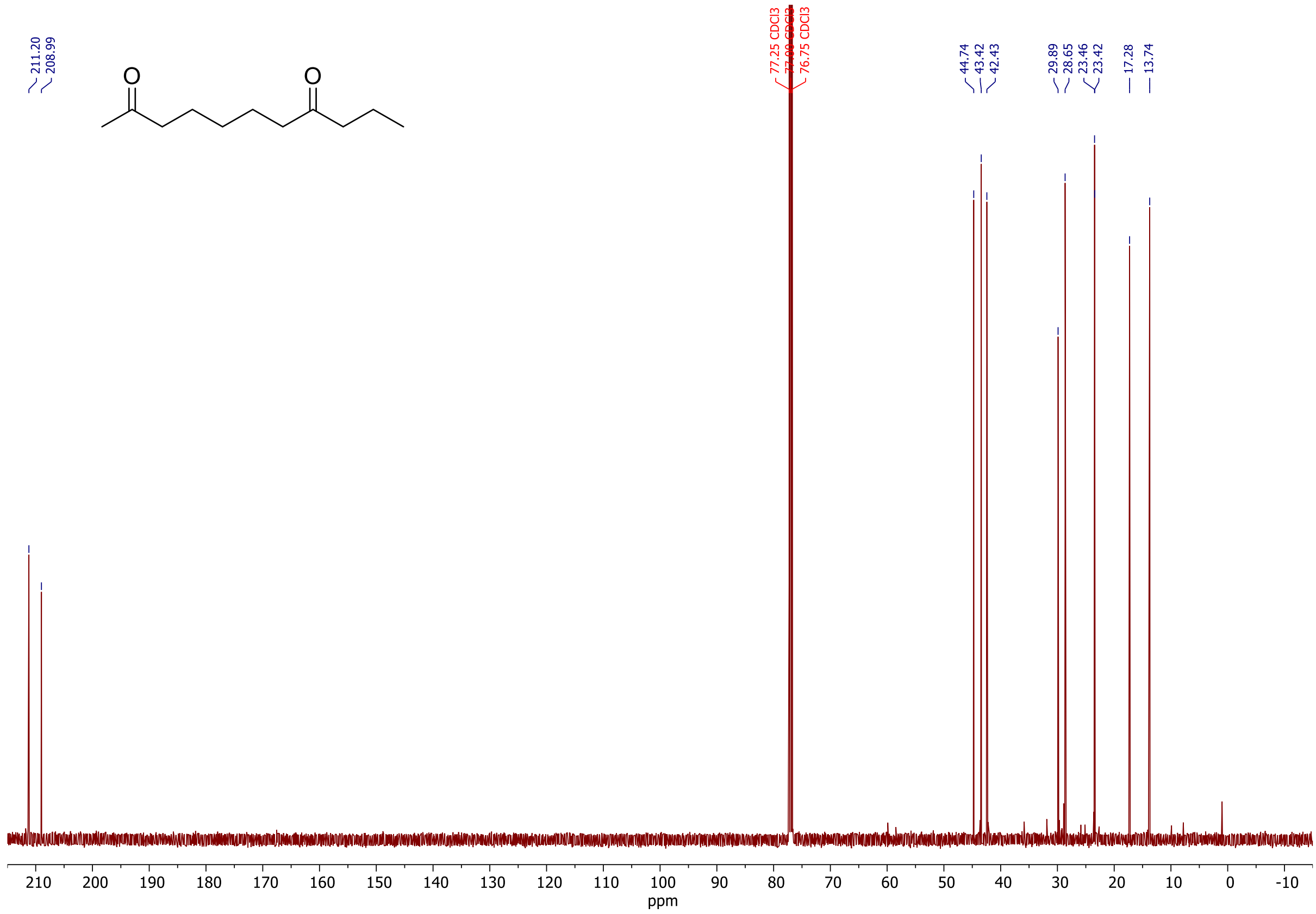

^{1}H-^{13}C HMQC ($CDCl_3$, 298K) of C4-oxidized **S21**

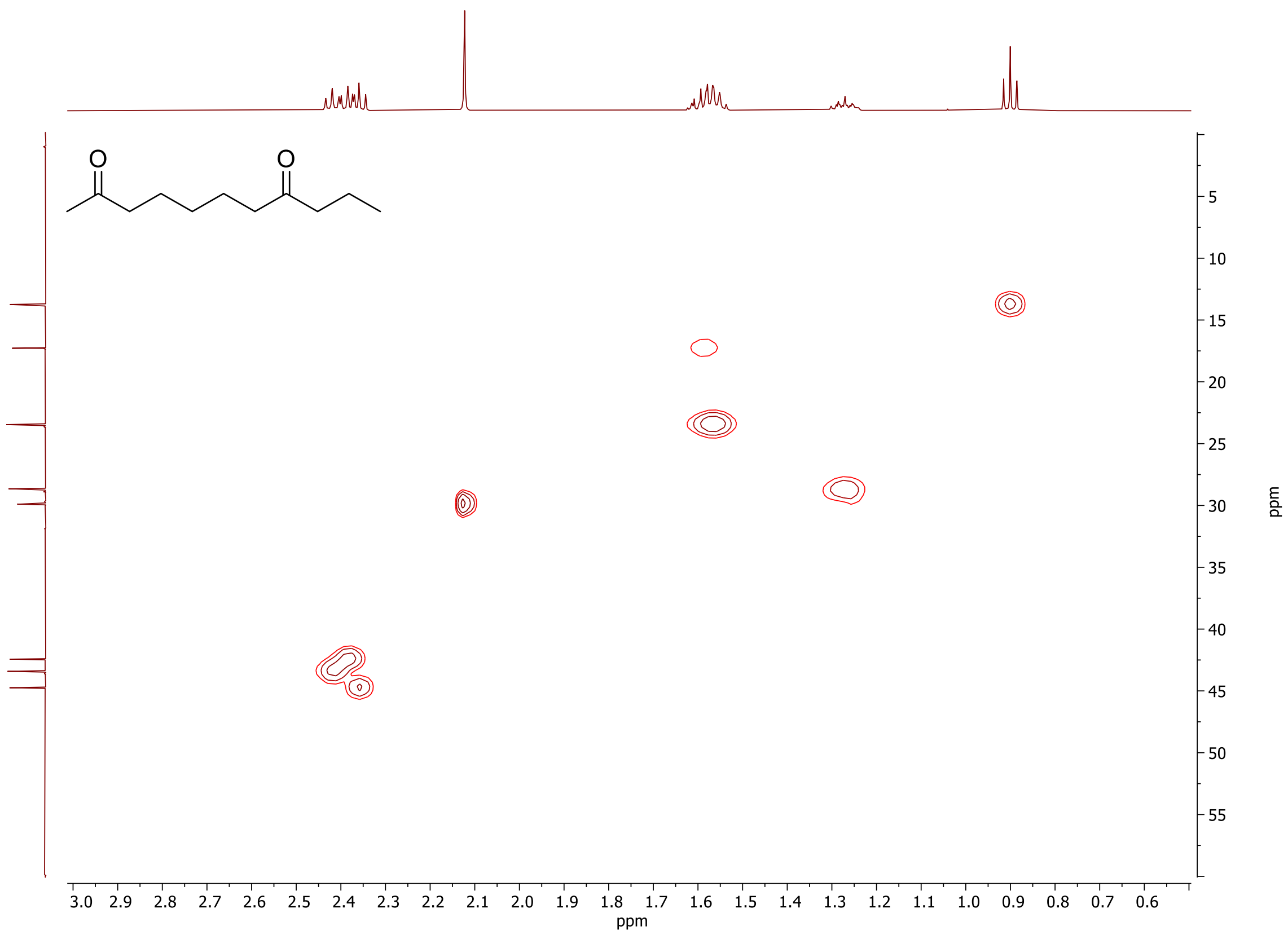

^{1}H-^{13}C HMBC ($CDCl_3$, 298K) of C4-oxidized **S21**

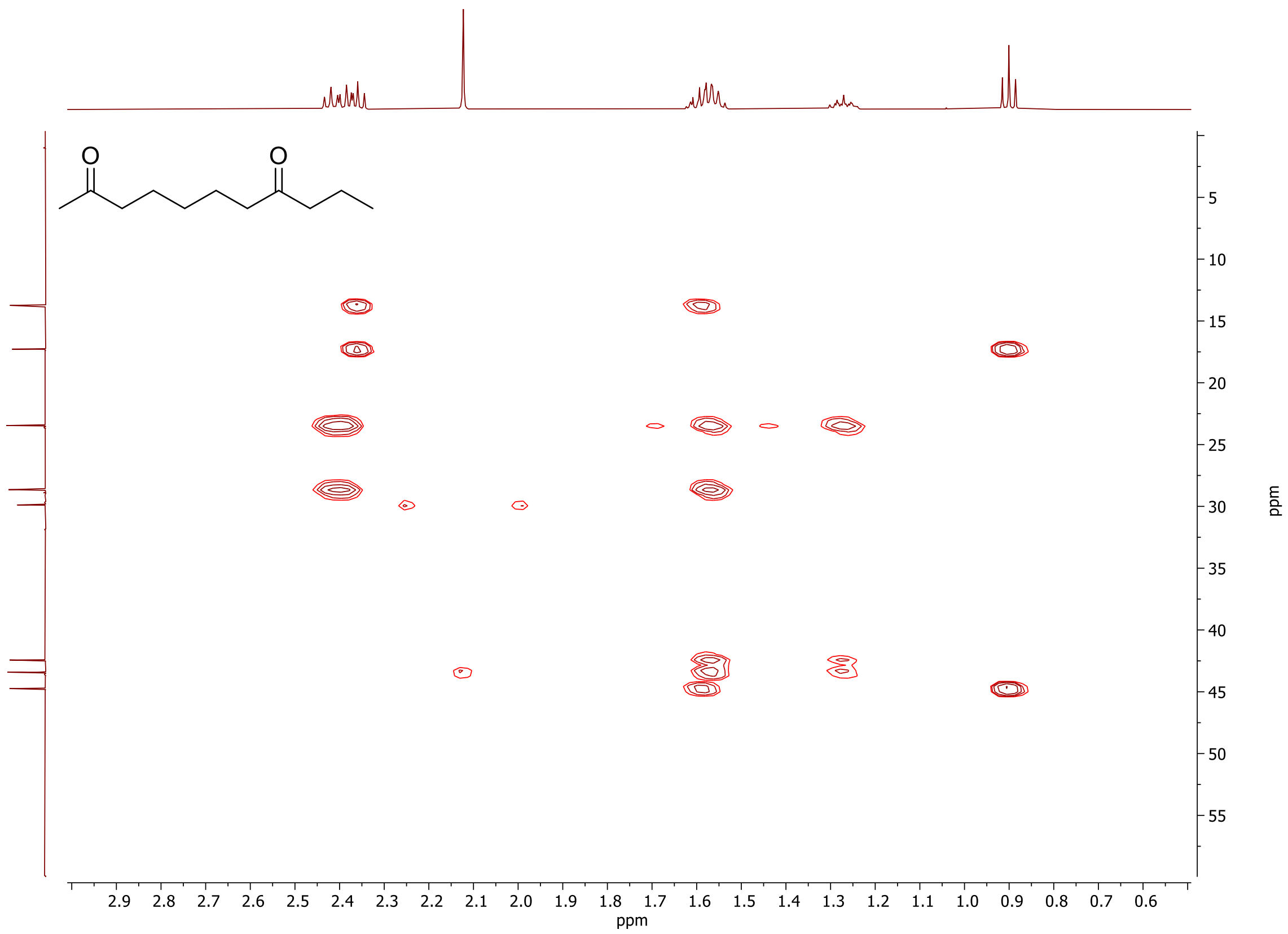

^{1}H NMR (500 MHz, $CDCl_3$, 298K) of C4-oxidized **S22**

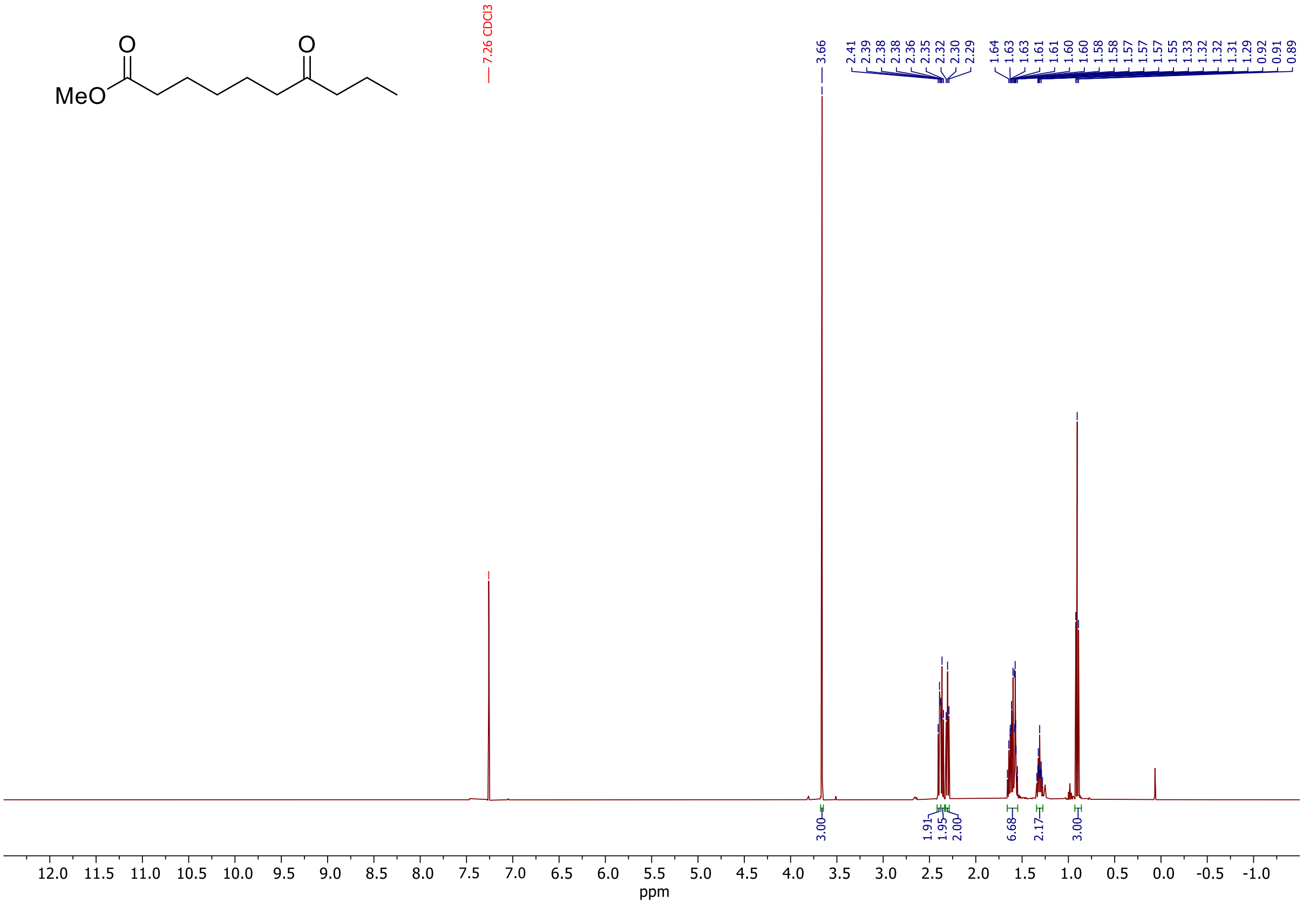

^{13}C NMR (125 MHz, $CDCl_3$, 298K) of C4-oxidized **S22**

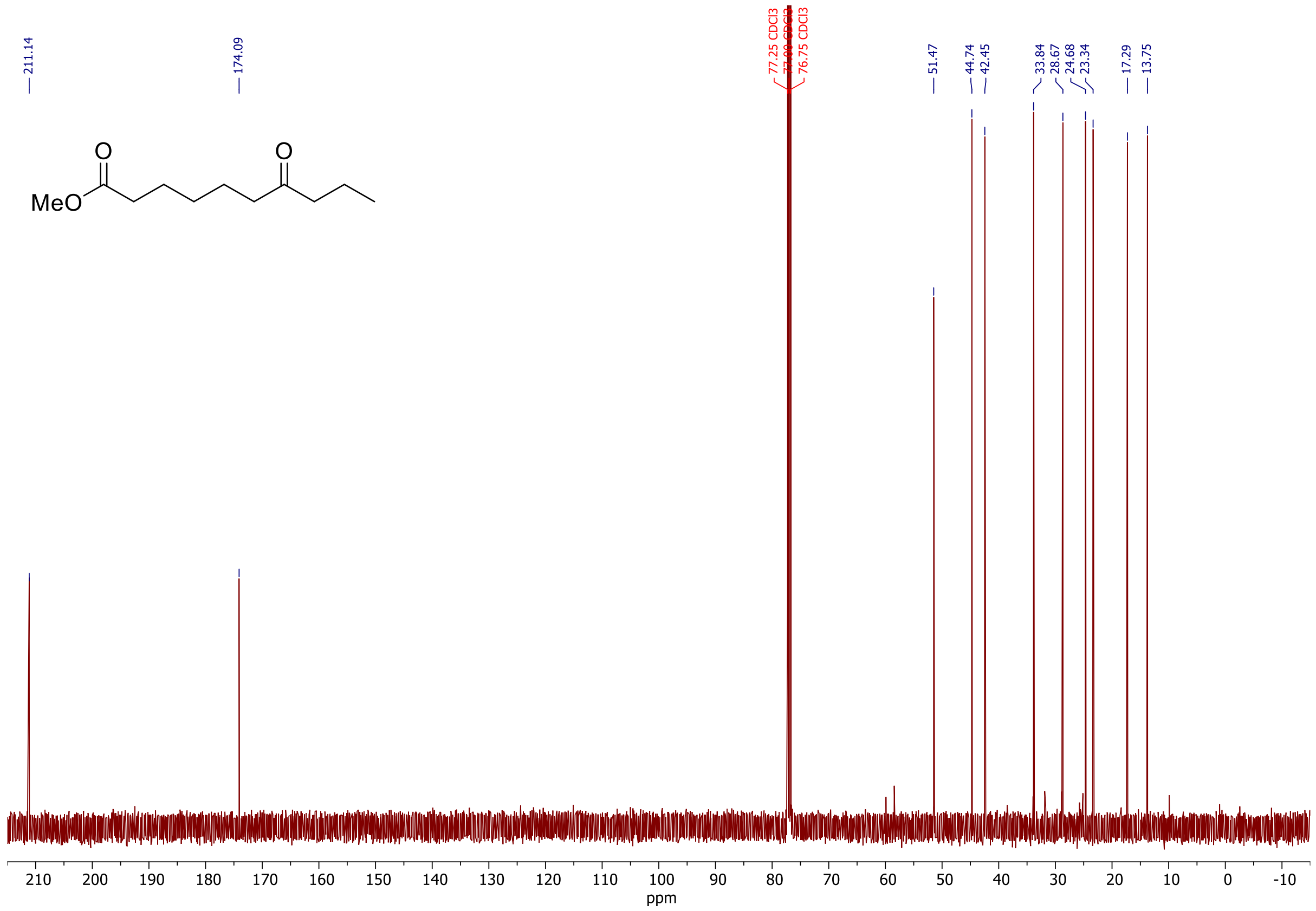

^{1}H-^{13}C HMQC ($CDCl_3$, 298K) of C4-oxidized **S22**

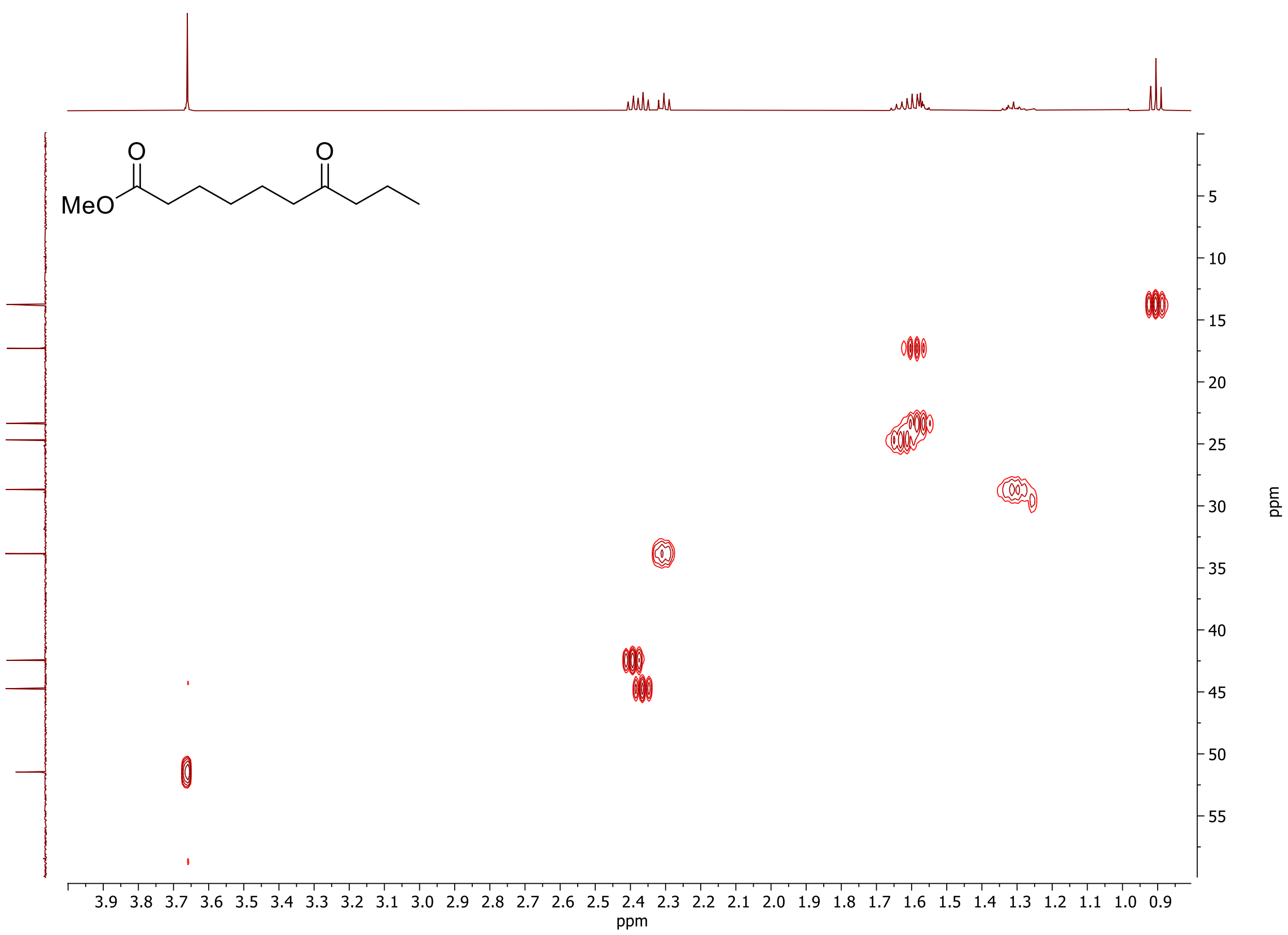

^{1}H-^{13}C HMBC ($CDCl_3$, 298K) of C4-oxidized **S22**

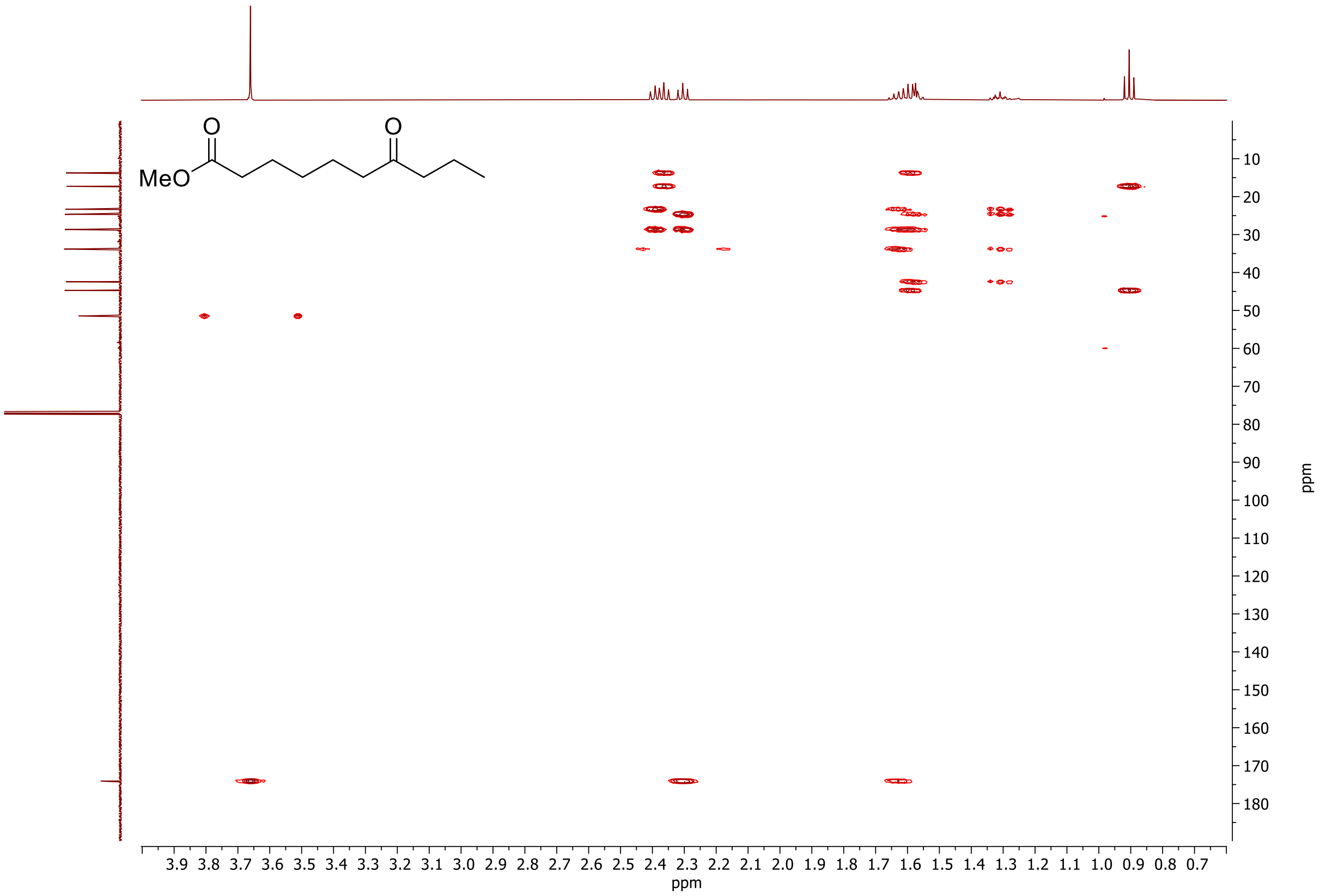

Supplement: Supplementary file 1 [file ja6c08837_si_001.pdf]
